# Fructose induces PERK-dependent ER stress and mitochondrial dysfunction via nucleotide depletion in clear cell renal cell carcinoma

**DOI:** 10.1038/s41419-026-09157-3

**Published:** 2026-08-07

**Authors:** Na Li, Yanhong Ding, Bowen Yu, Yongqiang Wang, Tongbin Gao, Huiyong Yin, Zhenhua Li, Shuzhen Liu, Guohua Yu, Ningning Wang

**Affiliations:** 1 First Affiliated Hospital, Weifang People’s Hospital, Shandong Second Medical University, Weifang, 261041 China; 2 Key Laboratory of Immune Microenvironment and Inflammatory Disease Research in Universities of Shandong Province, School of Basic Medical Sciences, Shandong Second Medical University, Weifang, 261053 China; 3 Frontier Medical Center, Tianfu Jincheng Laboratory, Chengdu, 611730 China; 4 West China School of Basic Medical Sciences and Forensic Medicine, Sichuan University, Chengdu, 610041 China; 5 Department of Biomedical Sciences, College of Biomedicine, Institute of Digital Medicine, Tung Biomedical Science Center, The Shenzhen Research Institute and Futian Research Institute, City University of Hong Kong, Hong Kong, China

**Keywords:** Urological cancer, Cancer metabolism

## Abstract

Clear cell renal cell carcinoma (ccRCC) exhibits a paradoxical fructose metabolism signature characterized by upregulation of the fructose transporter GLUT5 alongside downregulation of the catabolic enzymes (ketohexokinase, aldolase B, and triokinase), a pattern associated with poor prognosis. Functionally, unlike the pro-survival effect of fructose under glucose deprivation, in the presence of glucose, fructose co-treatment suppresses ccRCC cell proliferation, and induces profound mitochondrial dysfunction, including impaired oxidative phosphorylation, loss of membrane potential, excessive mitochondrial superoxide production, reduced mtDNA copy number, and downregulation of mitochondria-encoded electron transport chain subunits (notably ND2 and ND4 of complex I). Mechanistically, co-treatment with glucose and fructose creates a metabolic trap resulting in fructose-1-phosphate accumulation and ATP depletion. This energy crisis drives profound depletion of purine and pyrimidine nucleotide pools, which selectively triggers the PERK-eIF2S1-ATF4-CHOP axis of the integrated stress response, thereby mediating mitochondrial impairment and ultimately sensitizing ccRCC cells to intrinsic apoptosis via BID cleavage and caspase-3 activation under nutrient stress. Pharmacological treatment with the chemical chaperone 4-phenylbutyric acid (4-PBA) or nucleoside supplementation reverses mitochondrial dysfunction and fructose-induced cytotoxicity. The tumor-suppressive effect of fructose is validated in patient-derived organoids and xenograft mouse models, where fructose administration significantly attenuates tumor growth via ER stress. These findings reveal fructose-driven nucleotide depletion and PERK-dependent ER stress leading to mitochondrial dysfunction, which underlies the tumor-suppressive toxicity of fructose and exposes a targetable metabolic vulnerability in ccRCC.

## Introduction

Renal cell carcinoma (RCC) accounts for approximately 2–3% of global cancer-related mortality [1], with clear cell RCC (ccRCC) representing the predominant histopathological subtype originating from proximal renal tubular epithelial cells. A hallmark of ccRCC is its profound metabolic reprogramming, which encompasses the classical Warburg effect and extensive rewiring in fatty acid and amino acid metabolism [2].

Fructose consumption has increased dramatically since the 1970s, predominantly driven by the widespread addition of high-fructose corn syrup (HFCS, containing 42–55% fructose) as a cost-effective sweetener in processed foods [3]. Most dietary fructose is metabolized in the small intestine and liver [4], with less than 0.8 mM entering systemic circulation [5]. In the kidneys, fructose is filtered by the glomerulus and then reabsorbed in the proximal tubule via glucose transporter 5 (GLUT5) and sodium-glucose cotransporter 5 (SGLT5) on the apical membrane [6]. Intracellular fructose is phosphorylated by the rate-limiting enzyme ketohexokinase (KHK) to fructose-1-phosphate (F1P) and then cleaved by aldolase B (ALDOB) into dihydroxyacetone phosphate (DHAP) and glyceraldehyde (GA). GA is subsequently phosphorylated by triokinase (TKFC) to form glyceraldehyde-3-phosphate (GAP). These three-carbon intermediates can enter into glycolysis, gluconeogenesis, and tricarboxylic acid cycle (TCA cycle) [7]. Under physiological conditions, renal fructose is predominantly utilized as a substrate for gluconeogenesis to maintain systemic glucose homeostasis [8]. However, in pathological states such as diabetes mellitus, metabolic acidosis, and ischemia–reperfusion injury, the renal polyol pathway is markedly upregulated, leading to substantial endogenous fructose production from glucose [8]. Excessive fructose intake or aberrant endogenous production can promote renal tubular injury [9], inflammation [10], and oxidative stress [8, 11] through its metabolism.

Accumulating evidence links excessive fructose intake to cancer progression in malignancies such as acute myeloid leukemia (AML) [12, 13], intestinal tumor [14], breast cancer [15], and lung cancer [16]. In these contexts, fructose often serves as an alternative fuel source to support tumor proliferation, particularly under glucose scarcity. In contrast, several studies suggest fructose may exert tumor-suppressive effects. In hepatocellular carcinoma (HCC), KHK-mediated fructose metabolism is lost in HCC cells compared with normal hepatocytes [17], and fructose induces apoptosis in HCC cells [18]. Furthermore, Zhang et al. reported that fructose enhances leptin release from adipocytes, which in turn amplifies leptin-dependent CD8^+^ T-cell responses and restrains lung-tumor growth [19]. These disparate findings indicate that the role of fructose in tumorigenesis is context-dependent and cannot be generalized.

Current research regarding the impact of fructose metabolism in ccRCC remains limited and inconclusive. Existing studies have found a positive correlation between GLUT5 expression and moderately differentiated RCC [20, 21]. Under glucose deprivation, ccRCC cells can utilize fructose via GLUT5 to sustain proliferation [22, 23]. Controversially, paired clinical studies demonstrate 1.4-fold reduction in KHK expression and corresponding enzymatic activity in tumor tissues, suggesting impaired fructose catabolic capacity [24]. This metabolic paradox is further complicated by the finding that ccRCC cells maintain elevated fructose-1,6-bisphosphate (FBP) levels to suppress NOX4-driven oxidative stress while simultaneously downregulating ALDOB to enhance proliferative signaling. Clinically, low ALDOB expression correlates with poorer disease-free and overall survival in ccRCC patients [11]. The apparent discordance between enhanced fructose uptake (GLUT5 upregulated) and constrained metabolic utilization (KHK/ALDOB downregulated) underscores a critical knowledge gap regarding the functional consequences and regulatory mechanisms of fructose metabolism in ccRCC pathogenesis.

In this study, we resolve the paradox of fructose handling in ccRCC. We found that the discordance between GLUT5-driven fructose uptake and KHK/ALDOB-mediated fructose catabolic deficiency creates a metabolic trap associated with poor patient prognosis. Mechanistically, fructose co-treatment with glucose attenuates mitochondrial oxidative phosphorylation and electron transport chain expression, leading to comprehensive mitochondrial dysfunction and sensitization to intrinsic apoptosis in ccRCC cells. These effects are initiated by nucleotide pool depletion and mediated by PERK-dependent ER stress, and can be rescued by the chemical chaperone 4-phenylbutyric acid (4-PBA) both in vitro and in vivo. Our findings elucidate a novel PERK-dependent ER stress-mitochondrial quality control pathway that underlies the tumor-suppressive effects of fructose toxicity in ccRCC and reveal a targetable metabolic vulnerability contingent on the tumor’s intrinsic fructose catabolic deficiency.

## Results

### Fructose related metabolic genes are downregulated and associated with poor prognosis in ccRCC

**Fig. 1:**
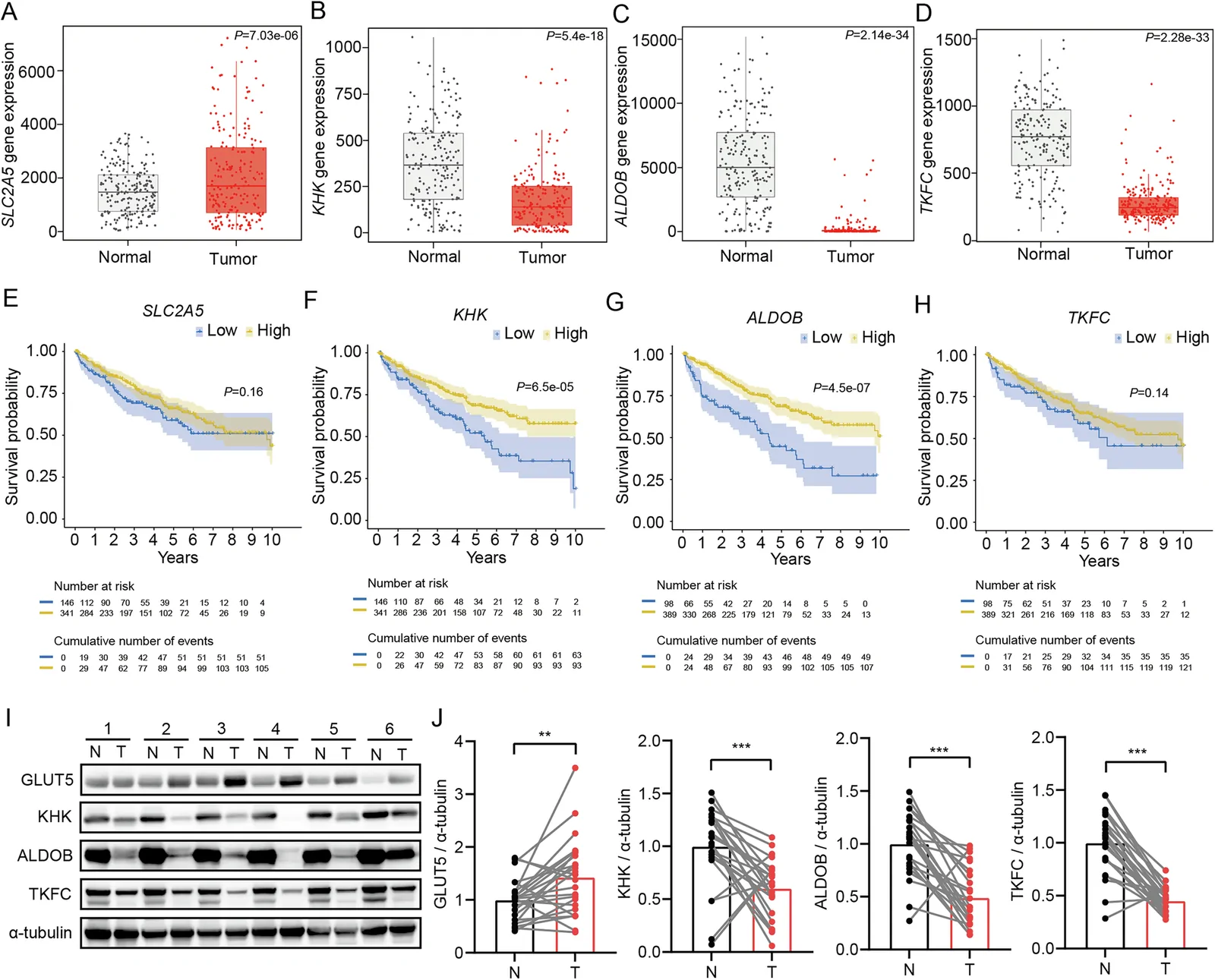
Fructose related metabolic genes are downregulated and associated with poor prognosis in ccRCC. Gene expression levels of *SLC2A5* (**A**), *KHK* (**B**), *ALDOB* (**C**), and *TKFC* (**D**) in paired tumor and adjacent normal tissues in kidney cancer, based on gene chip data from TNMplot (https://tnmplot.com/analysis/). Kaplan-Meier overall survival analysis of *SLC2A5* (**E**), *KHK* (**F**), *ALDOB* (**G**), and *TKFC* (**H**) expression in ccRCC patients using TCGA data from cSurvival (https://tau.cmmt.ubc.ca/cSurvival/). **I** Representative Western blots of proteins involved in fructose metabolism in paired tumor (T) and adjacent normal (N) tissues from ccRCC patients (6 of 24 pairs shown; see Supplementary Fig. 1 for all). **J** Quantification of the indicated proteins in paired tumor and adjacent normal tissues from all 24 paired ccRCC patients. Data are presented as mean ± SEM. *P* values were determined by two-tailed paired *t* test (**J**). **P < 0.01; ***P < 0.001.

To systematically assess fructose metabolism in ccRCC, we analyzed the expression of key fructose metabolic genes based on gene chip data from TNMplot (https://tnmplot.com/analysis/) in paired tumor and adjacent normal tissues, which revealed a significant upregulation of *SLC2A5* (the gene encoding GLUT5) in tumors, while *KHK*, *ALDOB*, and *TKFC* (the genes of metabolic enzymes) were consistently downregulated (Fig. 1A–D). Kaplan-Meier overall survival analysis with log-rank testing using TCGA data from cSurvival (https://tau.cmmt.ubc.ca/cSurvival/) platform indicated that high *SLC2A5* expression was not significantly associated with overall survival in ccRCC patients (*P* = 0.16, Fig. 1E). In contrast, low expression of KHK and ALDOB was significantly associated with poorer patient outcomes (Fig. 1F, G), whereas TKFC expression showed no significant association with overall survival (Fig. 1H). Moreover, a low-expression signature of the four-gene fructose-metabolism set (*SLC2A5, KHK, ALDOB, TKFC*) predicted worse survival (median split, log-rank *P* = 0.006, Fig. S1A). Consistent with the transcriptomic data, Western blot analysis of 24 paired ccRCC specimens revealed that GLUT5 was upregulated in tumor tissue, whereas KHK, ALDOB, and TKFC were downregulated relative to adjacent normal tissue (Fig. 1I–J and Fig. S1B). These findings suggest that despite upregulation of GLUT5 for fructose uptake, the intracellular fructose metabolic pathway is impaired in ccRCC, and this impairment is associated with adverse prognosis.

### Fructose metabolism exerts glucose-dependent effects on ccRCC cells

**Fig. 2:**
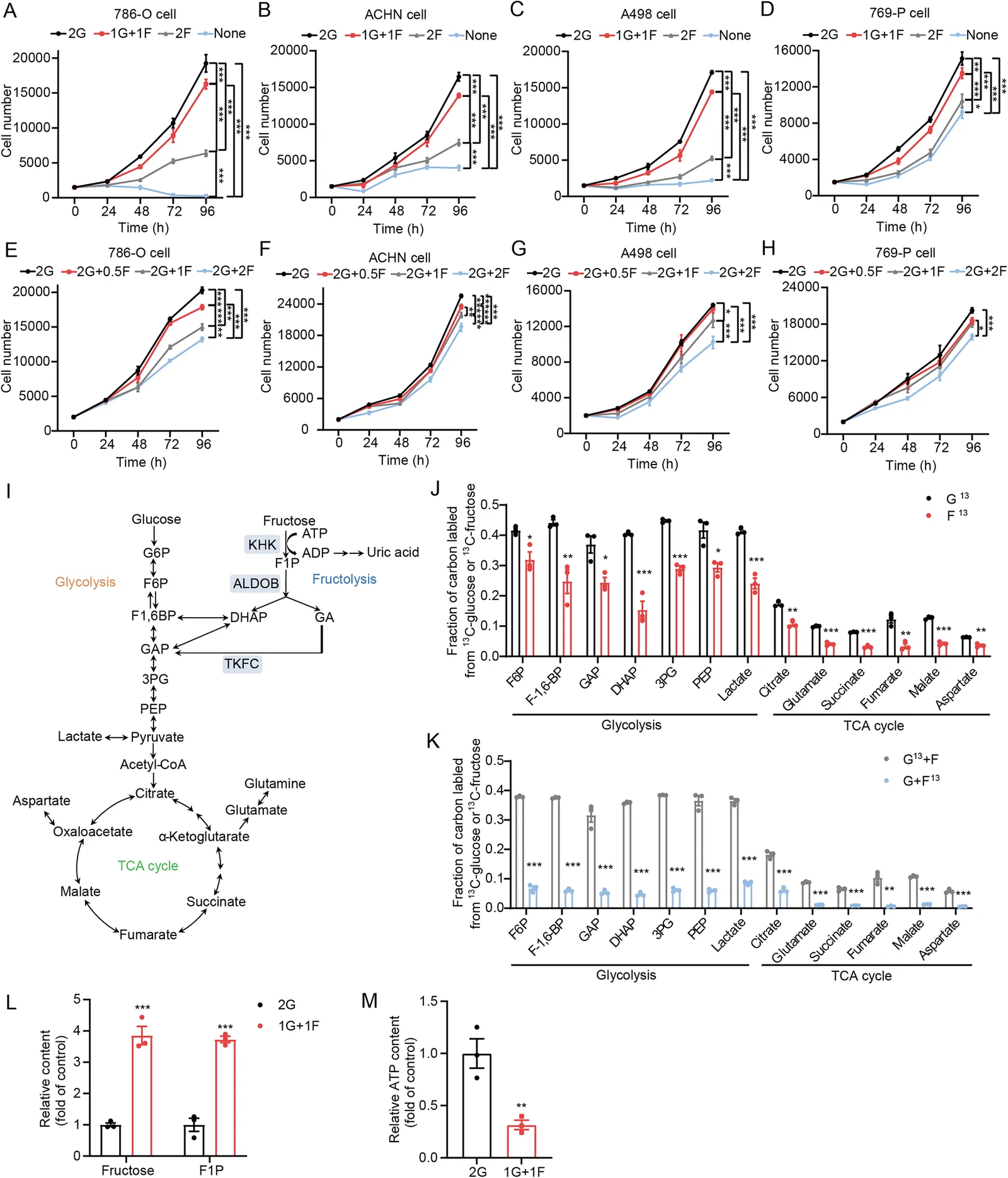
Fructose metabolism exerts glucose-dependent effects on ccRCC cells. **A–D** Viability of 786-O (**A**), ACHN (**B**), A498 (**C**), and 769-P (**D**) cells cultured in media with 2 g/L glucose (2 G), 1 g/L glucose plus 1 g/L fructose (1 G + 1 F), 2 g/L fructose (2 F), or no glucose and fructose (None) for the indicated times. **E–H** Viability of 786-O (**E**), ACHN (**F**), A498 (**G**), and 769-P (**H**) cells cultured in media containing 2 g/L glucose (2 G) supplemented with 0.5 g/L fructose (2 G + 0.5 F), 1 g/L fructose (2 G + 1 F), or 2 g/L fructose (2 G + 2 F) for the indicated times. **I** Schematic diagram of glucose and fructose metabolic pathways. KHK, ketohexokinase; ALDOB, aldolase B; TKFC, triokinase; F1P, fructose-1-phosphate; G6P, glucose-6-phosphate; F6P, fructose-6-phosphate; F1,6BP, fructose-1,6-bisphosphate; GA, glyceraldehyde; DHAP, dihydroxyacetone phosphate; GAP, glyceraldehyde 3-phosphate; 3PG, 3-phosphoglycerate; PEP, phosphoenolpyruvate. Fraction of labeled metabolites from glycolysis and the TCA cycle in 786-O cells, derived from U-^13^C-glucose or U-^13^C-fructose (uniformly labeled at all six carbons). Cells were treated for 24 h with: **J** 1 g/L U-^13^C-glucose plus 1 g/L unlabeled glucose (G^13^), or 1 g/L U-^13^C-fructose plus 1 g/L unlabeled fructose (F^13^); **K** 1 g/L U-^13^C-glucose plus 1 g/L unlabeled glucose and 2 g/L unlabeled fructose (G^13^ + F), or 1 g/L U-^13^C-fructose plus 1 g/L unlabeled fructose and 2 g/L unlabeled glucose (G + F^13^), *n* = 3. **L** Relative content of fructose and F1P in 786-O cells treated with 2 G or 1 G + 1 F media for 48 h, normalized to the 2 G group (*n* = 3). **M** Relative ATP content in 786-O cells treated with 2 G or 1 G + 1 F media for 48 h, normalized to the 2 G group (*n* = 3). All data are presented as mean ± SEM. *P* values were determined by two-way ANOVA with Tukey’s multiple comparisons test (**A–H**), multiple unpaired *t* tests with FDR correction (**J–L**) and two-tailed unpaired *t* test (**M**). *P < 0.05; **P < 0.01; ***P < 0.001.

To examine the biological consequences of fructose metabolism in ccRCC cells, we cultured multiple ccRCC cell lines (786-O, ACHN, A-498, 769-P) in media containing varying glucose and fructose concentrations. We found that, under a constant total sugar concentration, increasing fructose concentration led to a significant, dose-dependent decrease in cell proliferation. Notably, even the highest fructose conditions still exhibited higher proliferation than the no-sugar control, indicating that fructose alone sustains baseline proliferation (Fig. 2A–D). Consistent with previous reports [22, 23], under no-glucose or low-glucose conditions, fructose promotes cell proliferation in a dose-dependent manner, serving as an alternative energy source when glucose was limited (Fig. S2A and B). In marked contrast, under normal glucose conditions, elevated fructose concentrations did not exert an additive proliferative effect; instead, cell proliferation was markedly attenuated (Fig. 2E–H). These results suggest that, in the presence of glucose, fructose plays a metabolic role distinct from its pro-survival function under glucose-limited conditions.

Although glucose and fructose enter metabolism via distinct reactions, their carbon fluxes converge at GAP and thereafter share identical downstream routes through glycolysis and the TCA cycle (Fig. 2I). To quantify the differential utilization of the two sugars in different conditions, we performed stable-isotope tracing with U-^13^C-glucose or U-^13^C-fructose (each uniformly labeled at all six carbons). In single-substrate medium, although the ^13^C-label incorporation into glycolytic and TCA cycle metabolites from U-^13^C-fructose was markedly lower than that from U-^13^C-glucose in 786-O and ACHN cells, fructose metabolism remained robust, indicating that ccRCC cells can readily metabolize and utilize fructose (Fig. 2J and Fig. S2C). Importantly, in mixed substrate conditions, ^13^C-fructose labeling was significantly lower than ^13^C-glucose labeling and the coexistence of unlabeled glucose reduced the ^13^C contribution from U-^13^C-fructose compared to the single-fructose medium (Fig. 2K and Fig. S2D), confirming that ccRCC cells preferentially utilize glucose over fructose.

Given that ccRCC cells in vivo are not exposed to fructose as a sole carbon source, all subsequent experiments were performed in the presence of glucose to better recapitulate the physiological context. Indeed, under combined glucose and fructose treatment, fructose metabolism caused marked accumulation of F1P and intracellular fructose, along with substantial ATP depletion, consistent with previous reports [25] (Fig. 2L, M). We also observed significant accumulation of sorbitol, a key polyol pathway metabolite (Fig. S2E and F). Notably, unlike other metabolites, sorbitol labeling from U-^13^C-fructose was much lower than that from U-^13^C-glucose under single-substrate conditions, whereas fructose-derived sorbitol labeling increased markedly in the presence of glucose—an observation we will address in the Discussion (Fig. S2G). Together, these results indicate that in the presence of glucose, fructose metabolism is significantly reduced and its pro-survival effect is weakened. Furthermore, the profound ATP depletion suggests that fructose metabolism may induce metabolic disturbance under these conditions.

### Fructose attenuates oxidative phosphorylation and impairs complex I subunit expression in ccRCC cells in the presence of glucose

**Fig. 3:**
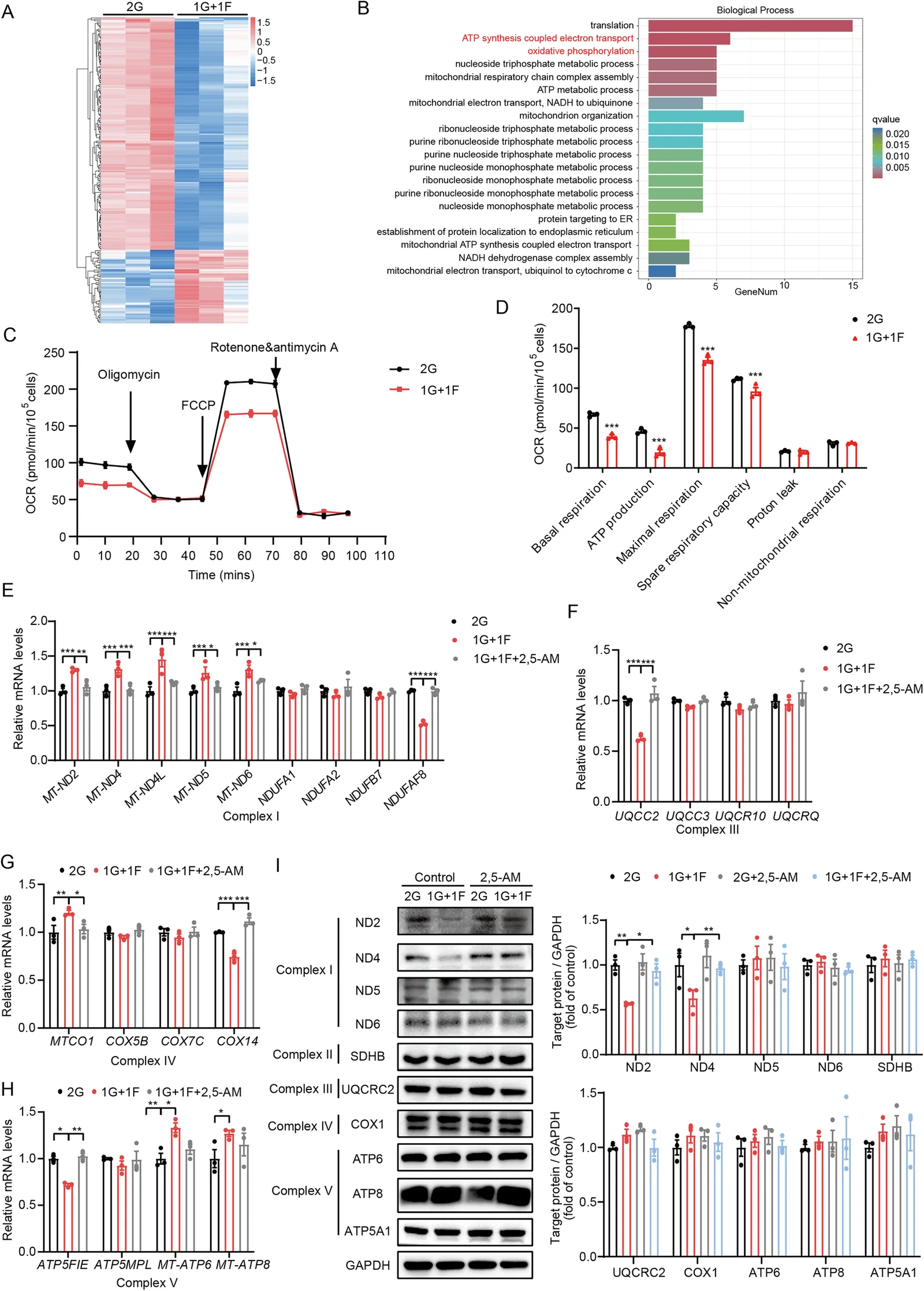
Fructose attenuates oxidative phosphorylation and impairs complex I subunit expression in ccRCC cells in the presence of glucose. **A** Heatmap of differentially expressed genes (DEGs) from RNA-Seq data in 786-O cells treated with 2 g/L glucose (2 G) or 1 g/L glucose plus 1 g/L fructose (1 G + 1 F) for 24 h (*n* = 3). Color scale: normalized expression (red: upregulation; blue: downregulation). **B** Gene Ontology (GO) enrichment analysis (Biological Process) of DEGs (1 G + 1 F vs 2 G). **C** Real-time oxygen consumption rate (OCR) of 786-O cells (pretreated with the indicated media for 24 h) following sequential treatment with oligomycin (1 μM), FCCP (1 μM), and rotenone/antimycin A (1 μM). OCR values were normalized to cell number (1 × 10⁵ cells per well) (*n* = 3). **D** Quantification of mitochondrial respiratory parameters derived from the OCR profiles in (**C**). **E–H** Relative mRNA expression levels of Electron Transport Chain (ETC) Complex I (**E**), III (**F**), IV (**G**), V (**H**) in 786-O cells treated with the indicated media and 2 mM 2,5-dehydro-D-mannitol (2,5-AM) for 24 h (normalized to the 2 G group) (*n* = 3). **I** Left panel: The protein expression of mtDNA-encoded subunits (ND2, ND4, ND5, ND6, COX1, ATP6, ATP8) and nuclear-encoded subunits (SDHB, UQCRC2, ATP5A1) of ETC Complex in 786-O cells treated with the indicated media and 2,5-AM for 48 h. Right panels: Quantitative analysis of the protein expression normalized to GAPDH and expressed as fold change relative to the 2 G control group (*n* = 3). All data are presented as mean ± SEM. *P* values were determined by multiple unpaired t-tests with FDR correction (**D**) and one-way ANOVA with Tukey’s multiple comparisons test (**E–I**). *P < 0.05; **P < 0.01; ***P < 0.001.

To further investigate the effects of fructose metabolism on ccRCC cells in the presence of glucose, we performed transcriptomic RNA sequencing (RNA-Seq) analysis on 786-O cells treated with 2 g/L glucose (2 G) or 1 g/L glucose plus 1 g/L fructose (1 G + 1 F) for 24 h. A total of 221 differentially expressed genes (DEGs) were identified, among which 168 genes were significantly downregulated and 53 genes were significantly upregulated in the 1 G + 1 F group compared to 2 G group (Fig. 3A). Gene Ontology (GO) enrichment analysis of Biological Process of the DEGs revealed significant alterations of translation as well as ATP synthesis coupled electron transport and oxidative phosphorylation (OXPHOS) (Fig. 3B). Cellular Component analysis indicated significant changes in mitochondria (Fig. S3A), while Molecular Function analysis showed notable alterations in mitochondrial electron transport chain (ETC) complex subunits (Fig. S3B). Collectively, these omics signatures suggested that fructose co-treatment preferentially dampens mitochondrial energy metabolism.

Given the strong bioinformatic indication of altered OXPHOS, we next sought to functionally validate this prediction by directly measuring mitochondrial respiratory activity. We measured the oxygen consumption rate (OCR) in 786-O cells and found that fructose addition significantly reduced OCR levels (Fig. 3C). This indicated a significant compromise in mitochondrial respiratory function, as evidenced by reductions in basal respiration, ATP production, maximal respiration, and spare respiratory capacity (Fig. 3D).

We then analyzed the DEGs from RNA-seq data and identified 21 significantly altered genes encoding electron-transport-chain subunits, predominantly within complexes I, III, IV, and V. Next, we validated these genes individually by RT-qPCR in 786-O cells and found that the mRNA levels of mitochondrial-DNA (mtDNA)-encoded ETC complex subunits—MT-ND2/4/4 L/5/6 (Complex I), MT-CO1 (Complex IV), and MT-ATP6/8 (Complex V)—were significantly upregulated, while nuclear-encoded subunits—NDUFAF8, UQCC2, COX14—were suppressed in 1 G + 1 F group compared to 2 G group (Fig. 3E–H), consistent with the RNA-seq data (Supplementary Table 5). Furthermore, treatment with 2,5-dehydro-D-mannitol (2,5-AM), an inhibitor of the fructose transporter GLUT5, significantly restored F1P level (Fig. S3C) and the expression changes of these genes (Fig. 3E–H), indicating that the change of mitochondrial genes was specifically caused by fructose rather than by glucose deprivation. Notably, we assessed key ETC complex protein levels by Western blot. We found that ND2 and ND4 protein levels were significantly reduced following fructose treatment, whereas 2,5-AM restored their expression (Fig. 3I and Fig. S3D). ND2 and ND4 are mtDNA-encoded core subunits of mitochondrial respiratory chain complex I (NADH: ubiquinone oxidoreductase or NADH dehydrogenase), which is essential for electron transfer, proton pumping, and ATP synthesis. The marked reduction of ND2 and ND4 protein levels following fructose treatment therefore suggests impaired complex I integrity or function.

The discordance between elevated mtDNA-encoded transcripts and decreased ND2/ND4 protein levels likely reflects a mitochondrial translation bottleneck. Indeed, Gene set enrichment analysis (GSEA) revealed profound repression of the translation gene signature (NES = −2.823, *P* = 0.001433) (Fig. S3E), indicating global suppression of translational machinery upon fructose treatment. This translational inhibition provides a mechanistic basis for the observed mRNA-protein discordance, suggesting that the transcriptional upregulation of mtDNA genes represents a futile compensatory response.

### Fructose impairs mitochondrial function and sensitizes ccRCC cells to nutrient stress via intrinsic apoptosis in the presence of glucose

**Fig. 4:**
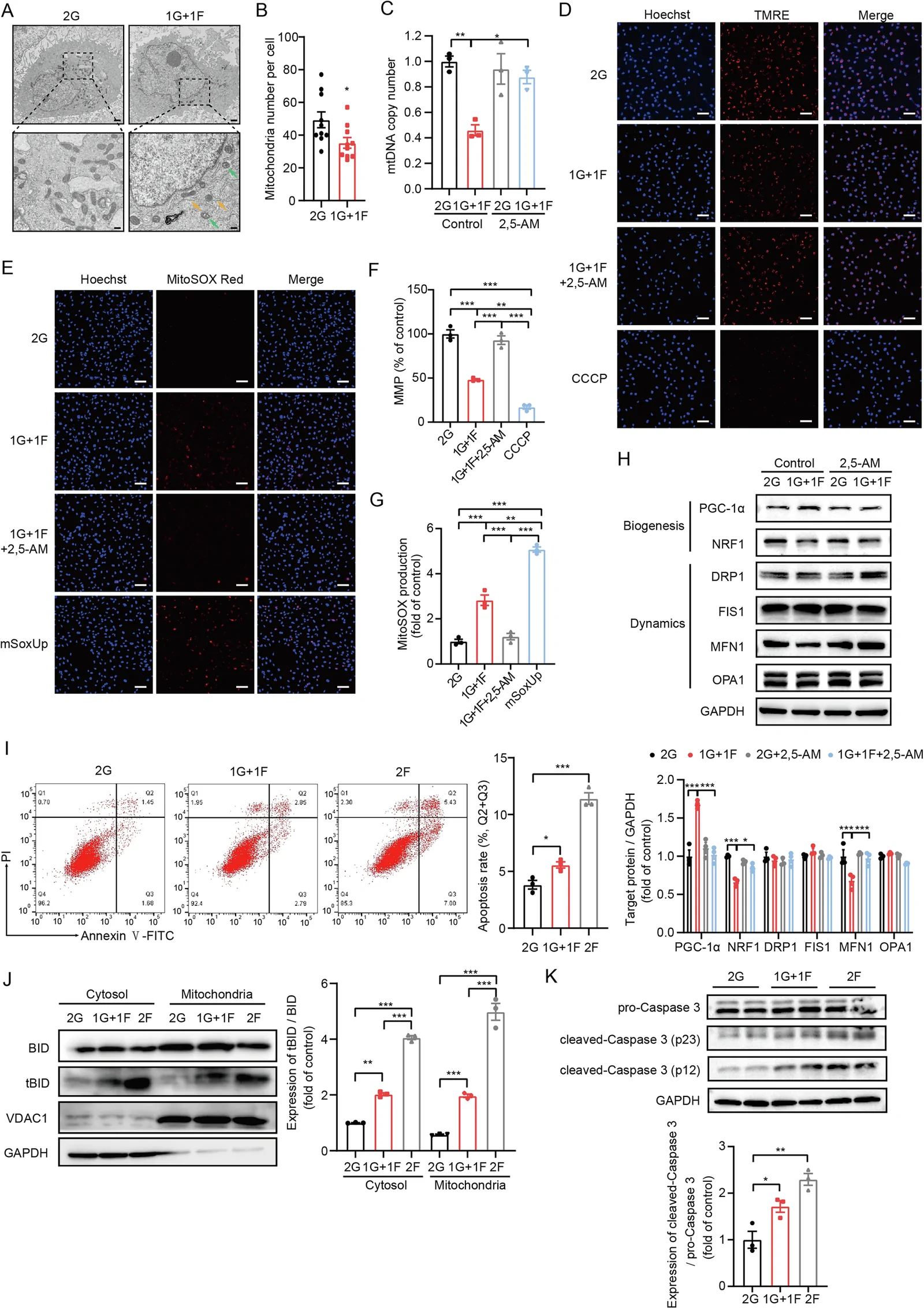
Fructose impairs mitochondrial function and sensitizes ccRCC cells to nutrient stress via intrinsic apoptosis in the presence of glucose. **A** Representative transmission electron microscopy (TEM) images of 786-O cells in 2 G or 1 G + 1 F group for 48 h (*n* = 3). Lower panel show magnified views of the boxed regions. Scale bars: upper panel, 1 μm; lower panel, 0.2 μm. Green arrows denote damaged mitochondria; orange arrows denote dilated endoplasmic reticulum. **B** Quantification of mitochondria number per cell from TEM images (10 random fields analyzed per group). **C** Relative mitochondrial DNA (mtDNA) copy number in 2 G and 1 G + 1 F groups treated with or without 2,5-AM (2 mM) in 786-O cells for 24 h (normalized to the 2 G control group, *n* = 3). **D** Representative fluorescence images of mitochondrial membrane potential (MMP) stained with TMRE in treated 786-O cells for 48 h. Positive controls: cells were pre-treated with CCCP for 1 h prior to staining. Scale bars: 100 μm. **E** Representative fluorescence images of mitochondrial superoxide stained with MitoSOX Red in treated 786-O cells for 48 h. Positive controls: cells were pre-treated with the superoxide inducer mSoxUp for 4 h prior to staining. Scale bars: 100 μm. **F–G** Quantitative analysis of TMRE (**F**) and MitoSOX Red (**G**) fluorescence intensity from (**D**) and (**E**) (normalized to the 2 G control group, *n* = 3), respectively. **H** Upper panel: The protein expression of mitochondrial biogenesis (PGC-1α, NRF1) and dynamics (DRP1, FIS1, MFN1, OPA1) regulators in 786-O cells treated with 2 G, 1 G + 1 F, or in combination with 2,5-AM (2 mM) for 48 h. Lower panel: Quantitative analysis of the protein expression normalized to GAPDH and expressed as fold change relative to the 2 G control group (*n* = 3). **I** Left panel: Apoptosis analysis by Annexin V-FITC/PI dual staining flow cytometry. 786-O cells were treated with indicated media for 48 h. Right panel: Quantification of total apoptosis rate (Q2 + Q3). Q2 (Annexin V⁺/PI⁺) and Q3 (Annexin V⁺/PI⁻) were defined as late and early apoptotic populations, respectively (*n* = 3). **J** Left panel: The protein expression of BID (full-length) and tBID (truncated, pro-apoptotic) in cytosolic/mitochondrial fractions in treated 786-O cells for 48 h (VDAC1: mitochondrial loading control; GAPDH: cytosolic loading control). Right panel: Quantitative analysis of tBID / BID and expressed as fold change relative to the cytosol of 2 G group (*n* = 3). **K** Upper panel: The protein expression of pro-Caspase 3 and its cleaved active forms (cleaved-Caspase 3, p23 and p12) in 786-O cells treated with the indicated media for 48 h under serum-free conditions. Lower panel: Quantitative analysis of cleaved-Caspase 3 (p23 and p12) / pro-Caspase 3 and expressed as fold change relative to the 2 G control group (*n* = 3). *P* values were determined by two-tailed unpaired *t* test (**B**) and one-way ANOVA with Tukey’s multiple comparisons test (**C**, **F–K**). All data are presented as mean ± SEM. *P < 0.05; **P < 0.01; ***P < 0.001.

In order to investigate the effect of fructose on mitochondria in ccRCC cells, we performed transmission electron microscopy (TEM) analysis in 786-O cells. As shown in Fig. 4A, mitochondria in the 2 G group displayed clear morphology and intact architecture, characterized by typical double membranes and well-defined cristae with a regular distribution. In stark contrast, mitochondria in the 1 G + 1 F group displayed severe aberrations, including swollen morphology, disintegrated and diminished cristae, and concurrent dilation of the adjacent ER. Quantitative analysis confirmed a significant reduction in mitochondrial number per cell following fructose co-treatment (Fig. 4B). The mitochondrial DNA (mtDNA) copy number, a key indicator of mitochondrial mass, was significantly depleted in the 1 G + 1 F group, whereas co-treatment with 2,5-AM reversed this reduction in 786-O cells (Fig. 4C) and ACHN cells (Fig. S4A).

Given the observed structural abnormalities, we next investigated whether mitochondrial function was also compromised. We therefore measured the mitochondrial membrane potential (MMP, ΔΨm) and reactive oxygen species (ROS) levels in ccRCC cells. Indeed, we observed a profound loss of ΔΨm in fructose-treated cells using TMRE staining (Fig. 4D, F and Fig. S4B, D). This disruption of the electron transport chain led to electron leakage and directly drove excessive mitochondrial superoxide production [26], as quantified by MitoSOX Red fluorescence (Fig. 4E, G and Fig. S4C, E). Notably, co-treatment with 2,5-AM largely restored ΔΨm and suppressed ROS elevation, suggesting that these functional defects are specifically driven by fructose uptake via GLUT5.

To investigate the molecular basis underlying mitochondrial dysfunction, we assessed the protein expression of key mitochondrial regulators by Western blot. As shown in Fig. 4H and Fig. S4F, fructose treatment significantly upregulated the mitochondrial biogenesis protein PGC-1α but downregulated NRF1. Among the mitochondrial dynamics regulators, the fusion-related protein MFN1 was markedly reduced, whereas the expression of OPA1 and the fission-related proteins DRP1 and FIS1 remained unchanged. Notably, co-treatment with 2,5-AM reversed these alterations. These results suggest that fructose selectively disrupts mitochondrial biogenesis and the fusion machinery, which may contribute to the observed mtDNA depletion and mitochondrial network disruption.

The profound mitochondrial dysfunction state, a well-established trigger of the intrinsic apoptotic pathway—particularly via the activation of the BID-tBID axis—invariably led to cell death [27]. Indeed, fructose significantly increased the percentage of early (Annexin V^+^/PI^-^) and late (Annexin V^+^/PI^+^) apoptotic 786-O cells, and this increase was more pronounced under fructose-only culture conditions (Fig. 4I). We also observed the proteolytic cleavage of BID to its active, pro-apoptotic form tBID in the mitochondria (Fig. 4J). Moreover, under serum-free conditions—which minimize interference from serum-derived survival factors—we observed activation of executioner caspase-3 in 786-O and ACHN cells (Fig. 4K and Fig. S4G). Collectively, these data demonstrate that fructose induces mitochondrial dysfunction and sensitizes ccRCC cells to nutrient stress via intrinsic apoptosis in the presence of glucose.

### PERK-dependent ER stress mediates fructose-induced mitochondrial dysfunction in ccRCC cells

**Fig. 5:**
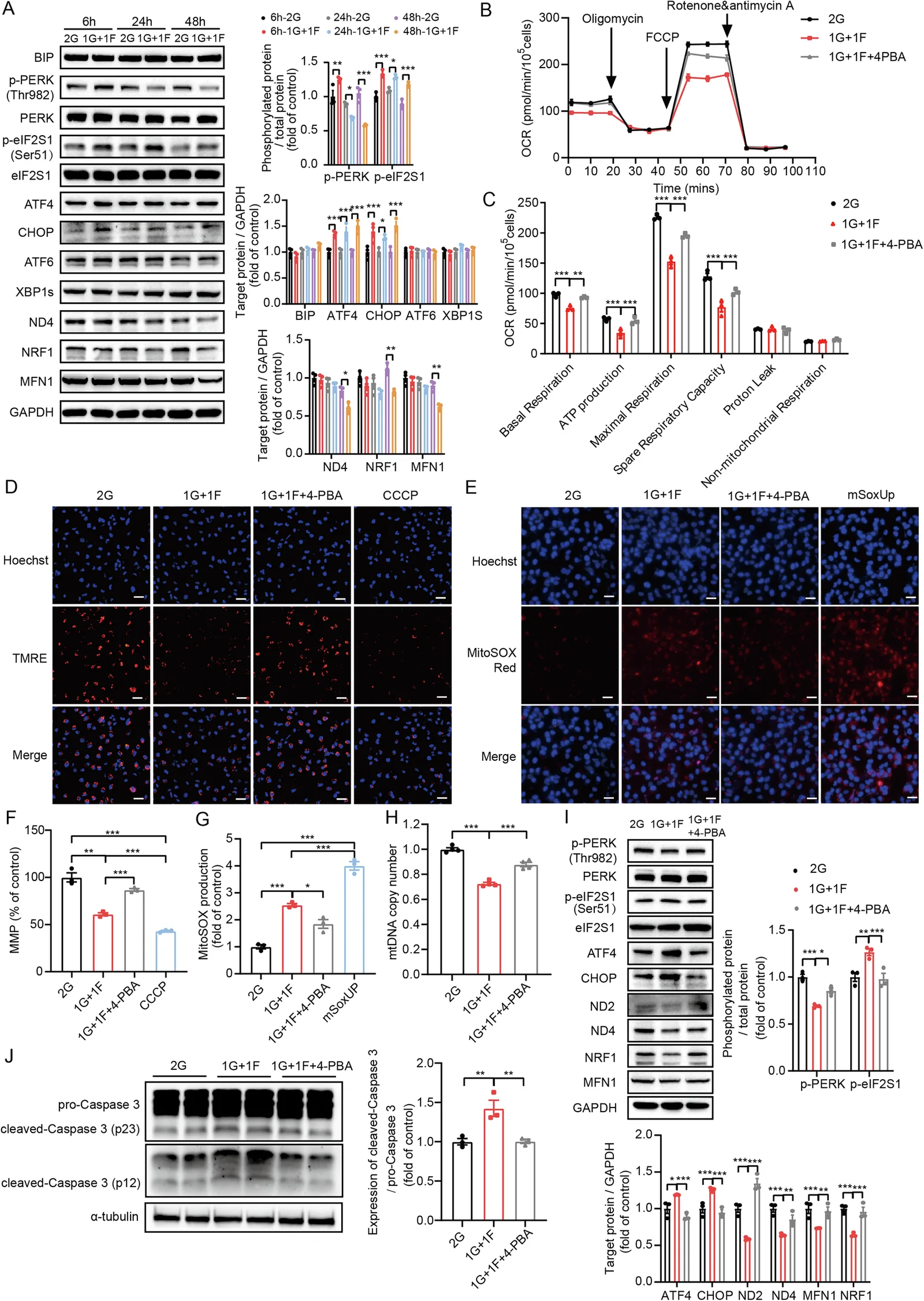
PERK-dependent ER stress mediates fructose-induced mitochondrial dysfunction in ccRCC cells. **A** Left panel: The protein expression of ER stress markers (BIP, p-PERK, PERK, p-eIF2S1, eIF2S1, ATF4, CHOP, ATF6, XBP1s) and mitochondrial proteins (ND4, NRF1, MFN1) in 786-O cells treated with the indicated media for 6 h, 24 h, and 48 h. Right panels: Quantitative analysis of the protein expression normalized to GAPDH and expressed as fold change relative to the 2 G control group (*n* = 3). **B** OCR of 786-O cells treated with the indicated media for 24 h. 4-PBA: ER stress inhibitor. 0.5 mM 4-PBA was added in the media for 24 h in 1 G + 1 F + 4-PBA group. OCR values were normalized to cell number (1 × 10⁵ cells per well). **C** Quantification of mitochondrial respiratory parameters derived from the OCR profiles in (**B**) (*n* = 3). **D–E** Representative fluorescence images of MMP stained with TMRE (**D**) and mitochondrial superoxide stained with MitoSOX Red (**E**) in treated 786-O cells for 24 h. Scale bars: 50 μm (**D**). Scale bars: 25 μm (**E**). **F–G** Quantitative analysis of TMRE (**F**) and MitoSOX Red (**G**) fluorescence intensity from (**D**) and (**E**) (normalized to the 2 G control group, *n* = 3), respectively. **H** Relative mtDNA copy number in treated 786-O cells for 24 h (normalized to the 2 G control group, *n* = 3). **I** Left panel: The protein expression of ER stress markers (p-PERK, PERK, p-eIF2S1, eIF2S1, ATF4, CHOP) and mitochondrial proteins (ND2, ND4, NRF1, MFN1) in 786-O cells treated with the indicated media for 48 h. Right panel and lower panel: Quantitative analysis of the protein expression normalized to GAPDH and expressed as fold change relative to the 2 G control group (*n* = 3). **J** Left panel: The protein expression of pro-Caspase 3 and its cleaved active forms (cleaved-Caspase 3, p23, and p12) in 786-O cells treated with the indicated media for 48 h under serum-free conditions. Right panel: Quantitative analysis of cleaved-Caspase 3 (p23 and p12) / pro-Caspase 3 and expressed as fold change relative to the 2 G control group (*n* = 3). All data are presented as mean ± SEM. *P* values were determined by one-way ANOVA with Tukey’s multiple comparisons test (**A**, **C**, **F–J**). *P < 0.05; **P < 0.01; ***P < 0.001.

We next explored the upstream mechanism linking fructose to mitochondrial damage. Our initial TEM analysis revealed a notable dilation of the ER in fructose-treated cells (Fig. 4A). In addition, GO enrichment analysis of the DEGs highlighted significant alterations in biological processes related to protein targeting to ER and establishment of protein localization to endoplasmic reticulum (Fig. 3B). These findings prompted us to hypothesize that fructose triggers ER stress. Indeed, fructose exposure selectively engaged the PERK-eIF2S1-ATF4 axis, as evidenced by phosphorylation of PERK and EIF2S1, induction of ATF4, and upregulation of CHOP (Fig. 5A and Fig. S5A). Notably, this occurred without activation of canonical unfolded protein response (UPR) markers (BIP, XBP1s, ATF6), indicating that fructose triggers the integrated stress response (ISR) through metabolic or oxidative stress. The temporal sequence—phosphorylation of PERK and EIF2S1 evident as early as 6 h, preceding the downregulation of ND4, MFN1, and NRF1 expression at 48 h—supports that the ISR acts upstream of mitochondrial dysfunction. Notably, the decreased PERK phosphorylation at 24 h and 48 h may reflect a feedback regulatory mechanism or adaptive attenuation of the stress signal.

Then we employed the chemical chaperone 4-phenylbutyric acid (4-PBA), which alleviates ER protein folding burden and restores ER homeostasis, to test whether modulating ER integrity could rescue fructose-induced phenotypes. Notably, 4-PBA treatment significantly restored mitochondrial respiratory function (Fig. 5B, C), the loss of ΔΨm (Fig. 5D, F and Fig. S5B), the accumulation of mitochondrial superoxide (Fig. 5E, G and Fig. S5C), as well as mtDNA copy number (Fig. 5H and Fig. S5D) in fructose-treated cells (1 G + 1 F + 4-PBA group). Furthermore, the protein expression of the PERK-EIF2S1-ATF4-CHOP axis, mitochondria-related proteins and the activation of caspase-3 was significantly restored upon 4-PBA treatment (Fig. 5I, J and Fig. S5E, F). To specifically investigate whether PERK-dependent ER stress mediates fructose-induced mitochondrial dysfunction, we knocked down CHOP using siRNA, which recapitulated the rescue effects observed with 4-PBA treatment. CHOP knockdown significantly restored ΔΨm, attenuated mitochondrial superoxide production, increased mtDNA copy number, and recovered the protein levels of key mitochondrial regulators ND4, NRF1, and MFN1 (Fig. S6A–F). These rescue data collectively indicate that PERK-dependent ER stress serves as the critical mediating event driving fructose-induced mitochondrial dysfunction.

### Nucleotide pool depletion mediates fructose-induced PERK-dependent ER stress and mitochondrial dysfunction in ccRCC cells

**Fig. 6:**
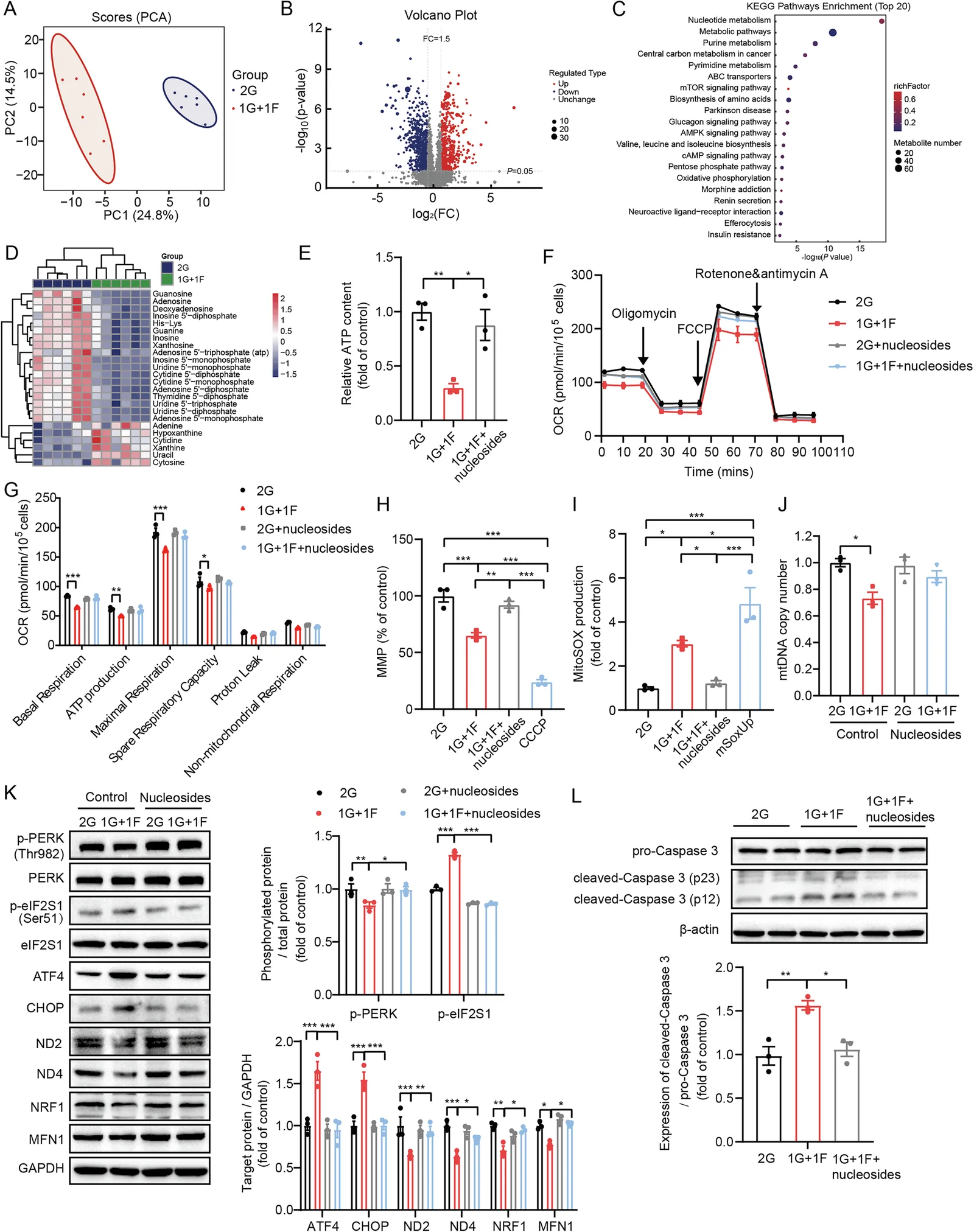
Nucleotide pool depletion mediates fructose-induced PERK-dependent ER stress and mitochondrial dysfunction in ccRCC cells. **A** Principal component analysis (PCA) score plot of metabolic profiles in negative ion mode from 786-O cells treated with 2 G or 1 G + 1 F for 48 h, *n* = 6. The ellipses represent 95% confidence intervals, showing clear separation between the two groups. PC1 and PC2 explain 24.8% and 14.5% of the total variance, respectively. **B** Volcano plot of differentially expressed metabolites ( | log₂FC | > 0.5, *P* < 0.05) in negative ion mode between the 2 G and 1 G + 1 F groups. **C** Top 20 enriched KEGG pathways based on differential metabolites. The color gradient indicates the enrichment factor, and the point size represents the number of metabolites involved in each pathway. **D** Hierarchical clustering heatmap of significantly altered metabolites involved in nucleotide metabolism. The color scale represents relative metabolite abundance (red: upregulated, blue: downregulated) in the 1 G + 1 F group compared to the 2 G control group. **E** Relative ATP content in 786-O cells treated with 2 G, 1 G + 1 F, 2 G+nucleosides, or 1 G + 1 F+nucleosides (25 µM each of guanosine, adenosine, and uridine) for 48 h (normalized to the 2 G group). **F** OCR of 786-O cells treated with the indicated media for 24 h. OCR values were normalized to cell number (1 × 10⁵ cells per well). **G** Quantification of mitochondrial respiratory parameters derived from the OCR profiles in (**F**) (*n* = 3). **H–I** Quantitative analysis of TMRE (**H**) and MitoSOX Red (**I**) fluorescence intensity from Fig. S8A and Fig. S8B (normalized to the 2 G group, *n* = 3), respectively. **J** Relative mtDNA copy number in treated 786-O cells for 24 h (normalized to the 2 G control group, *n* = 3). **K** Left panel: The protein expression of ER stress markers (p-PERK, PERK, p-eIF2S1, eIF2S1, ATF4, CHOP) and mitochondrial proteins (ND2, ND4, NRF1, MFN1) in 786-O cells treated with the indicated media for 48 h. Right panel and lower panel: Quantitative analysis of the protein expression normalized to GAPDH and expressed as fold change relative to the 2 G control group (*n* = 3). **L** Upper panel: The protein expression of pro-Caspase 3 and its cleaved active forms (cleaved-Caspase 3, p23 and p12) in 786-O cells treated with the indicated media for 48 h under serum-free conditions. Lower panel: Quantitative analysis of cleaved-Caspase 3 (p23 and p12) / pro-Caspase 3 and expressed as fold change relative to the 2 G control group (*n* = 3). All data are presented as mean ± SEM. *P* values were determined by one-way ANOVA with Tukey’s multiple comparisons test (**E**, **G**–**K**). *P < 0.05; **P < 0.01; ***P < 0.001.

To explore the metabolic alterations underlying fructose-induced PERK-dependent ER stress, we conducted untargeted metabolomics analysis. Quality control (QC) samples exhibited high correlation coefficients (R² ≥0.994) (Fig. S7A), low distribution of relative standard deviation (RSD) values (<30%) (Fig. S7B), and highly consistent total ion chromatograms in both ionization modes (Fig. S7C and D), demonstrating robust analytical reproducibility for subsequent metabolomics analysis. Metabolomics analysis demonstrated a clear separation between 1 G + 1 F and 2 G groups in principal component analysis (PCA), indicating robust metabolic reprogramming upon fructose exposure (Fig. 6A and Fig. S7E). Volcano plot analysis identified 167 significantly differentially expressed metabolites ( | log₂FC | > 0.5, *P* < 0.05) (Fig. 6B and Fig. S7F). KEGG pathway enrichment analysis (Top 20) highlighted that the most significantly altered pathways included nucleotide metabolism, purine metabolism, central carbon metabolism, as well as pathways related to energy production (oxidative phosphorylation) and stress response (AMPK/mTOR signaling) (Fig. 6C). These data suggest that fructose co-treatment profoundly rewires cellular metabolism, particularly impacting nucleotide and energy metabolism pathways, which may contribute to the activation of PERK-dependent ER stress and mitochondrial dysfunction.

Consistently, the heatmap of nucleotide metabolism showed that most purine and pyrimidine nucleotides (e.g., ATP, IMP, UMP, and their phosphorylated derivatives) were significantly downregulated in the 1 G + 1 F group, whereas their catabolic intermediates (e.g., hypoxanthine, xanthine, and uracil) were markedly upregulated (Fig. 6D). These findings indicate that fructose co-treatment drives a profound rewiring of nucleotide metabolism, characterized by enhanced nucleotide catabolism and reduced levels of high-energy nucleotides, which may contribute to impaired energy homeostasis and exacerbated cellular stress.

Given these nucleotide pool alterations, we hypothesized that restoring nucleotide pools might alleviate fructose-induced cellular stress. To test this, we supplemented fructose-treated cells with exogenous guanosine, adenosine, and uridine—precursors that replenish purine (ATP/ADP/AMP and GTP/GDP/GMP) and pyrimidine (UTP/UDP/UMP) nucleotide pools, respectively. We found that nucleoside supplementation significantly rescued ATP levels (Fig. 6E) and mitochondrial respiratory function (Fig. 6F, G). Moreover, the fructose-induced alterations in ΔΨm, mitochondrial superoxide production, and mtDNA copy number were also restored (Fig. 6H–J and Fig. S8A–F). Additionally, nucleoside supplementation attenuated PERK-dependent ER stress, restored the expression of key mitochondrial proteins (Fig. 6K and Fig. S8G), and reduced caspase-3 activation under nutrient stress (Fig. 6L). Taken together, these findings indicate that fructose-induced nucleotide pool depletion and the resulting energy stress are critical upstream events linking metabolic rewiring to PERK-dependent ER stress and mitochondrial dysfunction, as exogenous nucleoside supplementation largely rescues these phenotypes.

### Fructose impairs ccRCC organoid viability and inhibits tumor growth in vivo

**Fig. 7:**
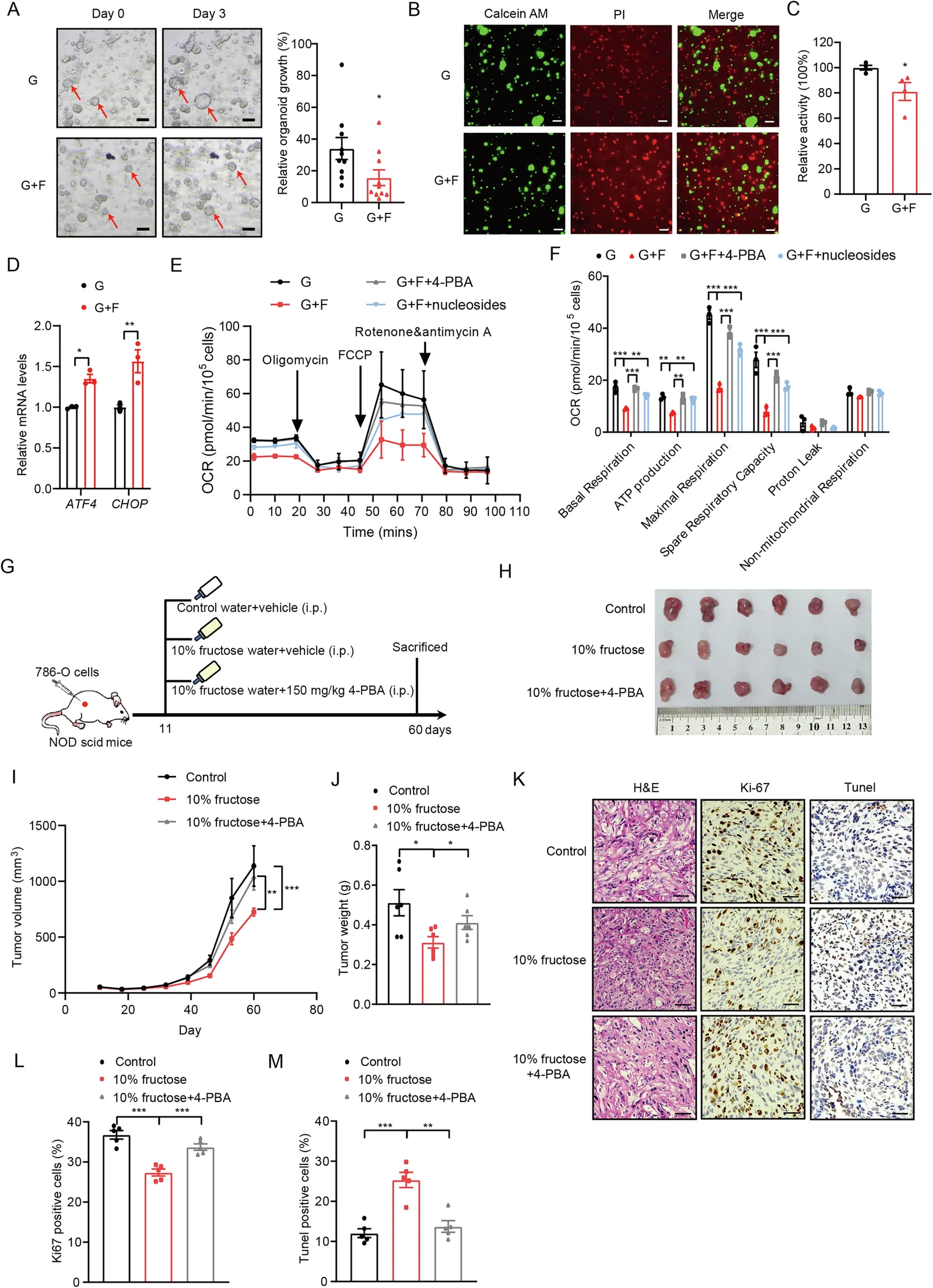
Fructose impairs ccRCC organoid viability and inhibits tumor growth in vivo. **A** Representative brightfield images (left) and quantification (right) of relative organoid growth in ccRCC organoid cultured in the indicated media at Day 0 and Day 3. G: 5 g/L glucose, G + F: 2.5 g/L glucose+2.5 g/L fructose. Relative organoid growth was calculated as the percentage increase in organoid diameter from day 0 to day 3: [(D₃ − D₀) / D₀] × 100%, normalized to the control group (set to 100%). Scale bar: 50 μm. **B** Viability assessment of organoids stained with Calcein AM (live cells, green) and PI (dead cells, red), *n* = 3. Scale bar: 50 μm. **C** Relative activity by measuring ATP content of treated organoids after 72 h of culture (normalized to the G group, *n* = 3). **D** Relative mRNA expression of *ATF4*, *CHOP* in treated organoids for 48 h (normalized to the G group, *n* = 3). **E** OCR of organoids treated with the indicated media for 48 h. OCR values were normalized to cell number (1 × 10⁵ cells per well). **F** Quantification of mitochondrial respiratory parameters derived from the OCR profiles in (**E**) (*n* = 3). **G** Schematic of the in vivo xenograft experiment. 786-O cells were subcutaneously injected into NOD scid mice. On day 11 post-inoculation, mice were randomized into three groups (*n* = 6 per group): 1) control drinking water + vehicle (0.9% NaCl, i.p., every other day); 2) 10% fructose water + vehicle; 3) 10% fructose water + 4-PBA (150 mg/kg, i.p., every other day). Treatments continued for 7 weeks. **H** Representative images of excised tumors from each group. **I** Tumor volume growth curve during the period of administration. Statistical analysis presented in the figure reflects comparisons at the terminal time point. **J** Final tumor weight in each group. **K** Histological and immunostaining analysis of tumors: H&E (morphology), Ki-67 (proliferation), TUNEL (apoptosis). Scale bar: 50 μm. **L–M** Quantification of Ki-67-positive cells (**L**) and TUNEL-positive cells (**M**) in tumor sections (5 random fields analyzed per group). All data are presented as mean ± SEM. *P* values were determined by two-tailed unpaired *t* test (**A**, **C**), multiple unpaired t-tests with FDR correction (**D**), two-way ANOVA with Tukey’s multiple comparisons test (**I**), and one-way ANOVA with Tukey’s multiple comparisons test (**F**, **J**, **L**, **M**). *P < 0.05; **P < 0.01; ***P < 0.001.

To further validate the physiological effect of fructose, we established patient-derived organoids (PDOs) from surgical specimens of ccRCC patients. These PDOs faithfully recapitulated the histopathological architecture and marker expression of the primary tumors, as confirmed by hematoxylin-eosin (H&E) and immunohistochemical staining (Fig. S9A). Upon fructose exposure, these organoids exhibited significantly slower growth in the glucose and fructose co-treatment (G + F) group than in the glucose (G) group by day 3 (Fig. 7A). Viability assessment using Calcein-AM (live) and propidium iodide (PI, dead) staining revealed a significant reduction in green fluorescence and a concomitant increase in red signal following fructose co-treatment (Fig. 7B). This decline in cell viability was further supported by a marked reduction in intracellular ATP content (Fig. 7C). RT-qPCR analysis revealed that fructose upregulated ER stress markers ATF4 and CHOP (Fig. 7D). Consistently, the OCR was markedly reduced in the G + F group compared to the G group. Importantly, treatment with either 4-PBA or nucleosides supplementation significantly rescued OCR levels in the G + F group (Fig. 7E, F), establishing that fructose-induced nucleotide pool depletion and subsequent ISR activation converge to drive mitochondrial dysfunction in PDOs.

We next evaluated the impact of dietary fructose uptake on tumor growth in a xenograft mouse model. Following subcutaneous inoculation of 786-O cells into NOD scid mice, animals were randomized into three groups on day 11 post-inoculation: vehicle control, 10% (w/v) fructose in drinking water, and fructose water plus the ER stress inhibitor 4-PBA (150 mg/kg, i.p., every other day) for seven weeks (Fig. 7G). Chronic fructose administration significantly attenuated tumor growth, resulting in reduced final tumor volume and tumor weight without affecting body weights (Fig. S9B), which were substantially reversed by 4-PBA co-treatment (Fig. 7H–J). Immunohistochemical analysis of harvested tumors revealed that fructose feeding suppressed proliferation (lower Ki-67-positive cells; Fig. 7K, L) and promoted apoptosis (higher TUNEL-positive cells; Fig. 7K, M), both of which were mitigated by 4-PBA administration (Fig. 7K–M). Collectively, these complementary models demonstrate that fructose impairs ccRCC organoid viability and inhibits tumor growth in vivo via ER stress-dependent mechanism.

## Discussion

The metabolic landscape of ccRCC features profound nutrient reprogramming, yet the role of fructose remains paradoxically underexplored. Our study reveals a paradoxical metabolic vulnerability: ccRCC tumors overexpress GLUT5 while downregulating the downstream catabolic enzymes—KHK, ALDOB, and TKFC. This expression pattern, validated across TCGA cohorts and patient specimens, predicts poor prognosis (Fig. 1).

This paradox led us to systematically re-evaluate the functional consequence of fructose metabolism in ccRCC. Stable isotope tracing confirmed that ccRCC cells metabolize fructose efficiently under glucose-limited conditions (Fig. 2J, Fig. S2C), consistent with prior reports in AML [13] and breast cancer [28]. However, when glucose is present, fructose utilization is markedly impaired and elevated fructose suppresses tumor cell proliferation (Fig. 2E–H). Unlike other cancer types that upregulate fructose metabolic enzymes KHK [29] or ALDOB [30] to utilize fructose efficiently for proliferation and migration, ccRCC exhibited marked downregulation of KHK, ALDOB, and TKFC (Fig. 1), suggesting a limited capacity to channel fructose carbons into anabolic pathways. Consequently, fructose accumulates as F1P (Fig. 2L) and decreases ATP levels (Fig. 2M), leading to metabolic stress rather than serving as an alternative energy substrate.

While the Warburg effect supports tumor growth, functional mitochondria remain indispensable for oxidative phosphorylation [31], biosynthetic precursor supply [32], redox homeostasis [33], and apoptosis regulation [34]. ER and mitochondria communicate through mitochondria-associated membranes (MAMs), which facilitate the exchange of calcium, lipids, and regulatory signals [35]. When ER homeostasis is disrupted, it profoundly impacts mitochondrial function [36]. We showed that activated PERK-ATF4-CHOP signaling acts upstream of mitochondrial dysfunction to suppress mtDNA-encoded Complex I subunits ND2 and ND4, fusion protein MFN1, and biogenesis factor NRF1—effects reversed by 4-PBA and CHOP knockdown (Fig. 5 and Fig. S6). Notably, fructose upregulated PGC-1α without concurrent NRF1 induction, representing futile compensatory signaling. This mitochondrial compromise sensitized cells to intrinsic apoptosis via BID cleavage and caspase-3 activation under nutrient stress (Fig. 4J–K).

To understand the metabolic consequences of fructose-induced energy stress, we performed untargeted metabolomics analysis in ccRCC cells in the presence of glucose. This analysis revealed a profound rewiring of nucleotide metabolism, characterized by depletion of high-energy nucleotides and accumulation of catabolic products (Fig. 6A–D). This finding is consistent with previous researches that fructose phosphorylation by KHK is unregulated, driving massive F1P accumulation and ATP depletion [37]. The resulting energy crisis disrupts nucleotide homeostasis, as ATP depletion alters the AMP/ATP ratio and drives accelerated AMP degradation via AMP deaminase, ultimately depleting purine and pyrimidine pools [38–40]. Exogenous nucleoside supplementation restored ATP, mitochondrial respiration, membrane potential, and mtDNA copy number (Fig. 6E–J), while attenuating PERK signaling and caspase-3 activation (Fig. 6K–L). These results establish nucleotide depletion as the critical upstream event linking fructose exposure to ER stress and mitochondrial dysfunction.

Notably, while Feng et al. reported that glucose and fructose co-presence activates sorbitol dehydrogenase (SORD)-mediated fructose-to-sorbitol conversion to promote colorectal cancer metastasis [41], we observed significant sorbitol accumulation in fructose-treated ccRCC cells with glucose enhancing this flux (Fig. S2E–G). However, whether this conversion mediates the fructose-induced phenotype in our model remains to be determined. The direct reduction or indirect conversion via glucose intermediates remains ambiguous and would require SORD knockout validation. Moreover, the potential contribution of sorbitol-induced osmotic stress [42] or ER stress to the overall phenotype cannot be entirely excluded and warrants further investigation.

Translational validation in PDOs and xenograft models confirms the physiological relevance of this pathway. ccRCC organoids faithfully replicate the fructose sensitivity of established cell lines, with reduced viability, ATP depletion, and PERK-dependent ER stress activation (Fig. 7A–F). In vivo, chronic dietary fructose administration significantly suppresses tumor growth, an effect reversed by co-treatment with 4-PBA (Fig. 7G–M). These data demonstrate that the fructose-induced metabolic vulnerability is not an artifact of cell culture but an exploitable therapeutic target.

**Fig. 8:**
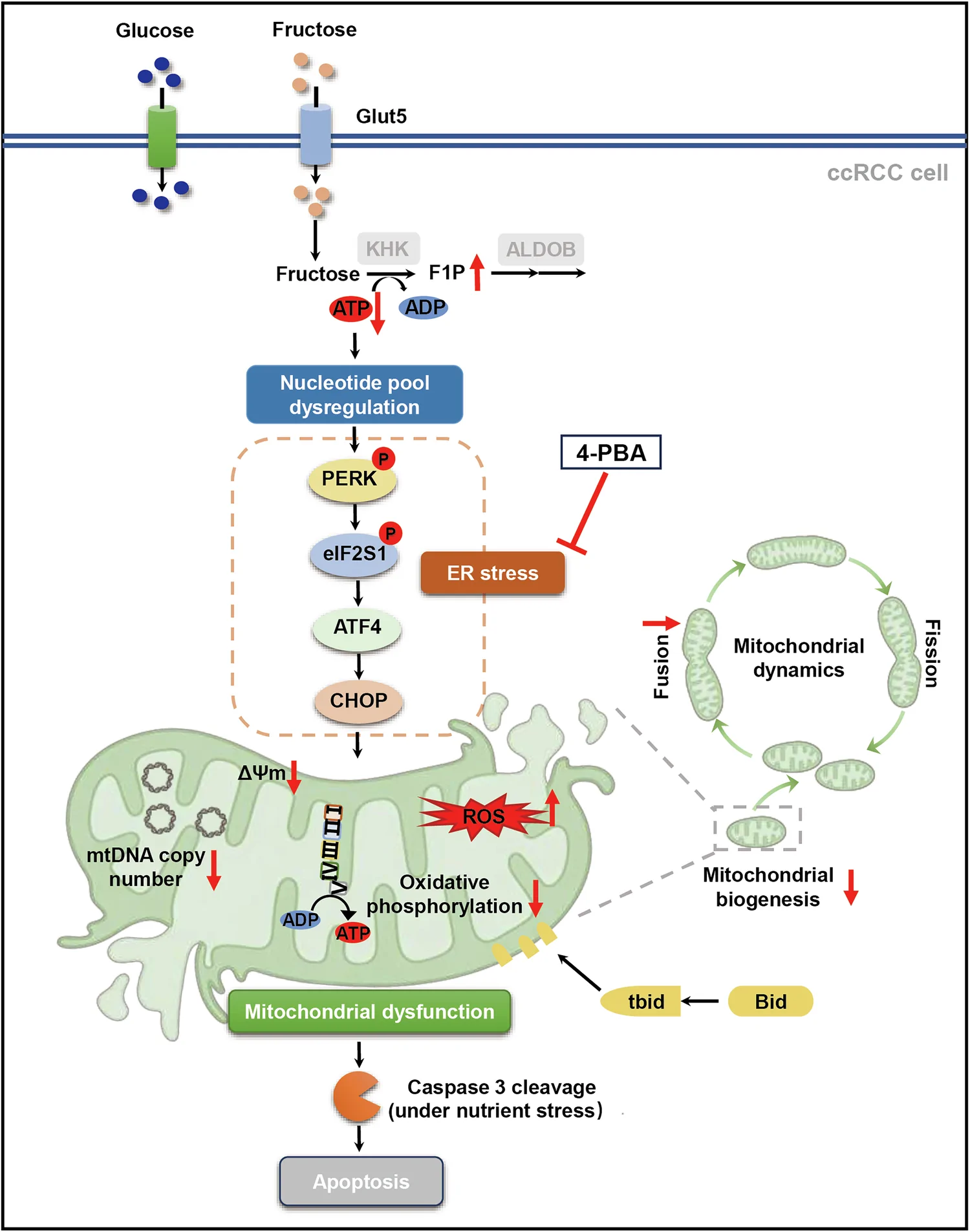
Proposed model for fructose induced PERK-dependent ER stress and mitochondrial dysfunction via nucleotide pool depletion in ccRCC. In ccRCC, fructose metabolism results in F1P accumulation and ATP depletion. This energy crisis drives nucleotide pool dysregulation, which selectively activates the PERK-eIF2S1-ATF4-CHOP axis of the integrated stress response (ISR). PERK-dependent ER stress impairs mitochondrial dynamics and biogenesis, and suppresses expression of mtDNA-encoded ETC subunits (notably ND2 and ND4 of Complex I), leading to mitochondrial dysfunction characterized by loss of membrane potential (ΔΨm), reduced mtDNA copy number, impaired oxidative phosphorylation, and increased ROS production. This mitochondrial compromise activates the intrinsic apoptosis via BID cleavage and caspase-3 activation under nutrient stress. Pharmacological intervention with the chemical chaperone 4-PBA alleviates ER stress and rescues the fructose-induced phenotype. This model reveals the tumor-suppressive toxicity of fructose in ccRCC and exposes a targetable metabolic vulnerability.

In summary, we propose a working model depicting the mechanism underlying glucose-dependent fructose toxicity in ccRCC (Fig. 8). In this model, fructose metabolism in the presence of glucose drives nucleotide depletion and induces PERK-dependent ER stress, ultimately compromising mitochondrial function and mediating the tumor-suppressive toxicity of fructose, which sensitizes cells to apoptosis upon nutrient stress. This model not only resolves the paradox of fructose metabolism in ccRCC but also highlights a novel, targetable metabolic vulnerability amenable to therapeutic exploitation.

However, several important questions remain to be addressed to fully elucidate the mechanistic and translational implications of fructose metabolism in ccRCC. First, how F1P accumulation and ATP depletion specifically disrupt nucleotide synthesis versus salvage pathways remains unresolved. Second, the precise molecular sensor linking nucleotide depletion to PERK-dependent ER stress activation—whether through ROS-mediated kinase oxidation, altered ER luminal redox state, or structural perturbation at mitochondria-ER contact sites—awaits identification. Third, the detailed signaling cascade from the stressed ER to the transcriptional and functional repression of mitochondrial OXPHOS requires elucidation. It will be crucial to determine whether this ER-to-mitochondria communication is primarily mediated by dysregulated Ca²⁺ homeostasis at MAMs, thereby impairing organelle function. Finally, from a translational perspective, our in vivo model does not fully recapitulate the intermittent dietary fructose absorption and systemic kinetics characteristic of human physiology, necessitating validation in more physiologically faithful models.

## Materials and methods

### Cell culture

786-O, ACHN, A498, and 769-P cells were purchased from Procell Life Science & Technology (Wuhan, China). The cell lines were authenticated by STR profiling and tested negative for mycoplasma contamination. ACHN and A498 cells were cultured in Dulbecco’s Modified Eagle Medium (DMEM; Gibco), while 786-O and 769-P cells were cultured in RPMI 1640 medium (Gibco). Both media were supplemented with 10% fetal bovine serum (FBS; Gibco) and 1% penicillin-streptomycin (Gibco). Cells were maintained at 37 °C in a humidified atmosphere containing 5% CO₂.

### Cell viability assay

Cells were seeded at a density of 2 × 10³ cells per well in 96-well plates. After adherence, cells were treated with the indicated media for different time points (*n* = 6 or 7). Cell viability was measured using the CCK-8 Cell Proliferation and Cytotoxicity Assay Kit (Beijing Solarbio Science & Technology, CA1210), following the manufacturer’s instructions. Absorbance at 450 nm was measured after incubating with the CCK-8 reagent for 1 h at 37 °C.

### Transmission electron microscop

786-O cells were treated with different medium containing 2 g/L glucose or 1 g/L glucose plus 1 g/L fructose for 48 h (*n* = 3). The cells were then fixed by 2.5% (v/v) glutaraldehyde at 4 °C overnight. Samples for TEM imaging were prepared and stained as described previously [43] and observed using the Hitachi HT-7800 transmission electron microscope (Japan).

### Mitochondria isolation

786-O cells were treated with the indicated medium containing 2 g/L glucose, 2 g/L fructose, or 1 g/L glucose plus 1 g/L fructose for 48 h, respectively (*n* = 3). Mitochondria were isolated using Cell Mitochondria Isolation Kit (Beyotime Biotechnology, C3601) according to the manufacturer’s protocol.

### Western blot

Western blot was performed as described previously [44]. Cells or patient tissues were lysed in RIPA buffer supplemented with protease inhibitors. Protein concentrations were determined using a BCA assay. The proteins were separated by SDS-PAGE and then transferred onto PVDF membranes (Millipore, IPVH00010). Membranes were blocked with 5% non-fat milk, incubated with primary antibodies overnight at 4 °C, followed by HRP-conjugated secondary antibodies for 1 h at room temperature. Protein bands were visualized using an Enhanced Chemiluminescence Detection Kit (Proteintech, PK10001). All western blot experiments were repeated at least three times with independent samples. Antibodies used in this study are listed in Supplementary Table 1.

### Apoptosis assay

786-O cells were seeded in 6-well plates and treated with different medium containing 2 g/L glucose, 2 g/L fructose, or 1 g/L glucose plus 1 g/L fructose for 48 h, respectively (*n* = 3). Adherent cells were digested using 0.25% trypsin, and suspended cells were also collected. Cells were stained with FITC Annexin V and PI using the FITC Annexin V Apoptosis Detection Kit (BD Pharmingen, 556547) according to the manufacturer’s protocol. The stained cells were analyzed by flow cytometry within 1 h.

### RNA isolation and reverse transcription quantitative PCR (RT-qPCR)

Total RNA was extracted from cells using Super FastPure Cell RNA Isolation Kit (Vazyme, RC102) according to the manufacturer’s protocol. Following ethanol precipitation purification, RNA was reverse-transcribed into cDNA using HiScript III All-in-one RT SuperMix Perfect for qPCR (Vazyme, R333-01) according to the manufacturer’s protocol. Quantitative PCR was performed on the Applied Biosystems QuantStudio 5 thermocycler (Thermo Fisher Scientific, USA) with Taq Pro Universal SYBR qPCR Master Mix (Vazyme, Q712-02) according to the manufacturer’s protocol. The 18S rRNA served as an endogenous reference for normalization, with results calculated using the ΔΔCt method and expressed as relative transcript levels compared to control groups. Primer sequences are detailed in Supplementary Table 2.

### DNA isolation and mtDNA copy number assay

Total genomic DNA was extracted from samples using the TIANamp Genomic DNA kit (TIANGEN BIOTECH, DP304-02) following the manufacturer’s protocol. The mtDNA copy number was quantified through real-time PCR analysis, as previously described [45]. Briefly, 10 ng of purified DNA was amplified using primers targeting the mitochondrial NADH dehydrogenase 1 gene (*MT-ND1*) as the mitochondrial marker, with nuclear β-2-microglobulin (*B2M*) serving as the single-copy reference gene for normalization. Primer sequences are provided in Supplementary Table 3.

### Metabolites extraction for GC-MS and LC-MS analysis

Cells were treated in the indicated media for 24 h or 48 h (*n* = 3). After removing the culture medium, adherent cells were washed twice with pre-chilled PBS, then gently scraped into pre-chilled PBS and collected by centrifugation at 300 × *g* for 5 min at 4 °C. 1 ml precooled extraction solvent (80% methanol and 20% water) was added to cell pellets. After ultrasonic treatment, 500 μL chloroform was added into the mixture. Centrifuge at 12,000 × *g* for 10 min at 4 °C and the supernatant was lyophilized in a vacuum concentrator until completely dried. For GC-MS analysis, lyophilized samples were added with 70 μL O-isobutylhydroxylamine (20 mg/ml) at 85 °C by metal bath for 20 min and then added with 30 μL N-tert-Butyldimethylsilyl-N-Methytrifluoro-acetamide (Sigma) at 85 °C by metal bath for 1 h. Centrifuge at 12,000 × *g* for 15 min at 4 °C and use the supernatant for GC-MS analysis. For LC-MS analysis, lyophilized samples were resuspended with 100 μl of mobile phase buffer A (see below). The metabolites were detected using TSQ Vantage LC-MS interfaced with Ultimate 3000 Liquid Chromatography system (Thermo Scientific) and Triple quadrupole mass spectrometry as previously described [46]. The detection of F1P was performed according to a method previously reported in the literature [47].

### Measurement of labeled metabolites of isotopomers by GC-MS and UPLC-QTOF MS

The detection conditions of GC-MS were as follows: injection temperature: 250 °C, split ratio:1/10, carrier gas was high purity helium gas, flow rate was 50 cm/s, the initial temperature was 110 °C. After running for 4 min, it was heated to 230 °C at a heating rate of 3 °C/min. Then it was heated to 280 °C at a heating rate of 20 °C/min and kept for 2 min. The GC column was 30 m × 0.25 mm × 0.25 mm HP-5ms. The ion source temperature was 200 °C. The ionizing voltage/current was 70 V/150 mA and scan range of m/z was 50-800. Sugars with phosphates were measured by a Nexera UHPLC-LC 30 A (Shimadzu, Japan) coupled with quadruple time-of-flight (QTOF) MS (AB 6600 TripleTOF, SCIEX). LC separation was on a Waters HILIC carbon column (Amide 4.6 100 mm ID 3.5 μm; part no: 186004868, Waters) using a gradient of mobile phase buffer A containing 95% water, 5% acetonitrile, 20 mM ammonium hydroxide, 20 mM ammonium acetate (pH 9.0 at 25 °C) and mobile phase buffer B (100% acetonitrile). The LC gradient was: 0-3 min, 15% A; 3–16 min, to 50% A; 16–20 min, to 85% A; 20–23 min, to 15% A; 23–25 min, 15% A. F1P was analysed by LC-MS according to previously established method [47]. All samples were measured in negative mode. Data were analysed to determine isotope labeling and implemented in MATLAB [48]. Isotope labeling was corrected for natural ^13^C abundance.

### Untargeted metabolomic analysis of 786-O cells by LC-MS/MS

786-O cells were treated with different media for 48 h and harvested (*n* = 3). Metabolites were extracted using pre-chilled methanol/acetonitrile/water (2:2:1, v/v), followed by vortexing, low-temperature sonication for 30 min, incubation at −20 °C for 10 min, and centrifugation at 14,000 × *g* at 4 °C for 20 min. The supernatant was vacuum-dried and reconstituted in acetonitrile/water (1:1, v/v), then centrifuged at 14,000 × *g* at 4 °C for 15 min. LC-MS/MS analysis was performed on a Vanquish UHPLC system coupled with an Orbitrap Exploris™ 480 mass spectrometer (Thermo Fisher Scientific). Chromatographic separation was achieved on a HILIC column at 25 °C with a flow rate of 0.5 mL/min and an injection volume of 2 μL. Mobile phase A consisted of water containing 25 mM ammonium acetate and 25 mM ammonia, and mobile phase B was acetonitrile. The gradient elution program was as follows: 0–0.5 min, 95% B; 0.5–7 min, linear decrease to 65% B; 7–8 min, linear decrease to 40% B; 8–9 min, 40% B; 9–9.1 min, linear increase to 95% B; 9.1–12 min, 95% B. ESI was operated in both positive (spray voltage 3500 V) and negative (spray voltage 2800 V) modes with an ion transfer tube temperature of 350 °C, sheath gas 50, and auxiliary gas 2. Full MS scans were acquired over m/z 70–1200 at a resolution of 60,000 with an injection time of 100 ms. Data-dependent MS/MS was performed at a resolution of 60,000 with a dynamic exclusion of 4 s. Raw data were converted to mzXML format using ProteoWizard and processed with XCMS for peak alignment, retention time correction, and peak area extraction. Features with >50% missing values were removed, missing values were imputed by KNN, and features with RSD > 50% were filtered. Subsequent univariate and multivariate statistical analyses, differential metabolite identification, and bioinformatic interpretation were performed. Metabolomic profiling and data analysis were conducted by Shanghai Applied Protein Technology Co., Ltd. (APTBIO, Shanghai, China).

### Measurement of mitochondrial membrane potential (MMP) and mitochondrial superoxide

ccRCC cells were seeded in 6-well plates and treated with different medium containing either 2 g/L glucose or 1 g/L glucose plus 1 g/L fructose for 48 h, respectively (*n* = 3). The MMP was detected using the Mitochondrial Membrane Potential Assay Kit with TMRE (Beyotime Biotechnology, C2001S) according to the manufacturer’s protocol. CCCP (10 µM) was used as a positive control to induce a decrease in MMP and was added to the cell culture medium 60 min prior to TMRE staining. The mitochondrial superoxide was detected using the Mitochondrial Superoxide Assay Kit with MitoSOX Red (Beyotime Biotechnology, S0061S) according to the manufacturer’s protocol. mSoxUp was used as a positive control to induce mitochondrial superoxide production and was added to the cell culture medium 4 h before MitoSOX staining. Both MMP and mitochondrial superoxide were measured using a fluorescence microscope. The fluorescence intensity was quantified using Image J software.

### Sample collection and tissue processing

Fresh ccRCC tissues and paired adjacent normal kidney tissues were obtained from patients undergoing radical or partial nephrectomy at Weifang People’s Hospital. Clinical characteristics of ccRCC patients are summarized in Supplementary Table 4. The samples were cut into small pieces for Western blot and organoid derivation. All participants provided written informed consent prior to sample collection. This study was approved by the Research Ethics Committee of Weifang People’s Hospital (Approval No. KYLL20221214-3).

### Organoid culture

ccRCC tumor tissues were minced into 1–2 mm³ fragments and were subjected to Tumor Tissue Digestion Solution (Biogenous Biotechnology, K601003) for 15 min at 37 °C in a water bath shaker. 2% FBS was added to the digestion suspension to neutralize trypsinization. The digested suspension was centrifuged at 250 × *g* for 3 min. After centrifugation, cells were suspended in basal medium (Biogenous Biotechnology, B213152) and cell suspensions were filtered through a 100 μm cell strainer prior to centrifugation. Cell pellets were resuspended in organoid culture medium and mixed with Matrigel (R&D Systems, 3432-010-01) to achieve a final concentration >70%. 3 × 10^4^ cells in 40 μL droplets were plated into prewarmed 24-well plates. Medium was refreshed every 2–3 days.

### ATP-based organoid viability assay

2 × 10^3^ ccRCC organoids were seeded in 5 μL droplets comprising a mixture of organoid culture medium and Matrigel in 96-well plates. The ccRCC organoids were treated with either 5 g/L glucose or 2.5 g/L glucose plus 2.5 g/L fructose for 72 h (*n* = 3). Organoid viability was quantified by measuring ATP content using the CellTiter-Meiluncell Luminescent Cell Viability Assay Kit (Meilun Biotechnology, PWL111) according to the manufacturer’s protocol.

### Calcein acetoxymethyl ester/propidium iodide (calcein-AM/PI) co-staining of organoids

Calcein-AM/PI co-staining was performed to assess live and dead cells in ccRCC organoids using Calcein/PI Cell Viability/Cytotoxicity Assay Kit (Beyotime Biotechnology, C2015S) according to the manufacturer’s protocol. Live cells emitted green fluorescence (Ex/Em 494/517 nm), whereas dead cells exhibited red nuclear staining (Ex/Em 535/617 nm). Fluorescent images were acquired a Keyence BZ-X800LE live cell workstation.

### Measurement of mitochondria respiration

ccRCC cells were seeded at a density of 1 × 10^4^ cells per well (*n* = 3) in XF 24-well microplates (Agilent Technologies, 100777-004). After adherence, cells were washed and incubated in the indicated treatment medium for 24 h. The oxygen consumption rate (OCR) was measured using a Seahorse XFe-24 Extracellular Flux Analyzer (Agilent Technologies, USA). MitoStress analyses were performed with sequential injections of 1 μM oligomycin, 1 μM carbonyl cyanide-4-(trifluoromethoxy) phenylhydrazone (FCCP), 1 μM rotenone, and 1 μM antimycin A at final concentrations, as detailed previously [49]. After Seahorse, cells were incubated with 5 µg/mL Hoechst 33342 (MedChemExpress, HY-15559) in complete medium for 20 min at 37 °C, protected from light, then washed with PBS. Images were acquired using a fluorescence microscope (excitation 350–370 nm, emission 461 nm) with a DAPI filter cube. Five random fields per well were captured. Nuclei were counted using Image J software. OCR values were normalized to cell number (1 × 10⁵ cells per well).

For organoid OCR analysis, organoids were cultured in either 5 g/L glucose or 2.5 g/L glucose plus 2.5 g/L fructose, treated with or without 0.5 mM 4-PBA or 25 µM nucleosides (guanosine, adenosine, and uridine) for 48 h, then harvested for mitochondrial respiration analysis. Organoids were dissociated, counted, and resuspended at 2.5 × 10⁴ cells per 100 µL pre-warmed Seahorse assay medium. Seahorse XF24 cell culture plates (Agilent, 100777-004) were pre-coated at 4 °C overnight with rat tail collagen I (Corning, 354236; 50 µL/well, diluted in 0.02 N acetic acid) and washed twice with PBS before use. Cell suspension was added to the coated plates (*n* = 3), centrifuged at 200 × *g* for 1 min (brake = 0), and incubated at 37 °C without CO₂ for 25–30 min to allow attachment. Then 400 µL pre-warmed assay medium was gently added, followed by 15–25 min equilibration before MitoStress analysis on the Seahorse XFe24 analyzer. Sequential injections: 1.5 µM oligomycin, 0.5 µM FCCP, and 0.5 µM rotenone/antimycin A. OCR values were normalized to cell number (1 × 10⁵ cells/well).

### Xenograft tumor growth mouse model

5 × 10^6^ 786-O cells suspended in a 100 μL 1:1 mixture of RPMI 1640 medium and Cultrex Basement Membrane Extract (R&D Systems, 3632-010-02) were subcutaneously injected into the flank of 5-week-old male NOD scid mice (Beijing Vital River Laboratory Animal Technology Co., Ltd). On day 11 post-inoculation, mice were randomly divided into 3 groups (*n* = 6) with *ad libitum* access to control water (group 1) or 10% fructose water (group 2 and 3). Mice that failed to develop tumors were excluded. All solutions were provided in bottles renewed twice weekly. Concurrently, mice were administrated with vehicle (0.9% NaCl solution, group 1 and 2) or 150 mg/kg 4-phenylbutyric acid (4-PBA, MedChemExpress, HY-15654, group 3) via intraperitoneal injection every other day for 7 weeks. No statistical methods were used to predetermine sample size. The sample size of n = 6 per group was based on our previous experience with this animal model [50]. Mice were randomly assigned to experimental groups using a random number table. Tumor volume and body weight were recorded weekly. After euthanasia, tumors were excised, weighed, and sectioned for further pathologic analysis. Tumor volume was calculated using formula V = 0.5 × a × b^2^ where “a” is tumor length and “b” is tumor width. All procedures were approved by the Institutional Animal Care and Use Committee of the Medical Laboratory Animal Center at Shandong Second Medical University (IACUC Approval No. 2025SDL626).

### RNA-sequence analysis

786-O cells were treated with either 2 g/L glucose or 1 g/L glucose plus 1 g/L fructose for 24 h (*n* = 3). Total RNA was extracted using TRlzol reagent. The library preparation and sequencing were carried out by Biomarker Technologies Co., Ltd (Beijing, China). The raw reads were further processed with a bioinformatic pipelinetool, BMKCloud (www.biocloud.net) online platform. Differential expression analysis of two groups was performed using the DESeq2. The resulting *P* values were adjusted using the Benjamini and Hochberg’s approach for controlling the false discovery rate. Genes with an adjusted *P* value < 0.01 & Fold Change≥2 found by DESeq2 were assigned as differentially expressed. Gene Ontology (GO) enrichment analysis of the differentially expressed genes (DEGs) was implemented by the clusterProfiler packages based Wallenius non-central hyper-geometric distribution. KOBAS database and clusterProfiler software were used to test the statistical enrichment of DEGs in KEGG pathways. The raw RNA-seq data of 786-O cells have been deposited in the NCBI BioProject database under accession number PRJNA1394179. We have included the complete normalized gene expression matrix (FPKM values) for all samples as Supplementary Table 5.

### Statistical analysis

Sample sizes were determined based on preliminary experiments and prior publications, with at least three independent biological replicates per condition. All exclusion criteria were pre-established prior to data collection. The investigator performing Western blot quantification and tumor volume measurements was blinded to group allocation. Data are presented as mean ± SEM from at least three independent experiments. Statistical analyses were performed using GraphPad Prism 9.0 (GraphPad Software, San Diego, CA, USA). *P* < 0.05 was considered statistically significant. Normality was assessed using the Shapiro-Wilk test. When the assumption of normality was violated, data were either log-transformed to achieve normality or analyzed using non-parametric tests. Homogeneity of variance was confirmed using Levene’s test (*P* > 0.05). In cases where variances were unequal, Welch’s correction was applied. Group comparisons were performed using two-tailed independent t-tests or one-way ANOVA with Tukey’s post hoc correction. Two-way ANOVA was used to examine the main effects of treatment and time point, as well as their interaction effect. All tests were appropriately selected based on the experimental design and data structure. All tests were two-sided with a significance level of α = 0.05. Detailed statistical methods and sample sizes for each experiment are provided in the corresponding figure legends. *P < 0.05; **P < 0.01; ***P < 0.001.

## Supplementary information


Supplementary figures+legends
Supplementary Tables
original WB blots


## Data Availability

The raw RNA-seq data of 786-O cells have been deposited in the NCBI BioProject database under accession number PRJNA1394179. All other raw data of this study are available from the corresponding author on reasonable request.

## References

[1] Bray F, Laversanne M, Sung H, Ferlay J, Siegel RL, Soerjomataram I, et al. Global cancer statistics 2022: GLOBOCAN estimates of incidence and mortality worldwide for 36 cancers in 185 countries. CA: A Cancer J Clin. 2024;74:229–63. 10.3322/caac.21834 38572751

[2] Wettersten HI, Aboud OA, Lara PN Jr, Weiss RH. Metabolic reprogramming in clear cell renal cell carcinoma. Nat Rev Nephrol. 2017;13:410–9. 28480903 10.1038/nrneph.2017.59

[3] Jung S, Bae H, Song WS, Jang C. Dietary fructose and fructose-induced pathologies. Annu Rev Nutr. 2022;42:45–66. 35995049 10.1146/annurev-nutr-062220-025831 PMC9904196

[4] Jang C, Hui S, Lu W, Cowan AJ, Morscher RJ, Lee G, et al. The small intestine converts dietary fructose into glucose and organic acids. Cell Metab. 2018;27:351–61.e3. 29414685 10.1016/j.cmet.2017.12.016 PMC6032988

[5] Vallon V, Nakagawa T. Renal tubular handling of glucose and fructose in health and disease. Compr Physiol. 2021;12:2995–3044. 34964123 10.1002/cphy.c210030 PMC9832976

[6] Forester BR, Zhang R, Schuhler B, Brostek A, Gonzalez-Vicente A, Garvin JL. Knocking out sodium glucose-linked transporter 5 prevents fructose-induced renal oxidative stress and salt-sensitive hypertension. Hypertension (Dallas, Tex : 1979). 2024;81:1296–307. 38545789 10.1161/HYPERTENSIONAHA.123.22535 PMC11096007

[7] Hannou SA, Haslam DE, McKeown NM, Herman MA. Fructose metabolism and metabolic disease. J Clin Investig. 2018;128:545–55. 29388924 10.1172/JCI96702 PMC5785258

[8] Nakagawa T, Johnson RJ, Andres-Hernando A, Roncal-Jimenez C, Sanchez-Lozada LG, Tolan DR, et al. Fructose production and metabolism in the kidney. J Am Soc Nephrol. 2020;31:898–906. 32253274 10.1681/ASN.2019101015 PMC7217403

[9] Guo H, Fang T, Cheng Y, Li T, Qu JR, Xu CF, et al. ChREBP-β/TXNIP aggravates frucose-induced renal injury through triggering ferroptosis of renal tubular epithelial cells. Free Radic Biol Med. 2023;199:154–65. 36828294 10.1016/j.freeradbiomed.2023.02.013

[10] Cirillo P, Gersch MS, Mu W, Scherer PM, Kim KM, Gesualdo L, et al. Ketohexokinase-dependent metabolism of fructose induces proinflammatory mediators in proximal tubular cells. J Am Soc Nephrol. 2009;20:545–53. 19158351 10.1681/ASN.2008060576 PMC2653686

[11] Wang J, Wu Q, Qiu J. Accumulation of fructose 1,6-bisphosphate protects clear cell renal cell carcinoma from oxidative stress. Lab Investig. 2019;99:898–908. 30760861 10.1038/s41374-019-0203-3

[12] Chen WL, Wang YY, Zhao A, Xia L, Xie G, Su M, et al. Enhanced fructose utilization mediated by SLC2A5 is a unique metabolic feature of acute myeloid leukemia with therapeutic potential. Cancer cell. 2016;30:779–91. 27746145 10.1016/j.ccell.2016.09.006 PMC5496656

[13] Jeong S, Savino AM, Chirayil R, Barin E, Cheng Y, Park SM, et al. High fructose drives the serine synthesis pathway in acute myeloid leukemic cells. Cell Metab. 2021;33:145–59.e6. 33357456 10.1016/j.cmet.2020.12.005 PMC8168776

[14] Goncalves MD, Lu C, Tutnauer J, Hartman TE, Hwang SK, Murphy CJ, et al. High-fructose corn syrup enhances intestinal tumor growth in mice. Sci (N Y, NY). 2019;363:1345–9. 10.1126/science.aat8515 PMC6487857 30898933

[15] Fan X, Liu H, Liu M, Wang Y, Qiu L, Cui Y. Increased utilization of fructose has a positive effect on the development of breast cancer. PeerJ. 2017;5:e3804. 28970966 10.7717/peerj.3804 PMC5622605

[16] Chen WL, Jin X, Wang M, Liu D, Luo Q, Tian H, et al. GLUT5-mediated fructose utilization drives lung cancer growth by stimulating fatty acid synthesis and AMPK/mTORC1 signaling. JCI insight. 2020;5:e131596. 10.1172/jci.insight.131596 PMC7098789 32051337

[17] Tee SS, Kim N, Cullen Q, Eskandari R, Mamakhanyan A, Srouji RM, et al. Ketohexokinase-mediated fructose metabolism is lost in hepatocellular carcinoma and can be leveraged for metabolic imaging. Sci Adv. 2022;8:eabm7985. 35385296 10.1126/sciadv.abm7985 PMC8985914

[18] Dewdney B, Alanazy M, Gillman R, Walker S, Wankell M, Qiao L, et al. The effects of fructose and metabolic inhibition on hepatocellular carcinoma. Sci Rep. 2020;10:16769. 33028928 10.1038/s41598-020-73653-5 PMC7541473

[19] Zhang Y, Yu X, Bao R, Huang H, Gu C, Lv Q, et al. Dietary fructose-mediated adipocyte metabolism drives antitumor CD8(+) T cell responses. Cell Metab. 2023;35:2107–18.e6. 37863051 10.1016/j.cmet.2023.09.011

[20] Medina Villaamil V, Aparicio Gallego G, Valbuena Rubira L, García Campelo R, Valladares-Ayerbes M, Grande Pulido E, et al. Fructose transporter GLUT5 expression in clear renal cell carcinoma. Oncol Rep. 2011;25:315–23. 21165569 10.3892/or.2010.1096

[21] Aparicio LM, Villaamil VM, Calvo MB, Rubira LV, Rois JM, Valladares-Ayerbes M, et al. Glucose transporter expression and the potential role of fructose in renal cell carcinoma: A correlation with pathological parameters. Mol Med Rep. 2010;3:575–80. 21472282 10.3892/mmr_00000300

[22] Jin X, Liang Y, Liu D, Luo Q, Cai L, Wu J, et al. An essential role for GLUT5-mediated fructose utilization in exacerbating the malignancy of clear cell renal cell carcinoma. Cell Biol Toxicol. 2019;35:471–83. 31102011 10.1007/s10565-019-09478-4

[23] Miao J, Wang D, Pang R, Zhang H, Wu Y, Sun X, et al. Role of fructose in renal cell carcinoma progression. Discov Oncol. 2025;16:897. 40410626 10.1007/s12672-025-02688-9 PMC12102030

[24] Hwa JS, Kim HJ, Goo BM, Park HJ, Kim CW, Chung KH, et al. The expression of ketohexokinase is diminished in human clear cell type of renal cell carcinoma. Proteomics. 2006;6:1077–84. 16372272 10.1002/pmic.200401345

[25] Mäenpää PH, Raivio KO, Kekomäki MP. Liver adenine nucleotides: fructose-induced depletion and its effect on protein synthesis. Sci (N Y, NY). 1968;161:1253–4. 10.1126/science.161.3847.1253 5673437

[26] Liu Y, Fiskum G, Schubert D. Generation of reactive oxygen species by the mitochondrial electron transport chain. J Neurochem. 2002;80:780–7. 11948241 10.1046/j.0022-3042.2002.00744.x

[27] Li H, Zhu H, Xu CJ, Yuan J. Cleavage of BID by caspase 8 mediates the mitochondrial damage in the Fas pathway of apoptosis. Cell. 1998;94:491–501. 9727492 10.1016/s0092-8674(00)81590-1

[28] Kim J, Kang J, Kang YL, Woo J, Kim Y, Huh J, et al. Ketohexokinase-A acts as a nuclear protein kinase that mediates fructose-induced metastasis in breast cancer. Nat Commun. 2020;11:5436. 33116123 10.1038/s41467-020-19263-1 PMC7595112

[29] Gao W, Li N, Li Z, Xu J, Su C. Ketohexokinase is involved in fructose utilization and promotes tumor progression in glioma. Biochem Biophys Res Commun. 2018;503:1298–306. 30031605 10.1016/j.bbrc.2018.07.040

[30] Bu P, Chen KY, Xiang K, Johnson C, Crown SB, Rakhilin N, et al. Aldolase B-mediated fructose metabolism drives metabolic reprogramming of colon cancer liver metastasis. Cell Metab. 2018;27:1249–62.e4. 29706565 10.1016/j.cmet.2018.04.003 PMC5990465

[31] Ashton TM, McKenna WG, Kunz-Schughart LA, Higgins GS. Oxidative phosphorylation as an emerging target in cancer therapy. Clin Cancer Res : J Am Assoc Cancer Res. 2018;24:2482–90. 10.1158/1078-0432.CCR-17-3070 29420223

[32] Boroughs LK, DeBerardinis RJ. Metabolic pathways promoting cancer cell survival and growth. Nat Cell Biol. 2015;17:351–9. 25774832 10.1038/ncb3124 PMC4939711

[33] Starkov AA. The role of mitochondria in reactive oxygen species metabolism and signaling. Ann N Y Acad Sci. 2008;1147:37–52. 19076429 10.1196/annals.1427.015 PMC2869479

[34] Bock FJ, Tait SWG. Mitochondria as multifaceted regulators of cell death. Nat Rev Mol cell Biol. 2020;21:85–100. 31636403 10.1038/s41580-019-0173-8

[35] Phillips MJ, Voeltz GK. Structure and function of ER membrane contact sites with other organelles. Nat Rev Mol cell Biol. 2016;17:69–82. 26627931 10.1038/nrm.2015.8 PMC5117888

[36] Malhotra JD, Kaufman RJ. Endoplasmic reticulum stress and oxidative stress: a vicious cycle or a double-edged sword? Antioxid Redox Signal. 2007;9:2277–93. 17979528 10.1089/ars.2007.1782

[37] Herman MA, Birnbaum MJ. Molecular aspects of fructose metabolism and metabolic disease. Cell Metab. 2021;33:2329–54. 34619074 10.1016/j.cmet.2021.09.010 PMC8665132

[38] Yanai H, Adachi H, Hakoshima M, Katsuyama H. Molecular biological and clinical understanding of the pathophysiology and treatments of hyperuricemia and its association with metabolic syndrome, cardiovascular diseases and chronic kidney disease. Int J Mol Sci. 2021;22:9221. 10.3390/ijms22179221 PMC8431537 34502127

[39] Johnson RJ, Nakagawa T, Sanchez-Lozada LG, Shafiu M, Sundaram S, Le M, et al. Sugar, uric acid, and the etiology of diabetes and obesity. Diabetes. 2013;62:3307–15. 24065788 10.2337/db12-1814 PMC3781481

[40] Masters SL, Lobito AA, Chae J, Kastner DL. Recent advances in the molecular pathogenesis of hereditary recurrent fevers. Curr Opin Allergy Clin Immunol. 2006;6:428–33. 17088647 10.1097/ACI.0b013e3280109b57

[41] Feng T, Luo Q, Liu Y, Jin Z, Skwarchuk D, Lee R, et al. Fructose and glucose from sugary drinks enhance colorectal cancer metastasis via SORD. Nat Metab. 2025;7:2018–32. 40973819 10.1038/s42255-025-01368-w PMC12552132

[42] Brownlee M. Biochemistry and molecular cell biology of diabetic complications. Nature. 2001;414:813–20. 11742414 10.1038/414813a

[43] Zhao X, Fang Y, Yang Y, Qin Y, Wu P, Wang T, et al. Elaiophylin, a novel autophagy inhibitor, exerts antitumor activity as a single agent in ovarian cancer cells. Autophagy. 2015;11:1849–63. 25893854 10.1080/15548627.2015.1017185 PMC4824600

[44] Li X, Cui XX, Chen YJ, Wu TT, Xu H, Yin H, et al. Therapeutic potential of a prolyl hydroxylase inhibitor FG-4592 for Parkinson’s diseases in vitro and in vivo: regulation of redox biology and mitochondrial function. Front Aging Neurosci. 2018;10:121. 29755339 10.3389/fnagi.2018.00121 PMC5935184

[45] Malik AN, Shahni R, Rodriguez-de-Ledesma A, Laftah A, Cunningham P. Mitochondrial DNA as a non-invasive biomarker: accurate quantification using real time quantitative PCR without co-amplification of pseudogenes and dilution bias. Biochem Biophys Res Commun. 2011;412:1–7. 21703239 10.1016/j.bbrc.2011.06.067

[46] Wang N, Liu H, Liu G, Li M, He X, Yin C, et al. Yeast β-D-glucan exerts antitumour activity in liver cancer through impairing autophagy and lysosomal function, promoting reactive oxygen species production and apoptosis. Redox Biol. 2020;32:101495. 32171725 10.1016/j.redox.2020.101495 PMC7076095

[47] Lanaspa MA, Andres-Hernando A, Orlicky DJ, Cicerchi C, Jang C, Li N, et al. Ketohexokinase C blockade ameliorates fructose-induced metabolic dysfunction in fructose-sensitive mice. J Clin Investig. 2018;128:2226–38. 29533924 10.1172/JCI94427 PMC5983342

[48] Portnoy VA, Scott DA, Lewis NE, Tarasova Y, Osterman AL, Palsson B. Deletion of genes encoding cytochrome oxidases and quinol monooxygenase blocks the aerobic-anaerobic shift in Escherichia coli K-12 MG1655. Appl Environ Microbiol. 2010;76:6529–40. 20709841 10.1128/AEM.01178-10 PMC2950451

[49] Liu L, Li T, Liao Y, Wang Y, Gao Y, Hu H, et al. Triose kinase controls the lipogenic potential of fructose and dietary tolerance. Cell Metab. 2020;32:605–18.e7. 32818435 10.1016/j.cmet.2020.07.018

[50] Wang Y, Zhang X, Wang N, Jiang H, Liang N, Du C, et al. Fructose 1-phosphate inhibits mannose phosphate isomerase to suppress hepatocellular carcinogenesis. Signal Transduction Targeted Therapy. 2026;11:195. 10.1038/s41392-026-02695-4 PMC13199471 42178306

