# Supplementary material for: Fructose induces PERK-dependent ER stress and mitochondrial dysfunction via nucleotide depletion in clear cell renal cell carcinoma
Source: Cell Death Dis. 2026 Aug 7;17(1):766. doi: 10.1038/s41419-026-09157-3 (PMC13530255; doi:10.1038/s41419-026-09157-3)
Supplement: Supplementary file 1 [file 41419_2026_9157_MOESM1_ESM.pdf]

Figure S1

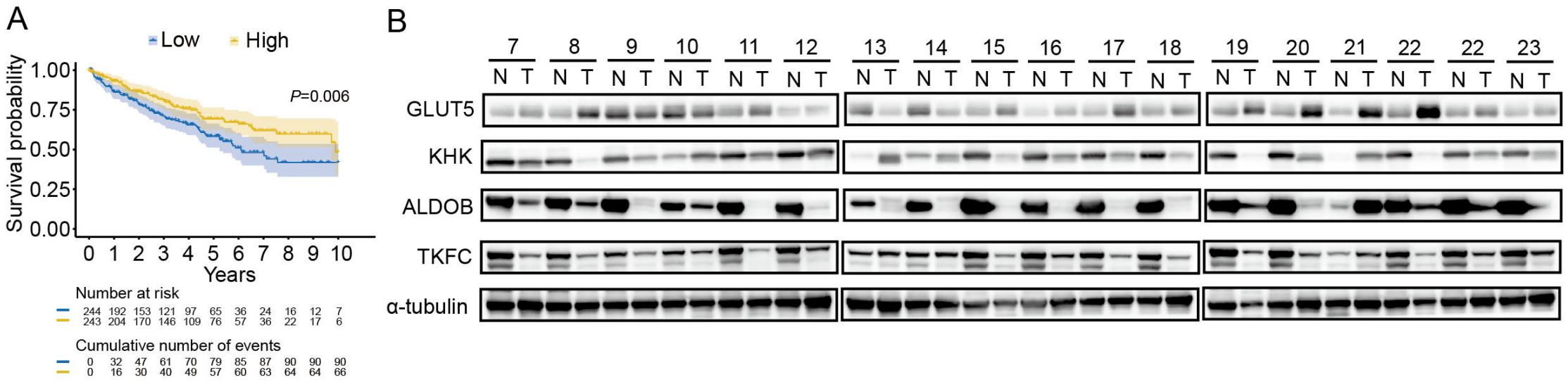

Figure S1. Fructose metabolic pathway analysis in ccRCC patients. (A) Kaplan-Meier overall survival analysis of a fructose-metabolism gene set (*SLC2A5*, *KHK*, *ALDOB*, and *TKFC*) in ccRCC patients based on TCGA data from cSurvival (https://tau.cmmt.ubc.ca/cSurvival/). **(B)** Western blots of proteins involved in fructose metabolism in the remaining 18 paired tumor (T) and adjacent normal (N) tissue samples (completing the cohort of n = 24).

Figure S2

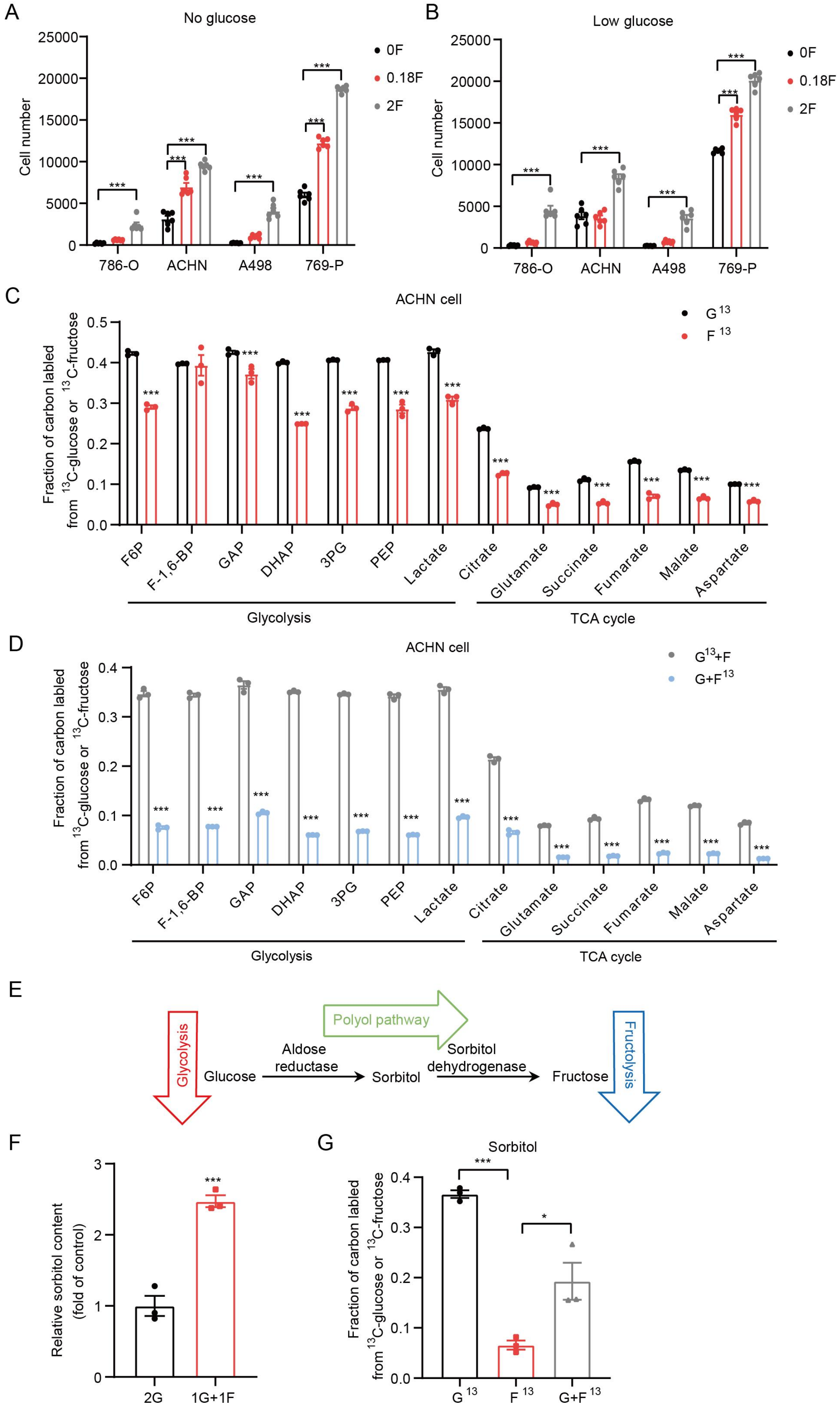

A
No glucose
Cell number
0F
0.18F
2F
786-O
ACHN
A498
769-P
B
Low glucose
Cell number
0F
0.18F
2F
786-O
ACHN
A498
769-P
C
ACHN cell
G 13
F 13
Fraction of carbon labled from 13C-glucose or 13C-fructose
F6P
F-1,6-BP
GAP
DHAP
3PG
PEP
Lactate
Citrate
Glutamate
Succinate
Fumarate
Malate
Aspartate
Glycolysis
TCA cycle
D
ACHN cell
G13+F
G+F13
Fraction of carbon labled from 13C-glucose or 13C-fructose
F6P
F-1,6-BP
GAP
DHAP
3PG
PEP
Lactate
Citrate
Glutamate
Succinate
Fumarate
Malate
Aspartate
Glycolysis
TCA cycle
E
Polyol pathway
Glycolysis
Glucose
Aldose reductase
Sorbitol
Sorbitol dehydrogenase
Fructose
Fructolysis
F
Relative sorbitol content (fold of control)
2G
1G+1F
G
Sorbitol
Fraction of carbon labled from 13C-glucose or 13C-fructose
G 13
F 13
G+F 13

Figure S2. Fructose metabolism exerts glucose-dependent effects on ccRCC cells. (A-B) Viability of ccRCC cells cultured in media containing no glucose **(A)** or low glucose (0.18 g/L glucose) **(B)** supplemented with different concentrations of fructose for 72 h. 0F, no fructose; 0.18F, 0.18 g/L fructose; 2F, 2 g/L fructose. **(C, D)** Fraction of labeled metabolites from glycolysis and the TCA cycle in ACHN cells, derived from U-^{13}C-glucose or U-^{13}C-fructose. Cells were treated for 24 h with: (**C**) 1 g/L U-^{13}C-glucose plus 1 g/L unlabeled glucose (G^{13}), or 1 g/L U-^{13}C-fructose plus 1 g/L unlabeled fructose (F^{13}); (**D**) 1 g/L U-^{13}C-glucose plus 1 g/L unlabeled glucose and 2 g/L unlabeled fructose (G^{13}+F), or 1 g/L U-^{13}C-fructose plus 1 g/L unlabeled fructose and 2 g/L unlabeled glucose (G+F^{13}), n = 3. **(E)** Schematic diagram of the polyol pathway. Glucose is converted to sorbitol by aldose reductase, and sorbitol is further oxidized to fructose by sorbitol dehydrogenase. **(F)** Relative sorbitol content in 786-O cells treated with 2G or 1G+1F media for 48 h, normalized to the 2G group (n = 3). **(G)** Fraction of labeled sorbitol derived from U-^{13}C-glucose or U-^{13}C-fructose in 786-O cells treated for 24 h (n = 3). All data are presented as mean ± SEM. *P* values were determined by one-way ANOVA with Tukey's multiple comparisons test **(A, B, G)**, multiple unpaired t-tests with FDR correction **(C, D)**, and two-tailed unpaired t-test **(F)**. $^{*}P < 0.05$; $^{***}P < 0.001$.

Figure S3

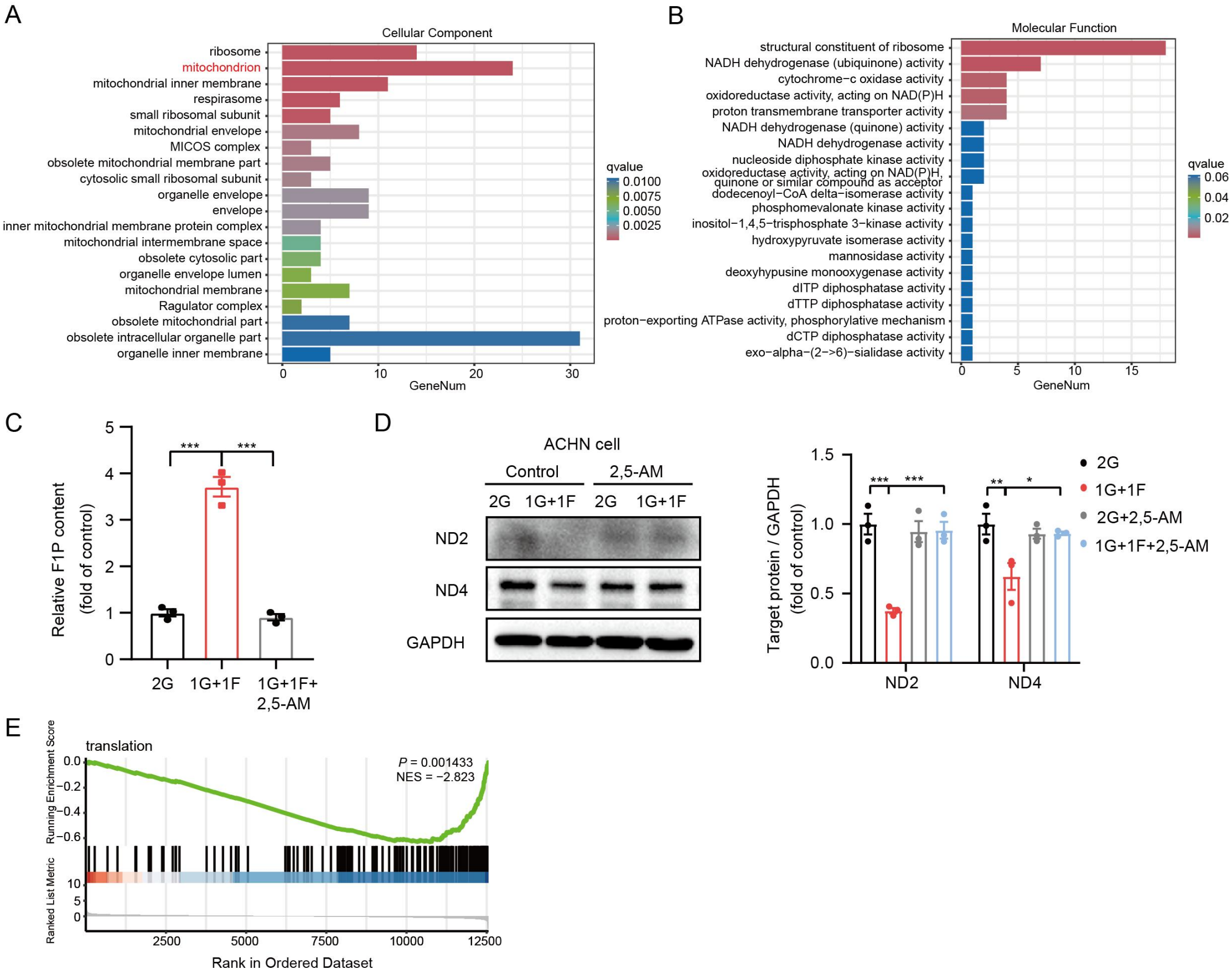

Figure S3. Fructose attenuates oxidative phosphorylation and impairs complex I subunit expression in ccRCC cells in the presence of glucose. (A) GO enrichment analysis (Cellular Component) of DEGs in 786-O cells (1G+1F vs 2G). **(B)** GO enrichment analysis (Molecular Function) of DEGs in 786-O cells (1G+1F vs 2G). **(C)** Relative F1P content in 786-O cells treated with the indicated media and 2,5-AM for 48 h, normalized to the 2G group (n = 3). **(D)** Left panel: The protein expression of ND2 and ND4 in ACHN cells treated with the indicated media and 2,5-AM for 48 h. Right panel: Quantitative analysis of the protein expression of ND2 and ND4 normalized to GAPDH and expressed as fold change relative to the 2G control group (n = 3). All data are presented as mean ± SEM. **(E)** Gene set enrichment analysis (GSEA) of the "Translation" gene signature in 786-O cells treated with 1G+1F vs 2G for 24 h. *P* values were determined by one-way ANOVA with Tukey's multiple comparisons test **(C, D)**. $^{*}P < 0.05$; $^{**}P < 0.01$; $^{***}P < 0.001$.

Figure S4

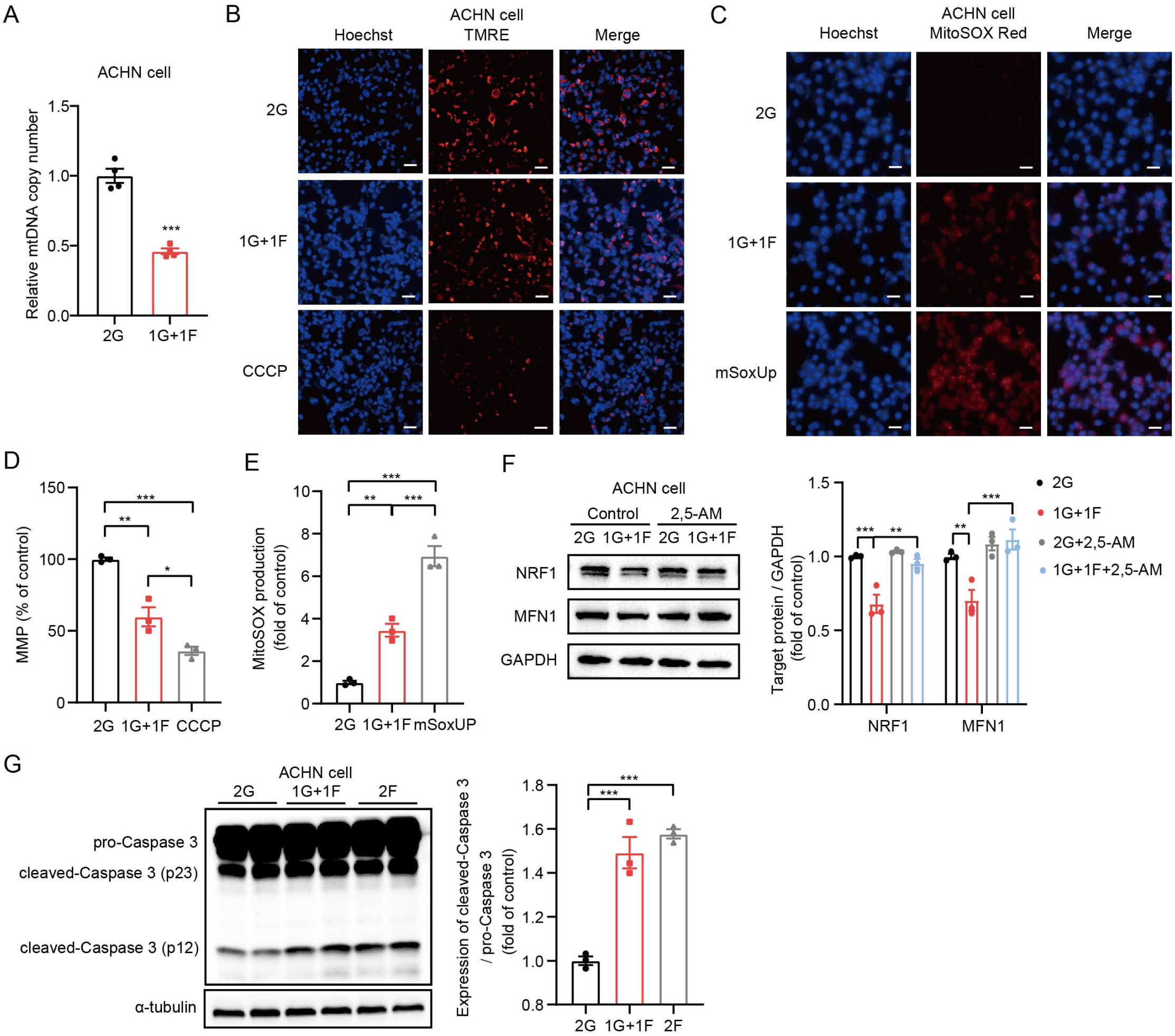

Figure S4. Fructose impairs mitochondrial function and sensitizes ccRCC cells to nutrient stress via intrinsic apoptosis in the presence of glucose. (A) Relative mtDNA copy number in treated ACHN cells for 24 h (normalized to the 2G group, n = 3). **(B, C)** Representative fluorescence images of MMP stained with TMRE **(B)** and mitochondrial superoxide stained with MitoSOX Red **(C)** in treated ACHN cells for 24 h. Scale bars: 50 µm **(B)**. Scale bars: 25 µm **(C)**. **(D, E)** Quantitative analysis of TMRE **(D)** and MitoSOX Red **(E)** fluorescence intensity from **(B)** and **(C)** (normalized to the 2G control group, n = 3), respectively. **(F)** Left panel: The protein expression of NRF1 and MFN1 in ACHN cells treated with 2G, 1G+1F, or in combination with 2,5-AM (2 mM) for 48 h. Right panel: Quantitative analysis of the protein expression normalized to GAPDH and expressed as fold change relative to the 2G control group (n = 3). **(G)** Left panel: The protein expression of pro-Caspase 3 and its cleaved active forms (cleaved-Caspase 3, p23 and p12) in ACHN cells treated with the indicated media for 48 h under serum-free conditions. Right panel: Quantitative analysis of cleaved-Caspase 3 (p23 and p12) / pro-Caspase 3 and expressed as fold change relative to the 2G control group (n = 3). All data are presented as mean ± SEM. *P* values were determined by two-tailed unpaired t-test **(A)** and one-way ANOVA with Tukey's multiple comparisons test **(D-G)**. $^{**}P < 0.01$; $^{***}P < 0.001$.

Figure S5

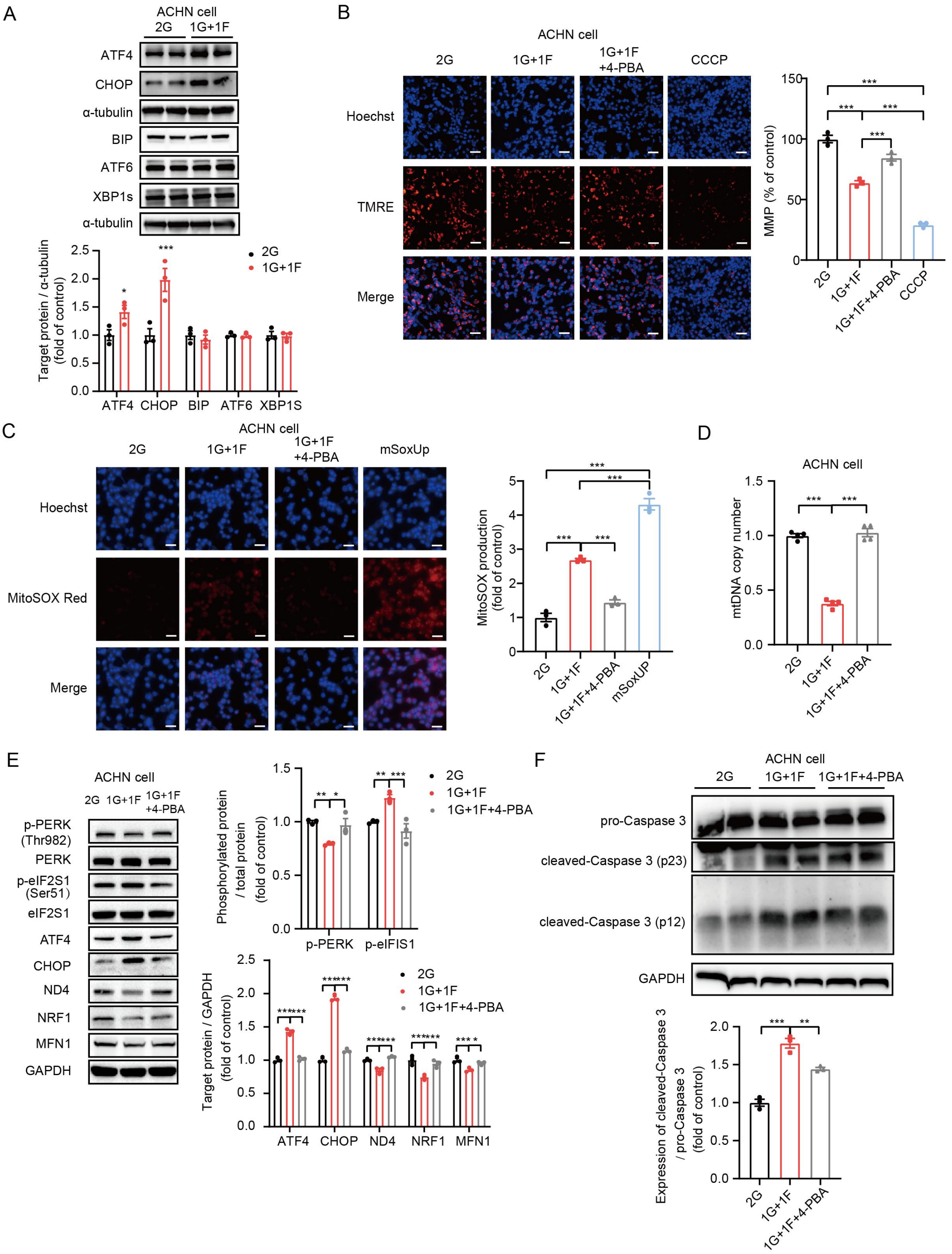

Figure S5. PERK-dependent ER stress mediates fructose-induced mitochondrial dysfunction in ccRCC cells. (A) Upper panel: The protein expression of ER stress markers (ATF4, CHOP, BIP, ATF6, XBP1s) in ACHN cells treated with the indicated media for 48 h. Lower panel: Quantitative analysis of the protein expression normalized to α-tubulin and expressed as fold change relative to the 2G control group (n = 3). **(B, C)** Left panel: Representative fluorescence images of MMP stained with TMRE **(B)** and mitochondrial superoxide stained with MitoSOX Red **(C)** in treated ACHN cells for 24 h. Scale bars: 50 μm **(B).** Scale bars: 25 μm **(C)**. Right panel: Quantitative analysis of TMRE and MitoSOX Red fluorescence intensity from **(B)** and **(C)** (normalized to the 2G

control group, n = 3), respectively. **(D)** Relative mtDNA copy number in treated ACHN cells for 24 h (normalized to the 2G control group, n = 3). **(E)** Left panel: The protein expression of ER stress markers (p-PERK, PERK, p-eIF2S1, eIF2S1, ATF4, CHOP) and mitochondrial proteins (ND4, NRF1, MFN1) in ACHN cells treated with the indicated media for 48 h. Right panels: Quantitative analysis of the protein expression normalized to GAPDH and expressed as fold change relative to the 2G control group (n = 3). **(F)** Upper panel: The protein expression of pro-Caspase 3 and its cleaved active forms (cleaved-Caspase 3, p23 and p12) in ACHN cells treated with the indicated media for 48 h under serum-free conditions. Lower panel: Quantitative analysis of cleaved-Caspase 3 (p23 and p12) / pro-Caspase 3 and expressed as fold change relative to the 2G control group (n = 3). All data are presented as mean ± SEM. *P* values were determined by multiple unpaired t-tests with FDR correction **(A)** and one-way ANOVA with Tukey's multiple comparisons test **(B-F)**. *$P < 0.05$; **$P < 0.01$; ***$P < 0.001$.

Figure S6

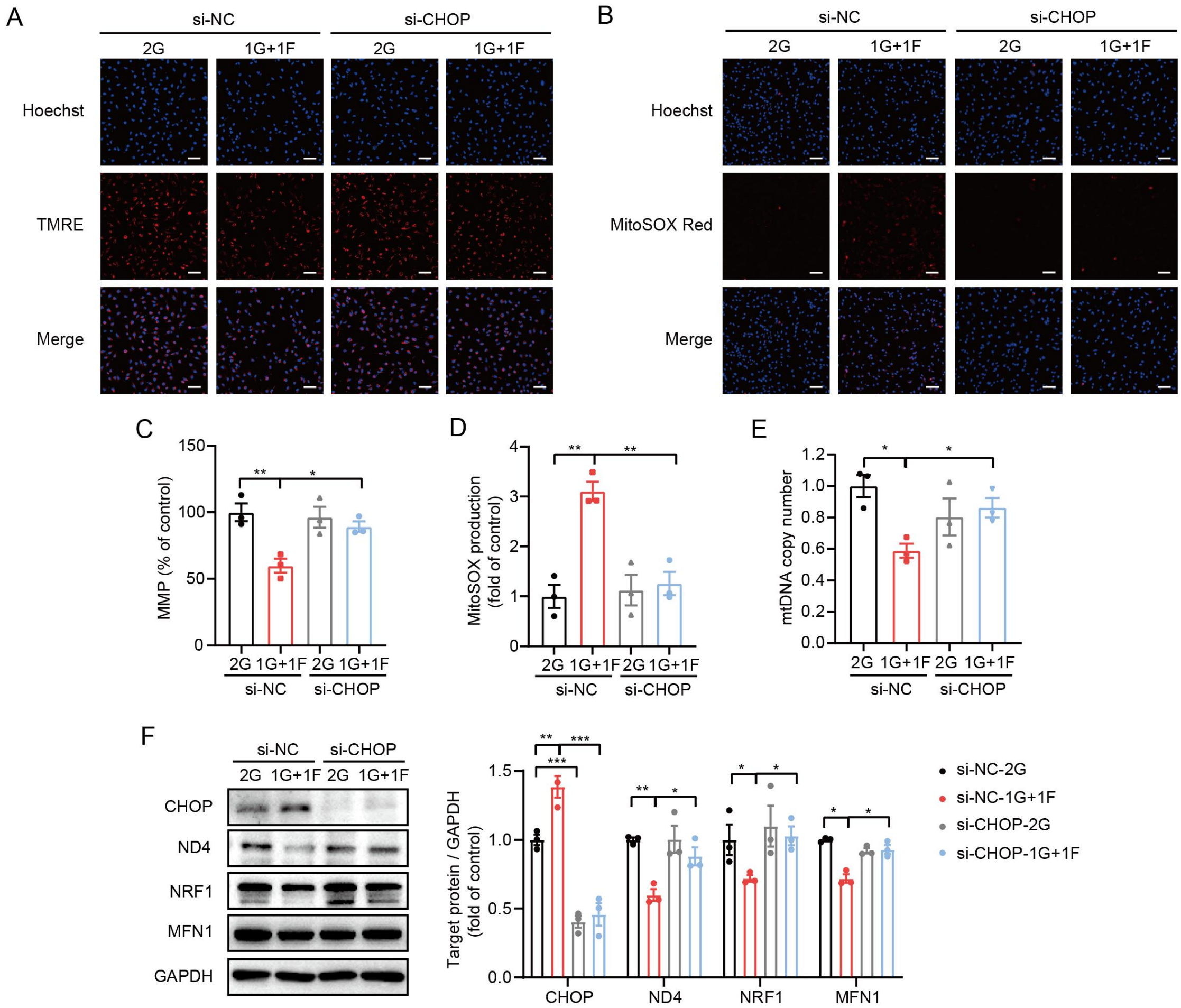

Figure S6. CHOP knockdown rescues fructose-induced mitochondrial dysfunction in ccRCC cells. (A, B) Representative fluorescence images of MMP stained with TMRE **(A)** and mitochondrial superoxide stained with MitoSOX Red **(B)** in 786-O cells transfected with si-NC or si-CHOP, followed by treatment with 2G or 1G+1F for 48 h. Scale bars, 100 µm. **(C, D)** Quantitative analysis of TMRE **(C)** and MitoSOX Red **(D)** fluorescence intensity from **(A)** and **(B)** (normalized to the 2G si-NC group, n = 3), respectively. **(E)** Relative mtDNA copy number in treated 786-O cells for 24 h (normalized to the 2G si-NC group, n = 3). **(F)** Left panel: The protein expression of CHOP, ND4, NRF1, MFN1 in 786-O cells transfected with si-NC or si-CHOP, followed by treatment with 2G or 1G+1F for 48 h. Right panel: Quantitative analysis of the protein expression normalized to GAPDH and expressed as fold change relative to the 2G si-NC group (n = 3). All data are presented as mean ± SEM. *P* values were determined by one-way ANOVA with Tukey's multiple comparisons test **(C-F)**. *$P < 0.05$; **$P < 0.01$; ***$P < 0.001$.

Figure S7

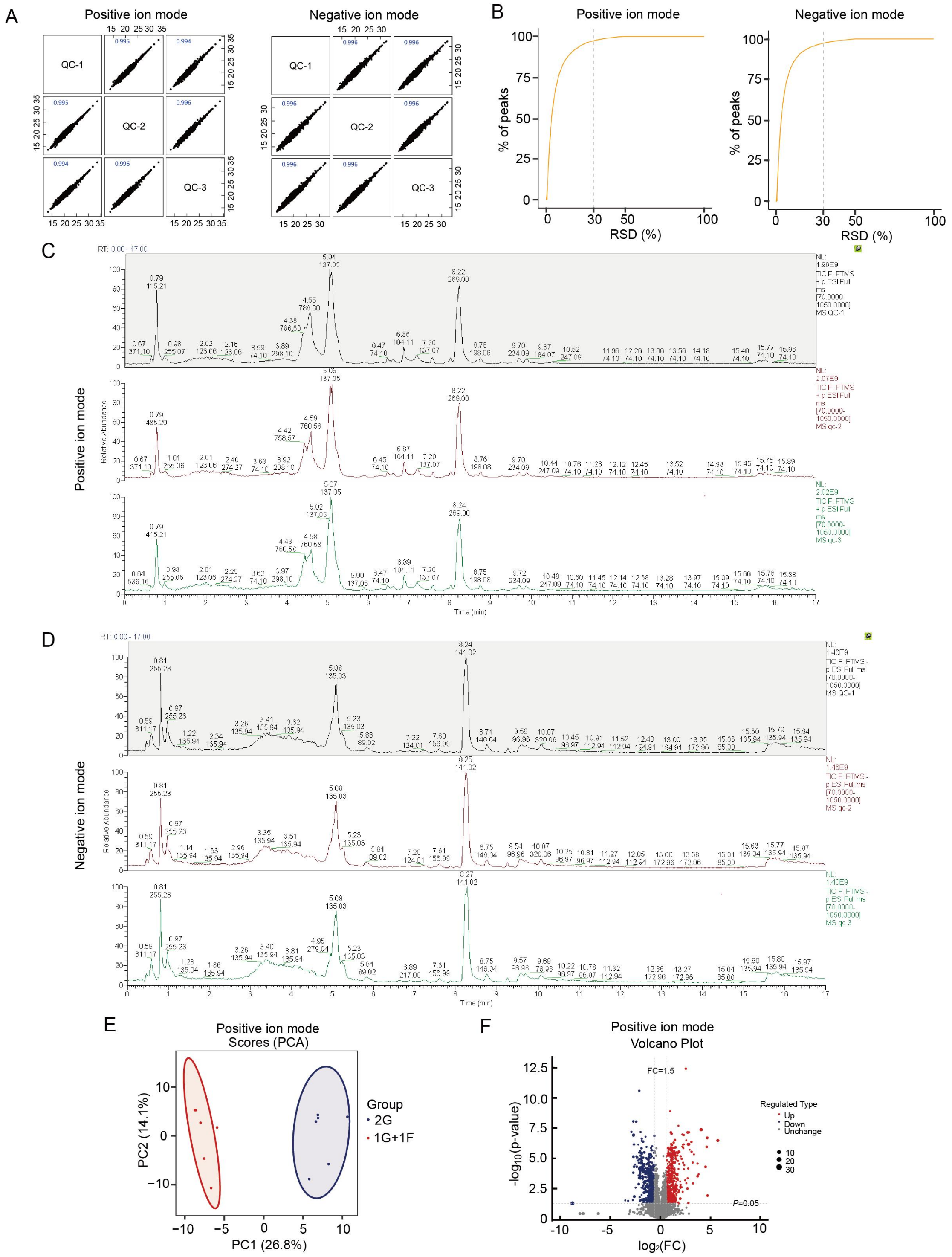

Figure S7. Quality control and differential metabolite analysis of untargeted metabolomics data. (A) Correlation analysis of quality control (QC) samples in positive (left) and negative (right) ion modes. Each plot displays pairwise comparisons of QC samples, with the diagonal representing the 1:1 reference line. Pearson correlation coefficients are shown, reflecting the reproducibility and stability of the analytical system. **(B)** Distribution of relative standard deviation (RSD) for metabolite peaks detected in QC samples in positive (left) and negative (right) ion modes. The dashed line indicates RSD = 30%, demonstrating good repeatability of the

dataset. **(C-D)** Representative total ion chromatograms (TICs) of samples (QC, control group, and treatment group) acquired in positive ion mode **(C)** and negative ion mode **(D)**. The x-axis is retention time (RT, min), and the y-axis is relative abundance, illustrating the overall metabolic profiles and peak consistency across samples. **(E)** Principal component analysis (PCA) score plot of metabolic profiles in positive ion mode from 786-O cells treated with 2G or 1G+1F for 48 h. The x-axis (PC1, 26.8% variance explained) and y-axis (PC2, 14.1% variance explained) show clear separation between the control group (2G, purple) and the treatment group (1G+1F, red). Ellipses represent 95% confidence intervals. **(F)** Volcano plot of differentially expressed metabolites ($|\log_2 FC| > 0.5$, $P < 0.05$) in positive ion mode between the 2G and 1G+1F groups.

Figure S8

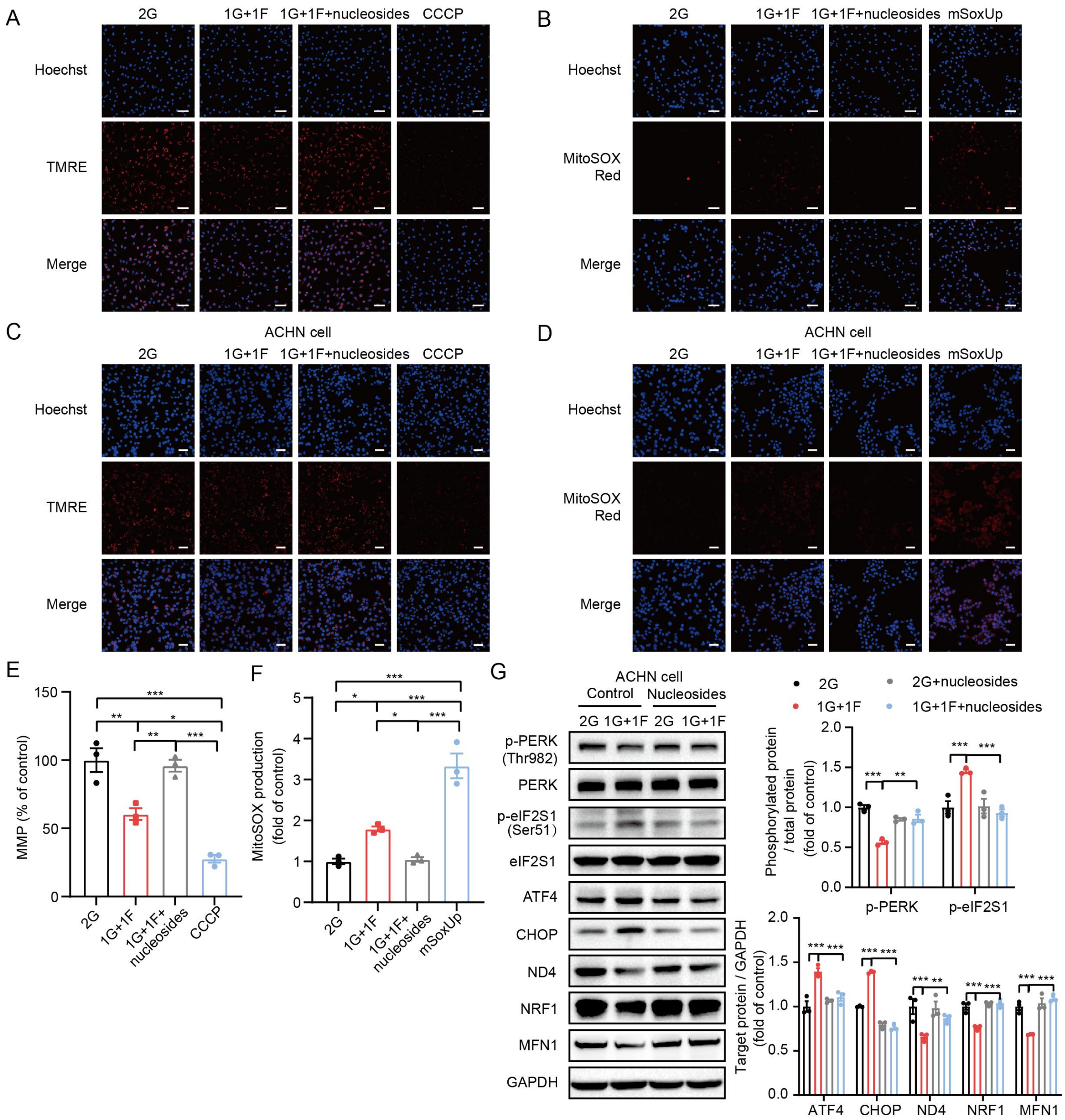

Figure S8. Nucleotide pool depletion mediates fructose-induced PERK-dependent ER stress and mitochondrial dysfunction in ccRCC cells. (A, B) Representative fluorescence images of MMP stained with TMRE **(A)** and mitochondrial superoxide stained with MitoSOX Red **(B)** in treated 786-O cells for 24 h. Scale bars: 100 μm. **(C, D)** Representative fluorescence images of MMP stained with TMRE **(C)** and mitochondrial superoxide stained with MitoSOX Red **(D)** in treated ACHN cells for 24 h. Scale bars: 50 μm. **(E, F)** Quantitative analysis of TMRE **(E)** and MitoSOX Red **(F)** fluorescence intensity from **(C)** and **(D)** (normalized to the 2G control group, n = 3), respectively. **(G)** Left panel: The protein expression of ER stress markers (p-PERK, PERK, p-eIF2S1, eIF2S1, ATF4, CHOP) and mitochondrial proteins (ND4, NRF1, MFN1) in ACHN cells treated with the indicated media for 48 h. Right panels: Quantitative analysis of the protein expression normalized to GAPDH and expressed as fold change relative to the 2G control group (n = 3). All data are presented as mean ± SEM. *P* values were determined by one-way ANOVA with Tukey's multiple comparisons test **(E-G)**. **P* < 0.05; ***P* < 0.01; ****P* < 0.001.

Figure S9

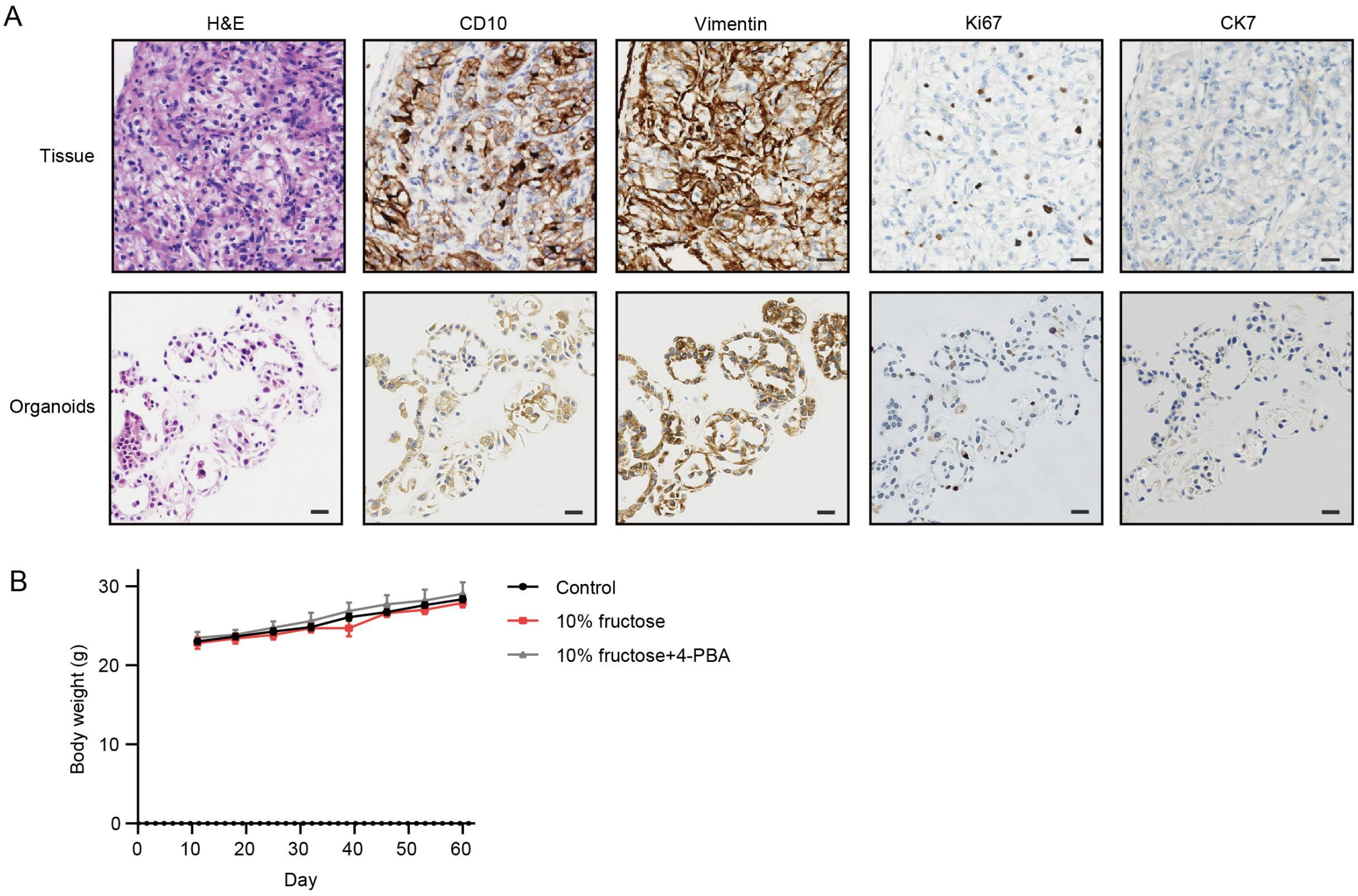

Figure S9. Fructose impairs ccRCC organoid viability and inhibits tumor growth in vivo. (A) Histological and immunostaining characterization of ccRCC tissue vs organoids. Scale bars: 20 μm. **(B)** Body weight changes of mice in xenograft experiment. *P* values were determined by two-way ANOVA with Tukey's multiple comparisons test. Data are presented as mean ± SEM.
