# Supplementary material for: Fructose induces PERK-dependent ER stress and mitochondrial dysfunction via nucleotide depletion in clear cell renal cell carcinoma
Source: Cell Death Dis. 2026 Aug 7;17(1):766. doi: 10.1038/s41419-026-09157-3 (PMC13530255; doi:10.1038/s41419-026-09157-3)
Supplement: Supplementary file 2 [file 41419_2026_9157_MOESM3_ESM.pdf]

Figure1

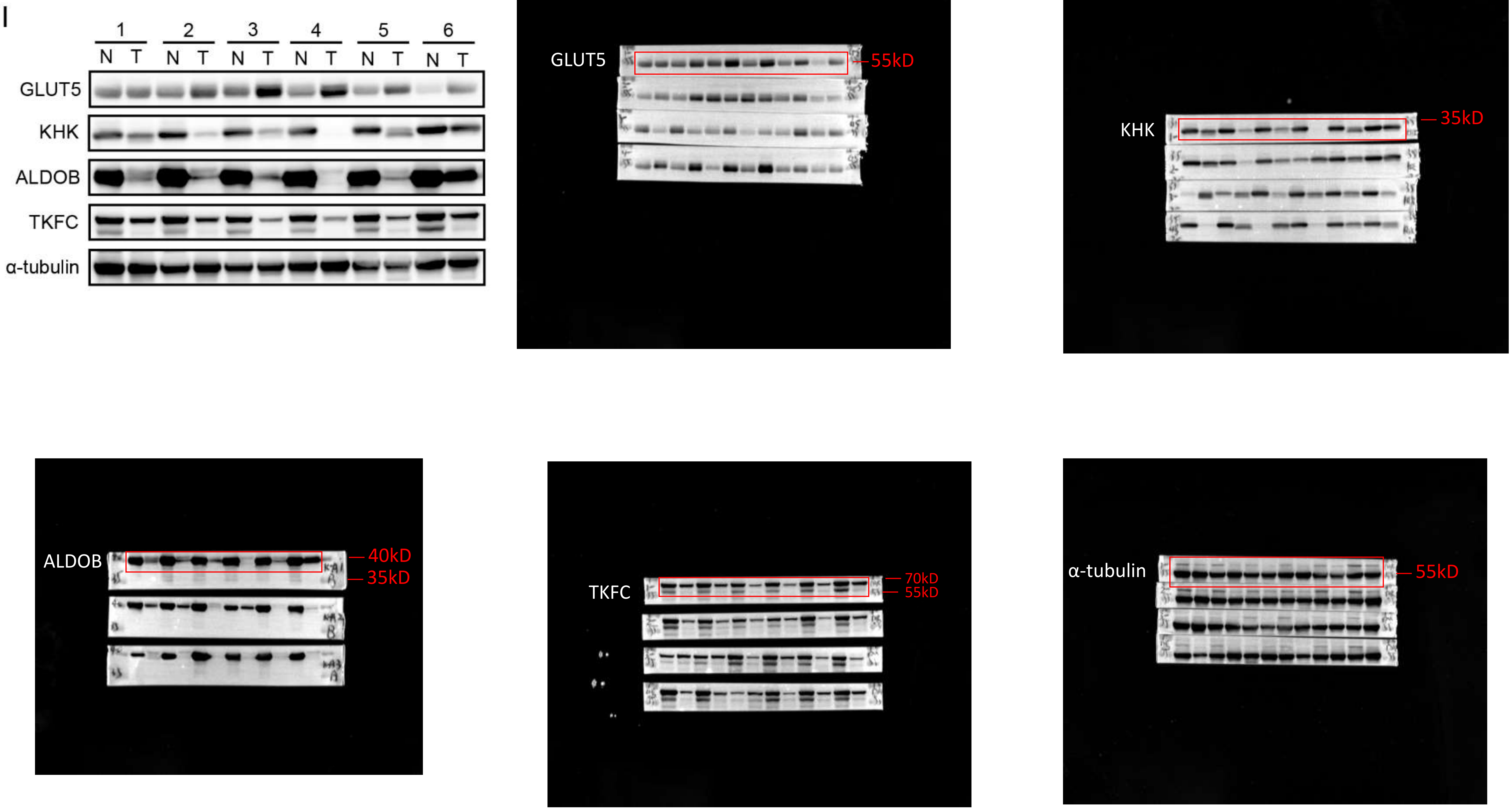
I
1
2
3
4
5
6
N T N T N T N T N T N T
GLUT5
KHK
ALDOB
TKFC
α-tubulin
GLUT5
55kD
KHK
35kD
ALDOB
40kD
35kD
TKFC
70kD
55kD
α-tubulin
55kD

Figure3

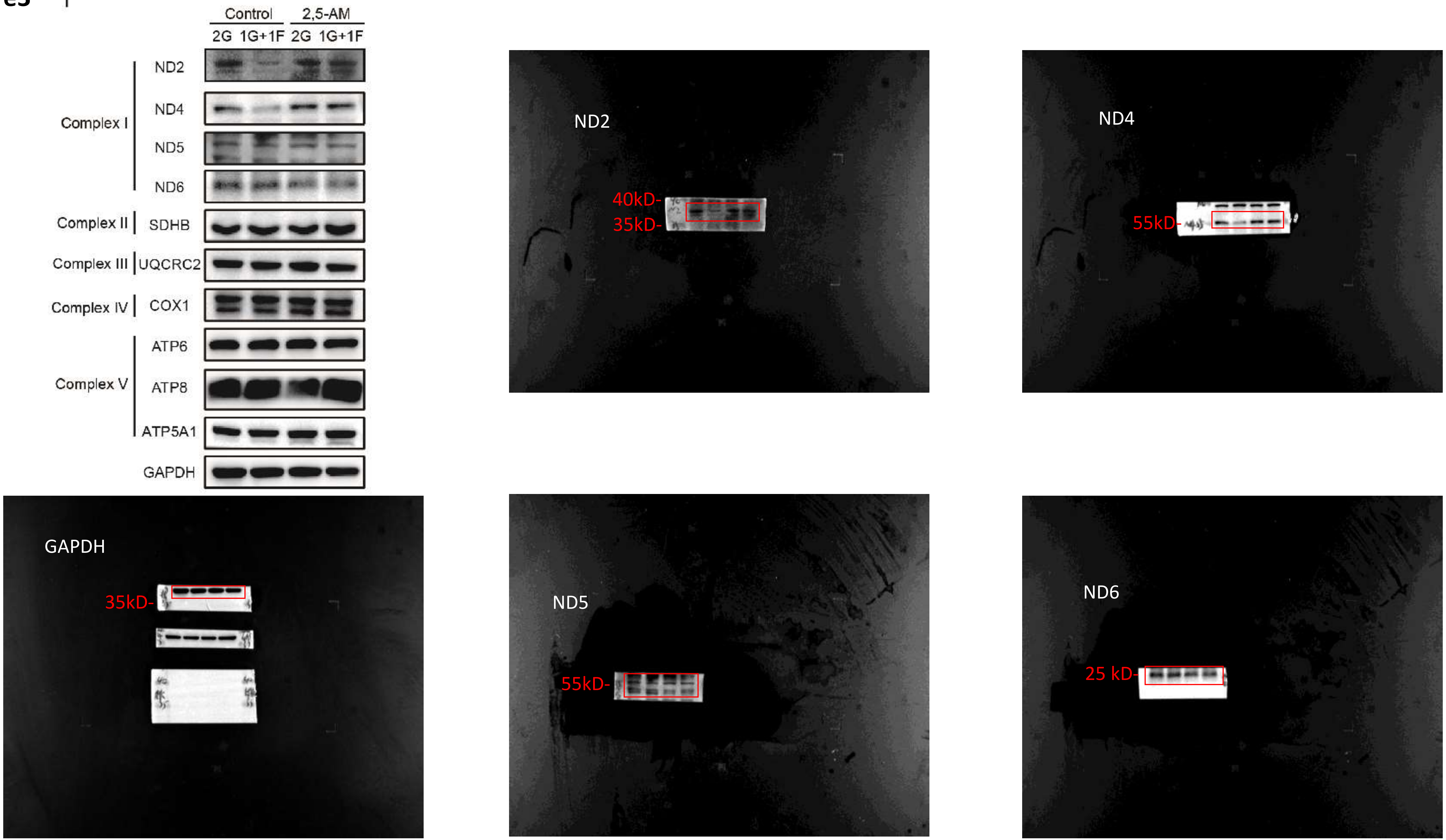

Control
2,5-AM
2G
1G+1F
2G
1G+1F
Complex I
ND2
ND4
ND5
ND6
Complex II
SDHB
Complex III
UQCRC2
Complex IV
COX1
Complex V
ATP6
ATP8
ATP5A1
GAPDH
ND2
40kD-
35kD-
ND4
55kD-
GAPDH
35kD-
ND5
55kD-
ND6
25 kD-

Figure3

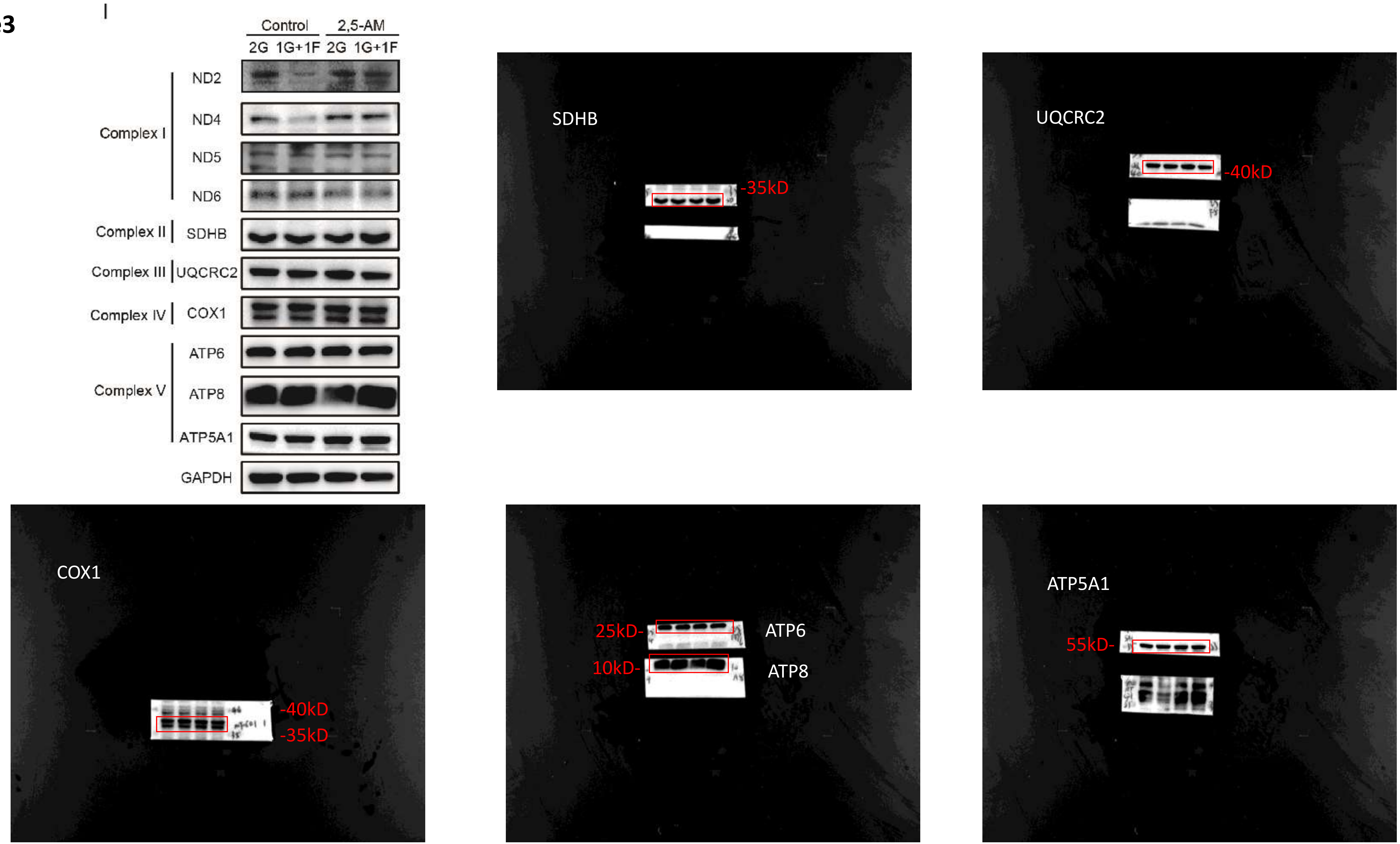

I
Control
2,5-AM
2G 1G+1F 2G 1G+1F
Complex I
ND2
ND4
ND5
ND6
Complex II
SDHB
Complex III
UQCRC2
Complex IV
COX1
Complex V
ATP6
ATP8
ATP5A1
GAPDH
SDHB
-35kD
UQCRC2
-40kD
COX1
-40kD
-35kD
25kD-
ATP6
10kD-
ATP8
ATP5A1
55kD-

Figure4

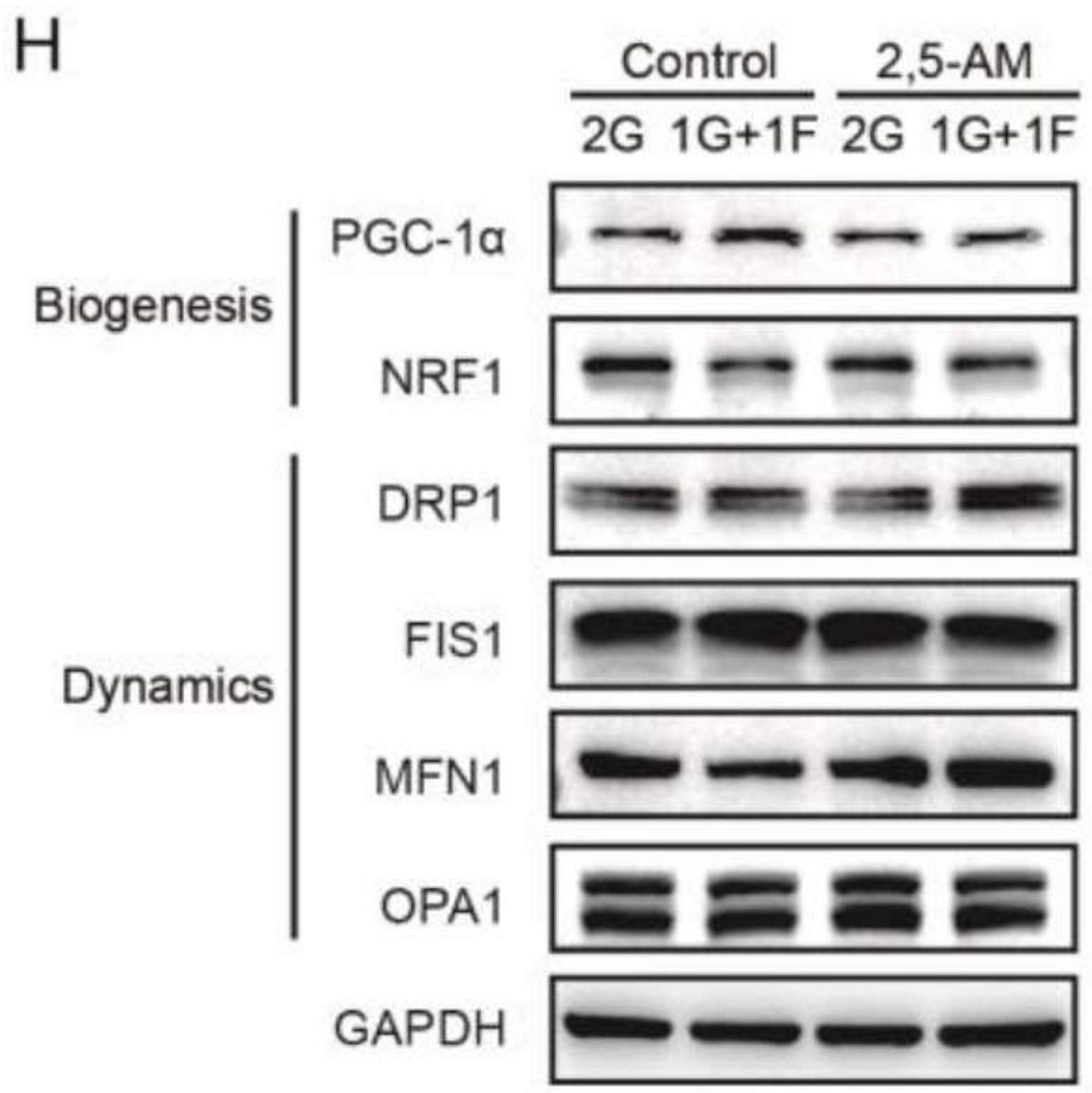
H
Control
2,5-AM
2G
1G+1F
2G
1G+1F
PGC-1α
Biogenesis
NRF1
DRP1
FIS1
Dynamics
MFN1
OPA1
GAPDH

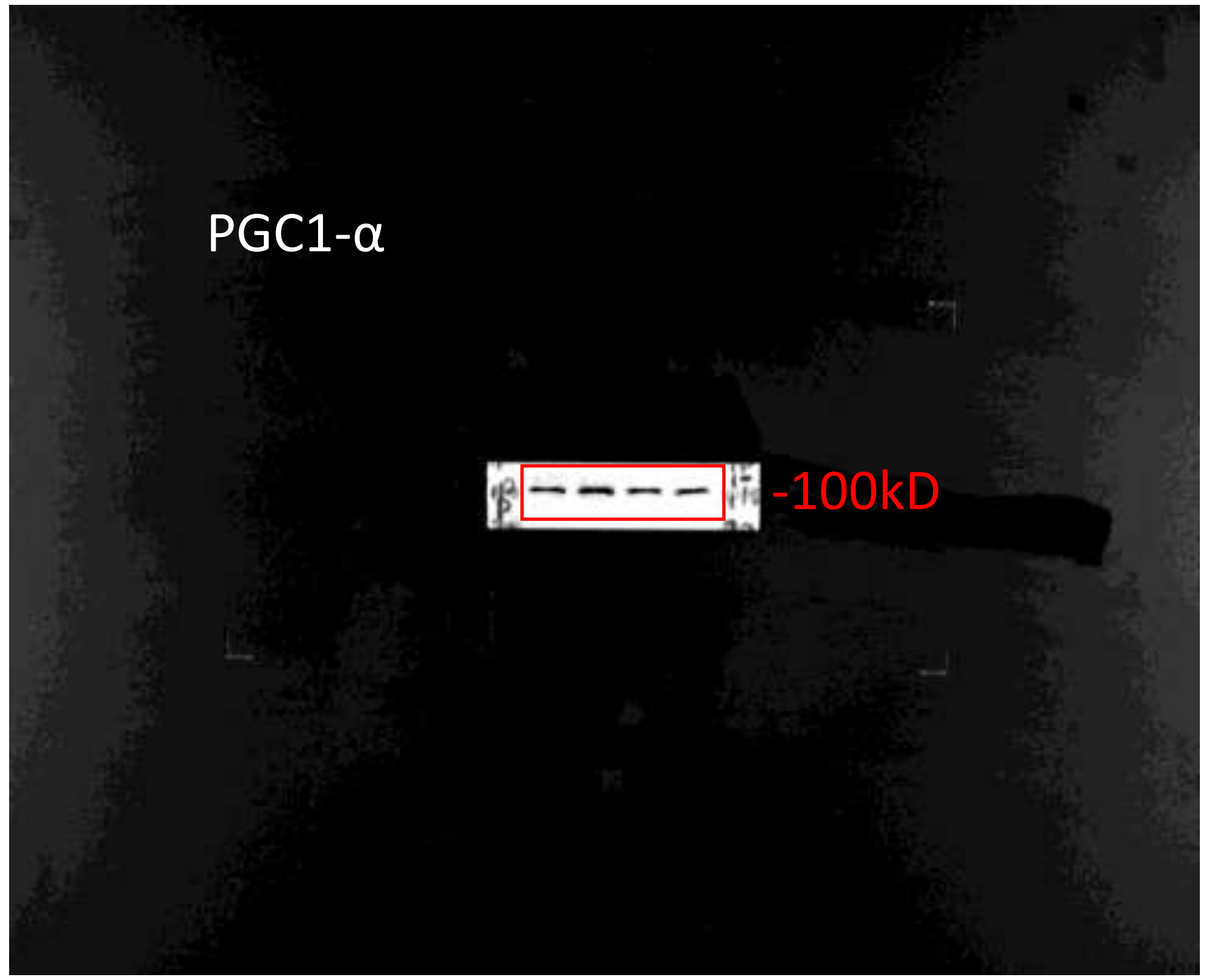
PGC1-α
-100kD

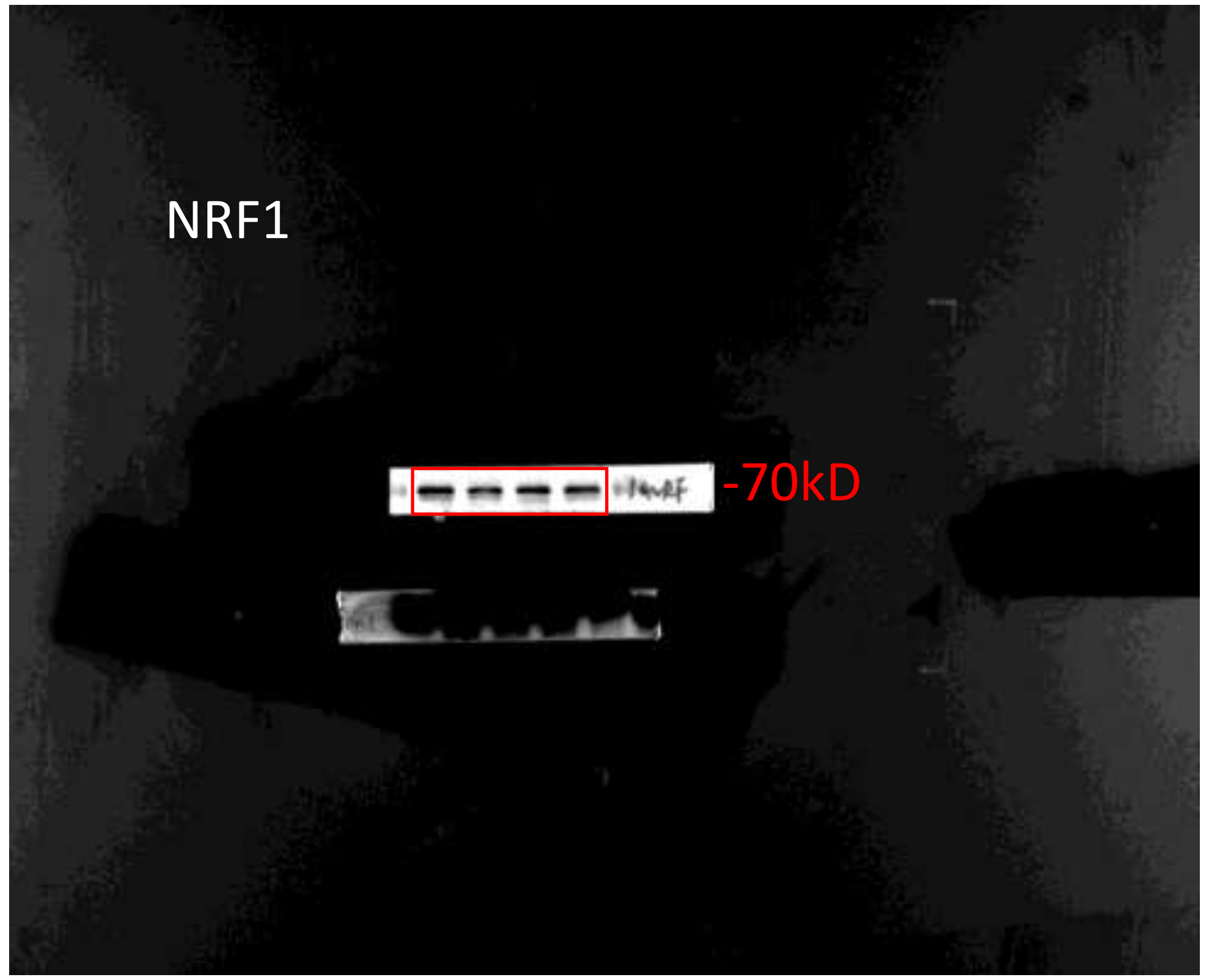
NRF1
-70kD

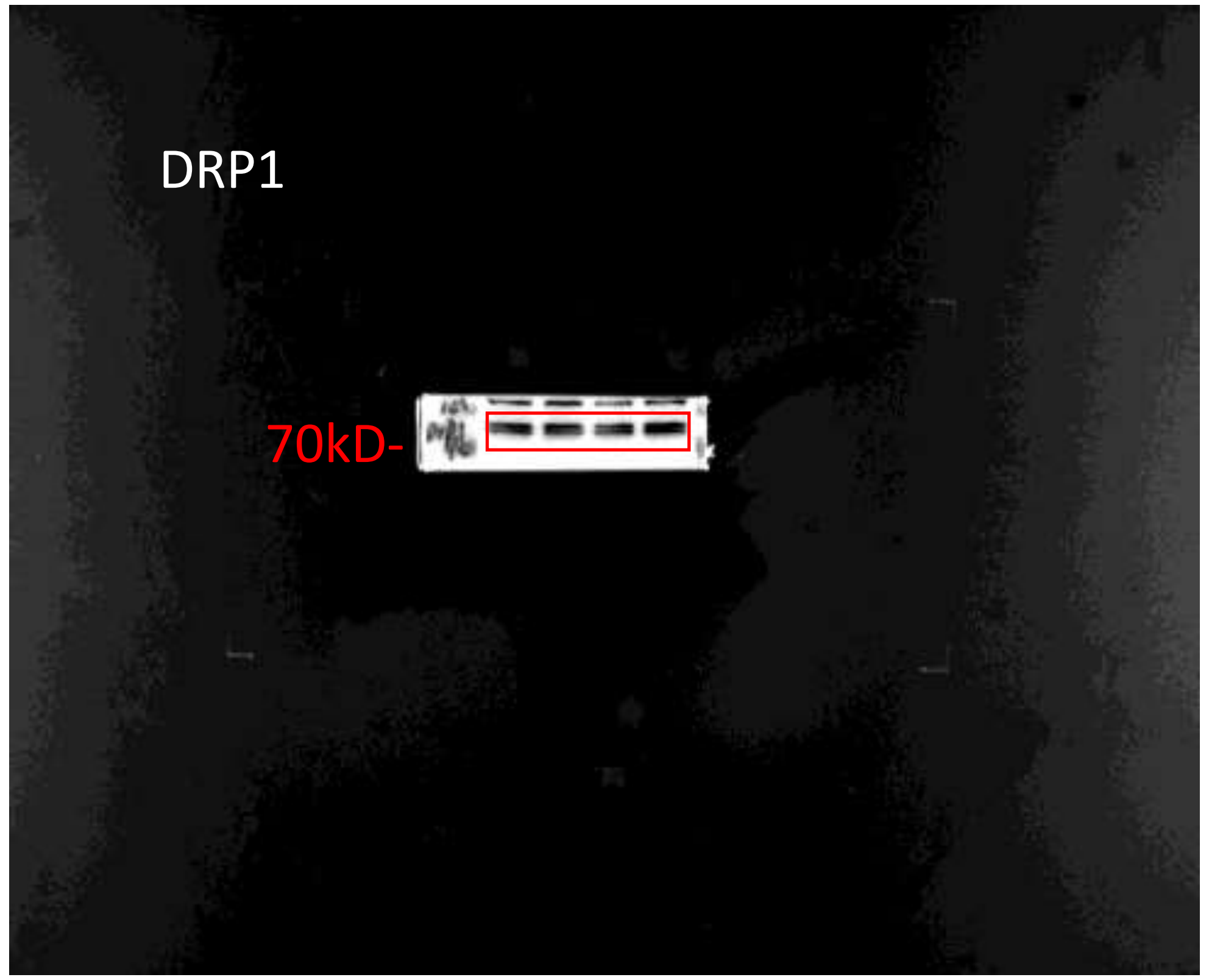
DRP1
70kD-

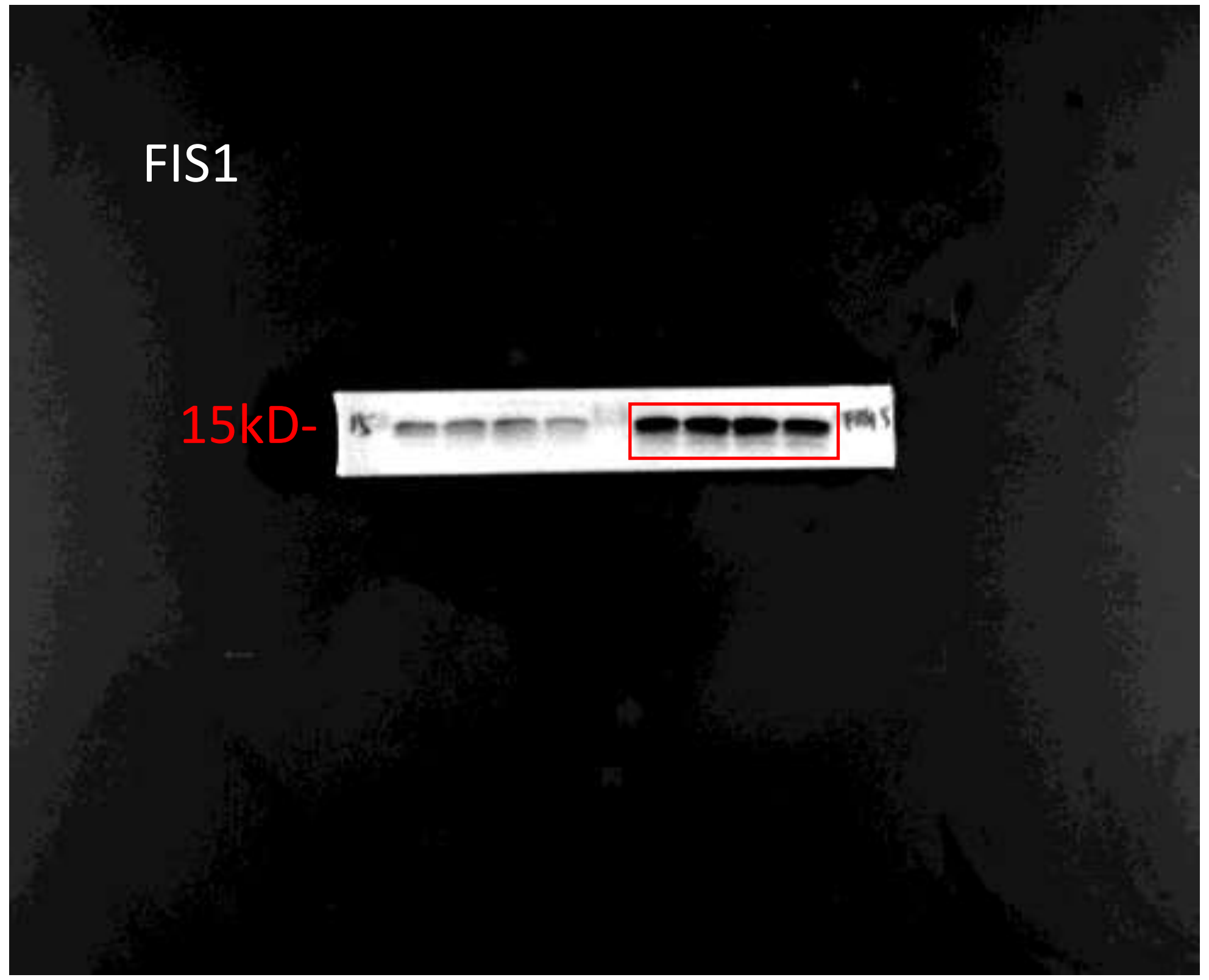
FIS1
15kD-

Figure4

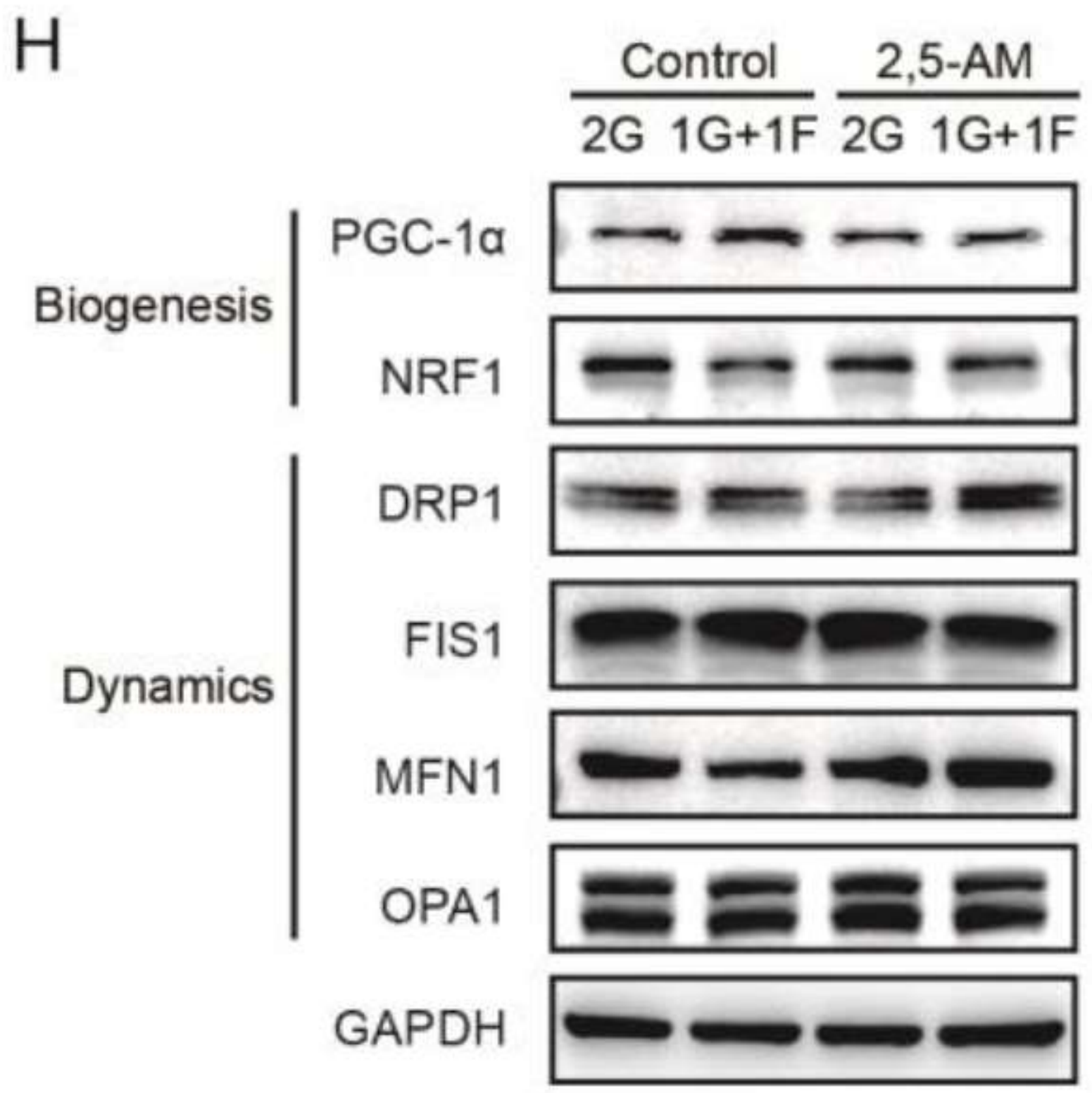
H
Control
2,5-AM
2G
1G+1F
2G
1G+1F
Biogenesis
PGC-1α
NRF1
Dynamics
DRP1
FIS1
MFN1
OPA1
GAPDH

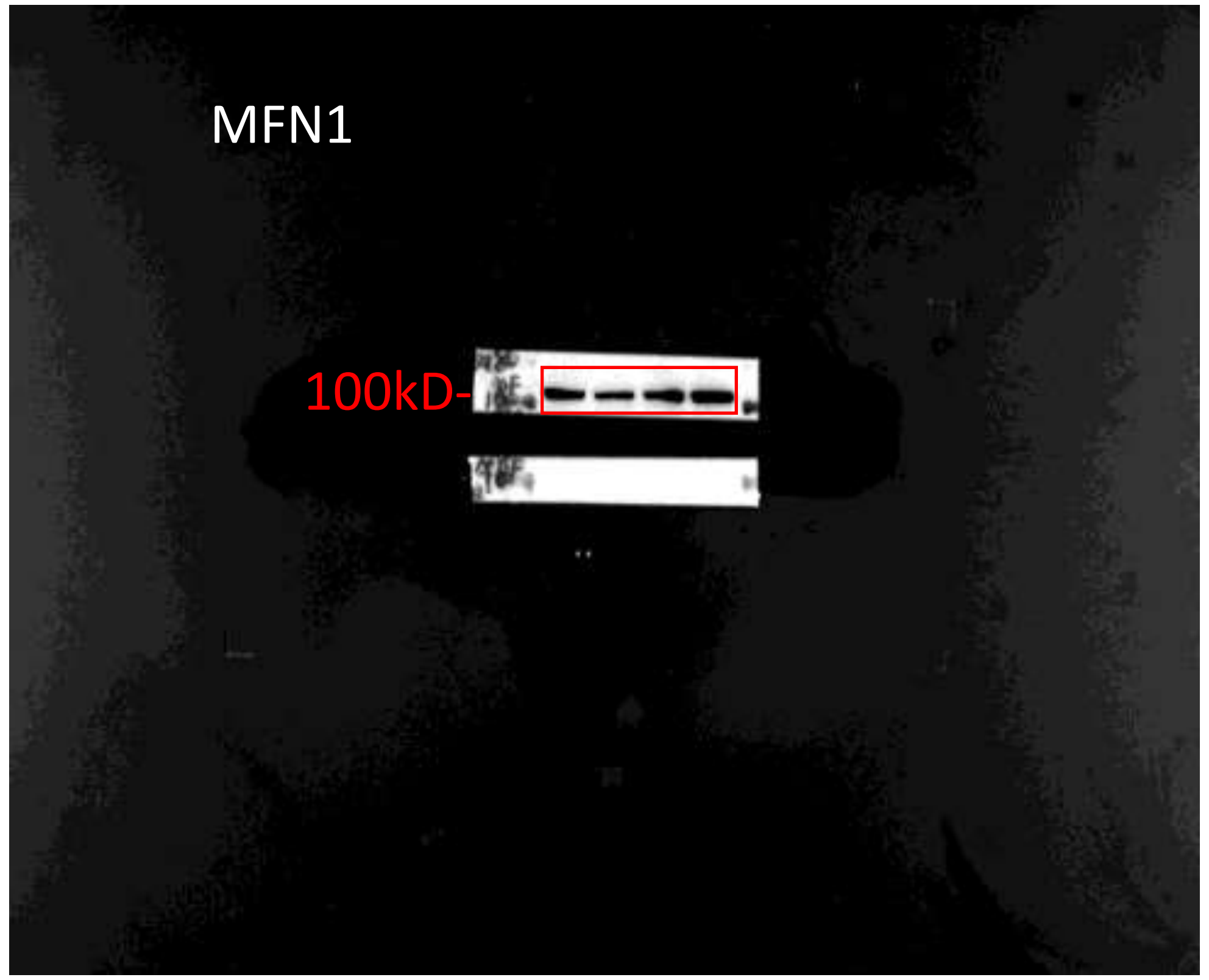
MFN1
100kD-

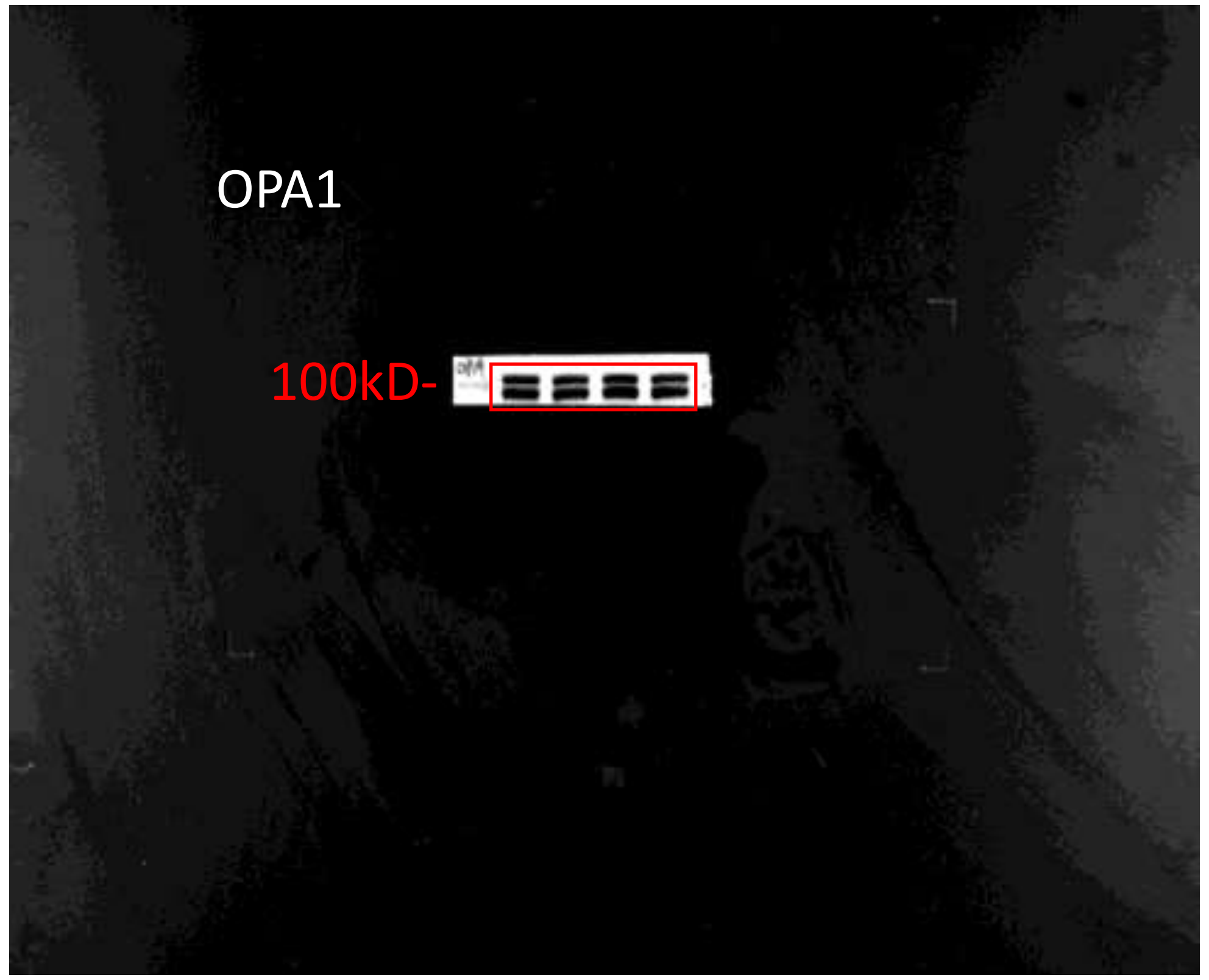
OPA1
100kD-

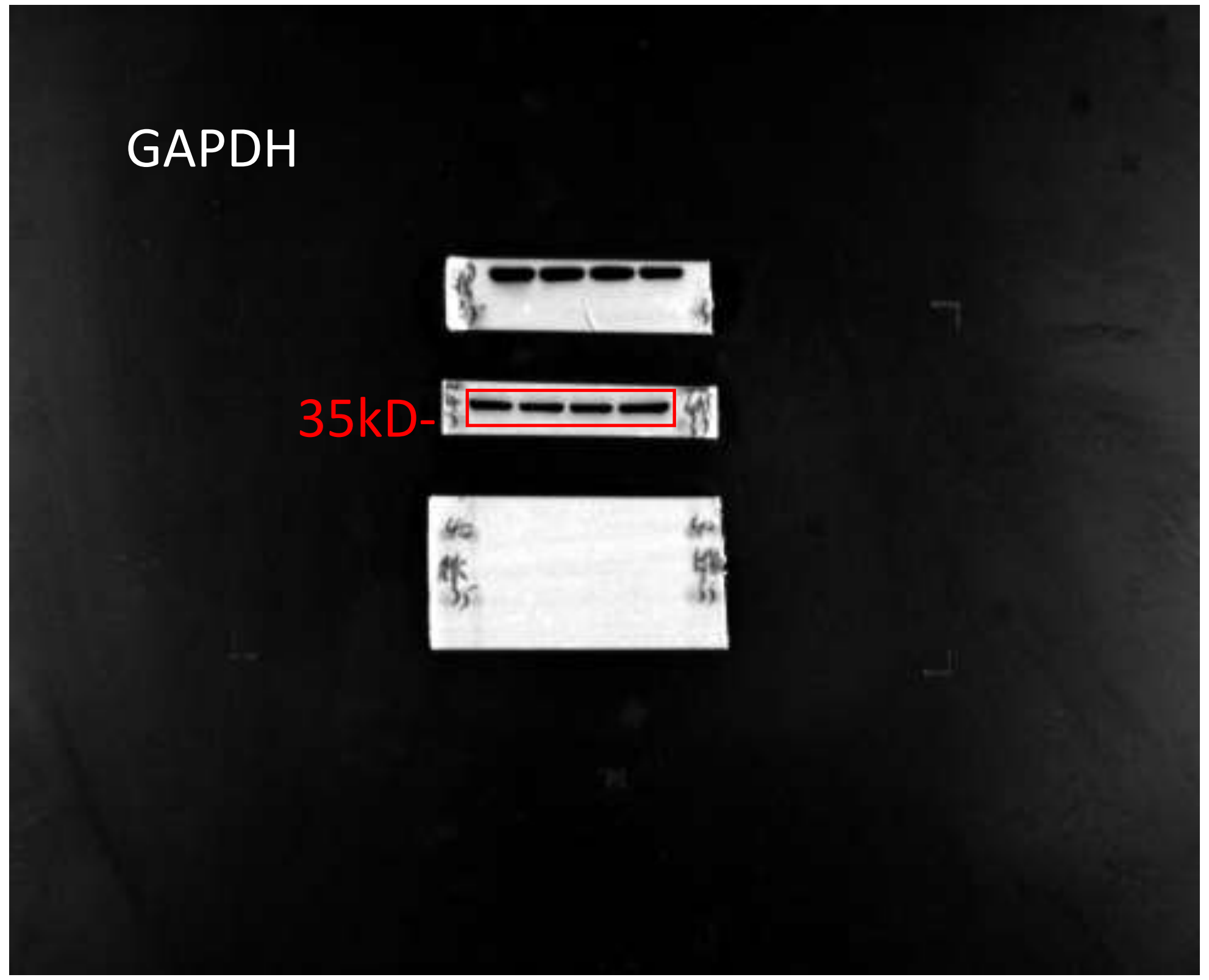
GAPDH
35kD-

Figure4

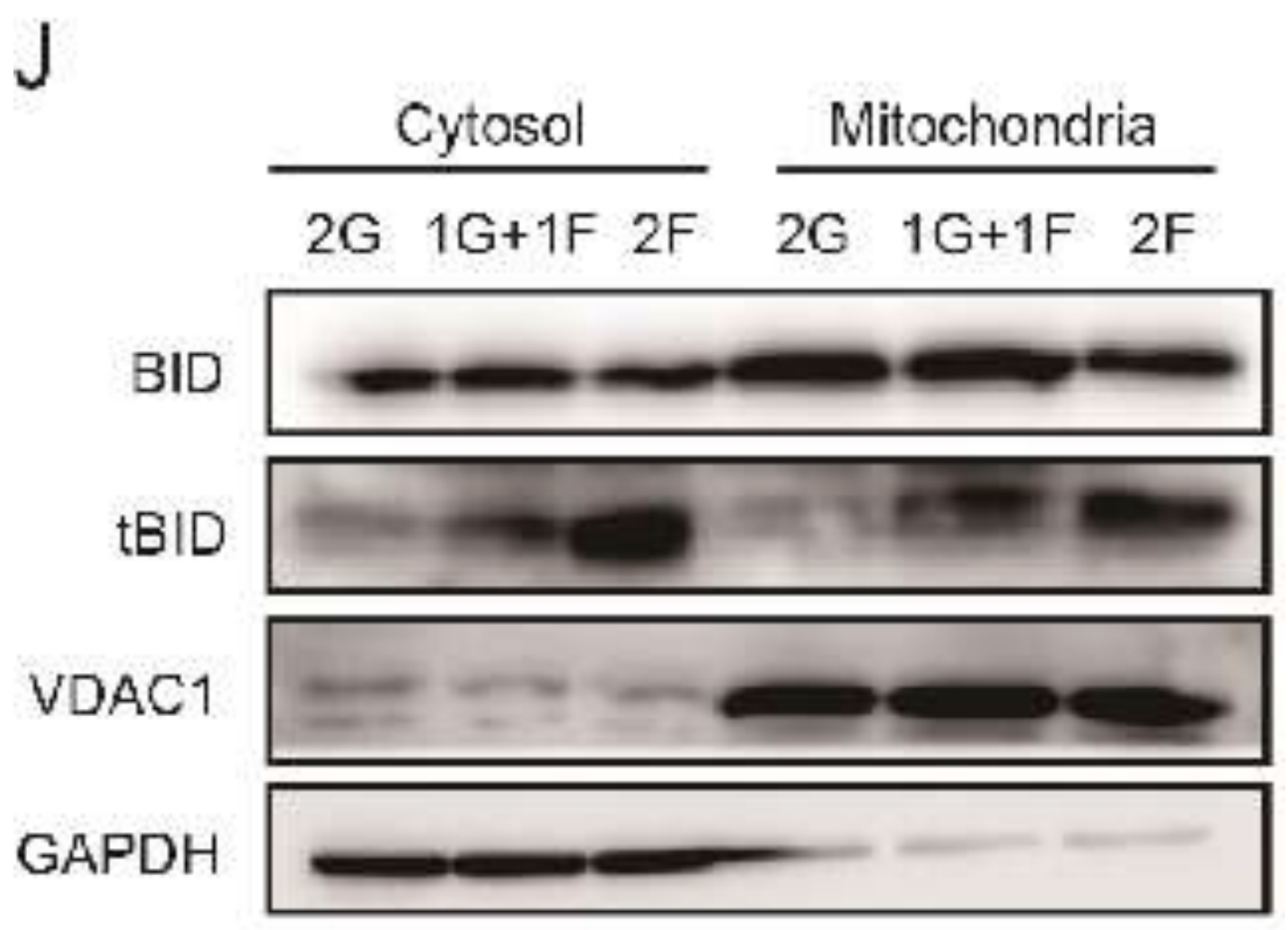
J
Cytosol
Mitochondria
2G 1G+1F 2F 2G 1G+1F 2F
BID
tBID
VDAC1
GAPDH

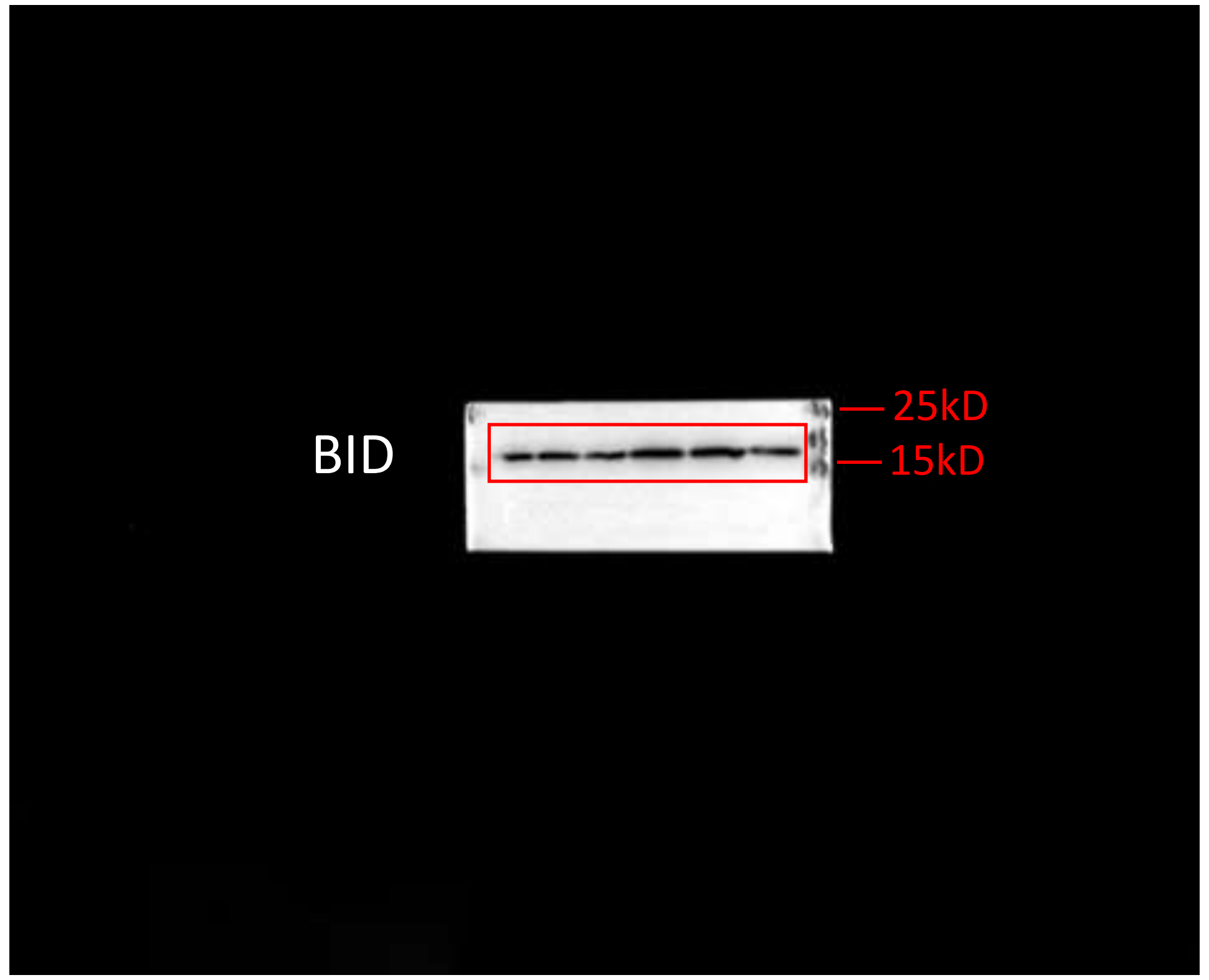
BID
25kD
15kD

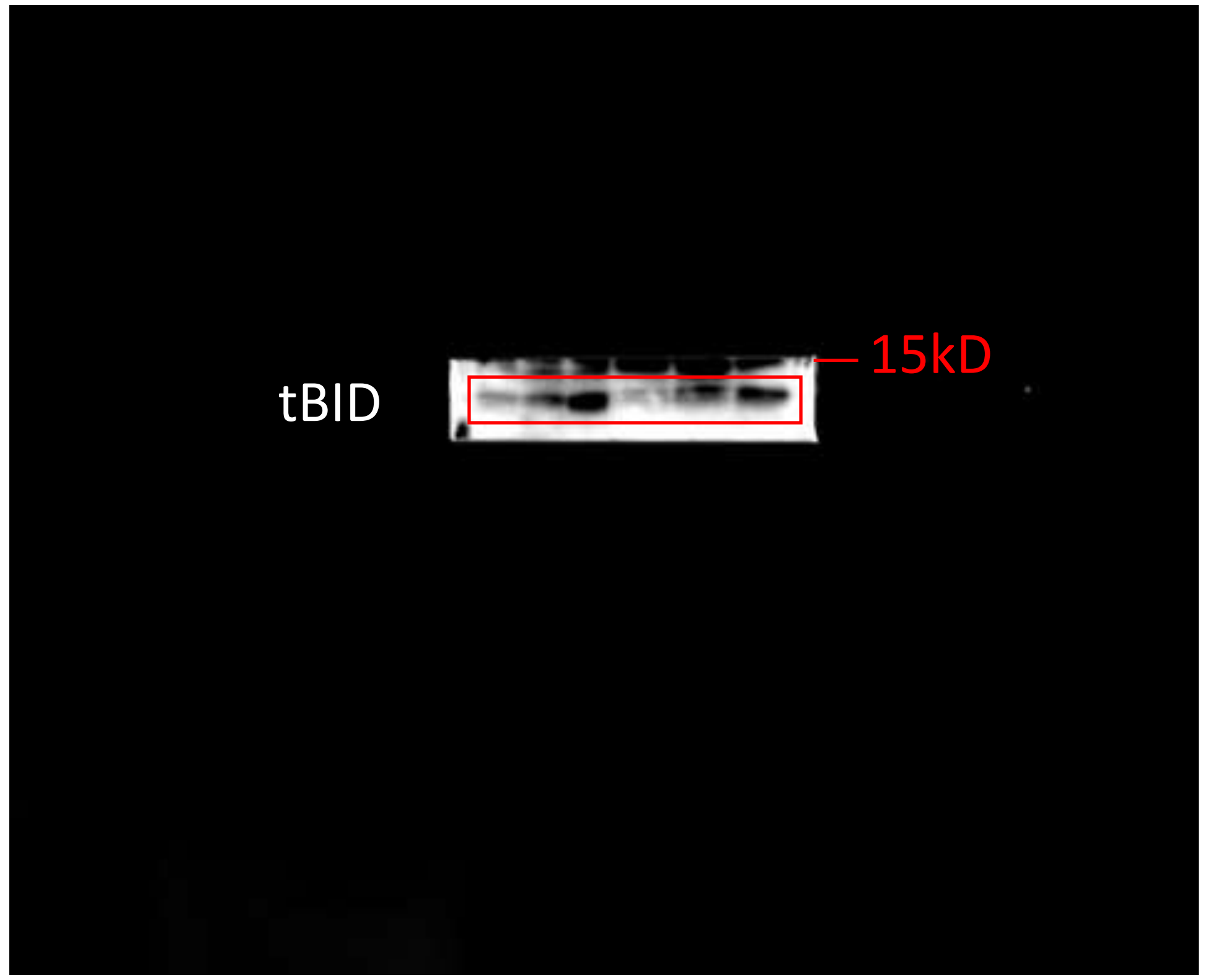
tBID
15kD

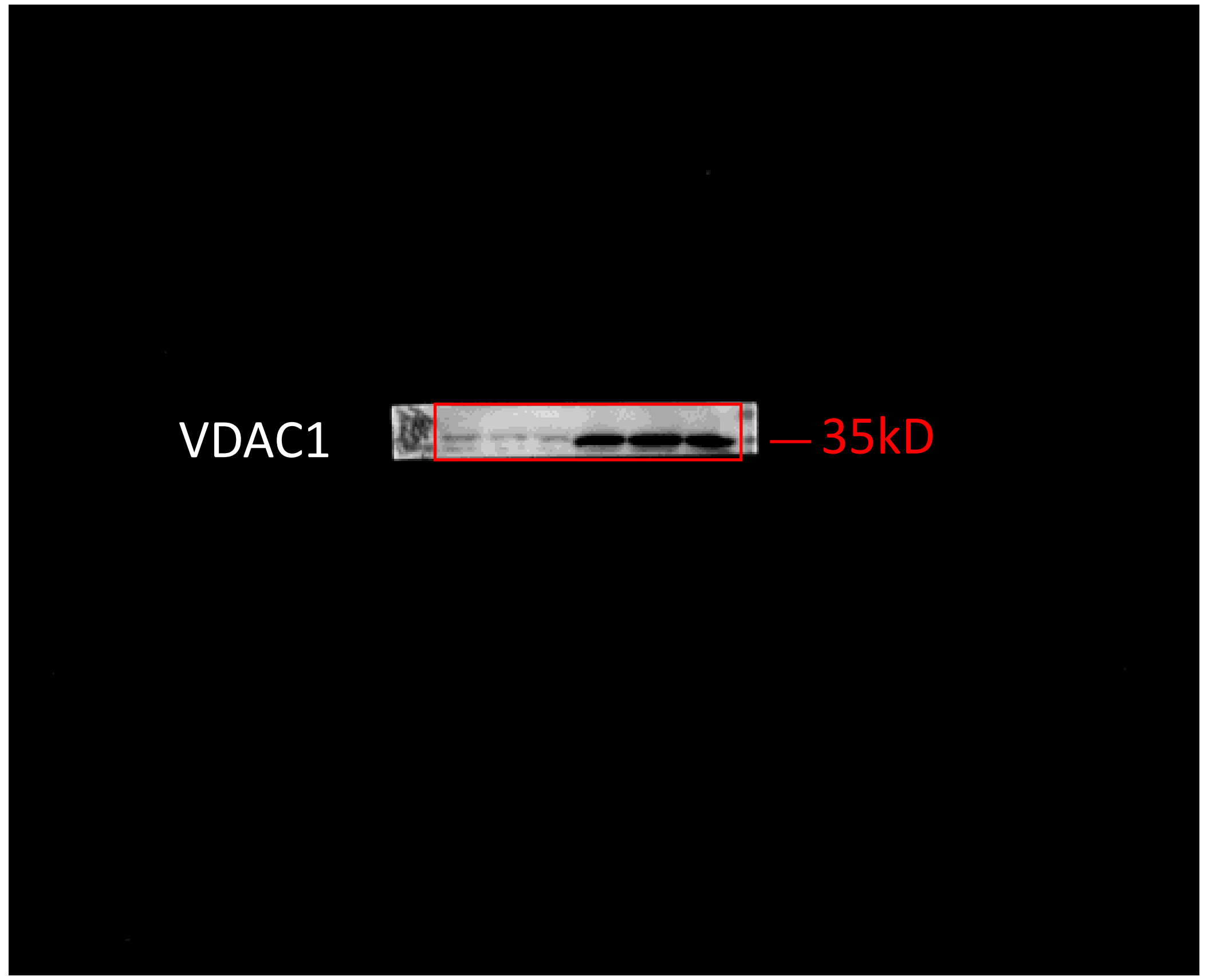
VDAC1
35kD

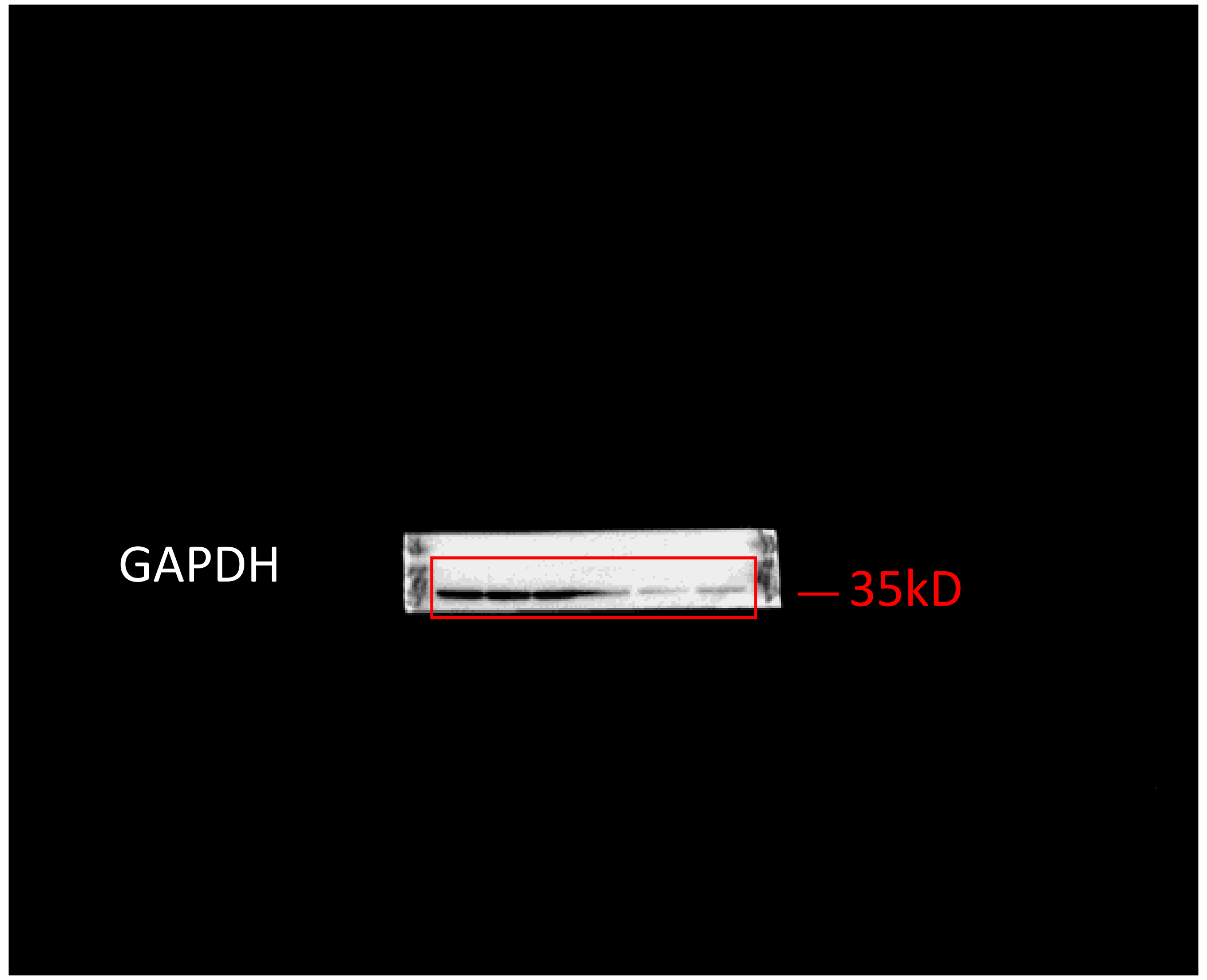
GAPDH
35kD

Figure4

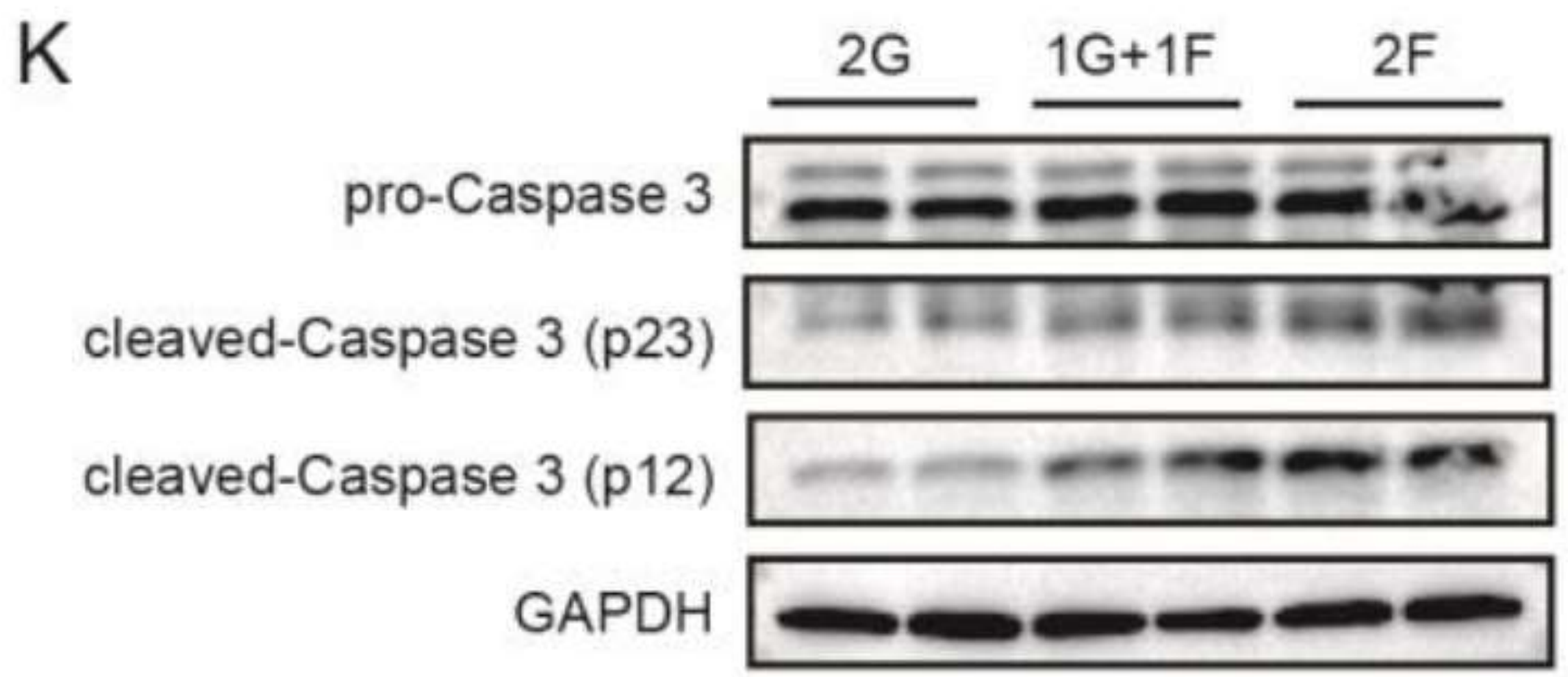
K
2G
1G+1F
2F
pro-Caspase 3
cleaved-Caspase 3 (p23)
cleaved-Caspase 3 (p12)
GAPDH

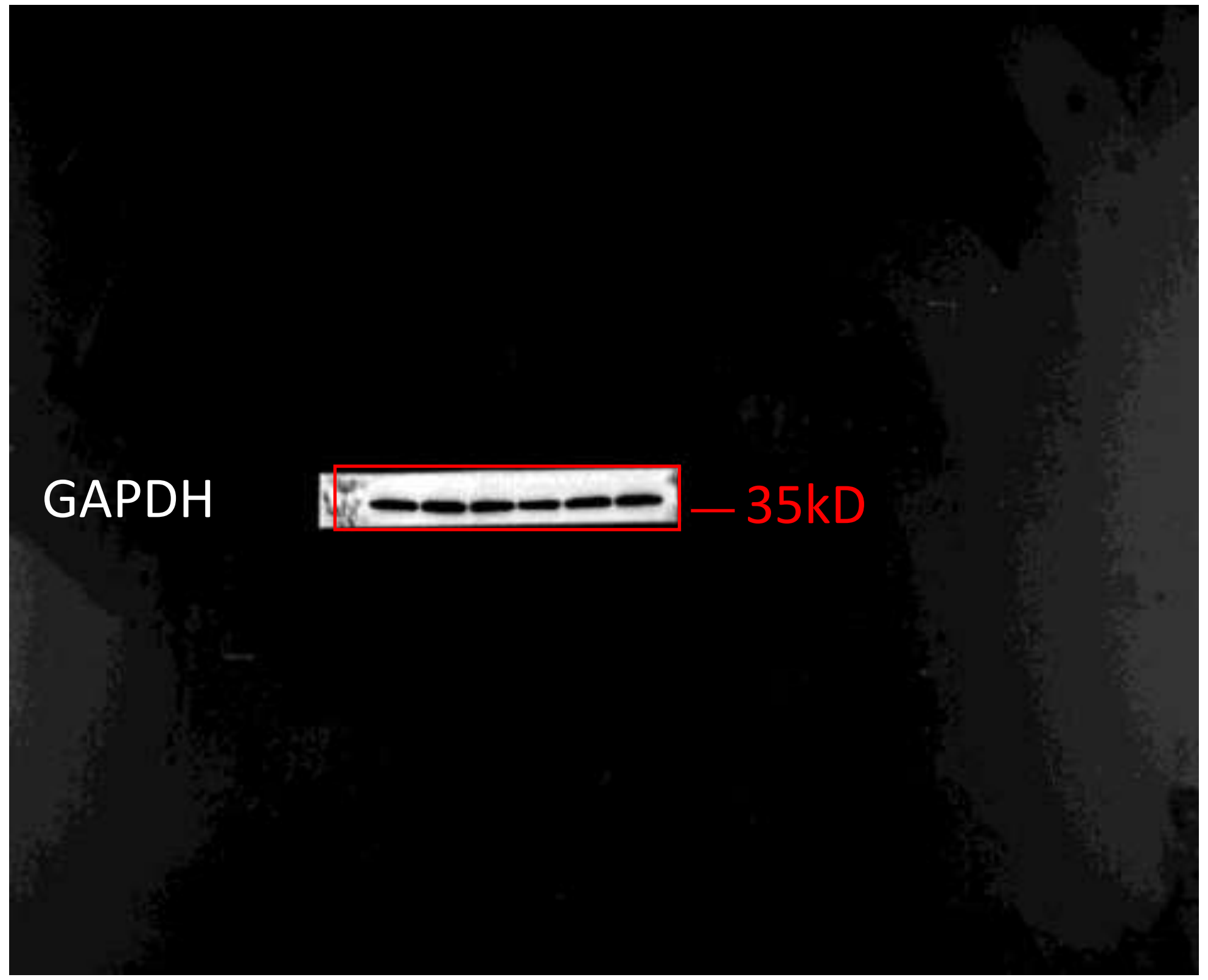
GAPDH
35kD

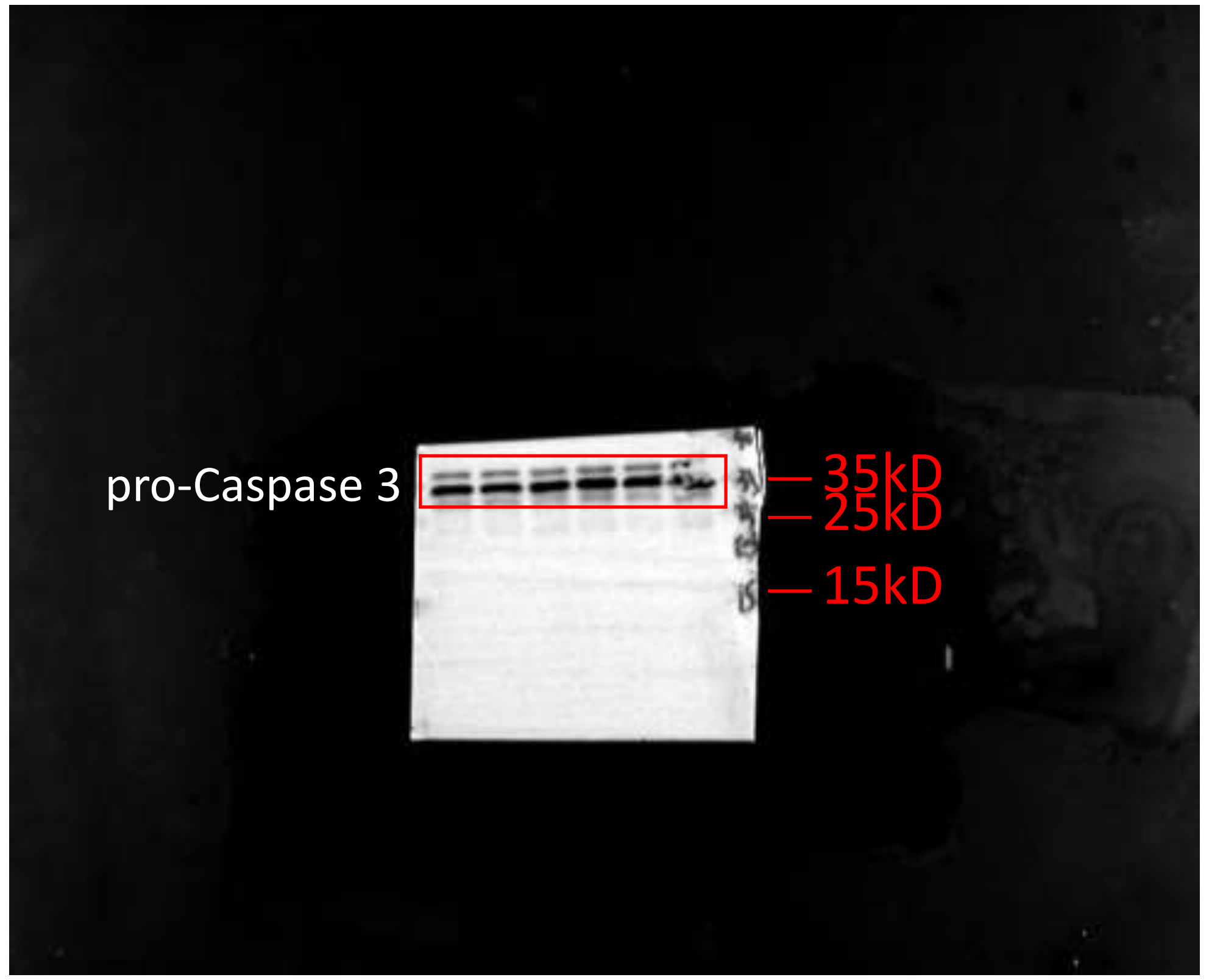
pro-Caspase 3
35kD
25kD
15kD

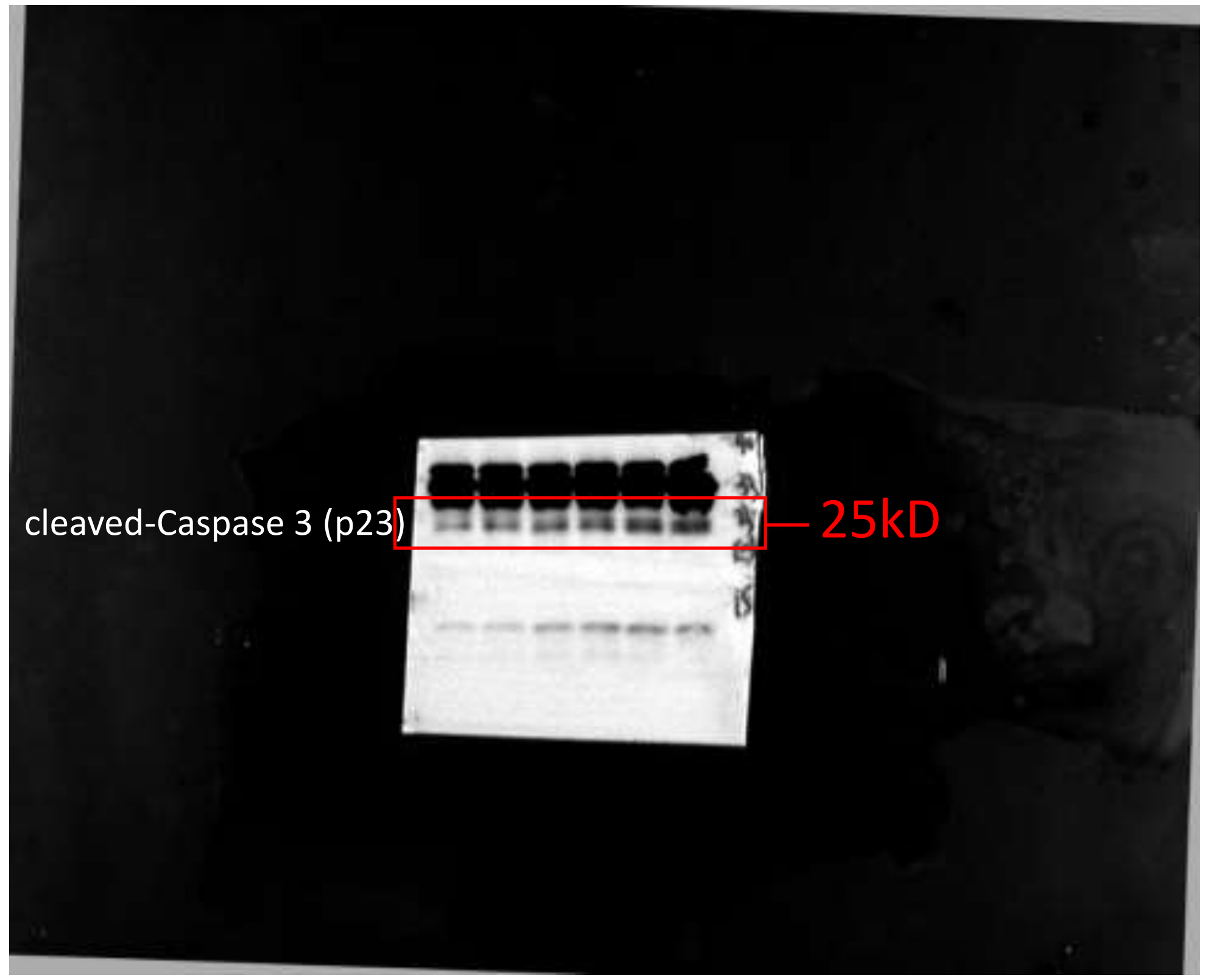
cleaved-Caspase 3 (p23)
25kD

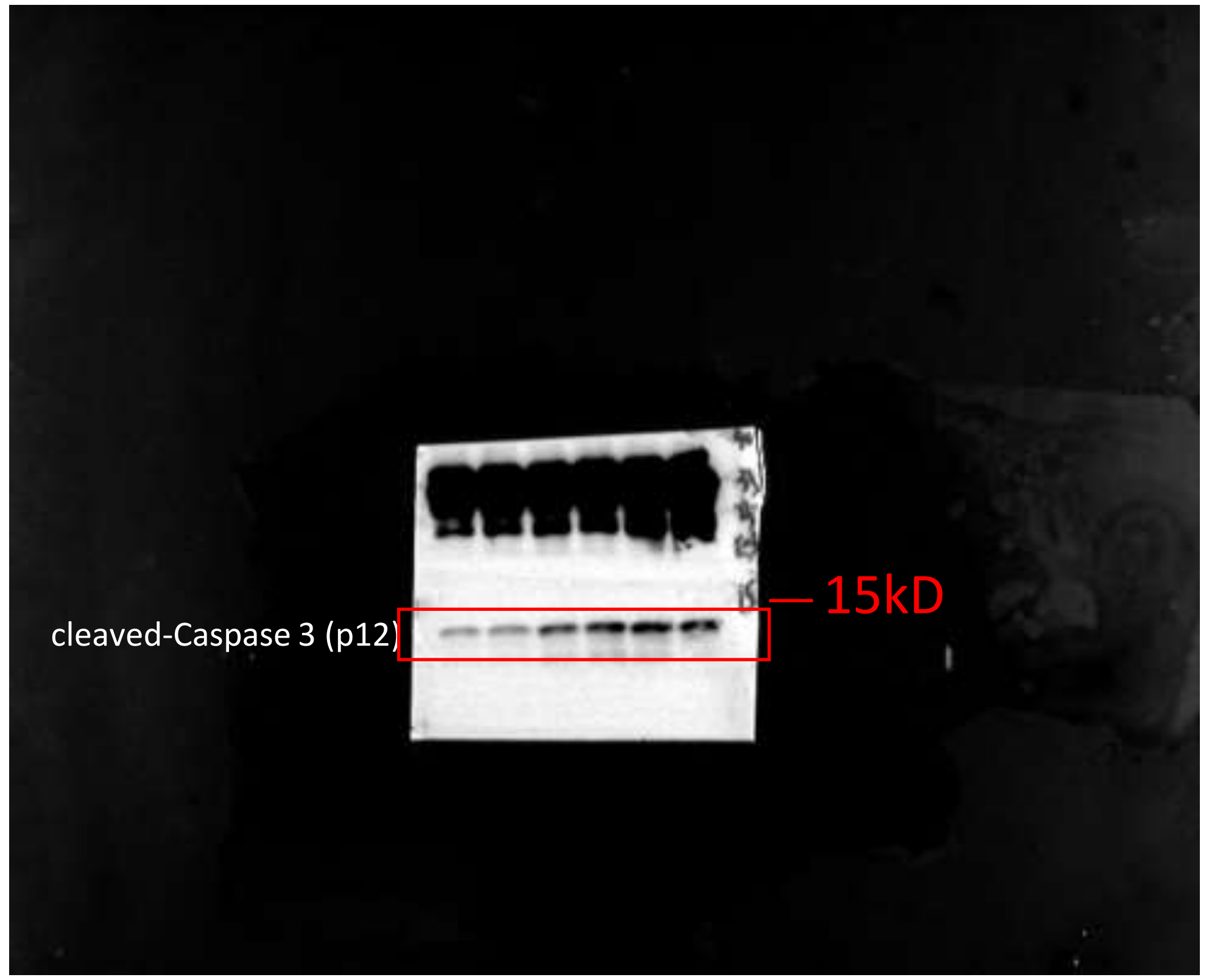
15kD
cleaved-Caspase 3 (p12)

Figure5

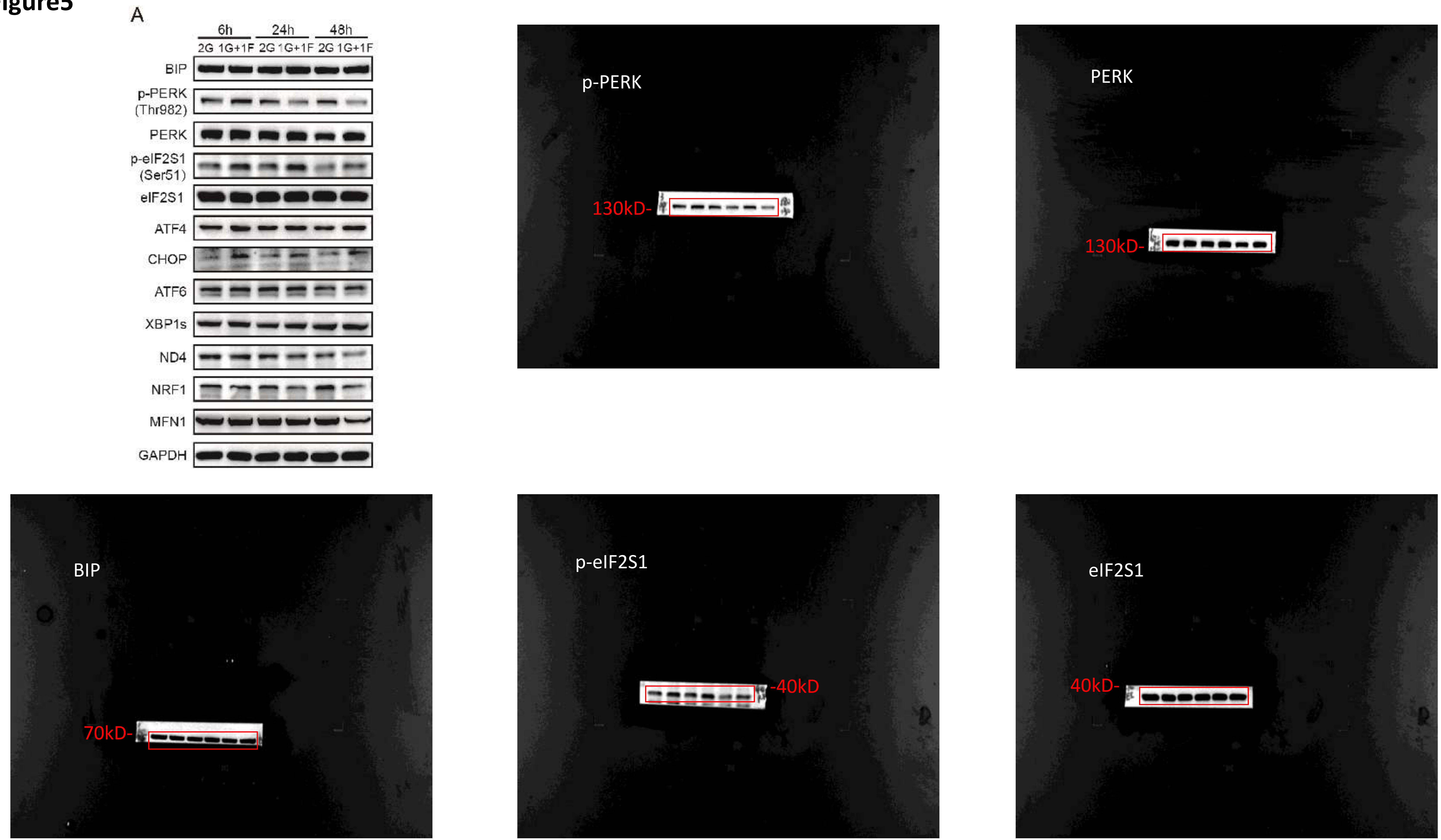
A
6h
24h
48h
2G 1G+1F 2G 1G+1F 2G 1G+1F
BIP
p-PERK (Thr982)
PERK
p-eIF2S1 (Ser51)
eIF2S1
ATF4
CHOP
ATF6
XBP1s
ND4
NRF1
MFN1
GAPDH
p-PERK
130kD-
PERK
130kD-
BIP
70kD-
p-eIF2S1
-40kD
eIF2S1
40kD-

Figure5

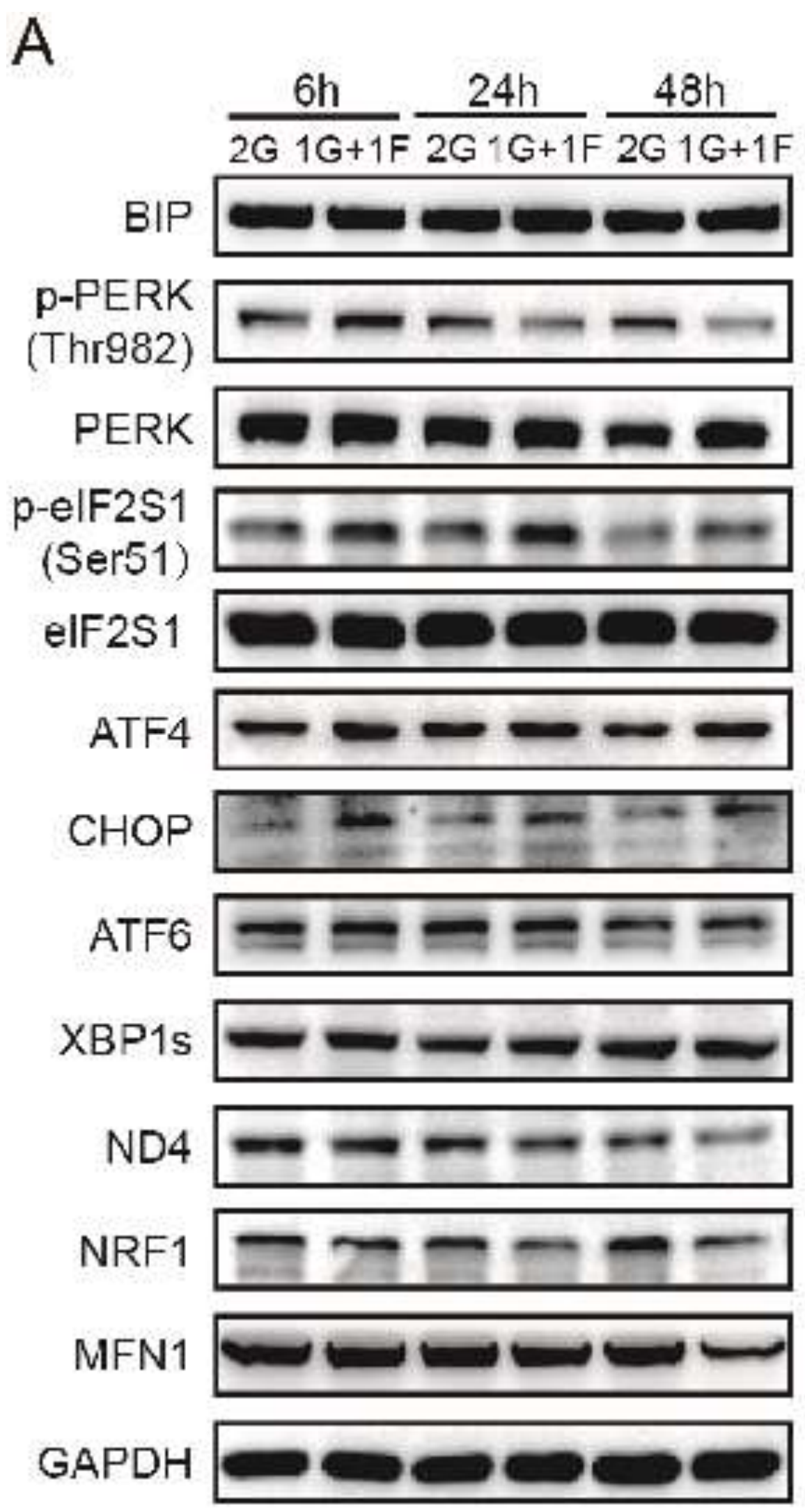
A
6h
24h
48h
2G 1G+1F 2G 1G+1F 2G 1G+1F
BIP
p-PERK (Thr982)
PERK
p-eIF2S1 (Ser51)
eIF2S1
ATF4
CHOP
ATF6
XBP1s
ND4
NRF1
MFN1
GAPDH

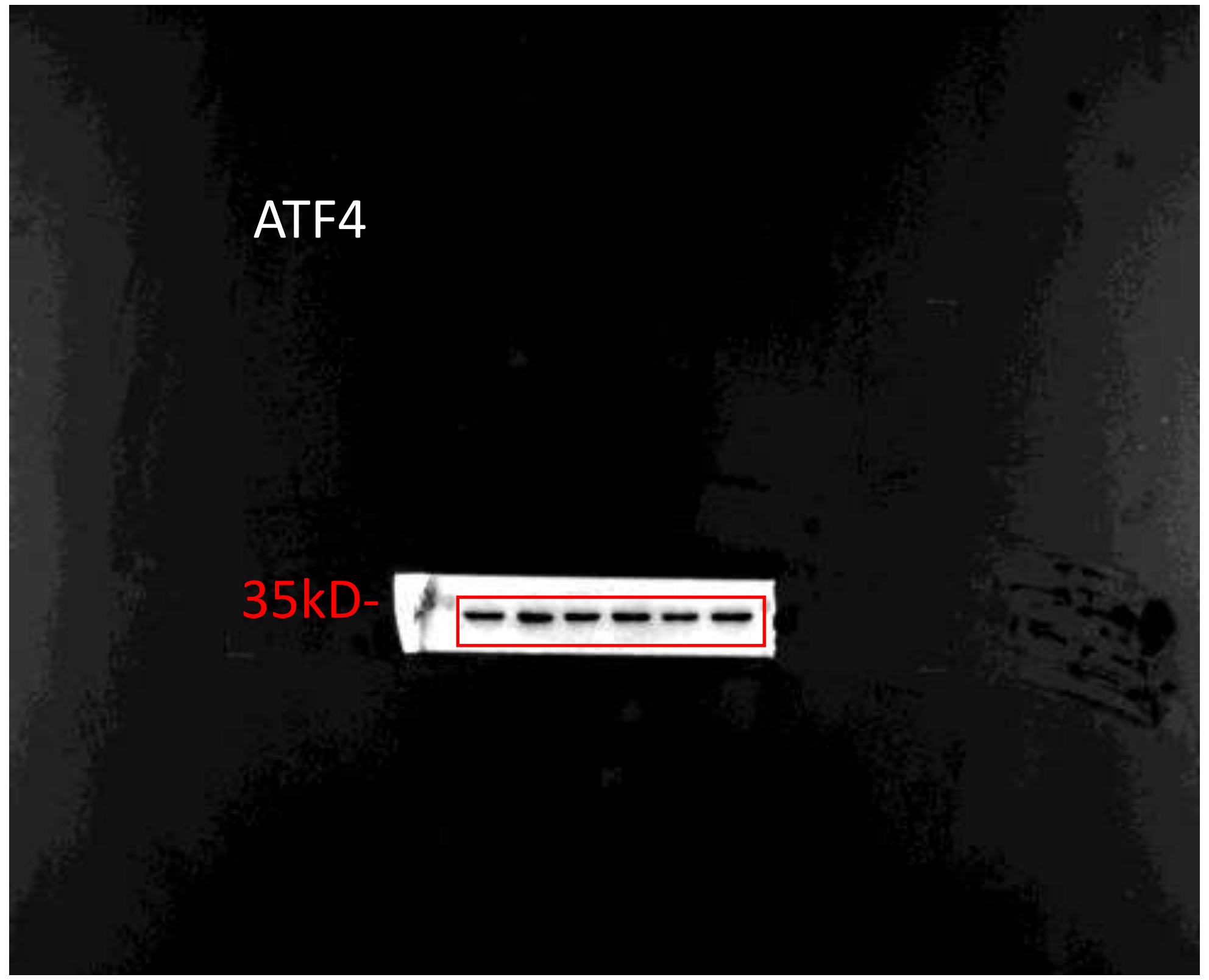
ATF4
35kD-

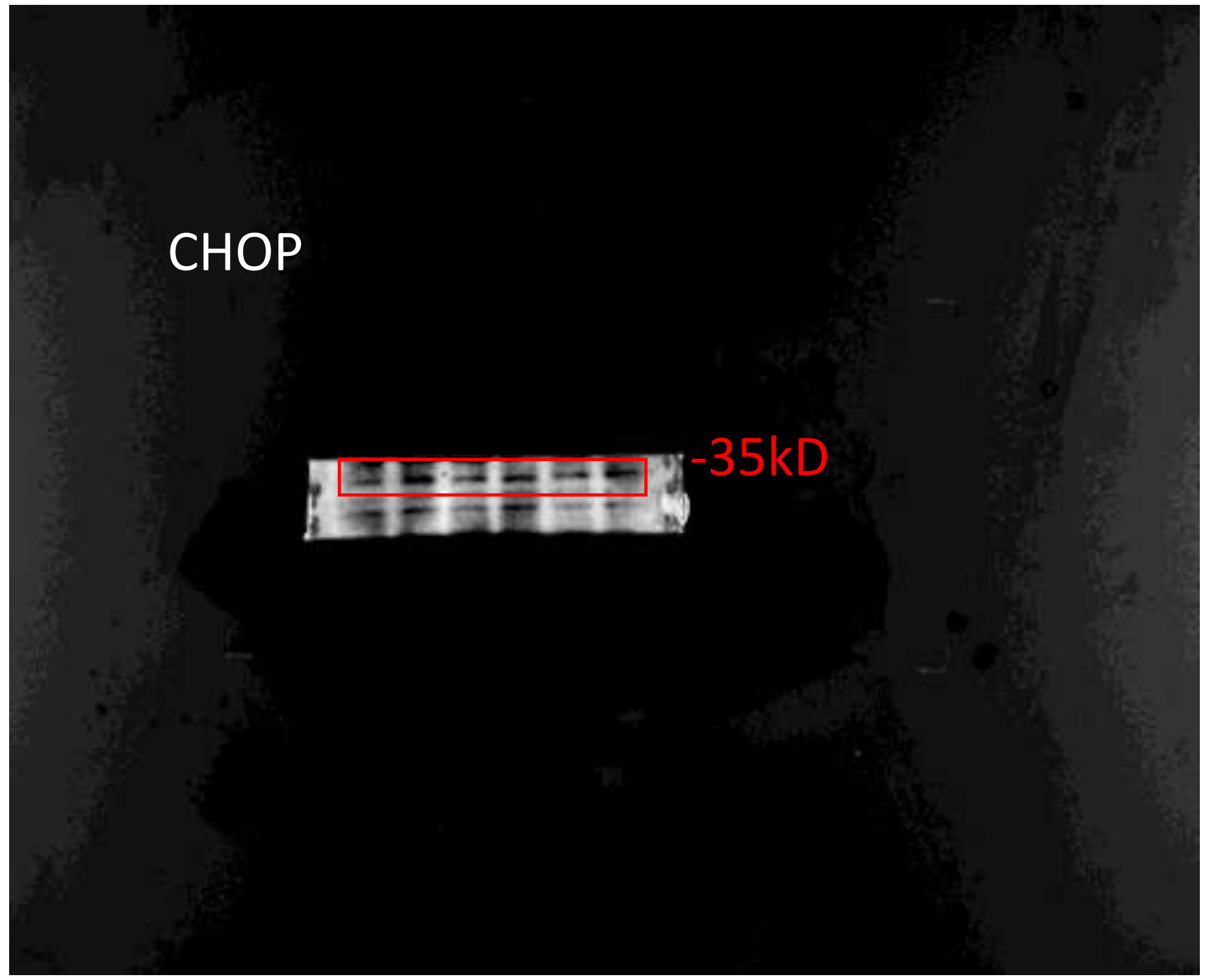
CHOP
-35kD

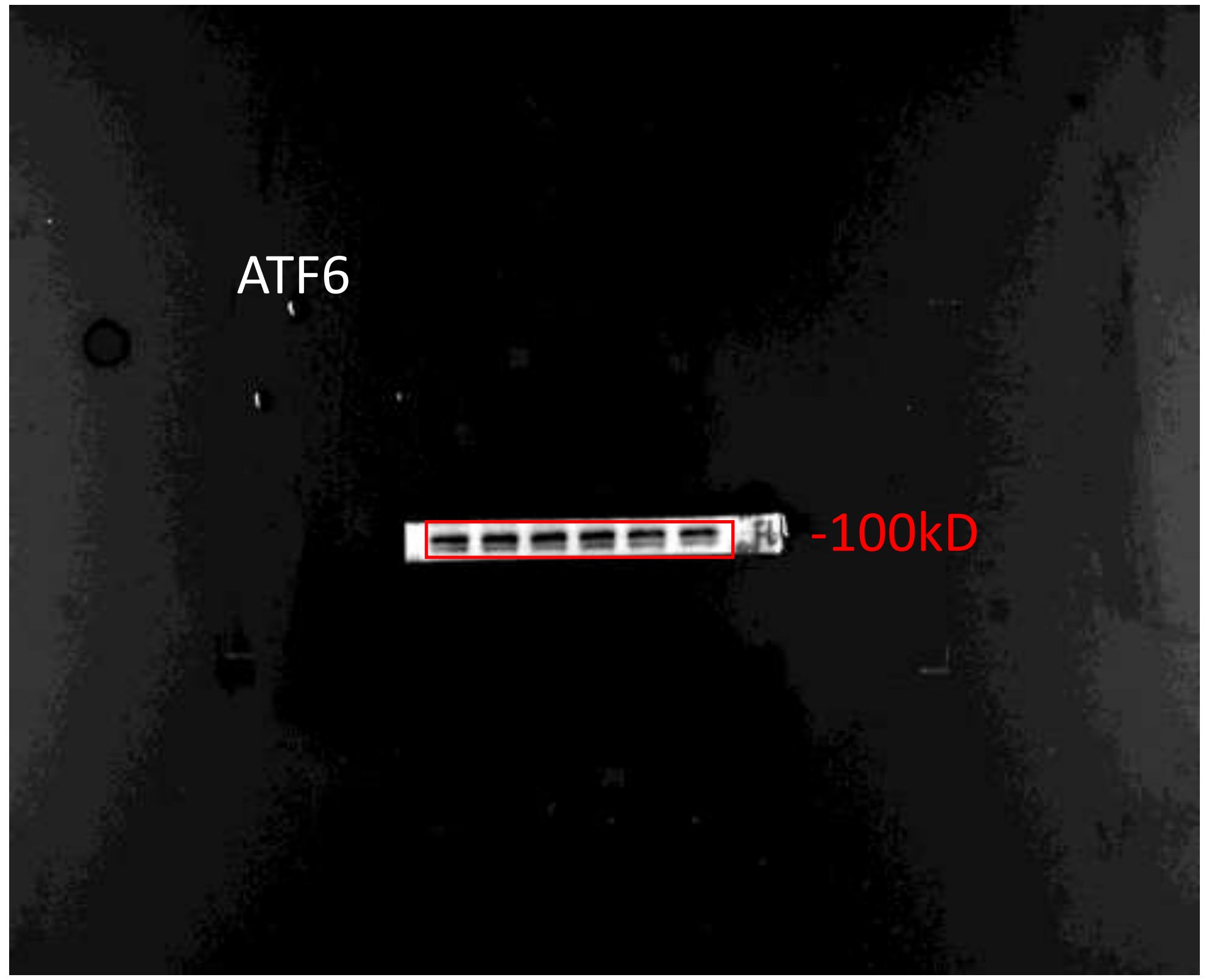
ATF6
-100kD

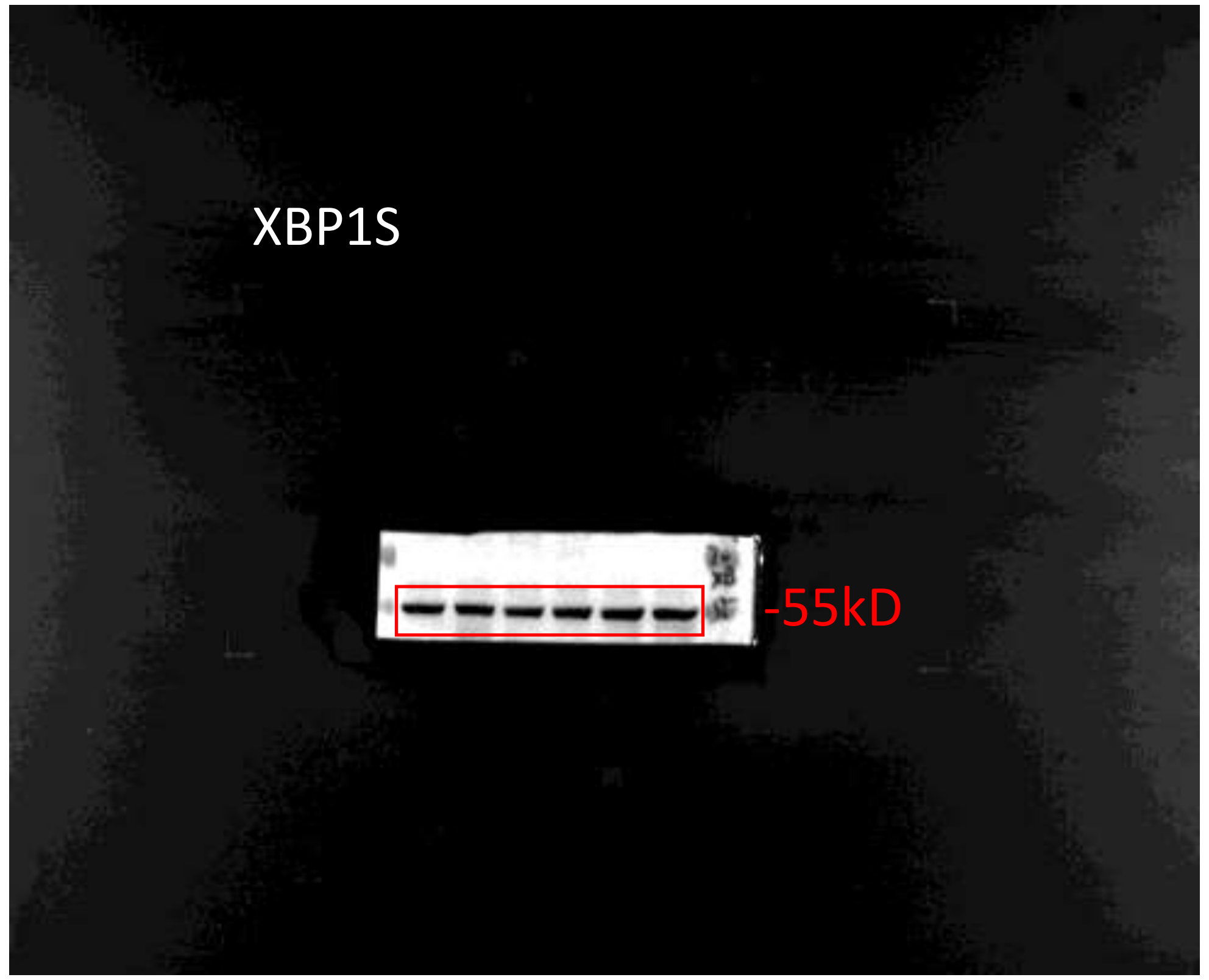
XBP1S
-55kD

Figure5

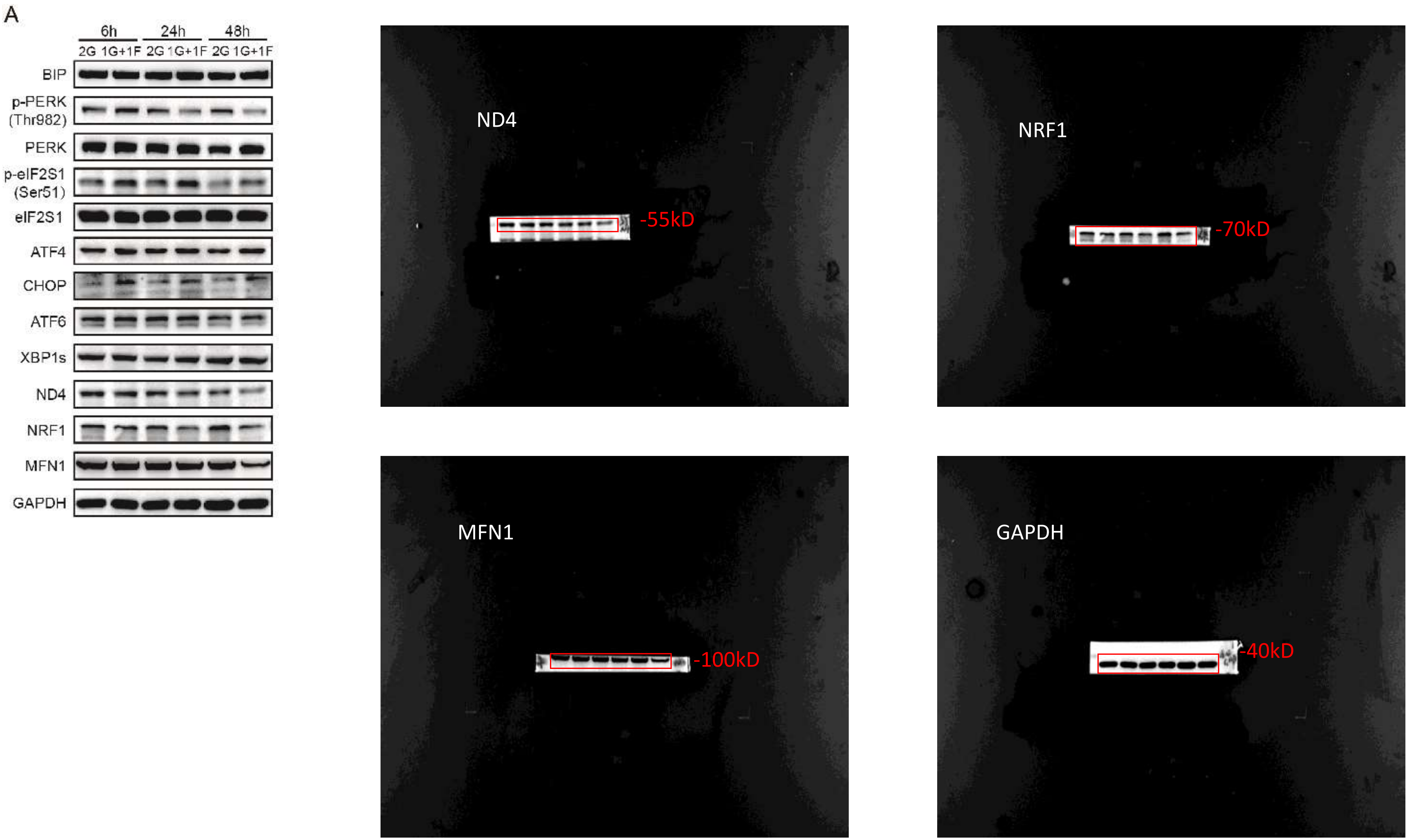
A
6h
24h
48h
2G 1G+1F 2G 1G+1F 2G 1G+1F
BIP
p-PERK (Thr982)
PERK
p-eIF2S1 (Ser51)
eIF2S1
ATF4
CHOP
ATF6
XBP1s
ND4
NRF1
MFN1
GAPDH
ND4
-55kD
NRF1
-70kD
MFN1
-100kD
GAPDH
-40kD

Figure5

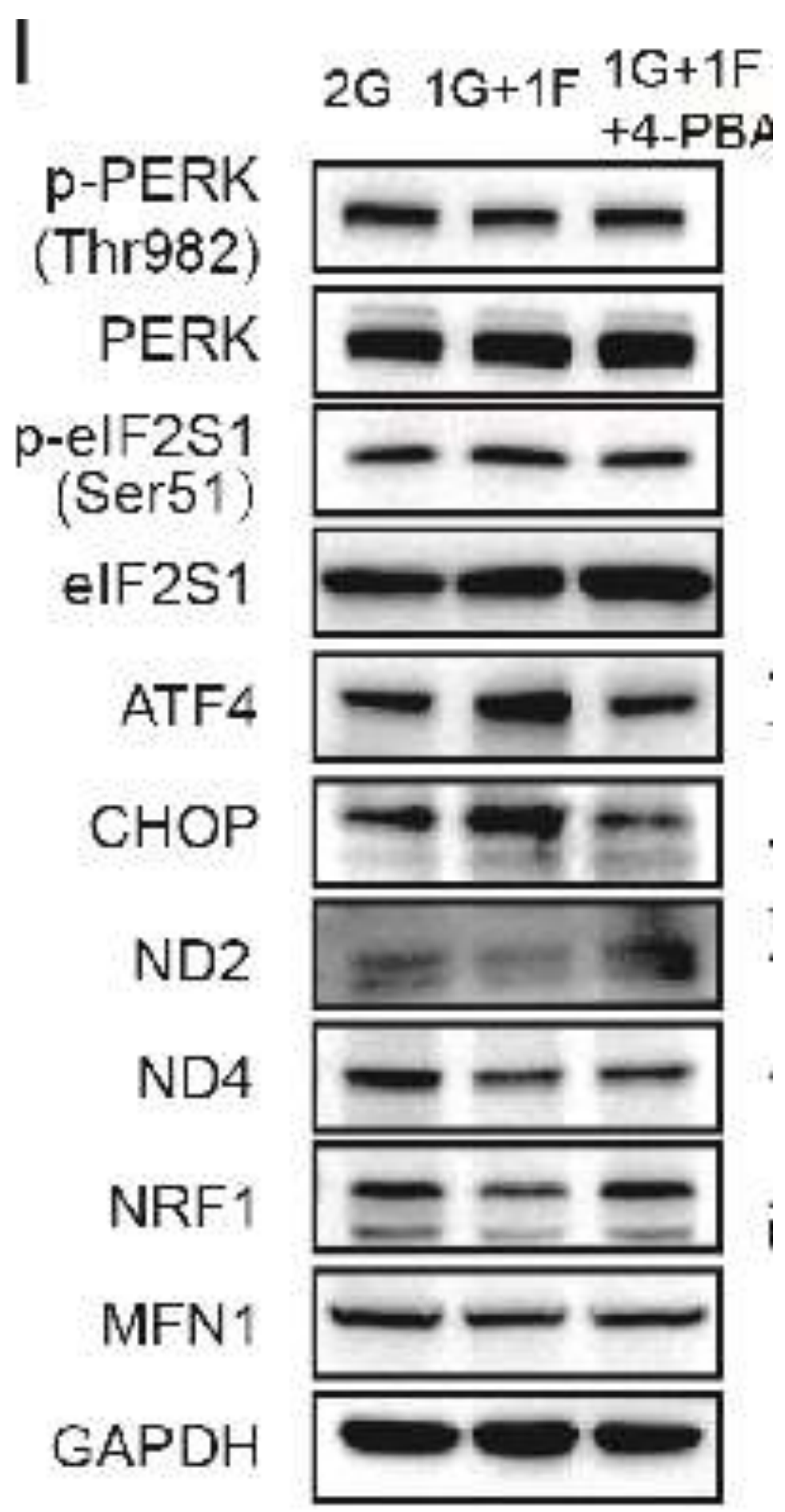
I
2G
1G+1F
1G+1F
+4-PBA
p-PERK
(Thr982)
PERK
p-eIF2S1
(Ser51)
eIF2S1
ATF4
CHOP
ND2
ND4
NRF1
MFN1
GAPDH

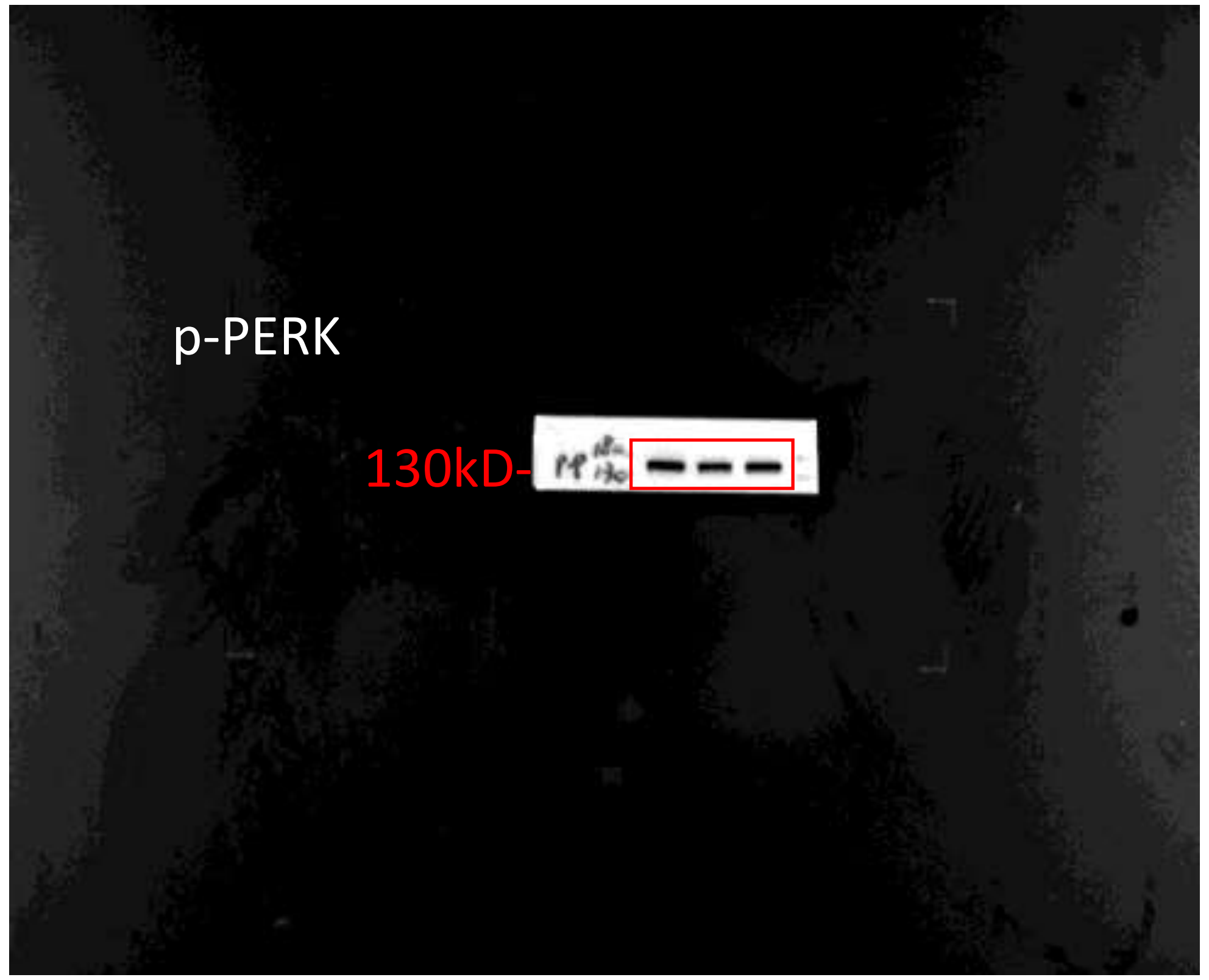
p-PERK
130kD-

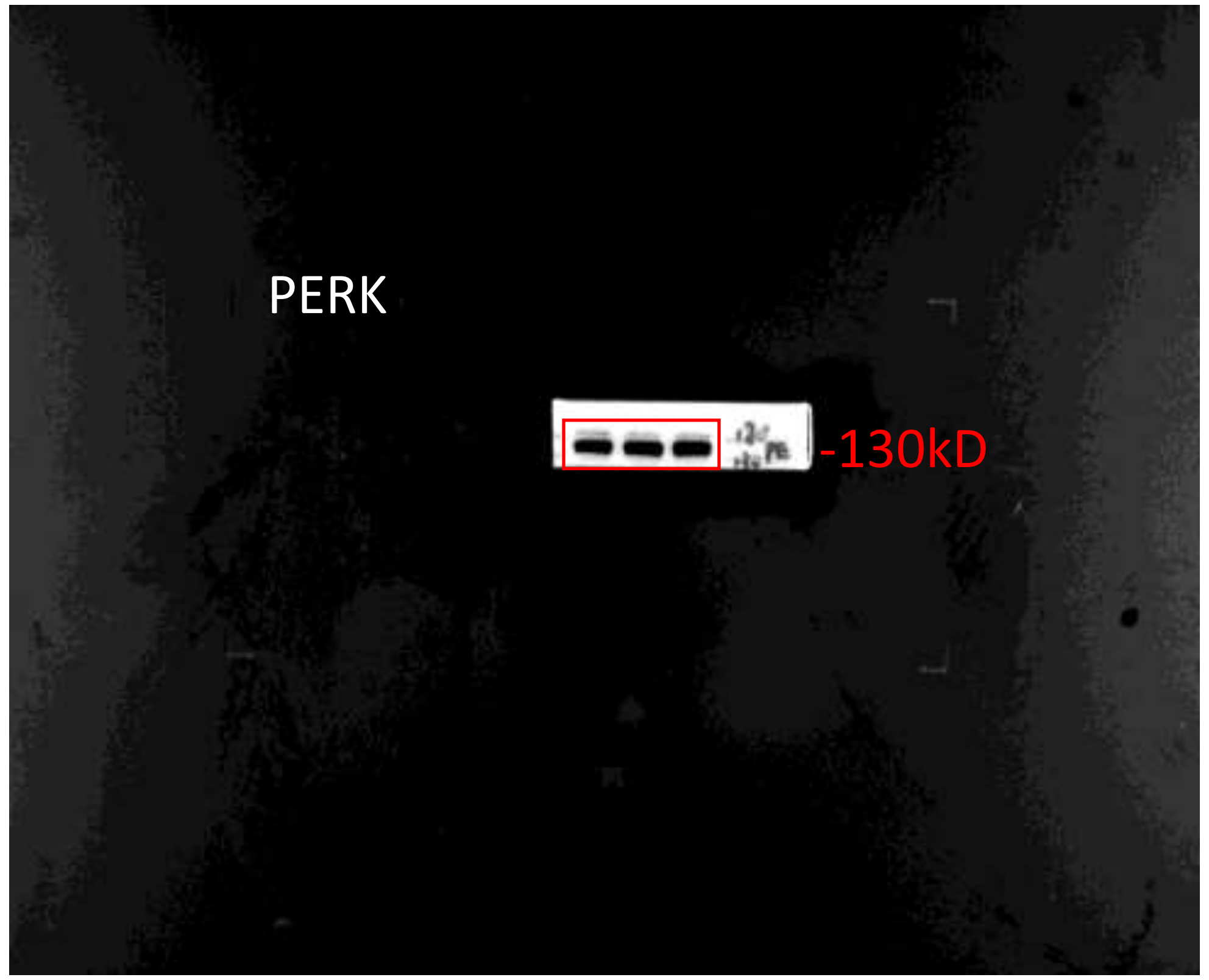
PERK
-130kD

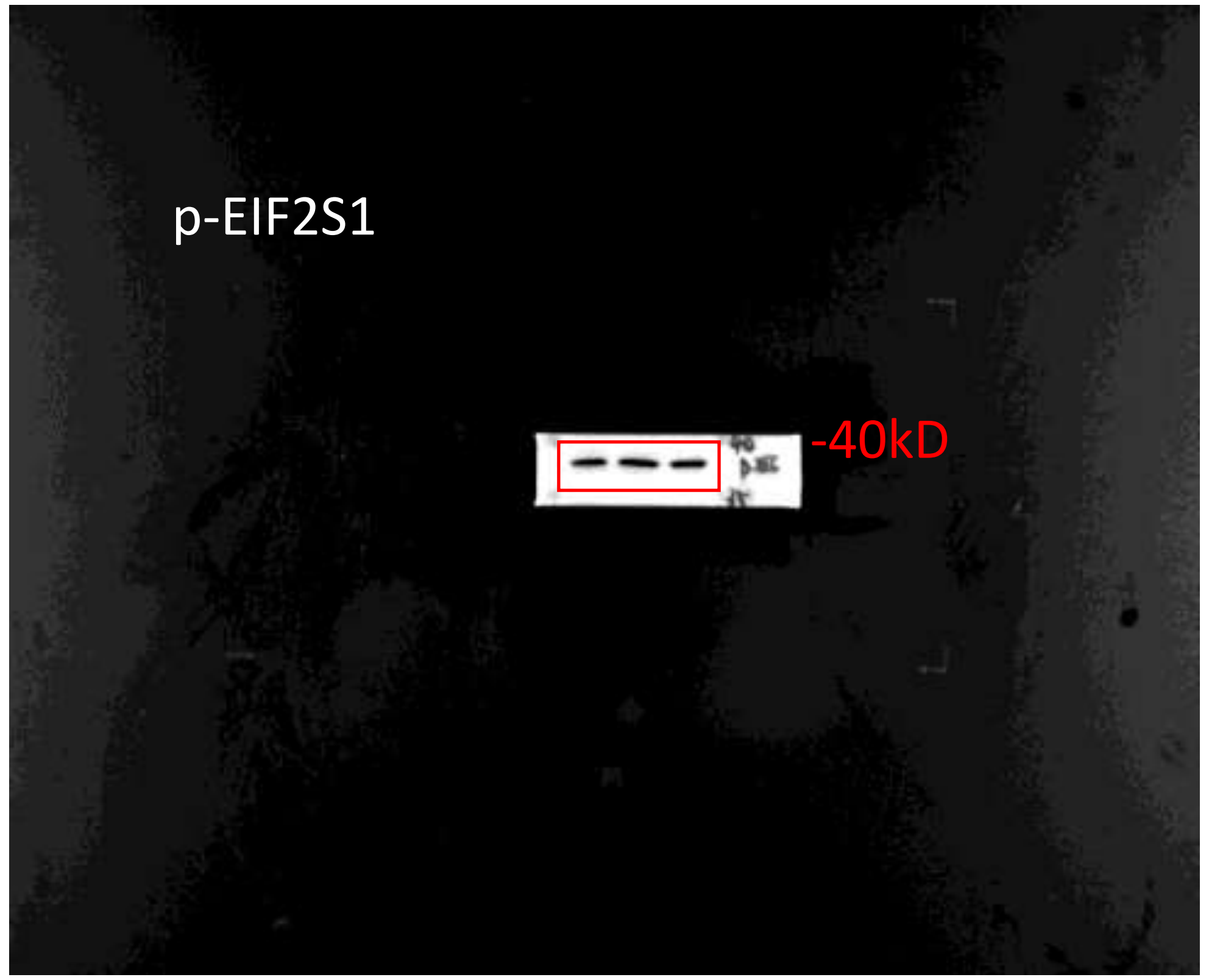
p-EIF2S1
-40kD

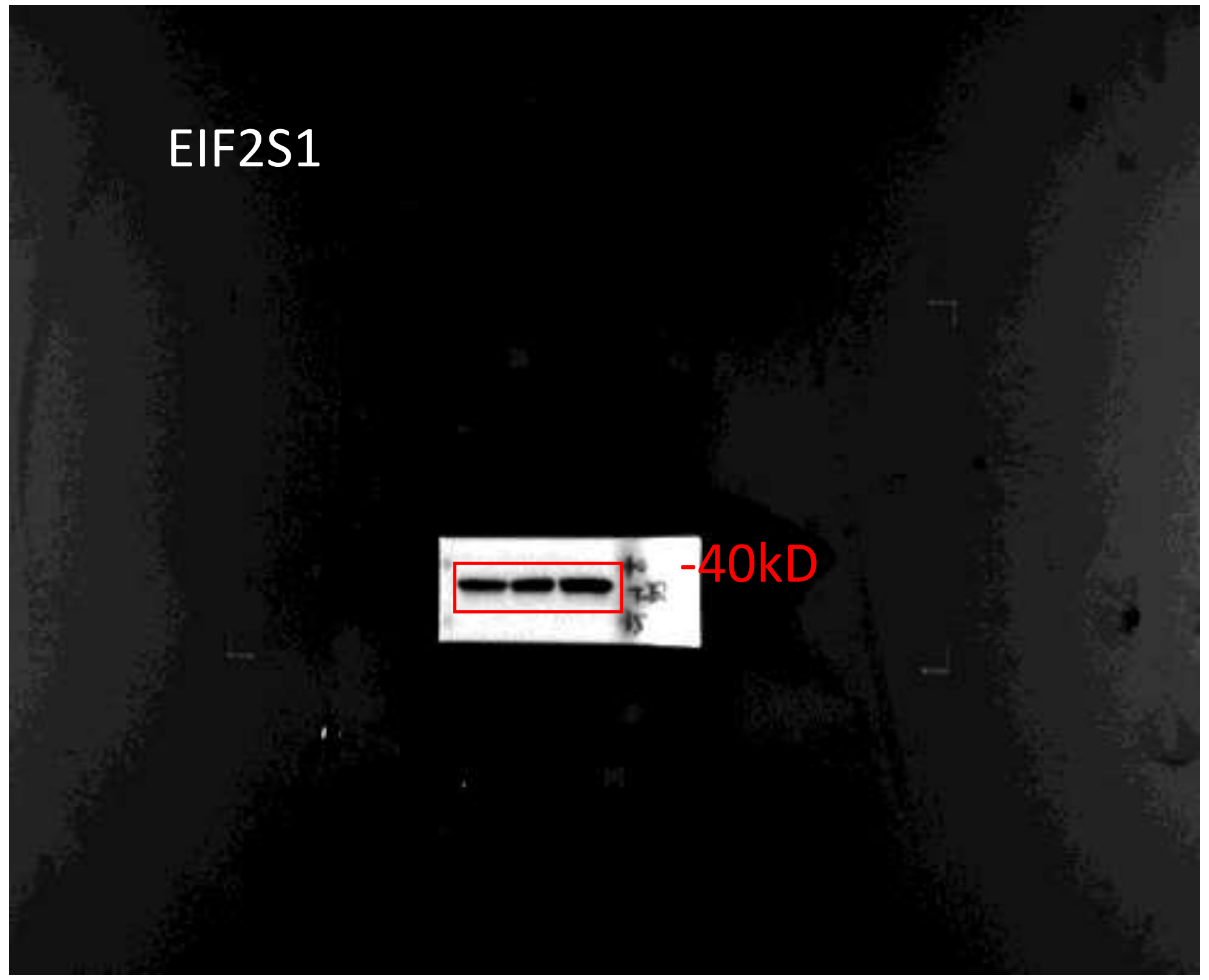
EIF2S1
-40kD

Figure5

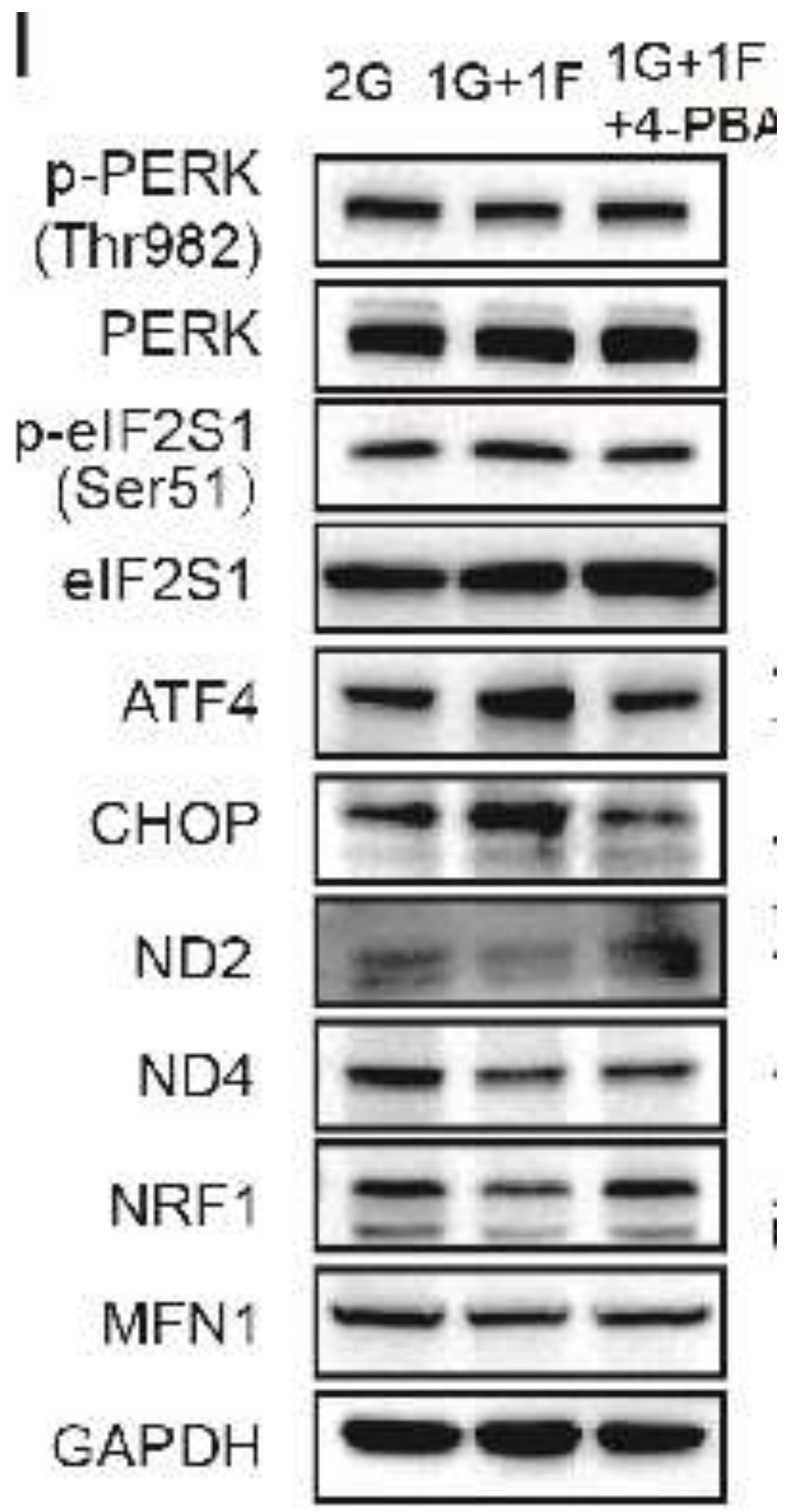
I
2G
1G+1F
1G+1F
+4-PBA
p-PERK
(Thr982)
PERK
p-eIF2S1
(Ser51)
eIF2S1
ATF4
CHOP
ND2
ND4
NRF1
MFN1
GAPDH

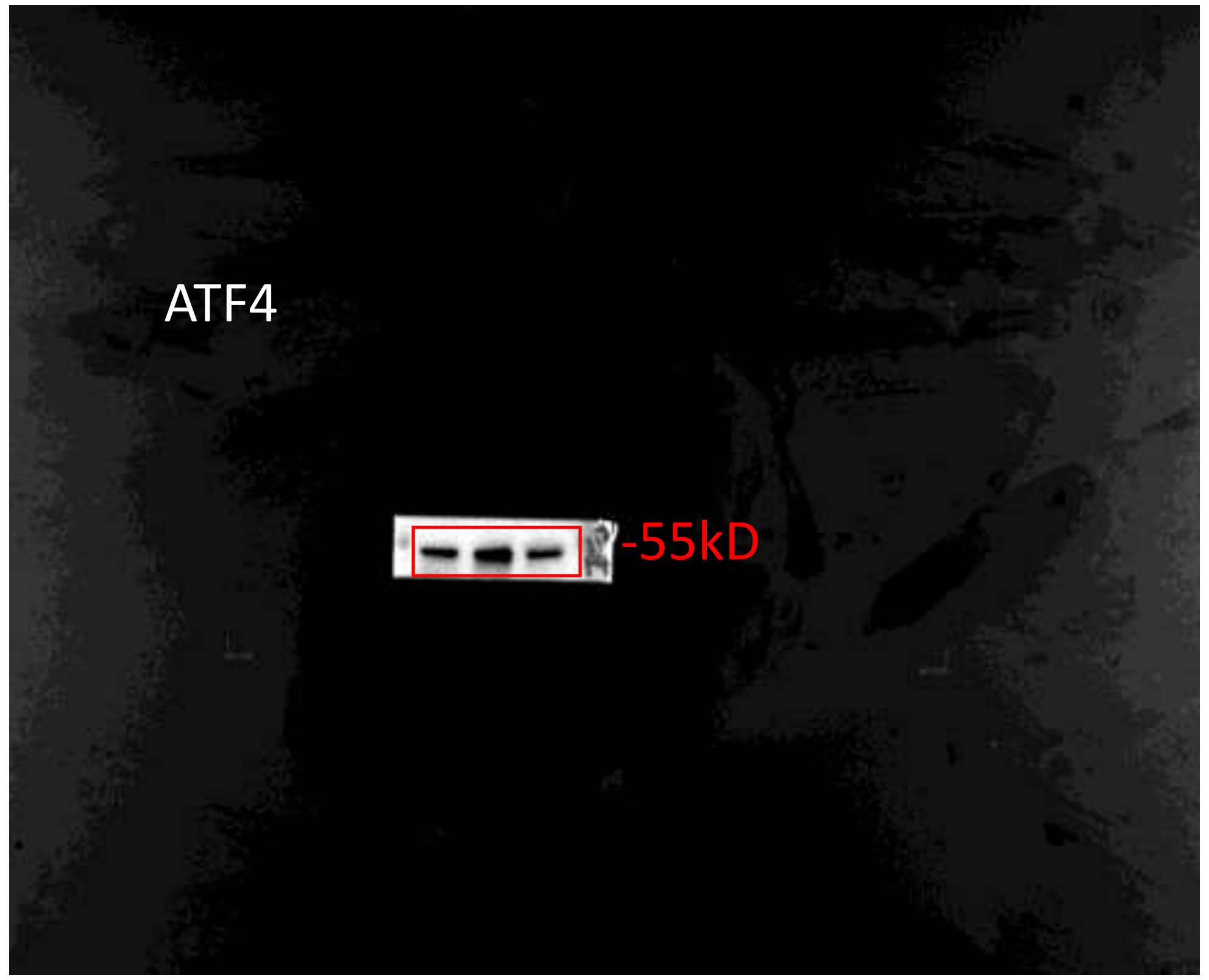
ATF4
-55kD

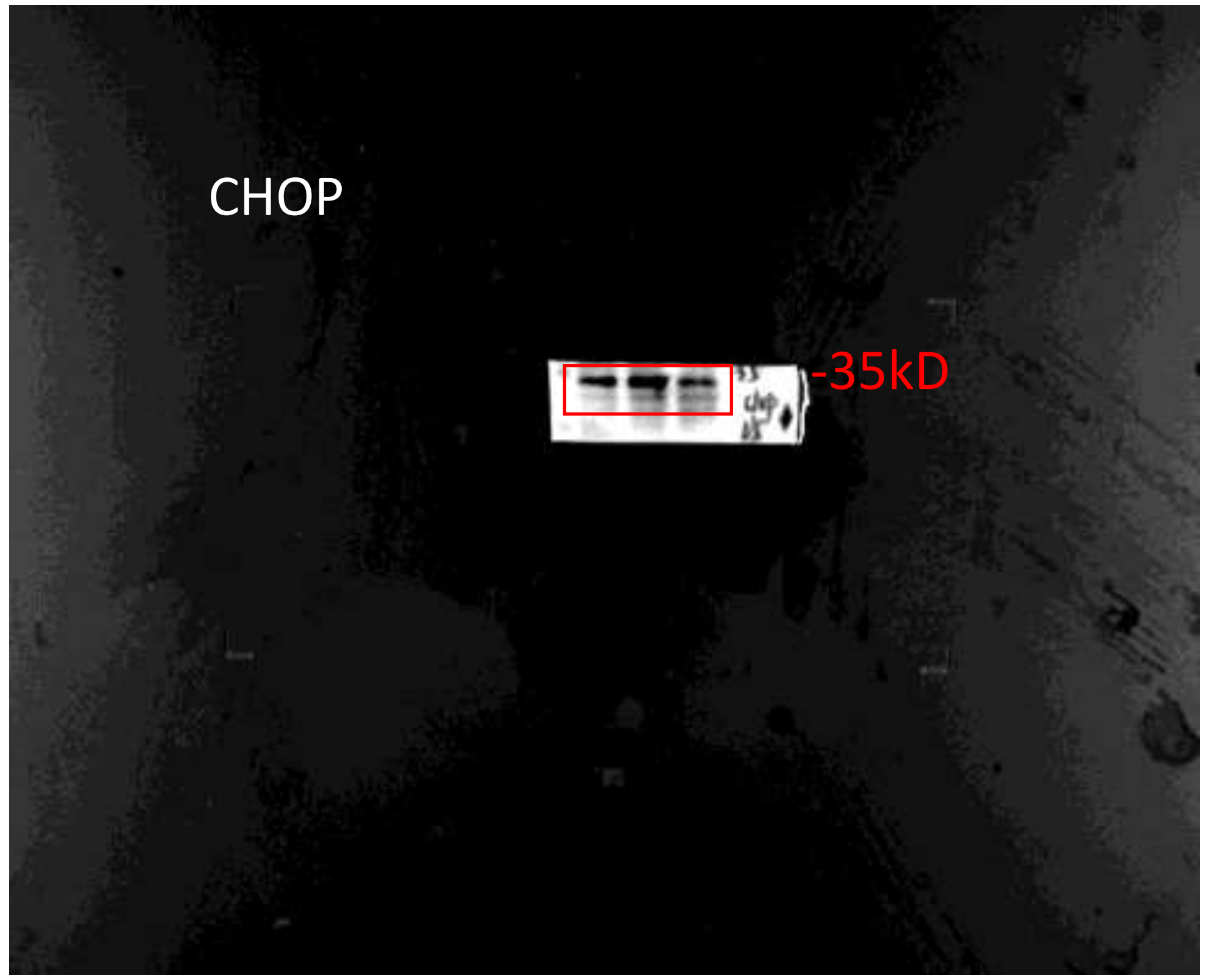
CHOP
-35kD

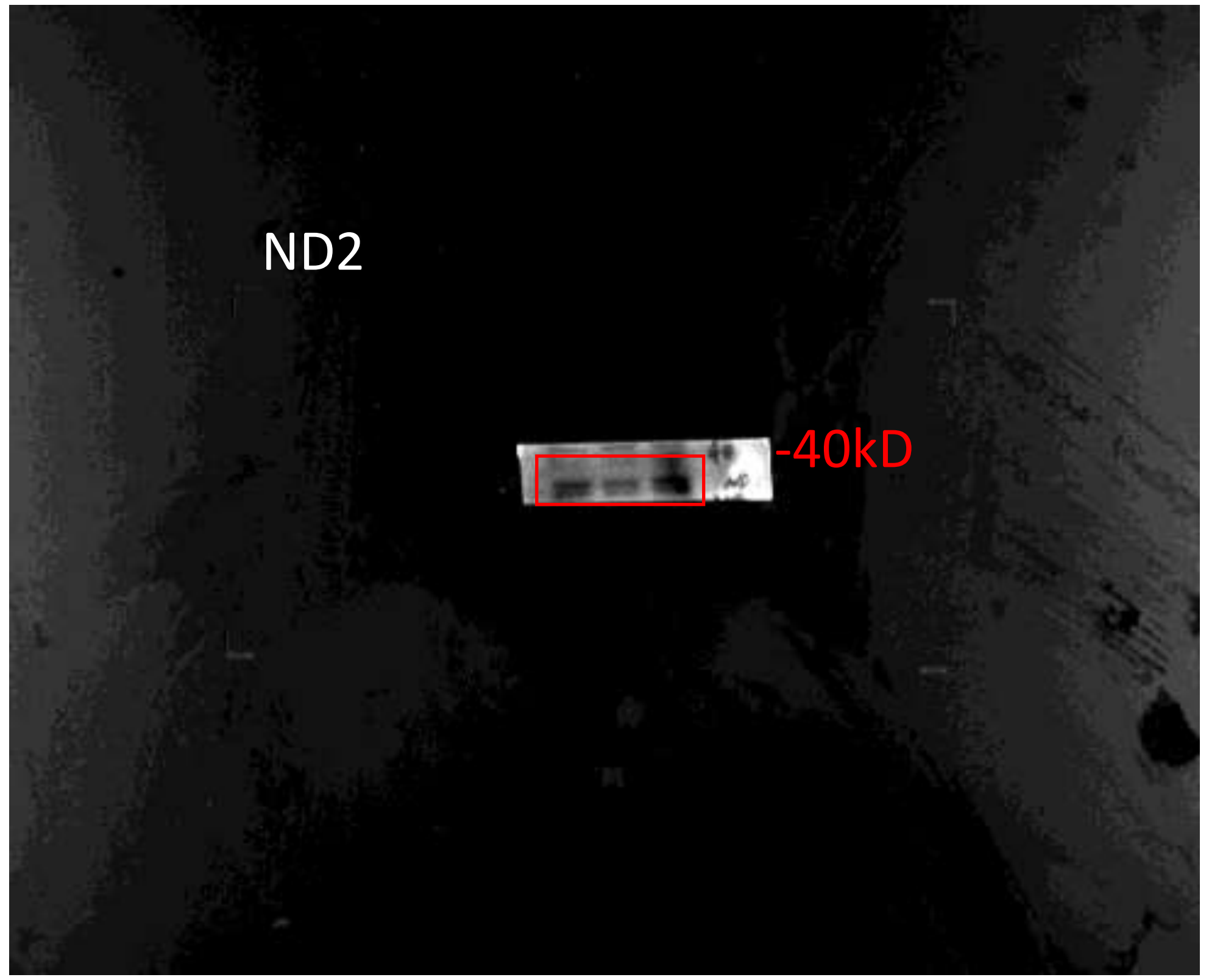
ND2
-40kD

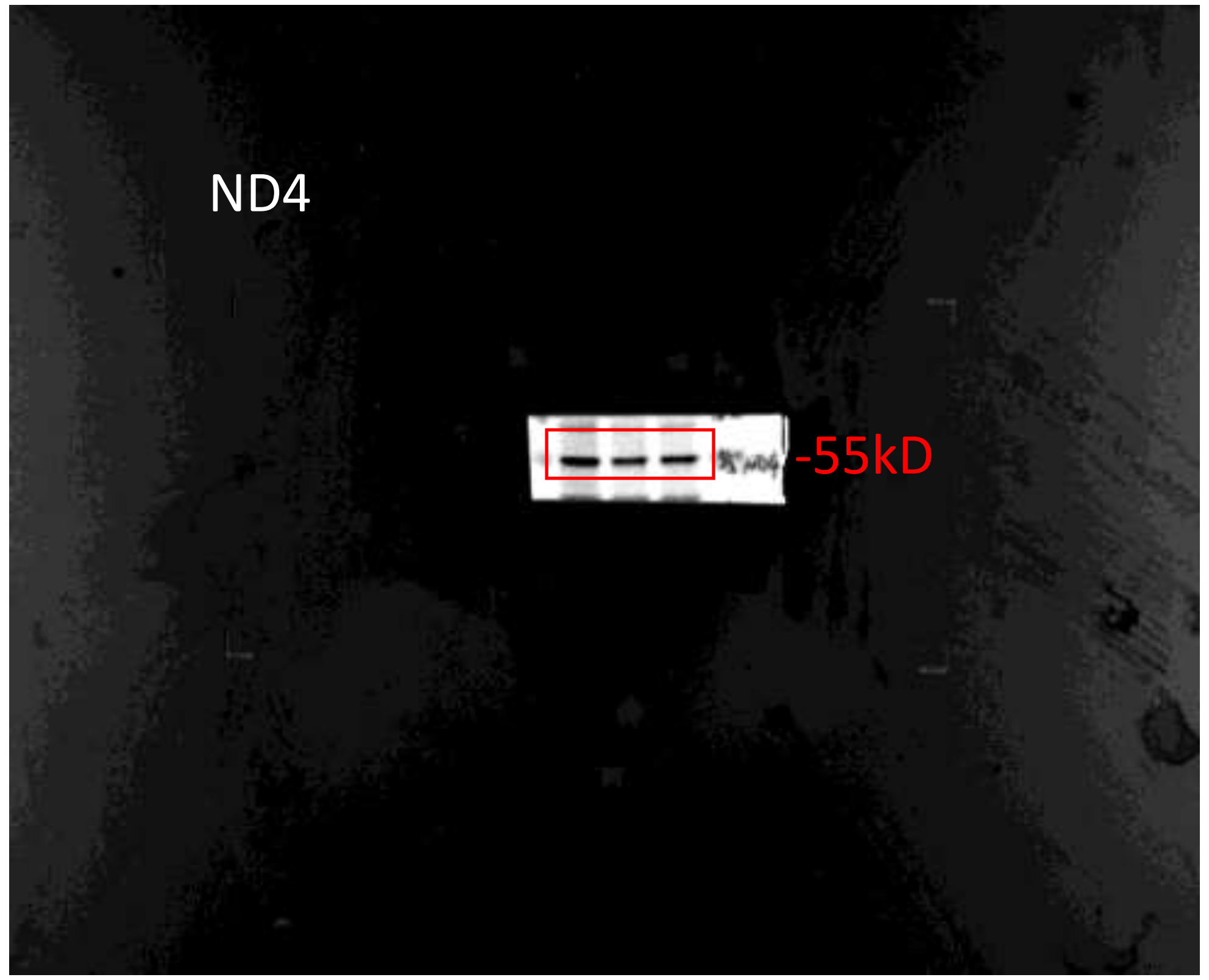
ND4
-55kD

Figure5

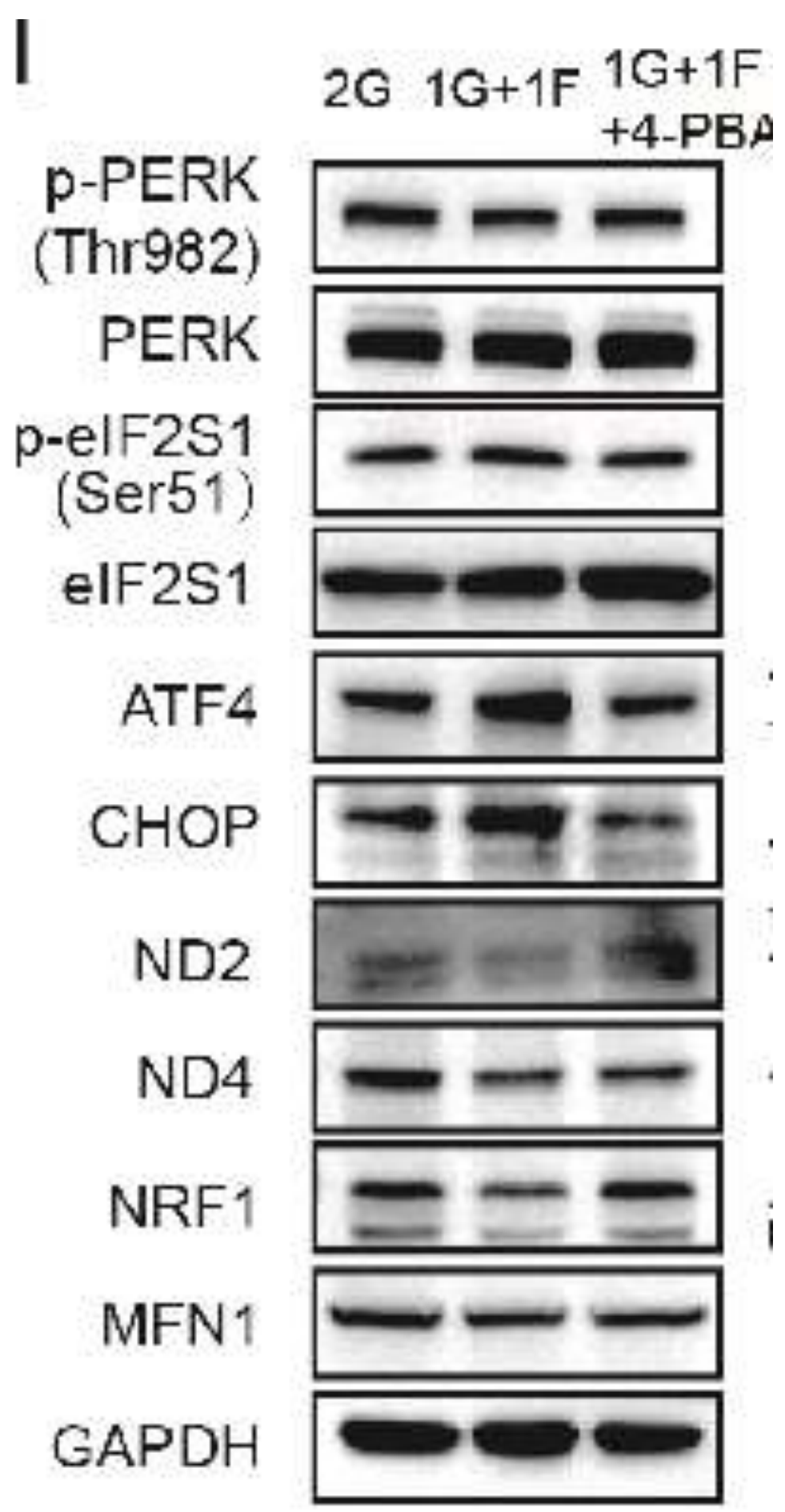
I
2G
1G+1F
1G+1F +4-PBA
p-PERK (Thr982)
PERK
p-eIF2S1 (Ser51)
eIF2S1
ATF4
CHOP
ND2
ND4
NRF1
MFN1
GAPDH

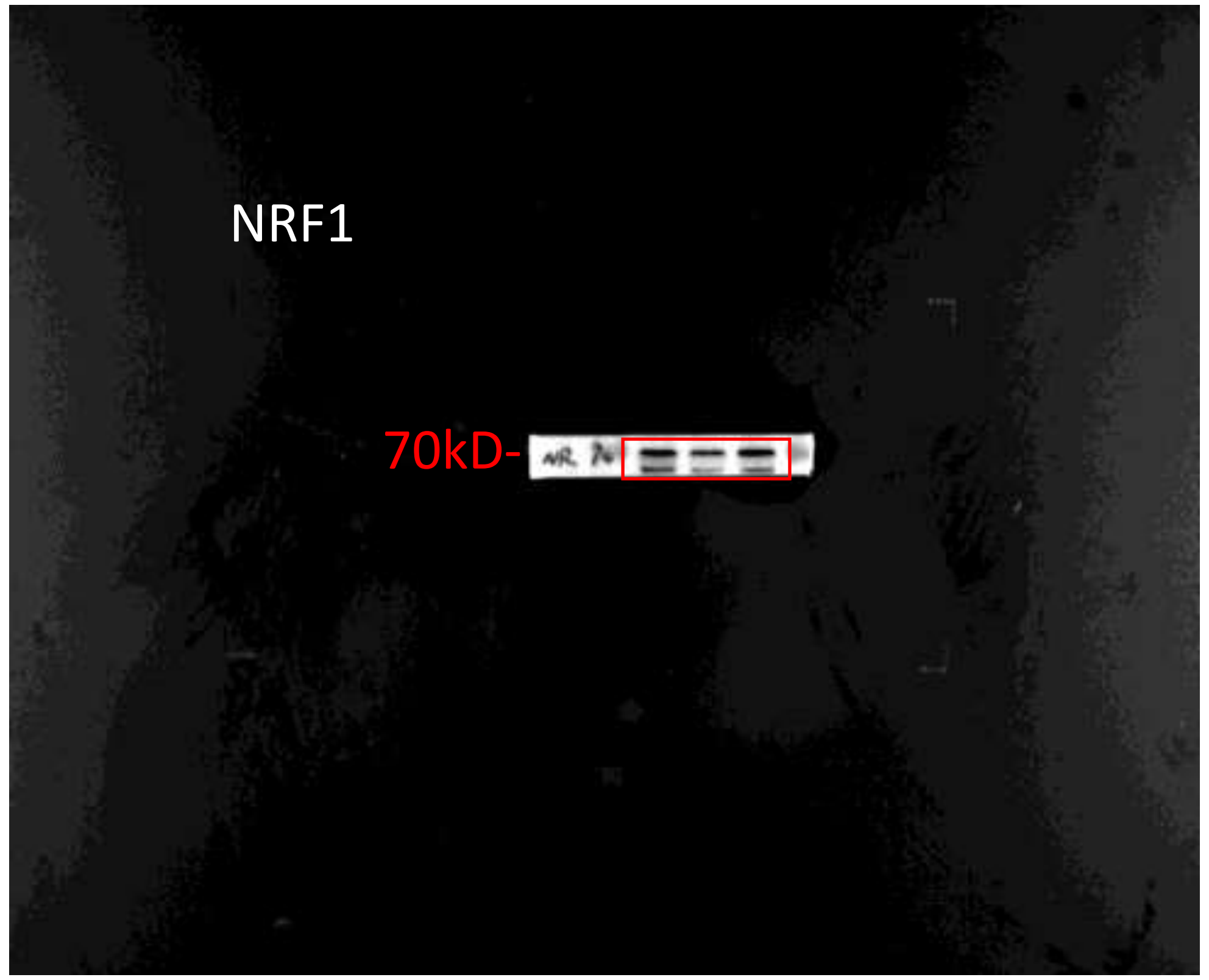
NRF1
70kD-

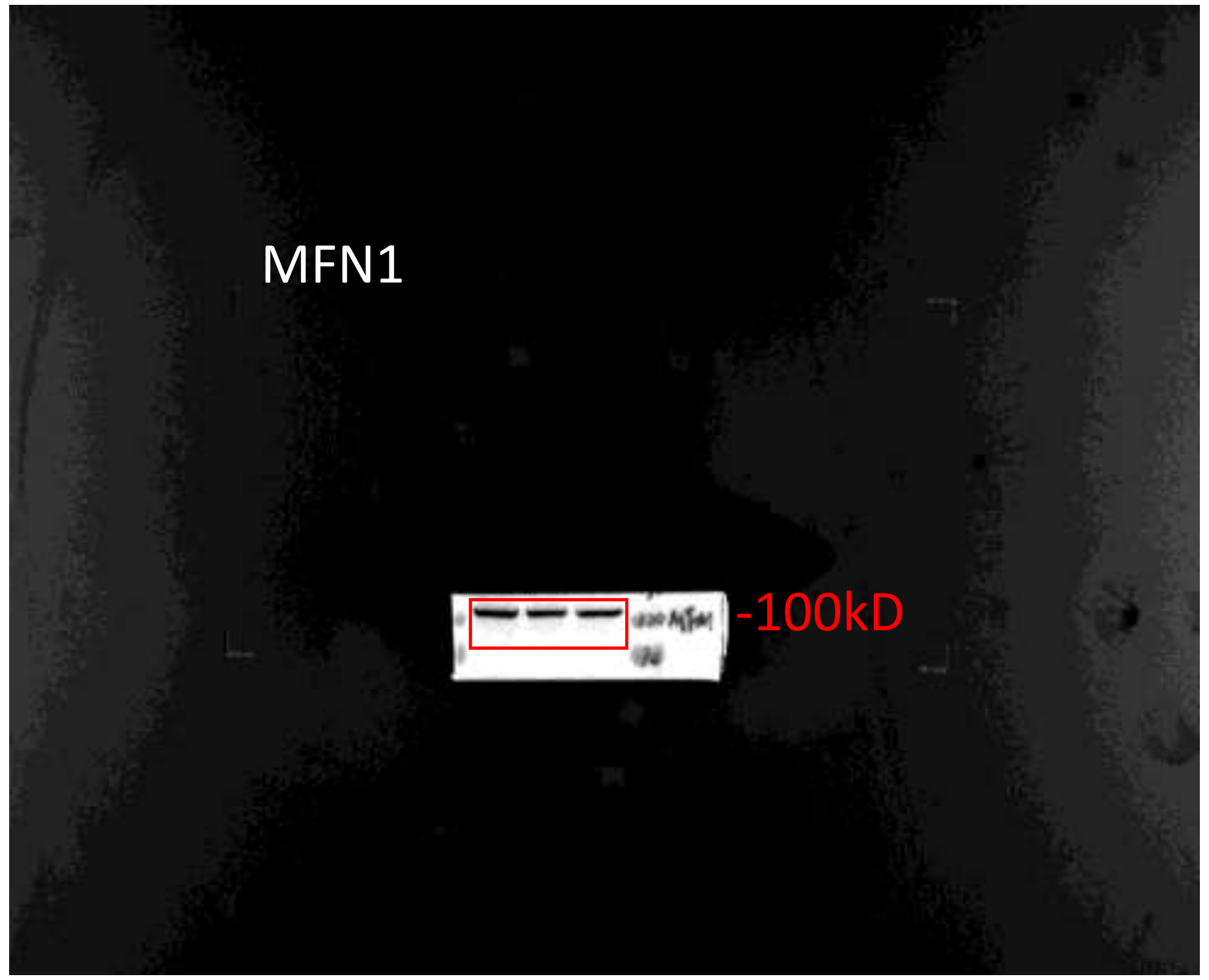
MFN1
-100kD

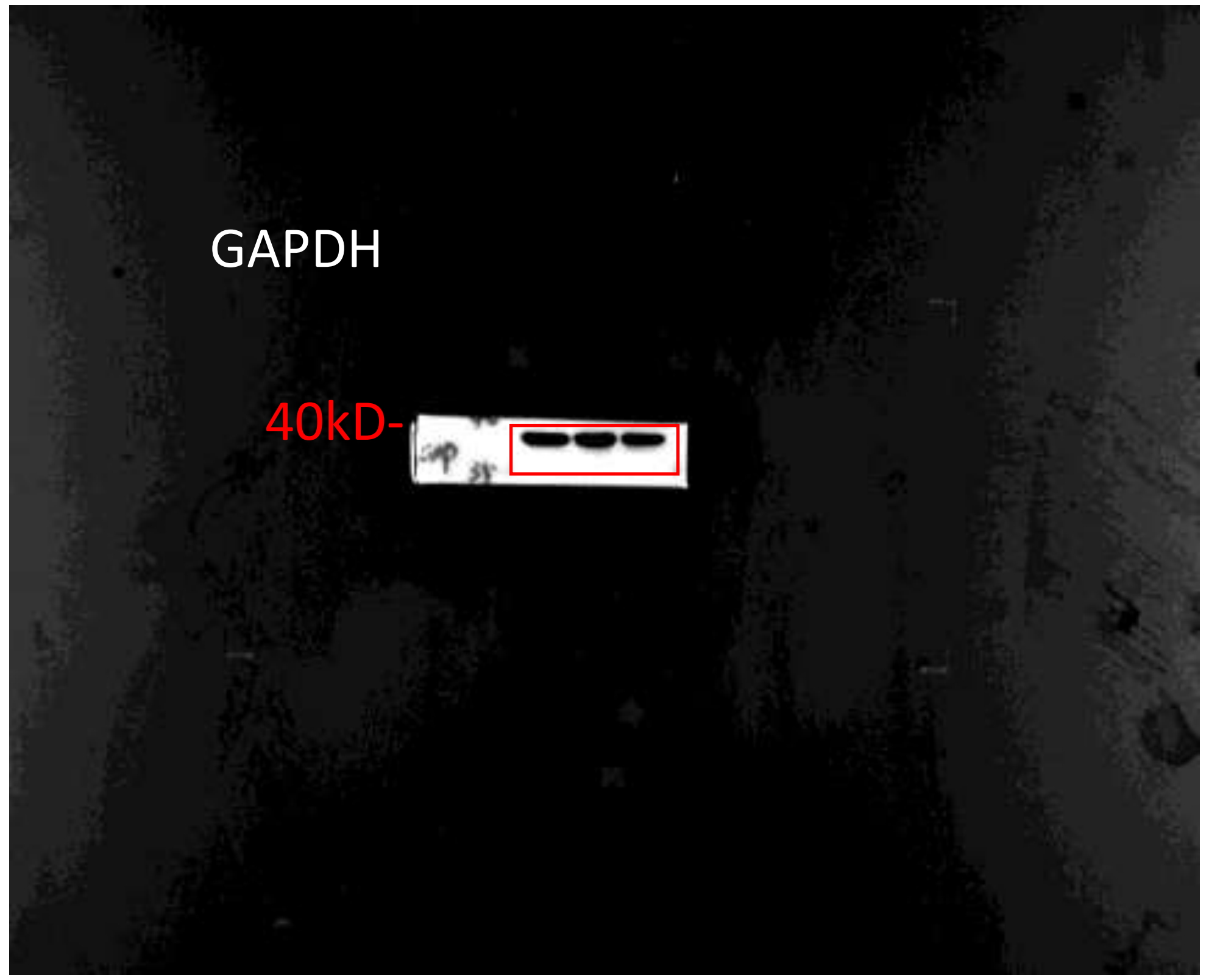
GAPDH
40kD-

Figure5

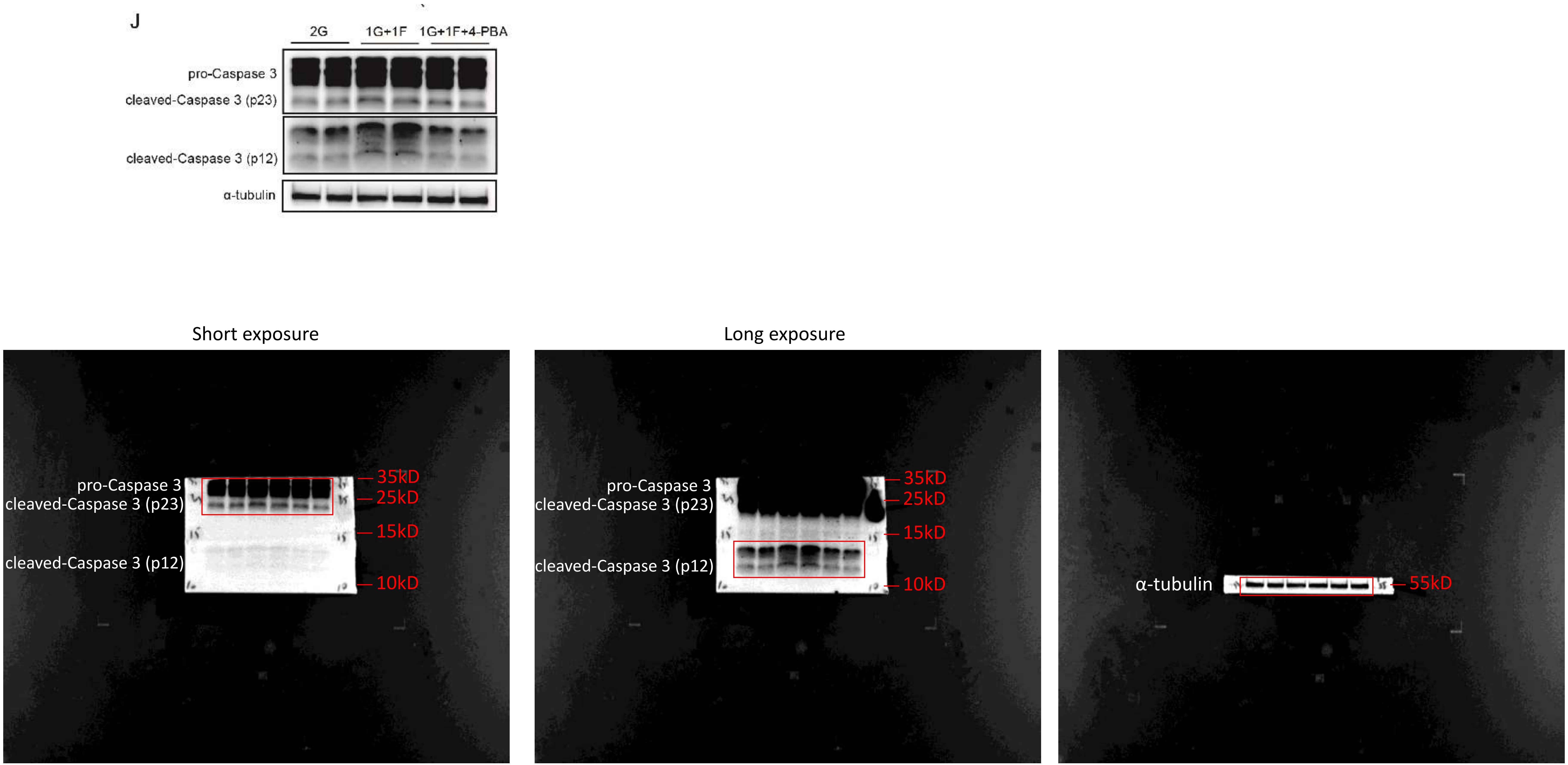
J
2G
1G+1F
1G+1F+4-PBA
pro-Caspase 3
cleaved-Caspase 3 (p23)
cleaved-Caspase 3 (p12)
α-tubulin
Short exposure
Long exposure
35kD
25kD
15kD
10kD
55kD

Figure6

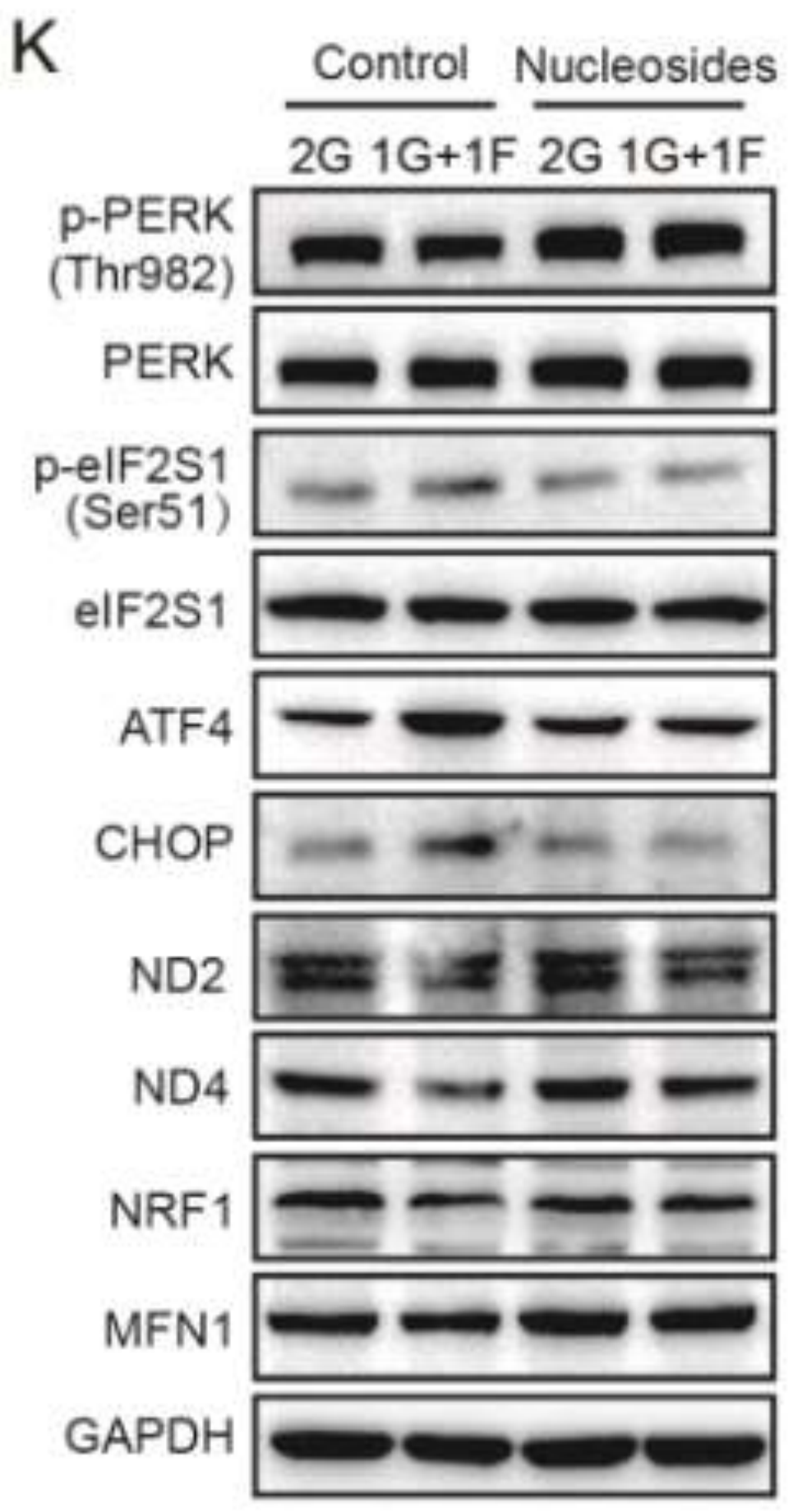
K
Control
Nucleosides
2G 1G+1F 2G 1G+1F
p-PERK (Thr982)
PERK
p-eIF2S1 (Ser51)
eIF2S1
ATF4
CHOP
ND2
ND4
NRF1
MFN1
GAPDH

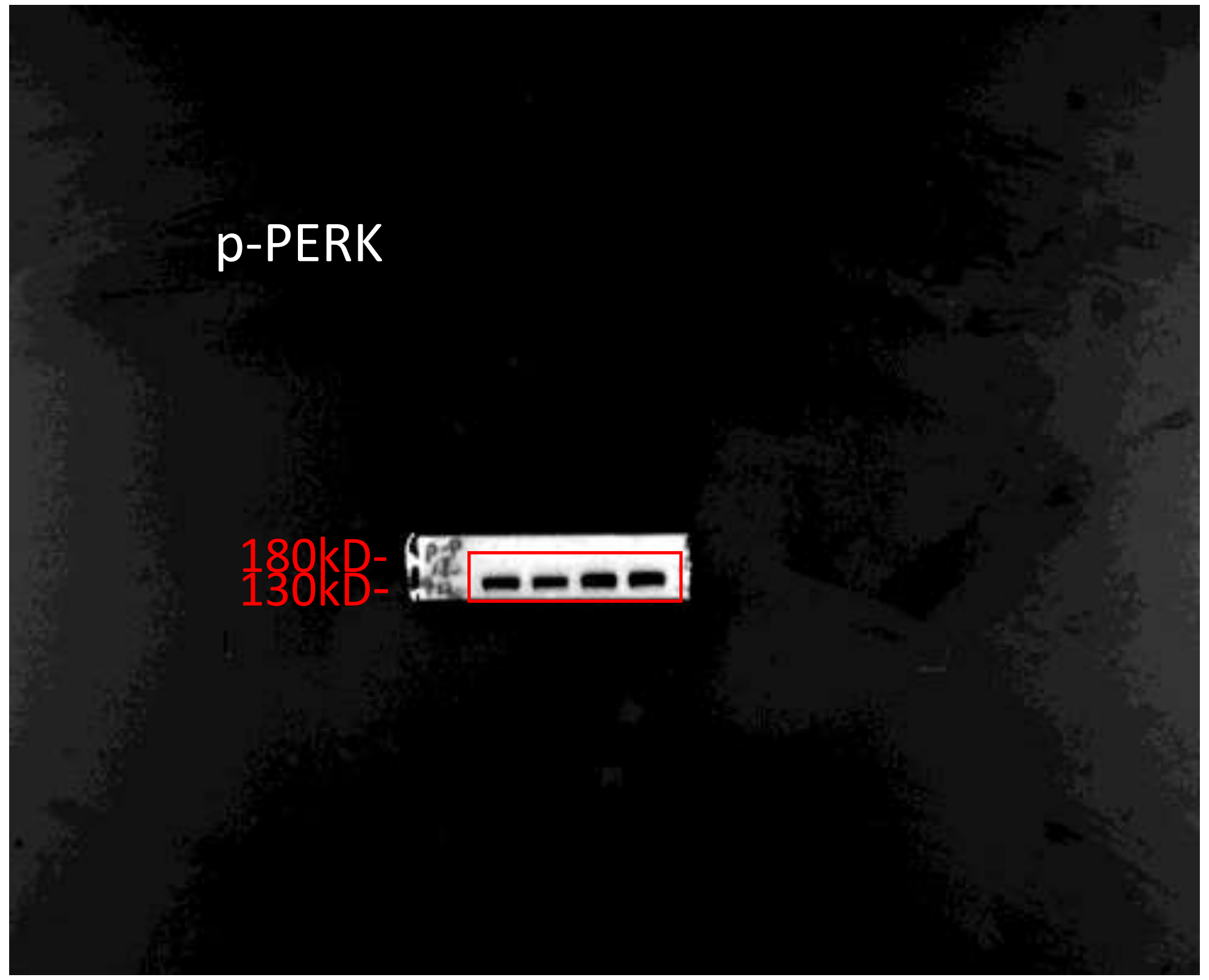
p-PERK
180kD-
130kD-

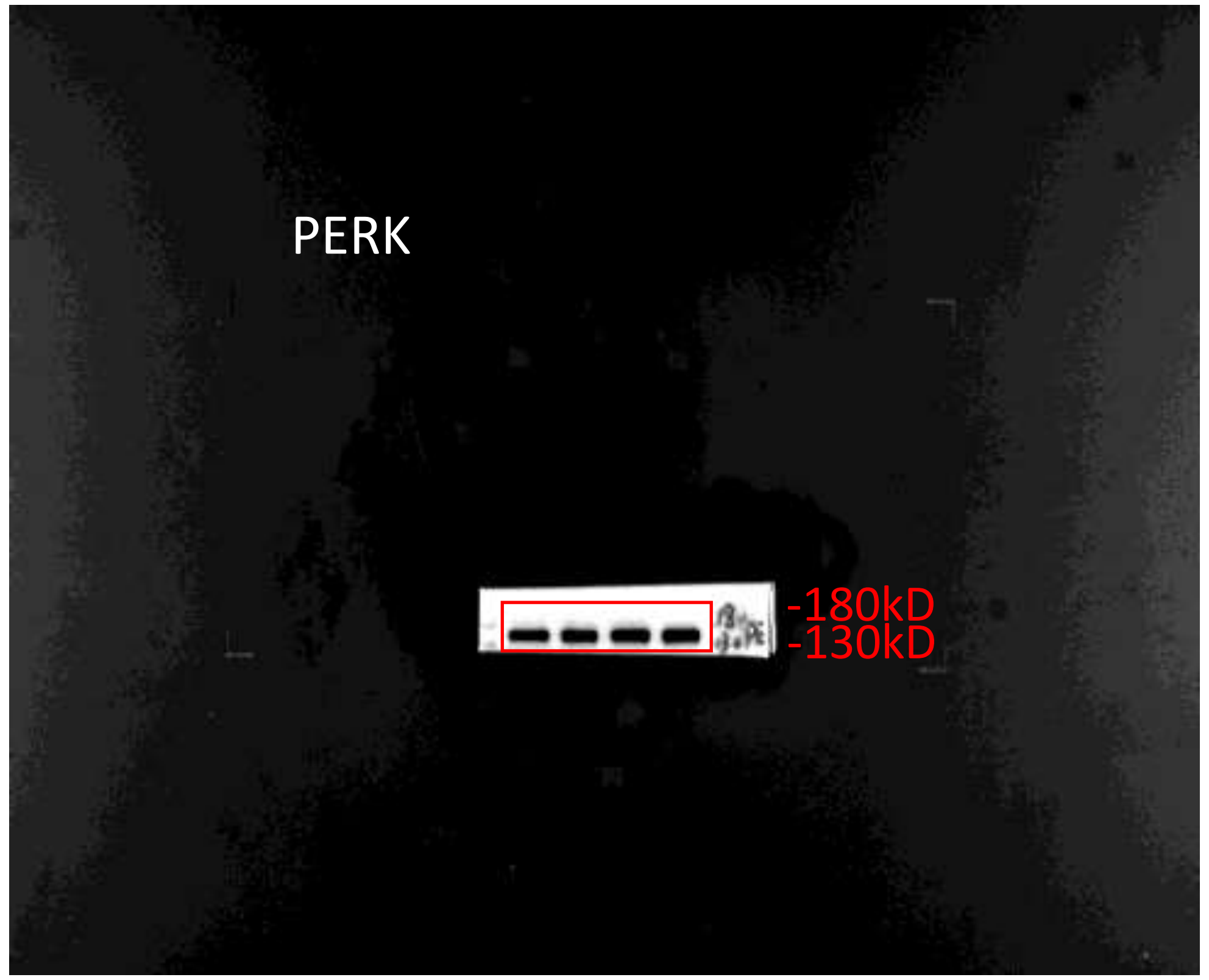
PERK
-180kD
-130kD

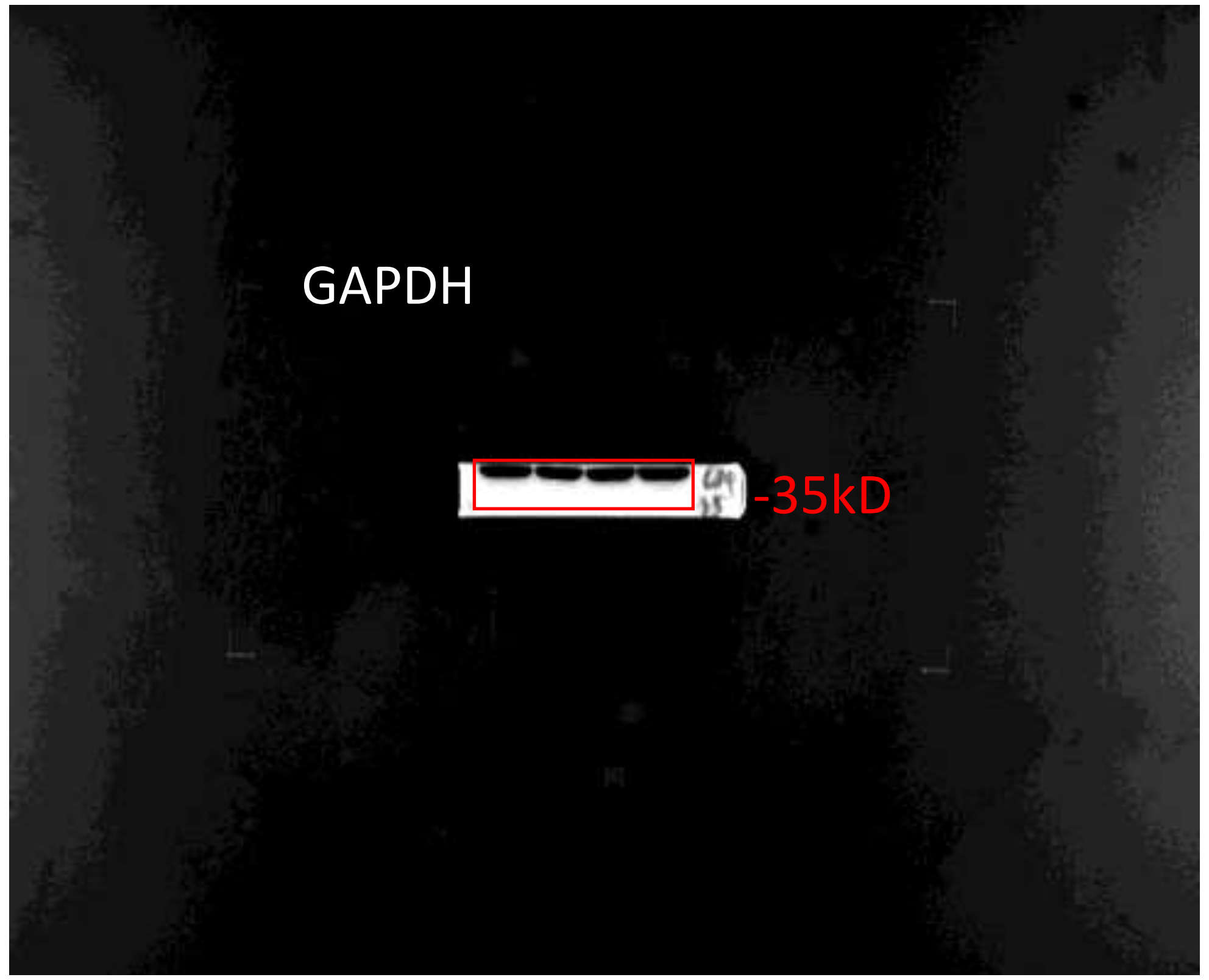
GAPDH
-35kD

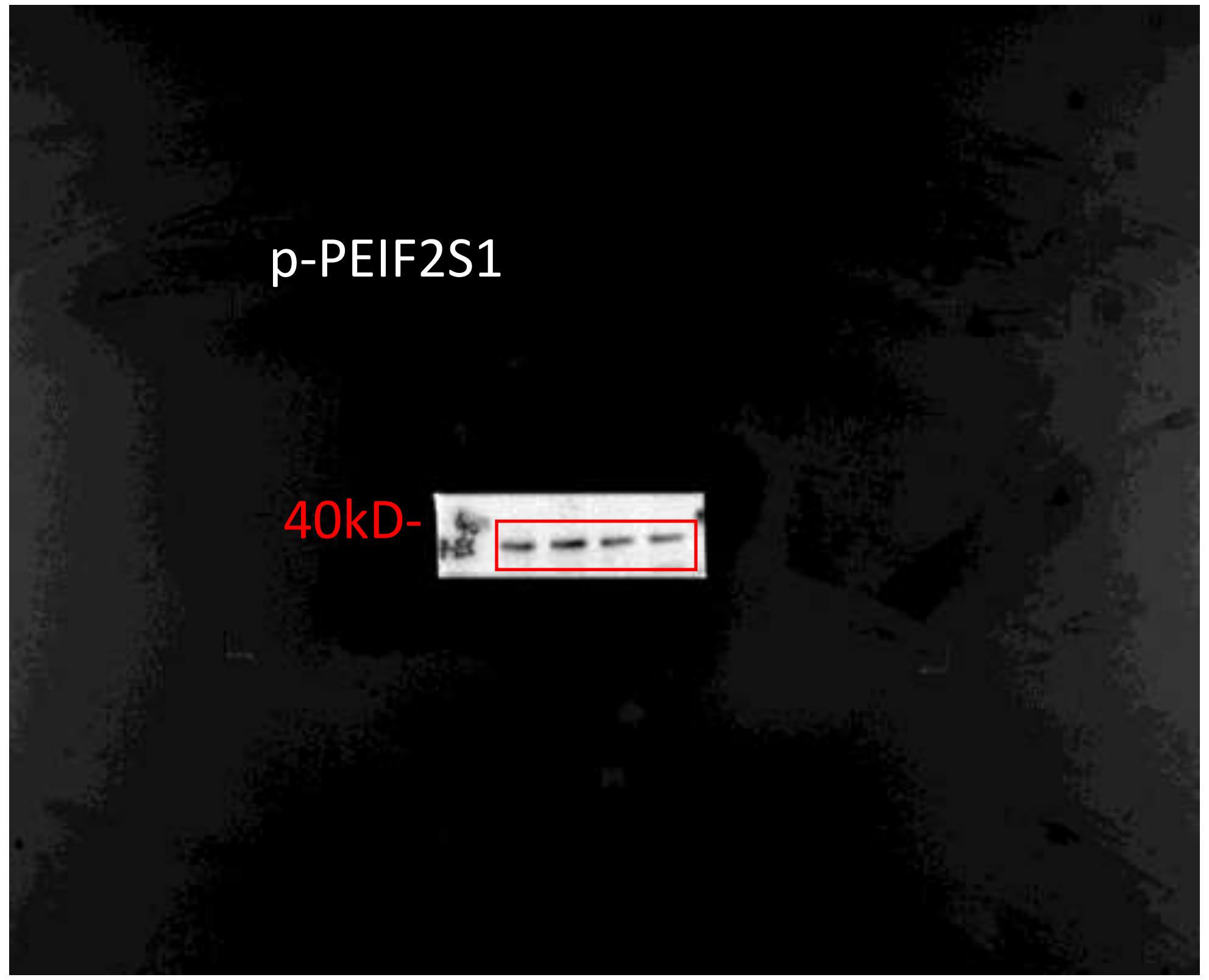
p-PEIF2S1
40kD-

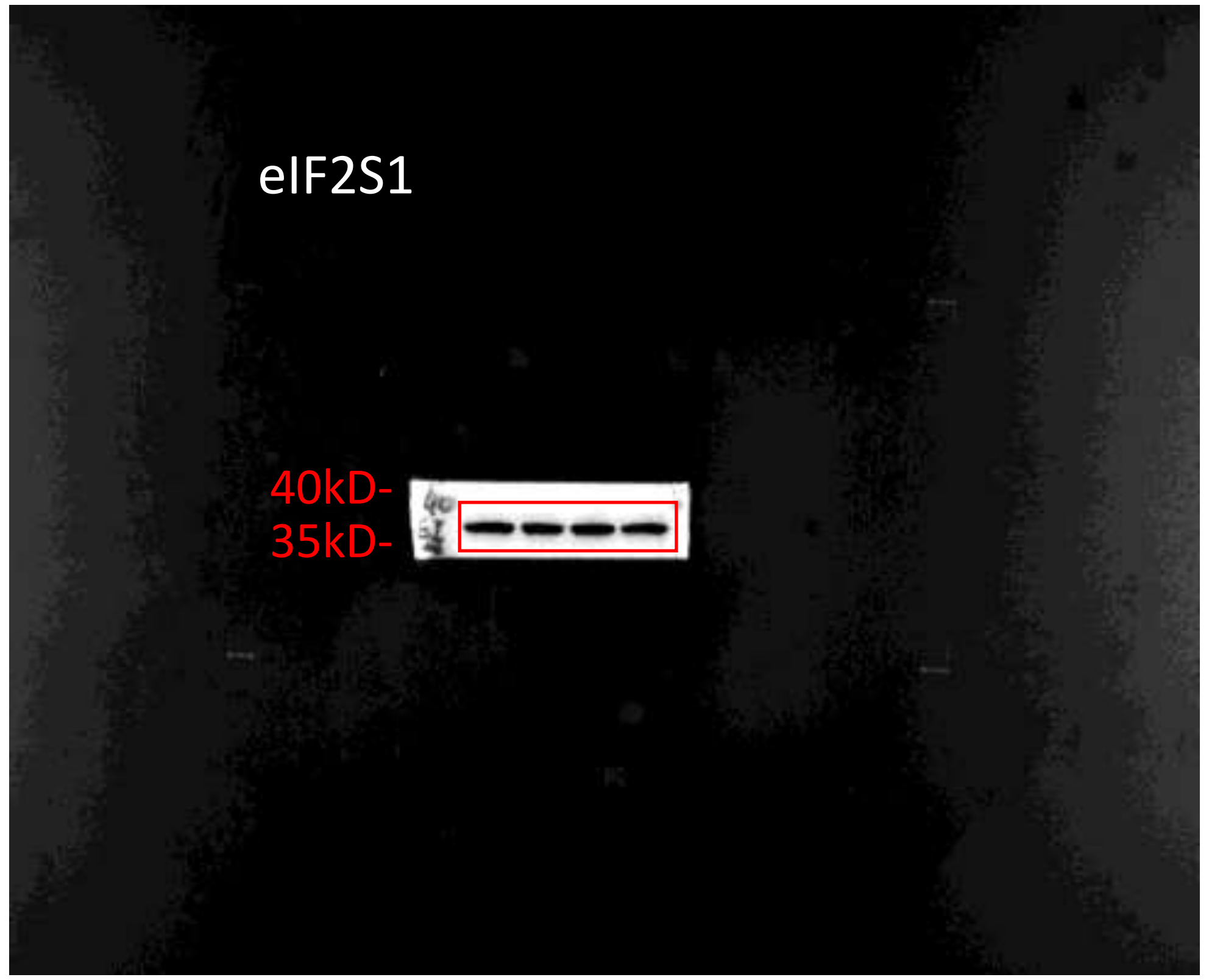
eIF2S1
40kD-
35kD-

Figure6

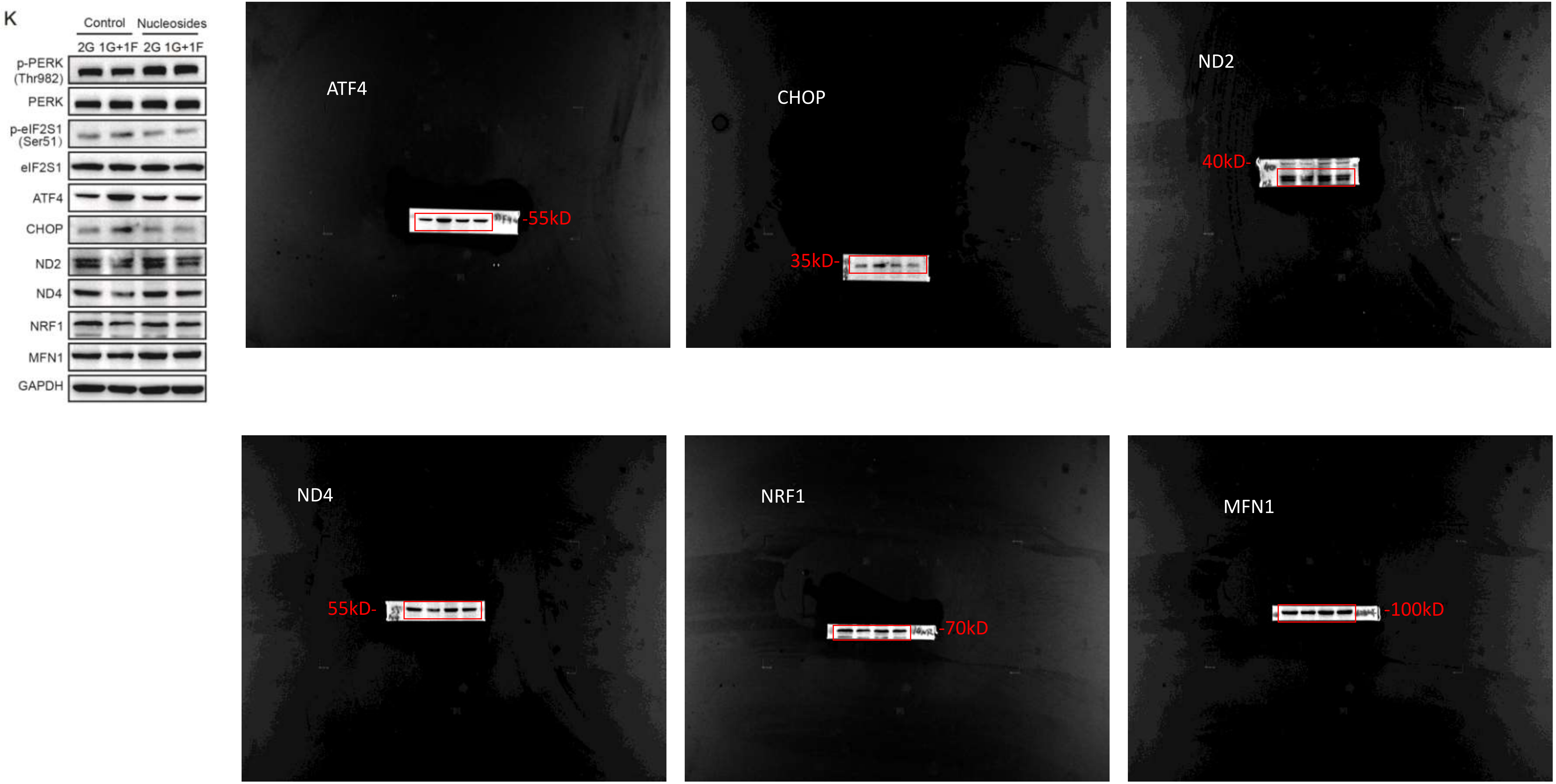
K
Control
Nucleosides
2G 1G+1F 2G 1G+1F
p-PERK (Thr982)
PERK
p-eIF2S1 (Ser51)
eIF2S1
ATF4
CHOP
ND2
ND4
NRF1
MFN1
GAPDH
ATF4
-55kD
CHOP
35kD-
ND2
40kD-
ND4
55kD-
NRF1
-70kD
MFN1
-100kD

Figure6

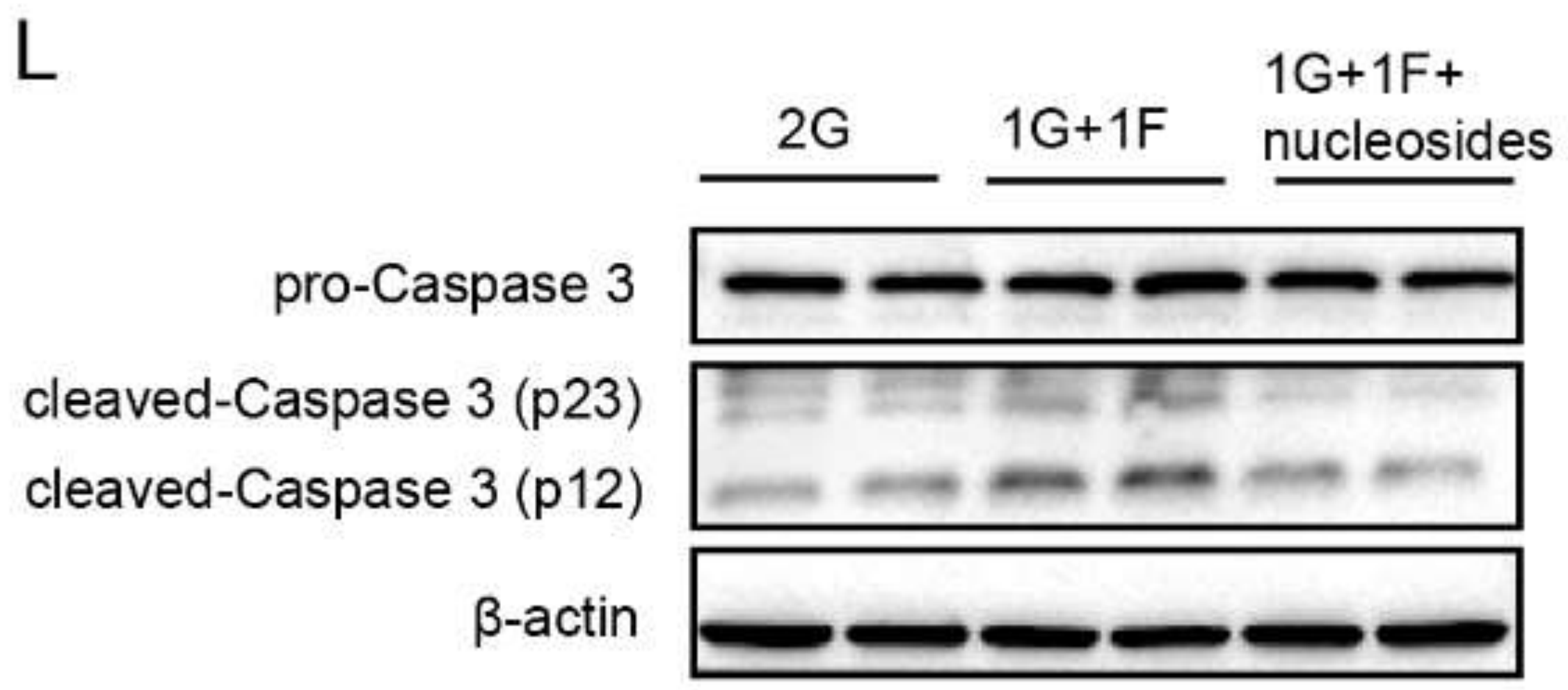

Short exposure

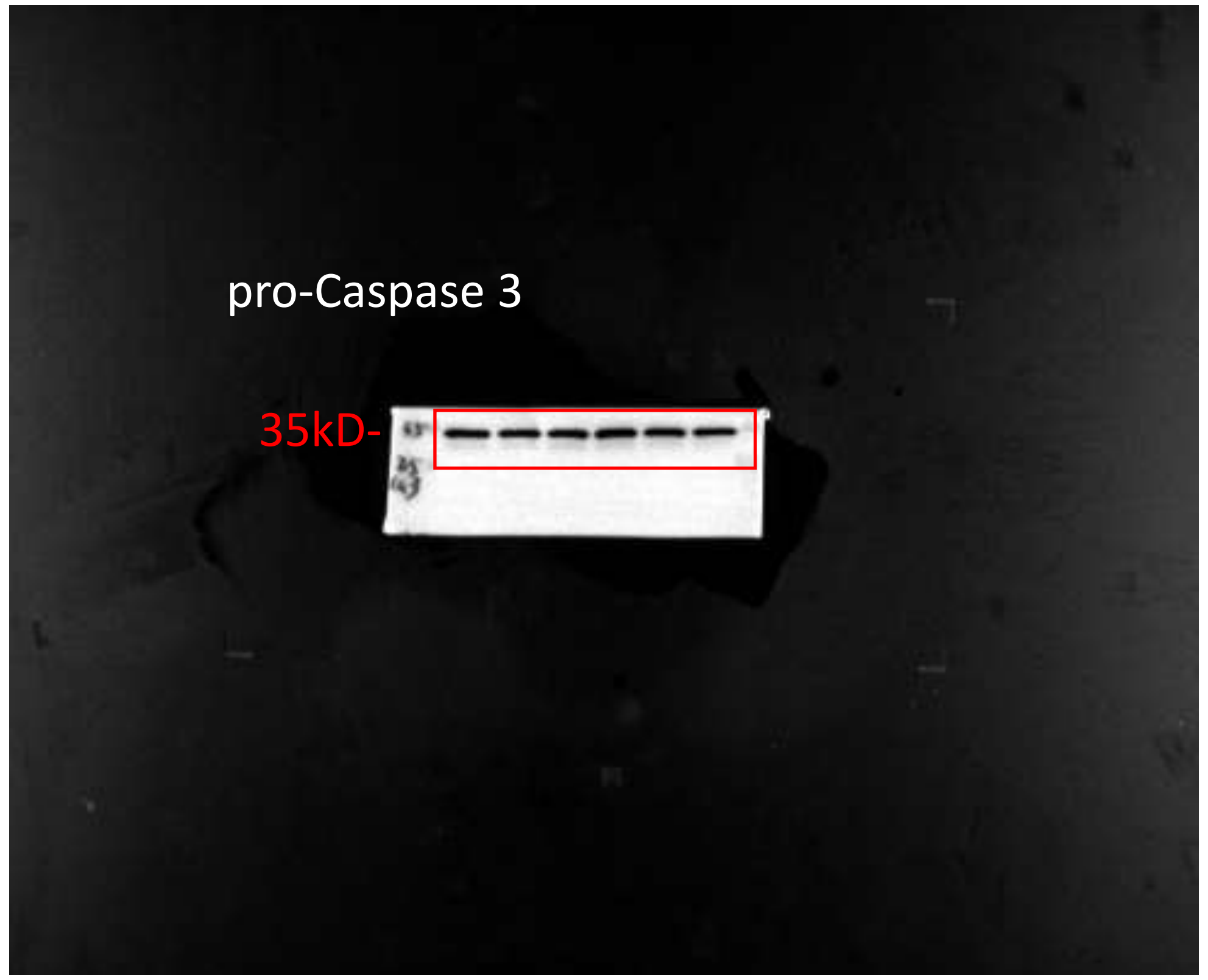

Long exposure

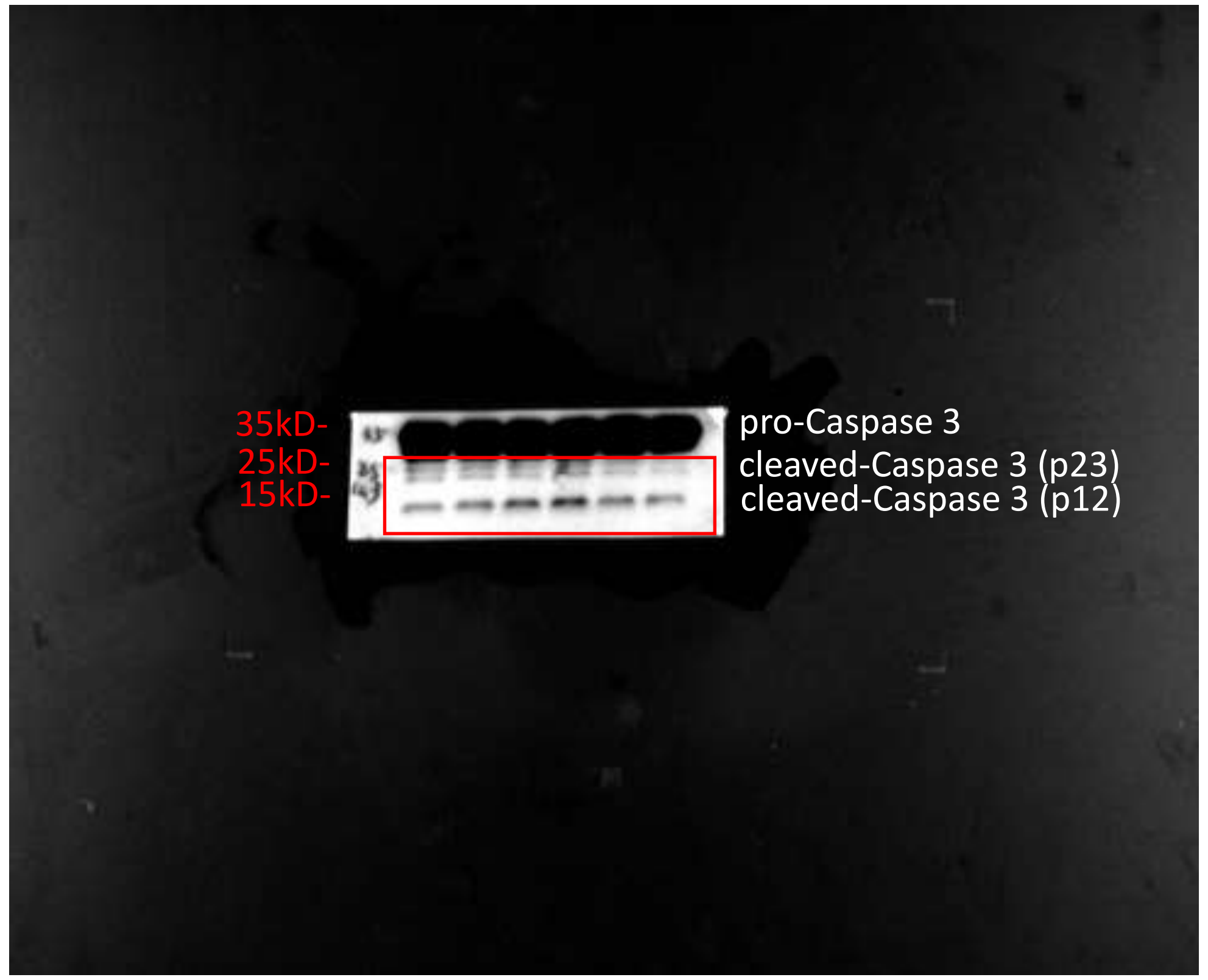

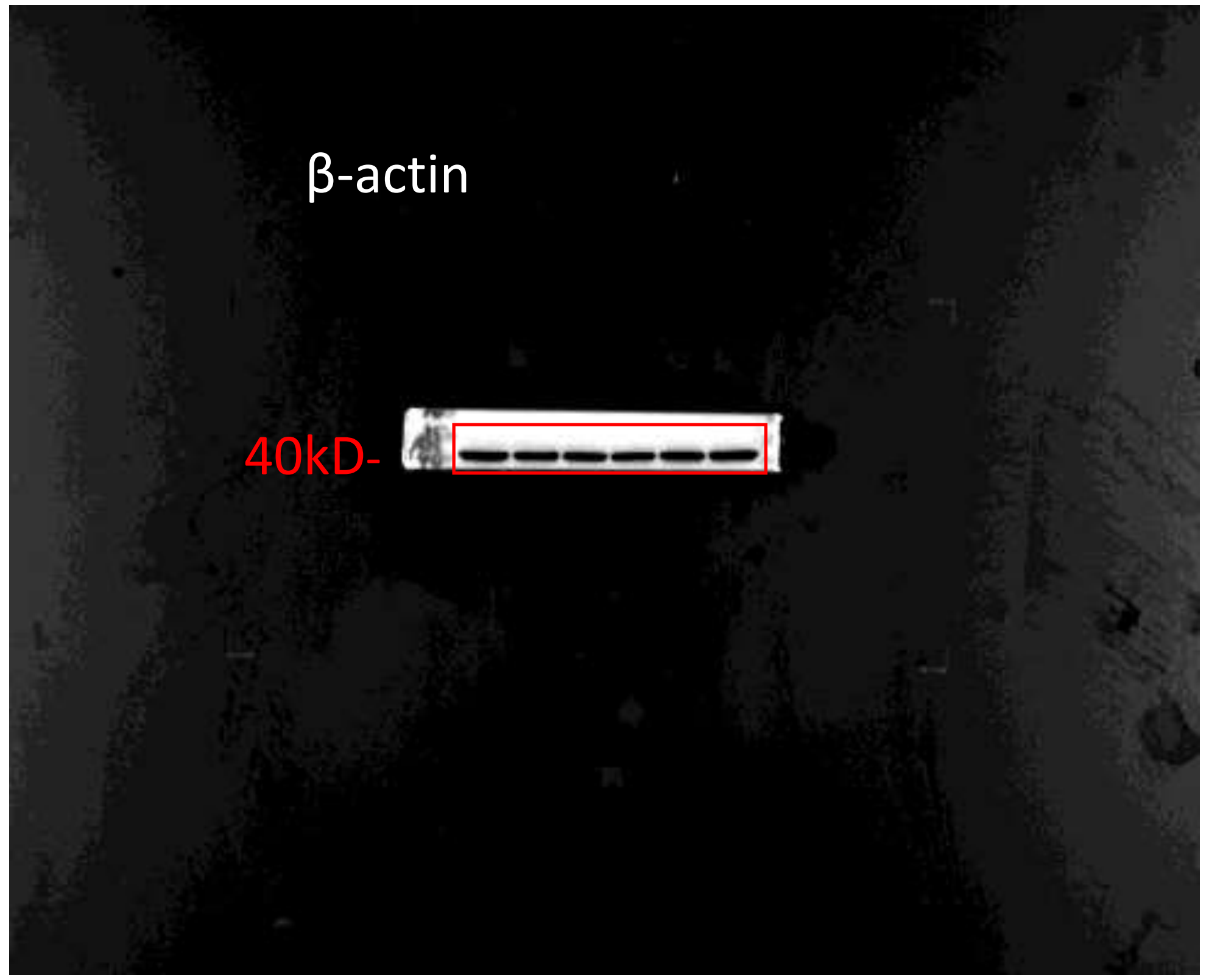

FigureS1

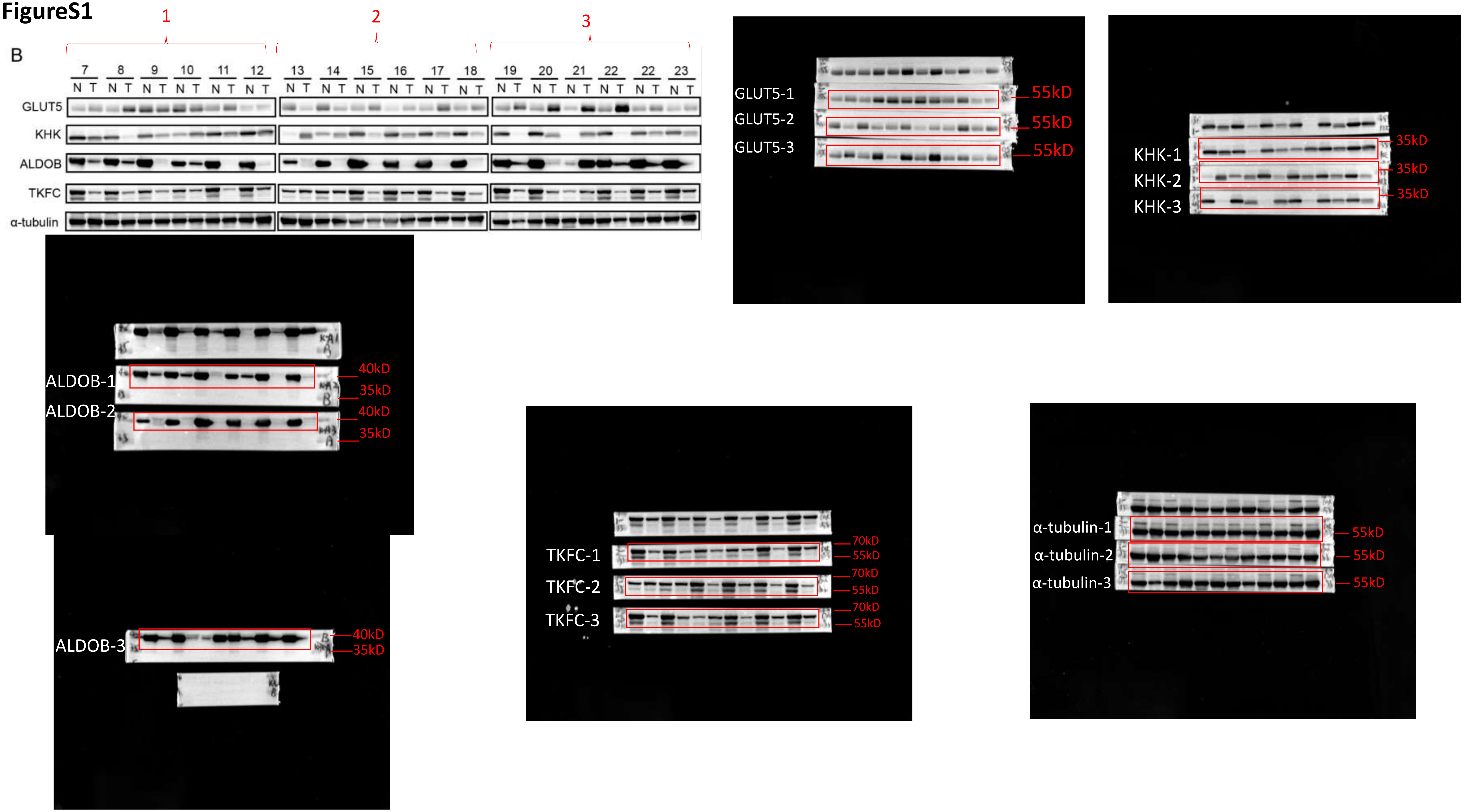
1
2
3
B
7 8 9 10 11 12 13 14 15 16 17 18 19 20 21 22 22 23
N T
GLUT5
KHK
ALDOB
TKFC
α-tubulin
GLUT5-1
GLUT5-2
GLUT5-3
55kD
KHK-1
KHK-2
KHK-3
35kD
ALDOB-1
ALDOB-2
ALDOB-3
40kD
35kD
TKFC-1
TKFC-2
TKFC-3
70kD
55kD
α-tubulin-1
α-tubulin-2
α-tubulin-3
55kD

FigureS3

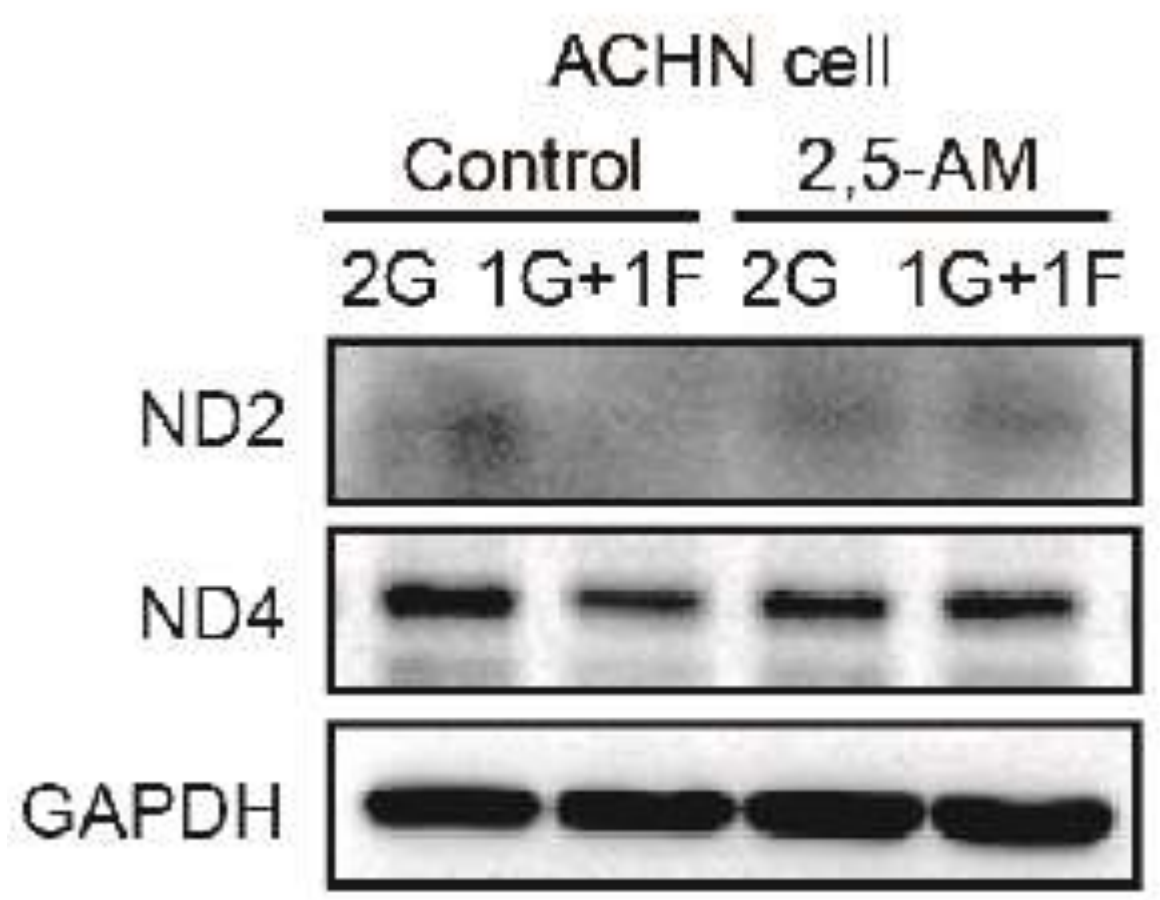
ACHN cell
Control
2,5-AM
2G
1G+1F
2G
1G+1F
ND2
ND4
GAPDH

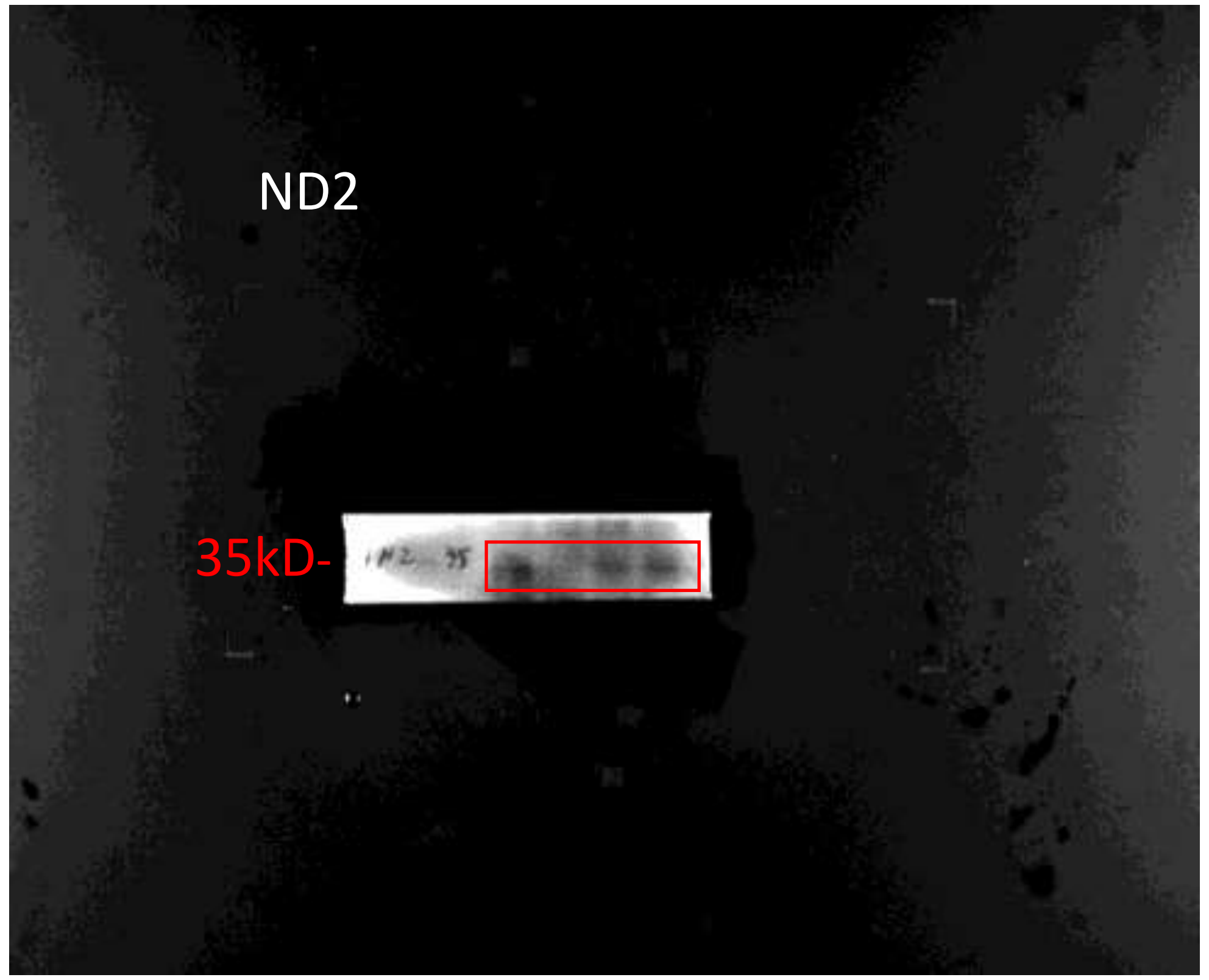
ND2
35kD-

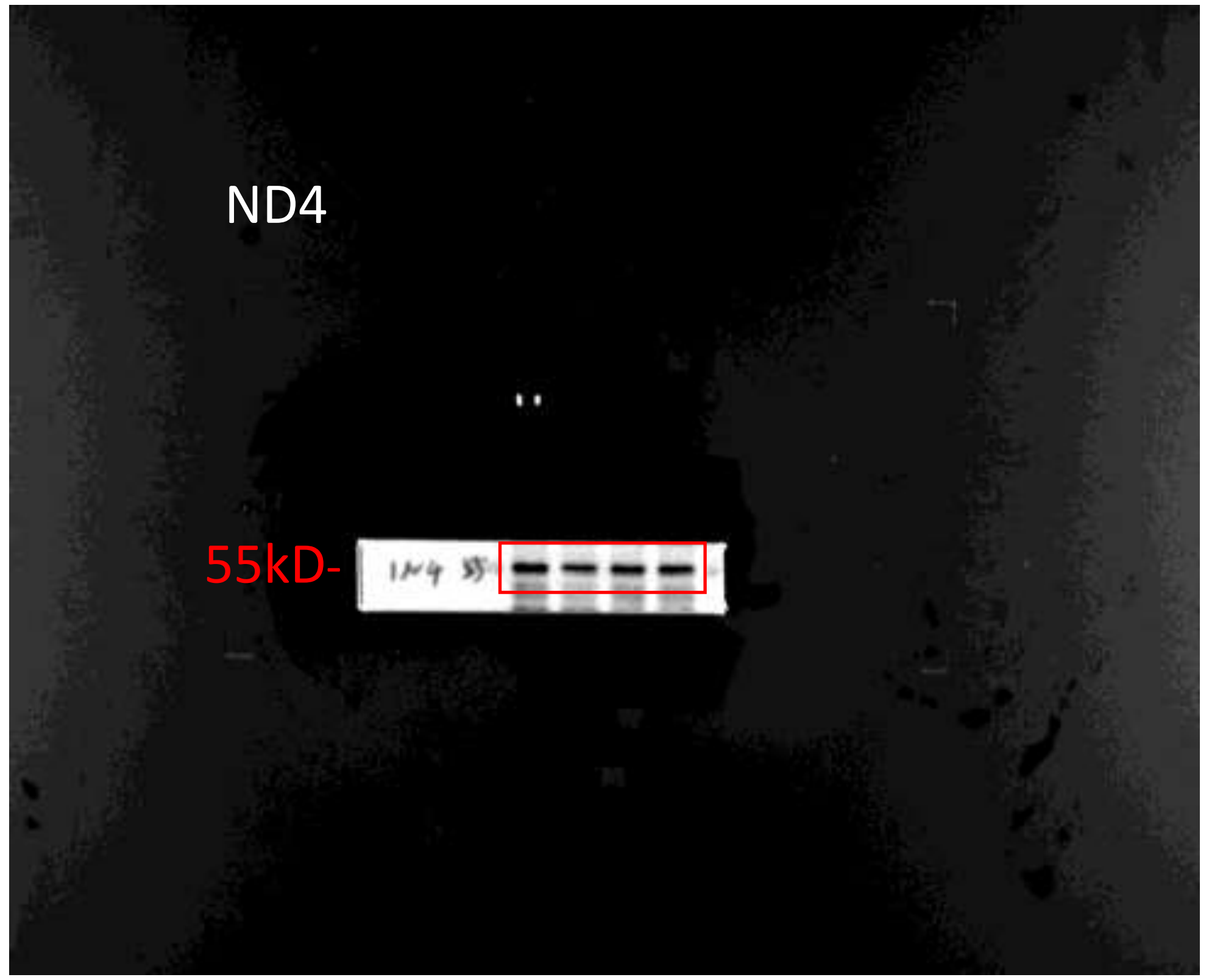
ND4
55kD-

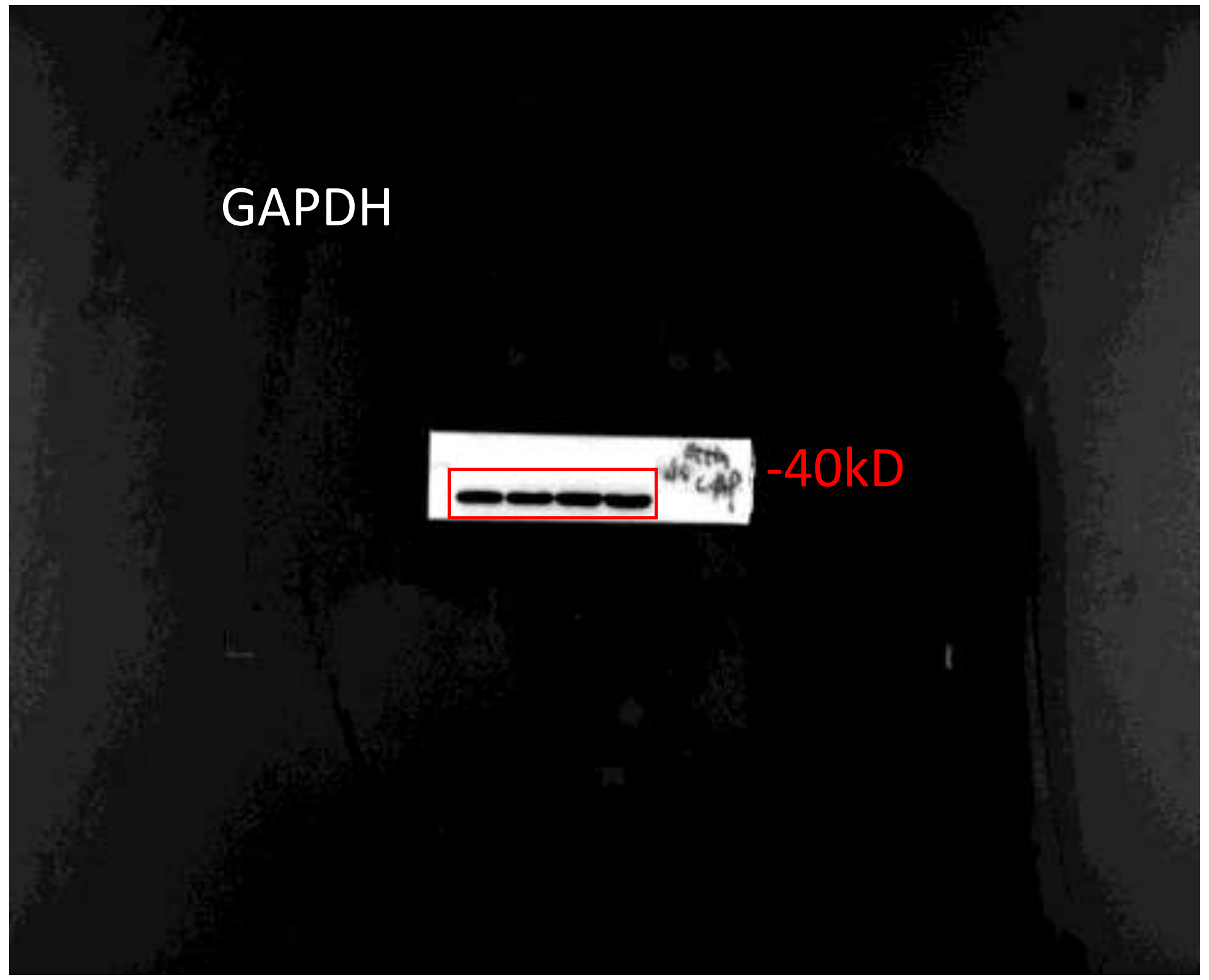
GAPDH
-40kD

D

FigureS4

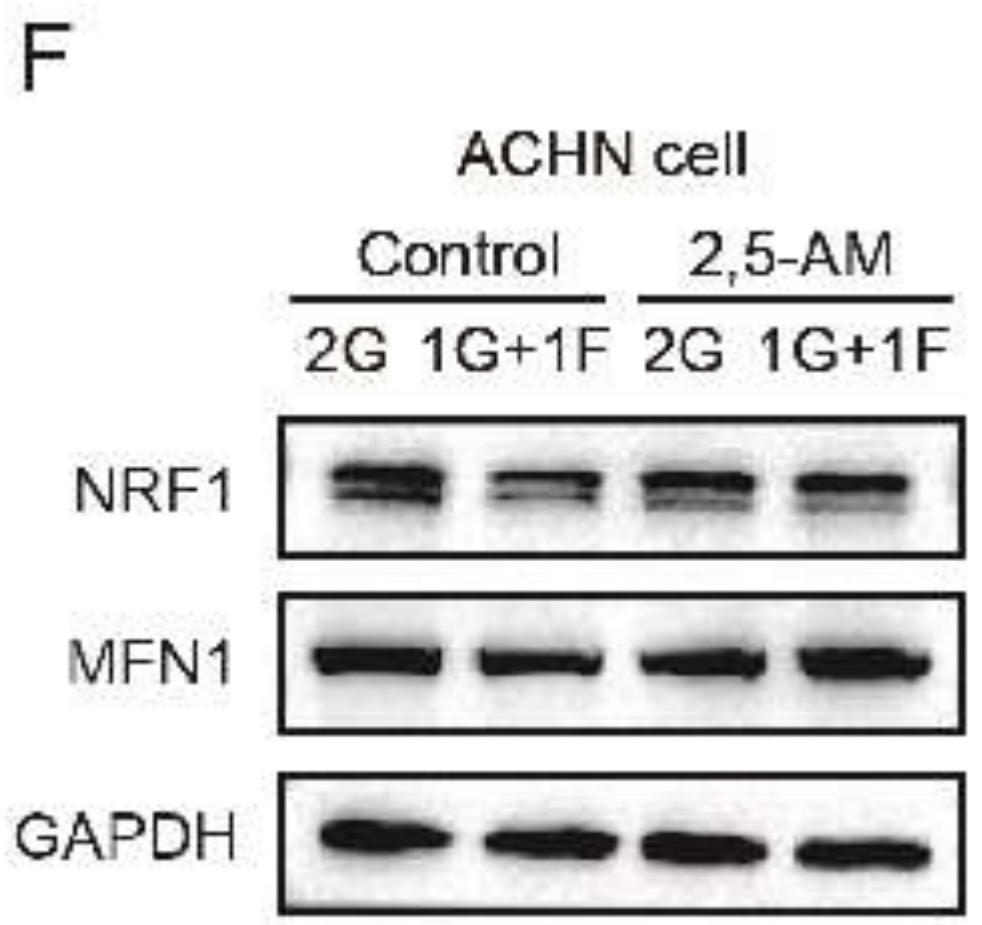
F
ACHN cell
Control
2,5-AM
2G 1G+1F 2G 1G+1F
NRF1
MFN1
GAPDH

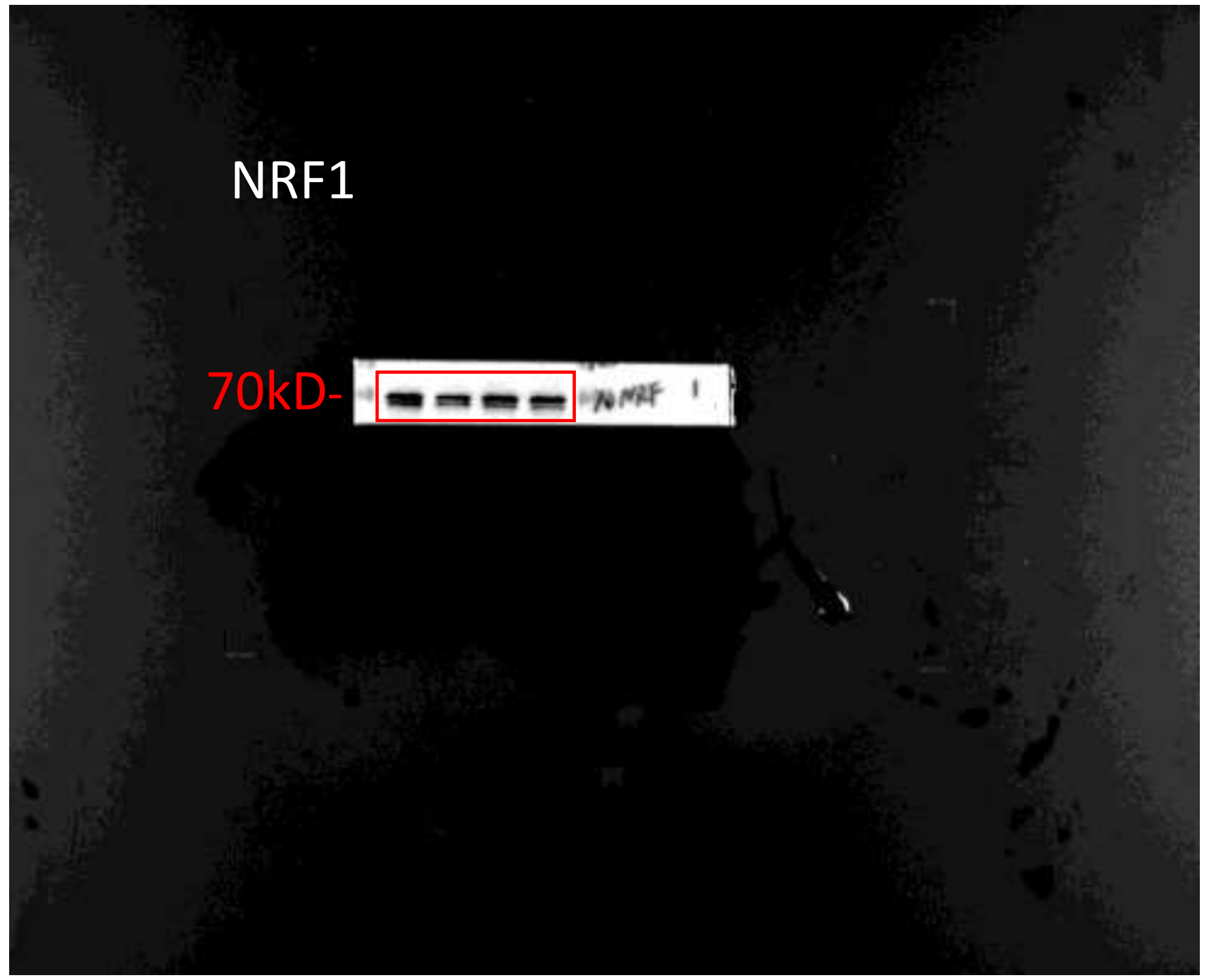
NRF1
70kD-

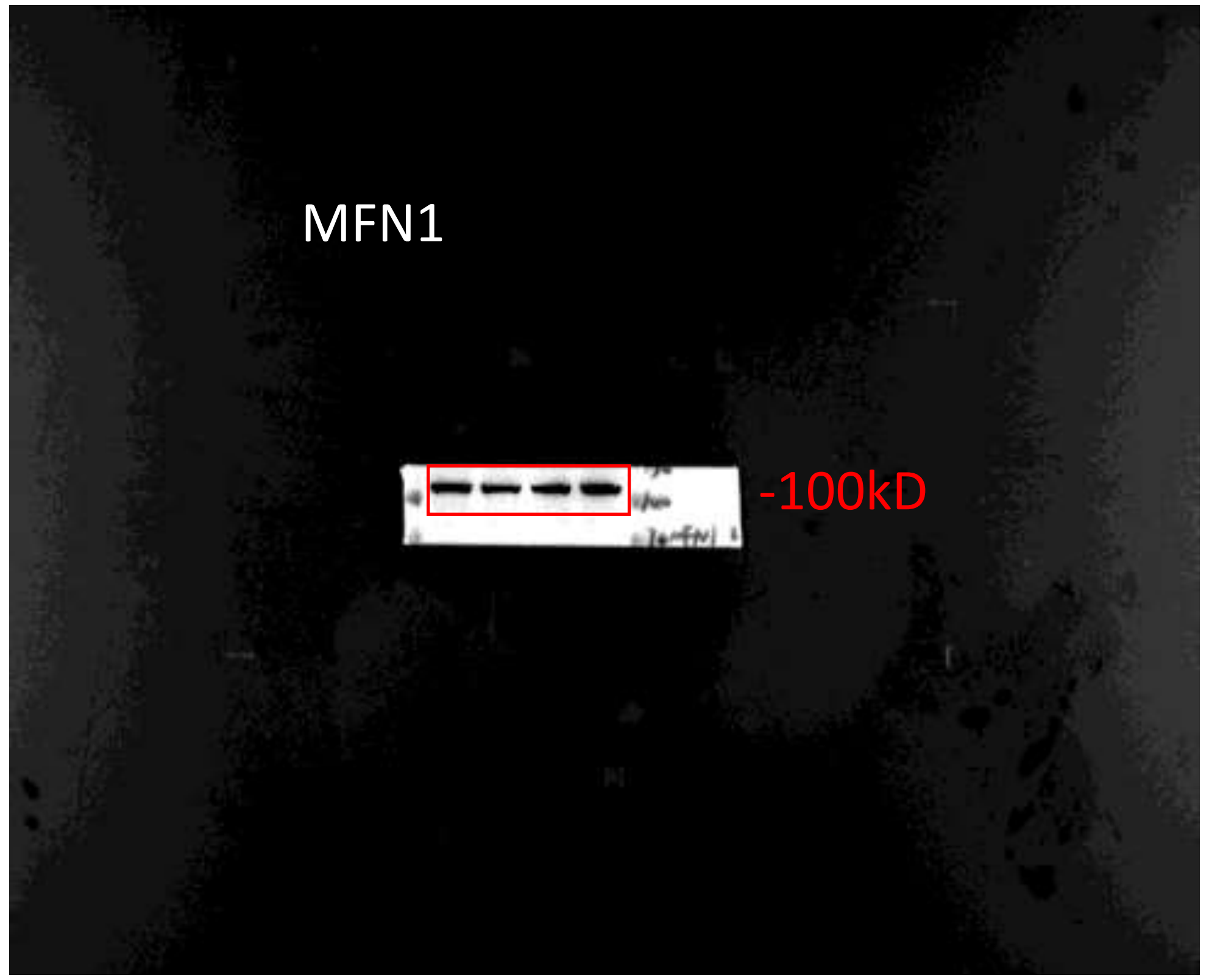
MFN1
-100kD

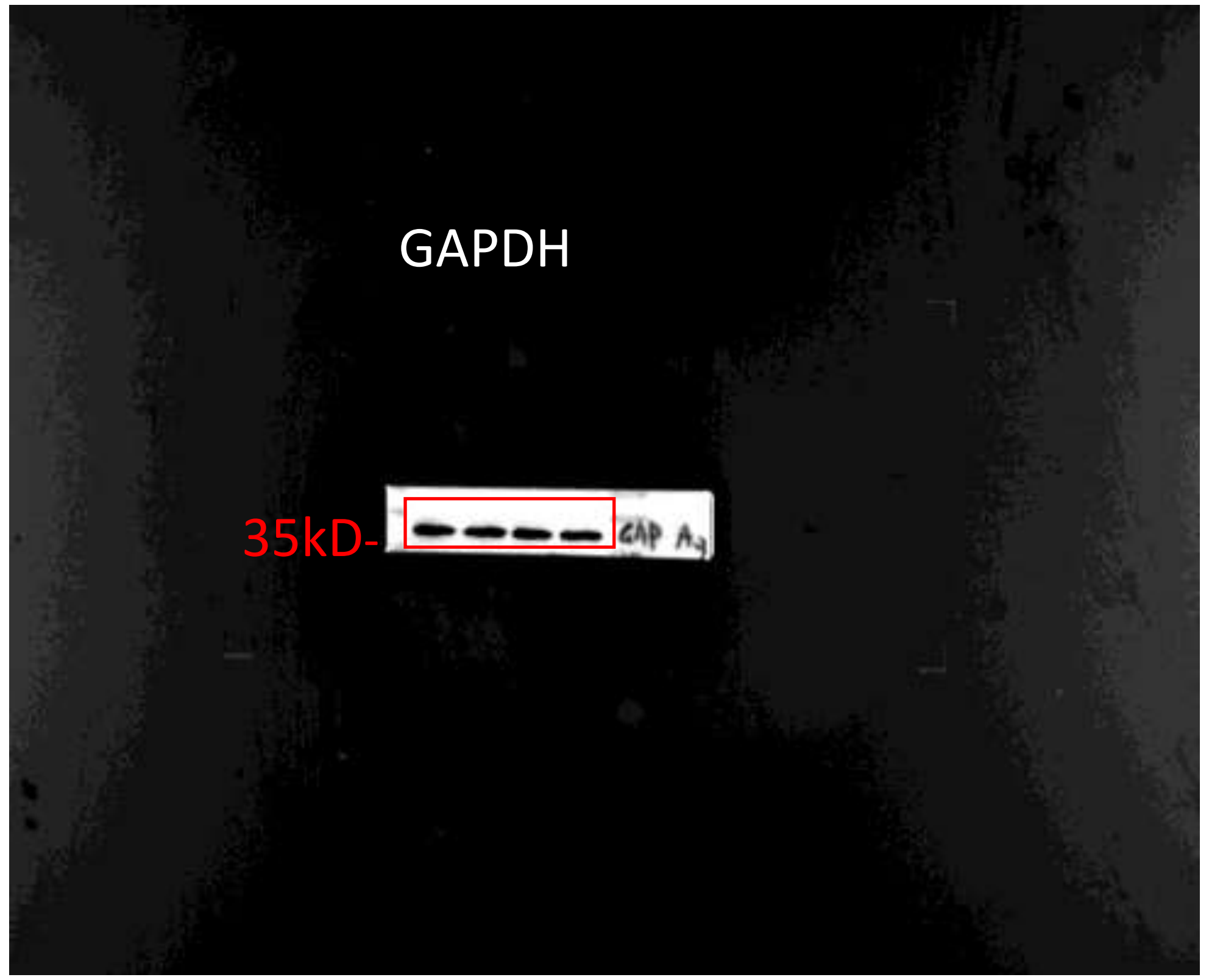
GAPDH
35kD-

FigureS4

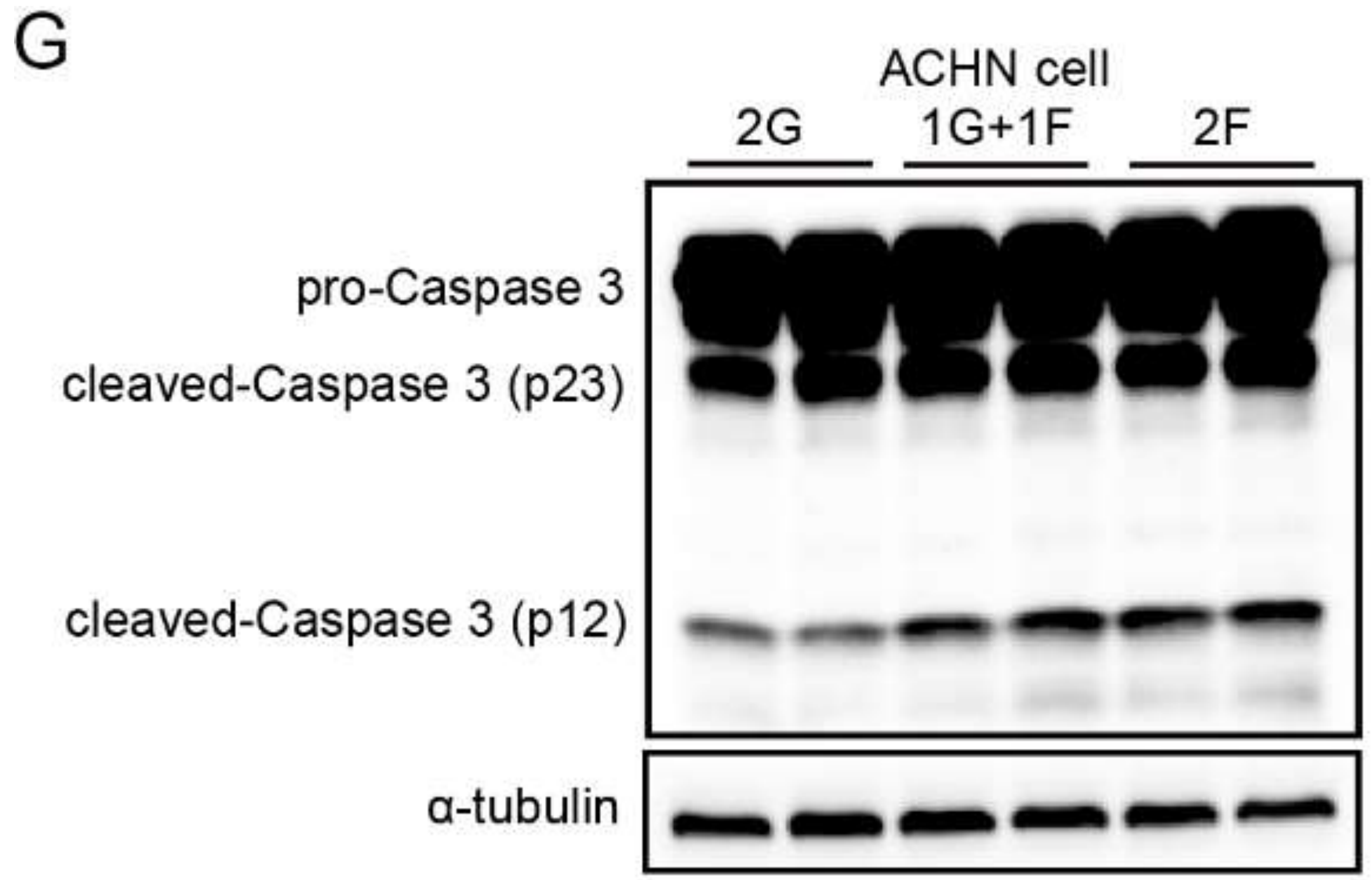
G
ACHN cell
2G
1G+1F
2F
pro-Caspase 3
cleaved-Caspase 3 (p23)
cleaved-Caspase 3 (p12)
α-tubulin

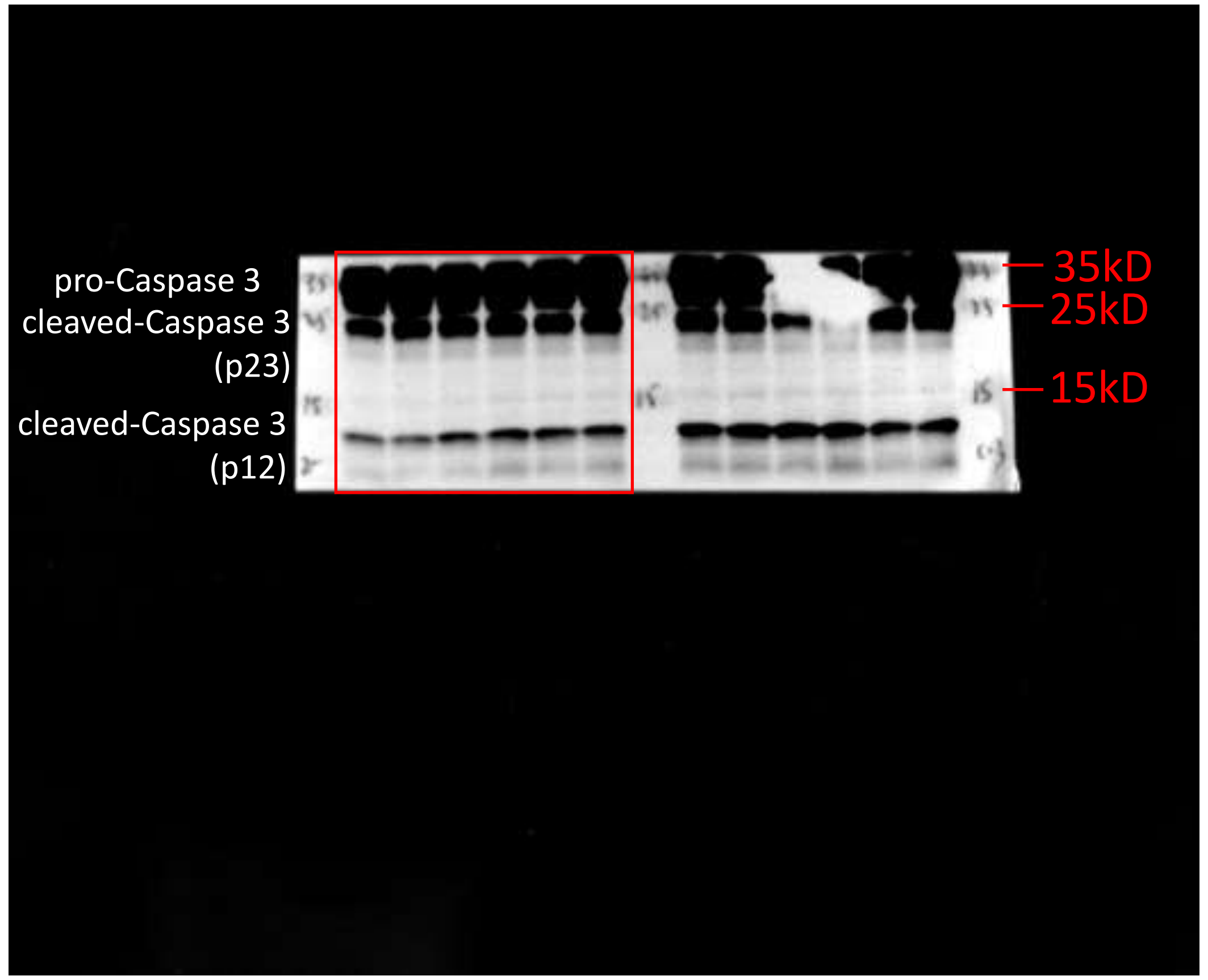
pro-Caspase 3
cleaved-Caspase 3
(p23)
cleaved-Caspase 3
(p12)
35kD
25kD
15kD

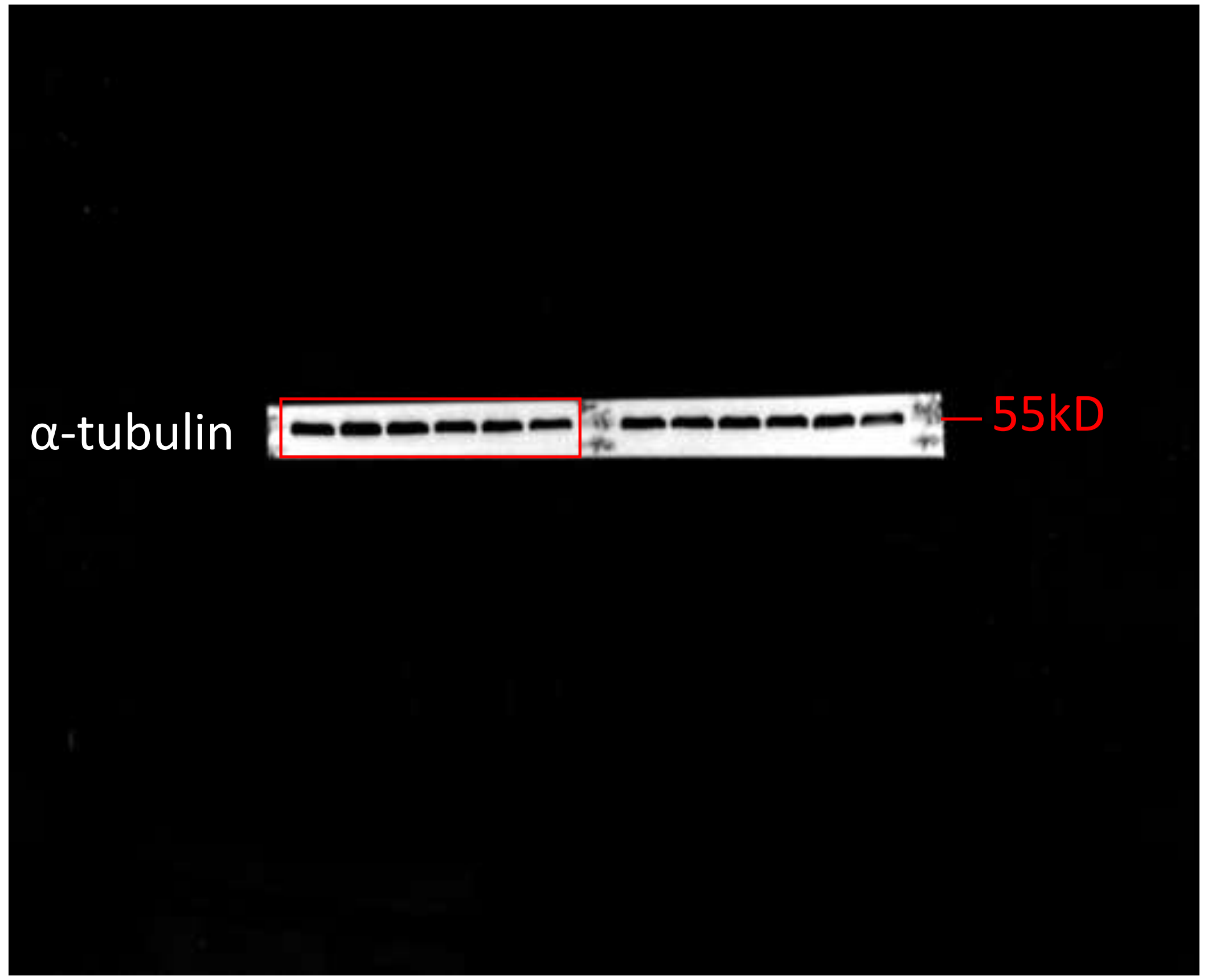
α-tubulin
55kD

FigureS5

A

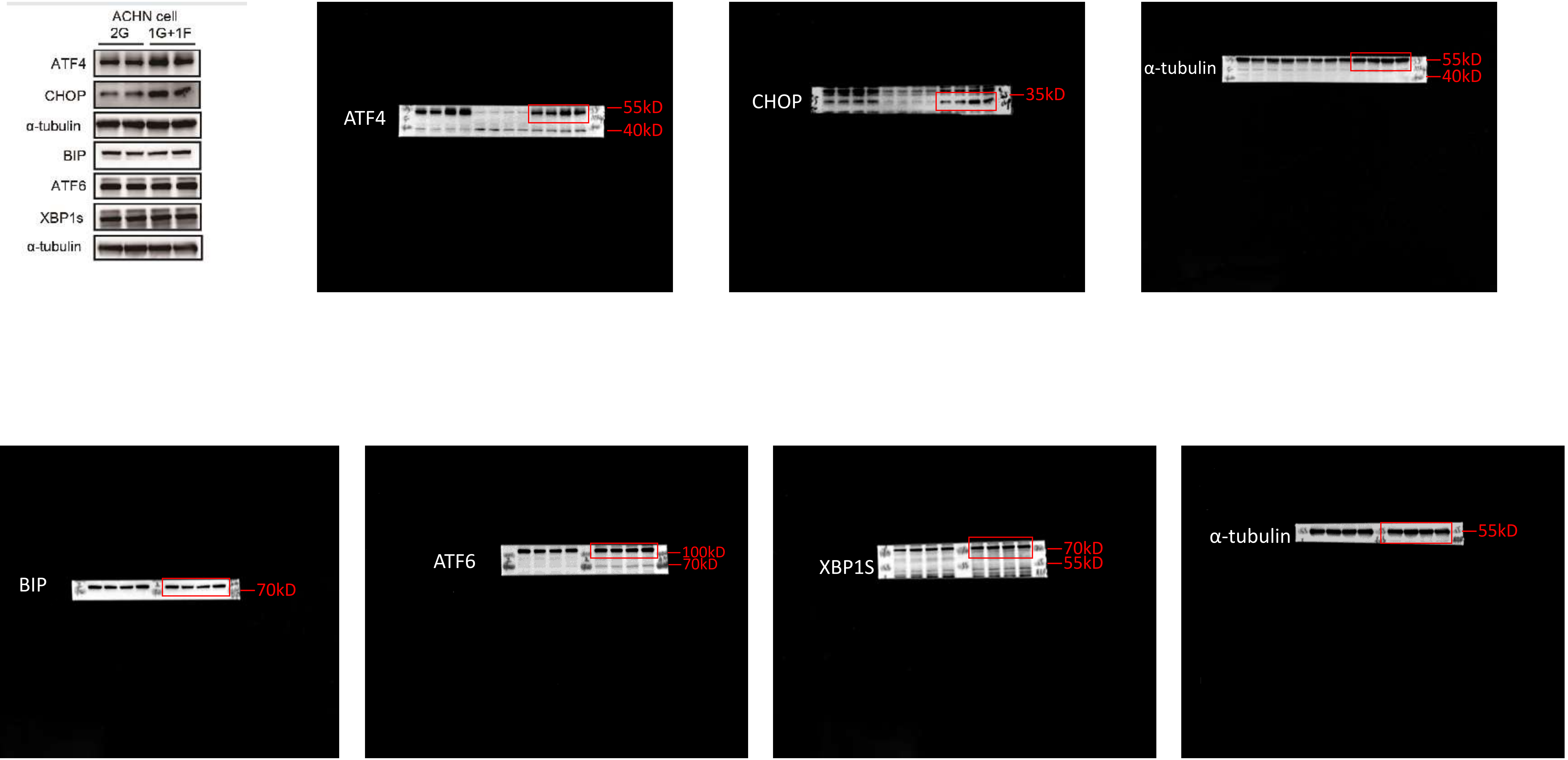
ACHN cell
2G
1G+1F
ATF4
CHOP
α-tubulin
BIP
ATF6
XBP1s
α-tubulin
ATF4
55kD
40kD
CHOP
35kD
α-tubulin
55kD
40kD
BIP
70kD
ATF6
100kD
70kD
XBP1S
70kD
55kD
α-tubulin
55kD

FigureS5

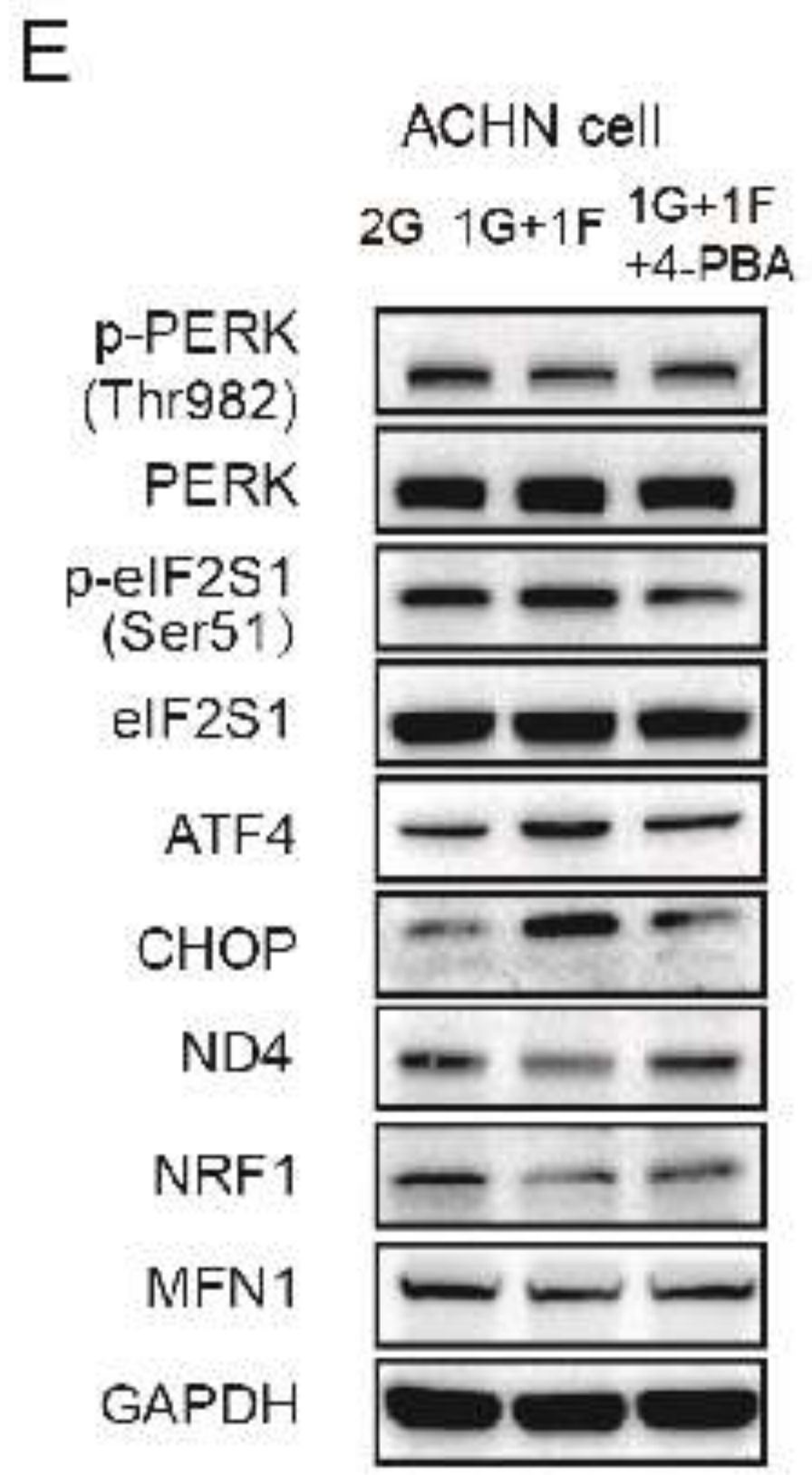
E
ACHN cell
2G
1G+1F
1G+1F +4-PBA
p-PERK (Thr982)
PERK
p-eIF2S1 (Ser51)
eIF2S1
ATF4
CHOP
ND4
NRF1
MFN1
GAPDH

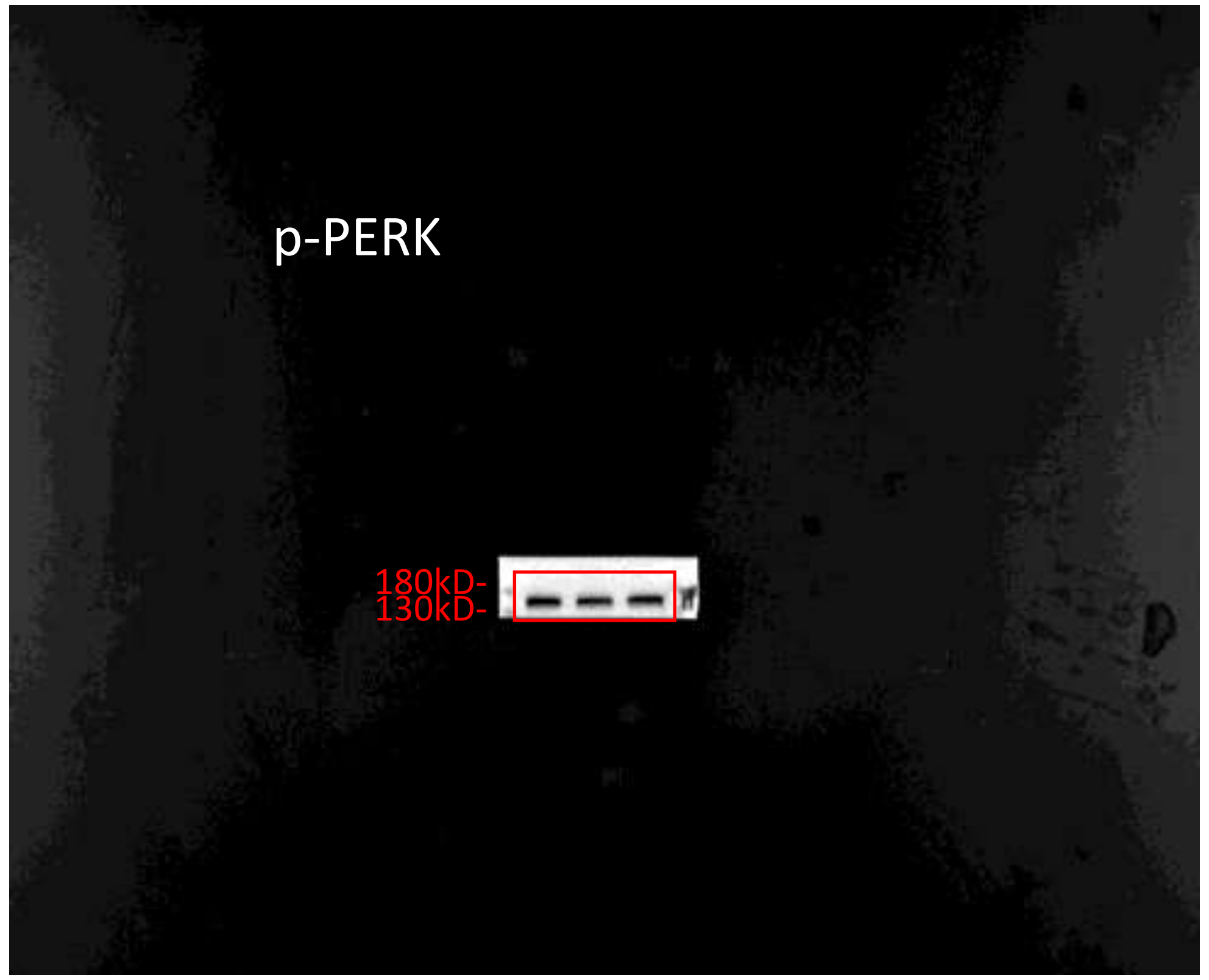
p-PERK
180kD-
130kD-

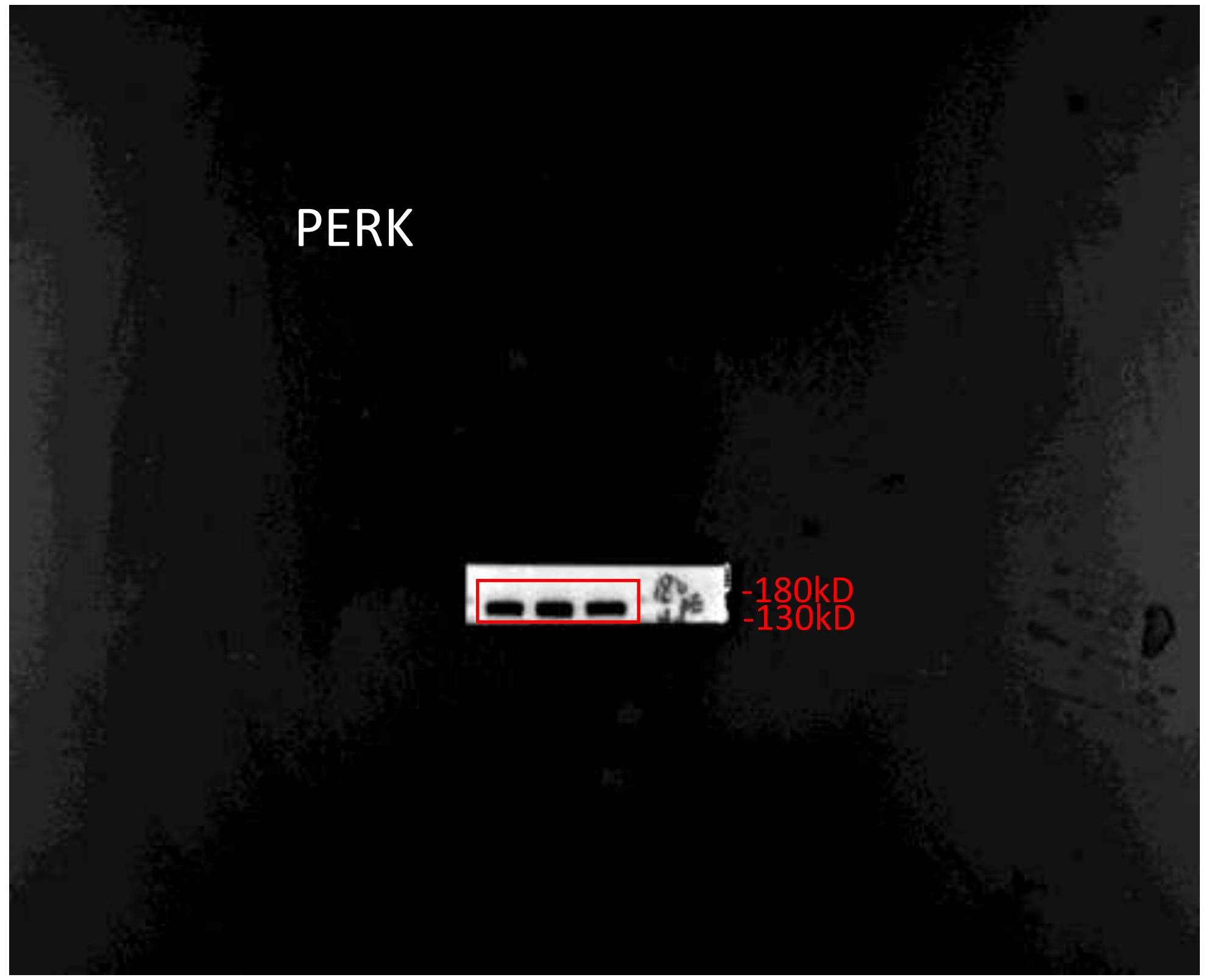
PERK
-180kD
-130kD

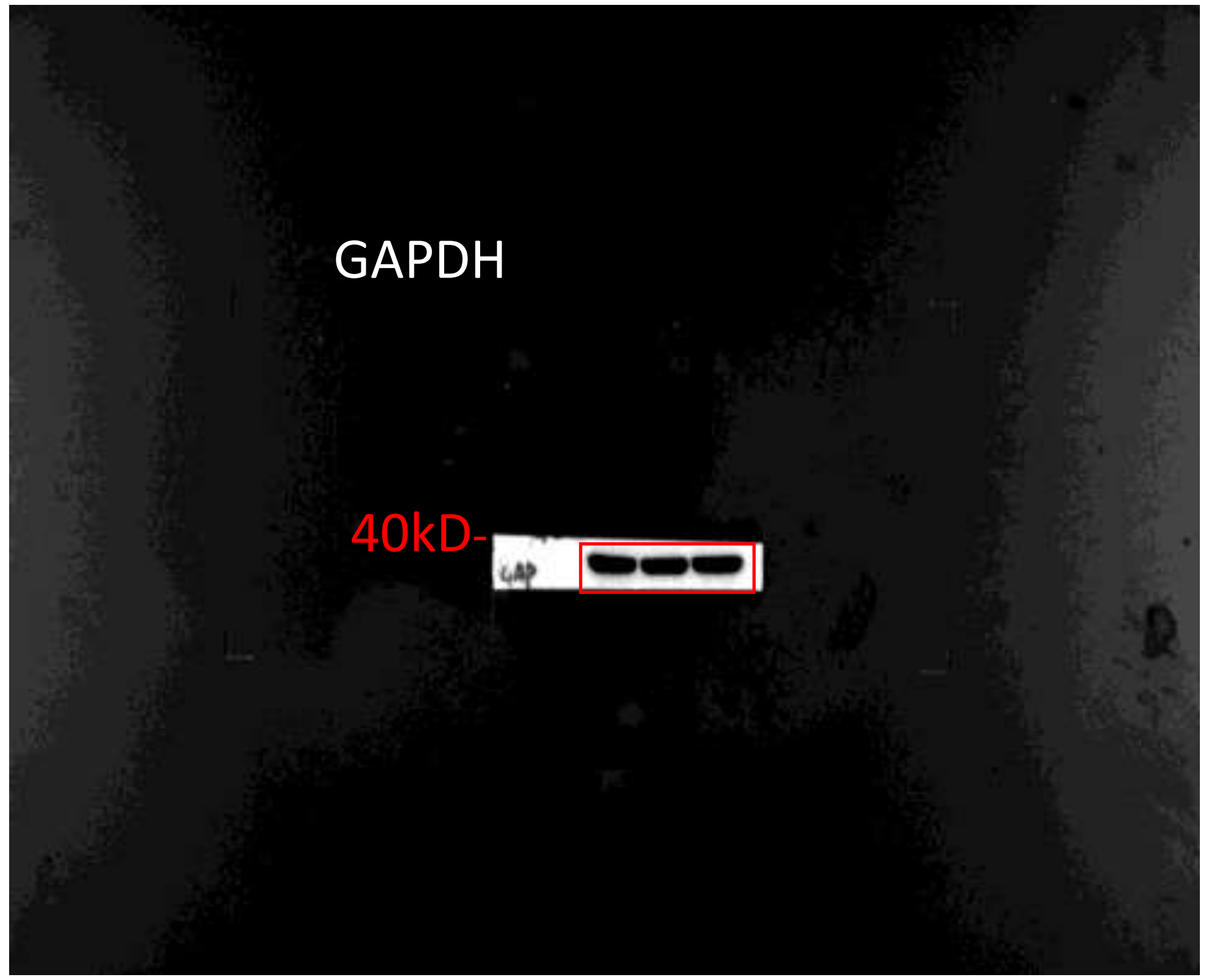
GAPDH
40kD-

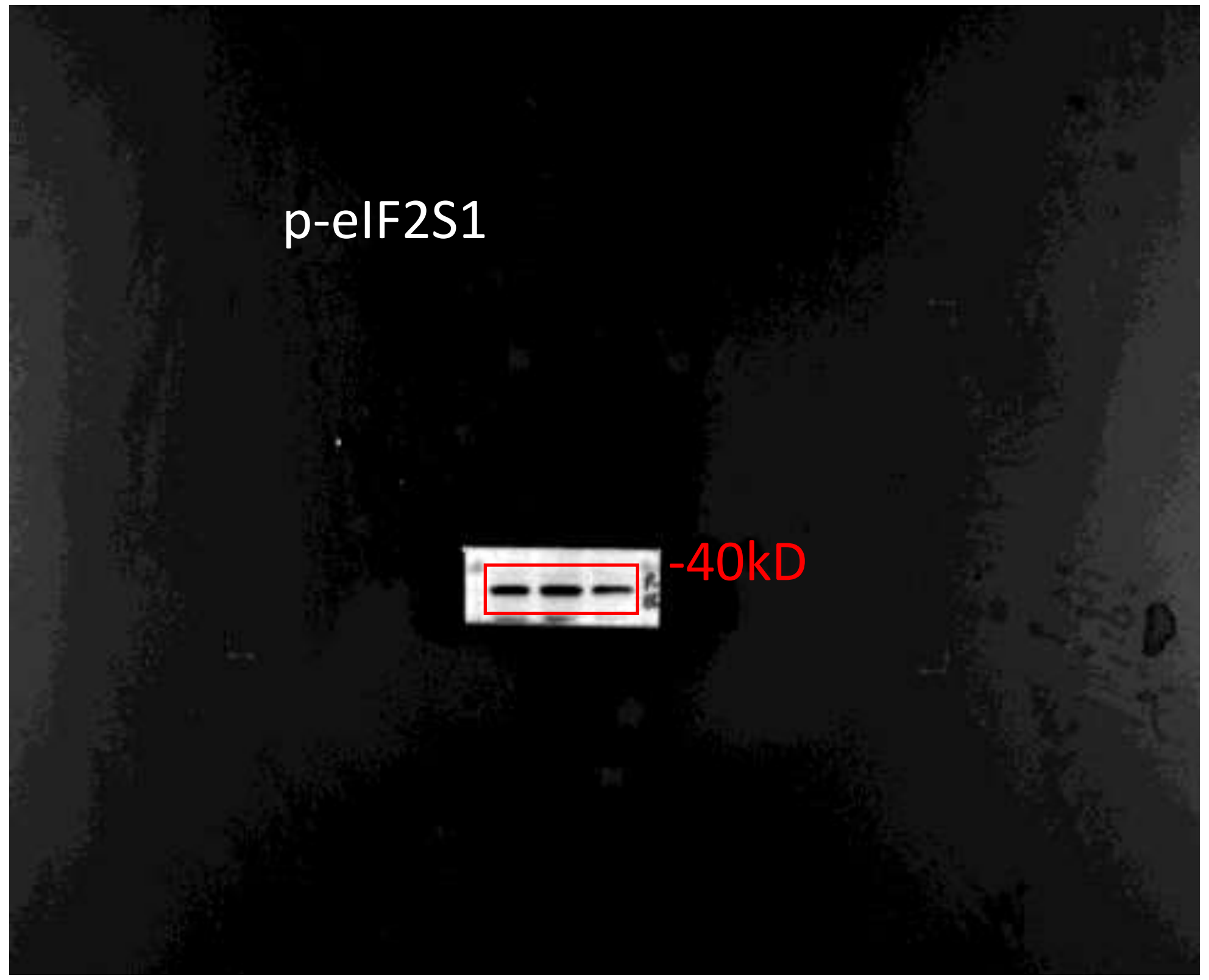
p-eIF2S1
-40kD

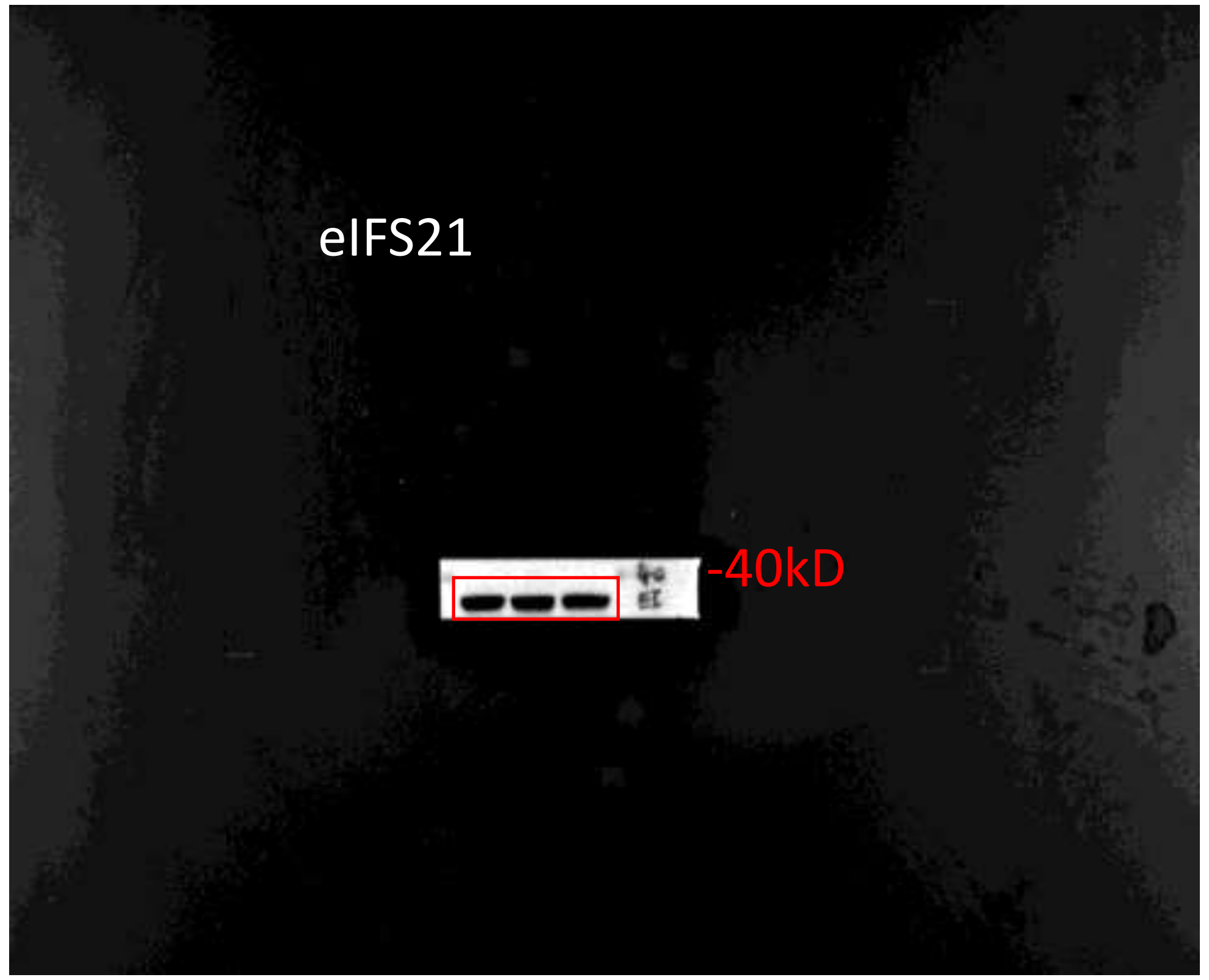
eIFS21
-40kD

FigureS5

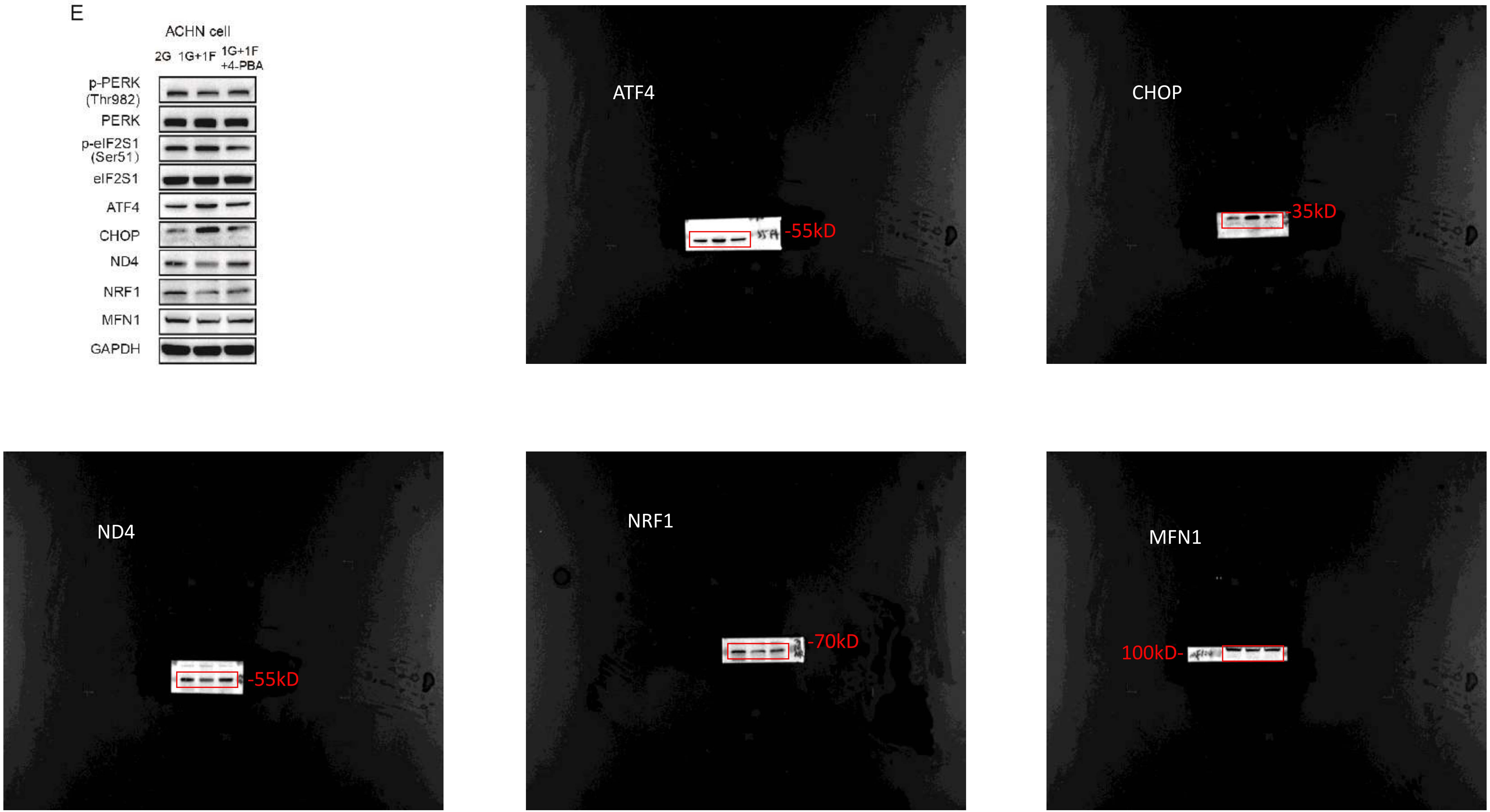
E
ACHN cell
2G
1G+1F
1G+1F +4-PBA
p-PERK (Thr982)
PERK
p-eIF2S1 (Ser51)
eIF2S1
ATF4
CHOP
ND4
NRF1
MFN1
GAPDH
ATF4
-55kD
CHOP
-35kD
ND4
-55kD
NRF1
-70kD
MFN1
100kD-

FigureS5

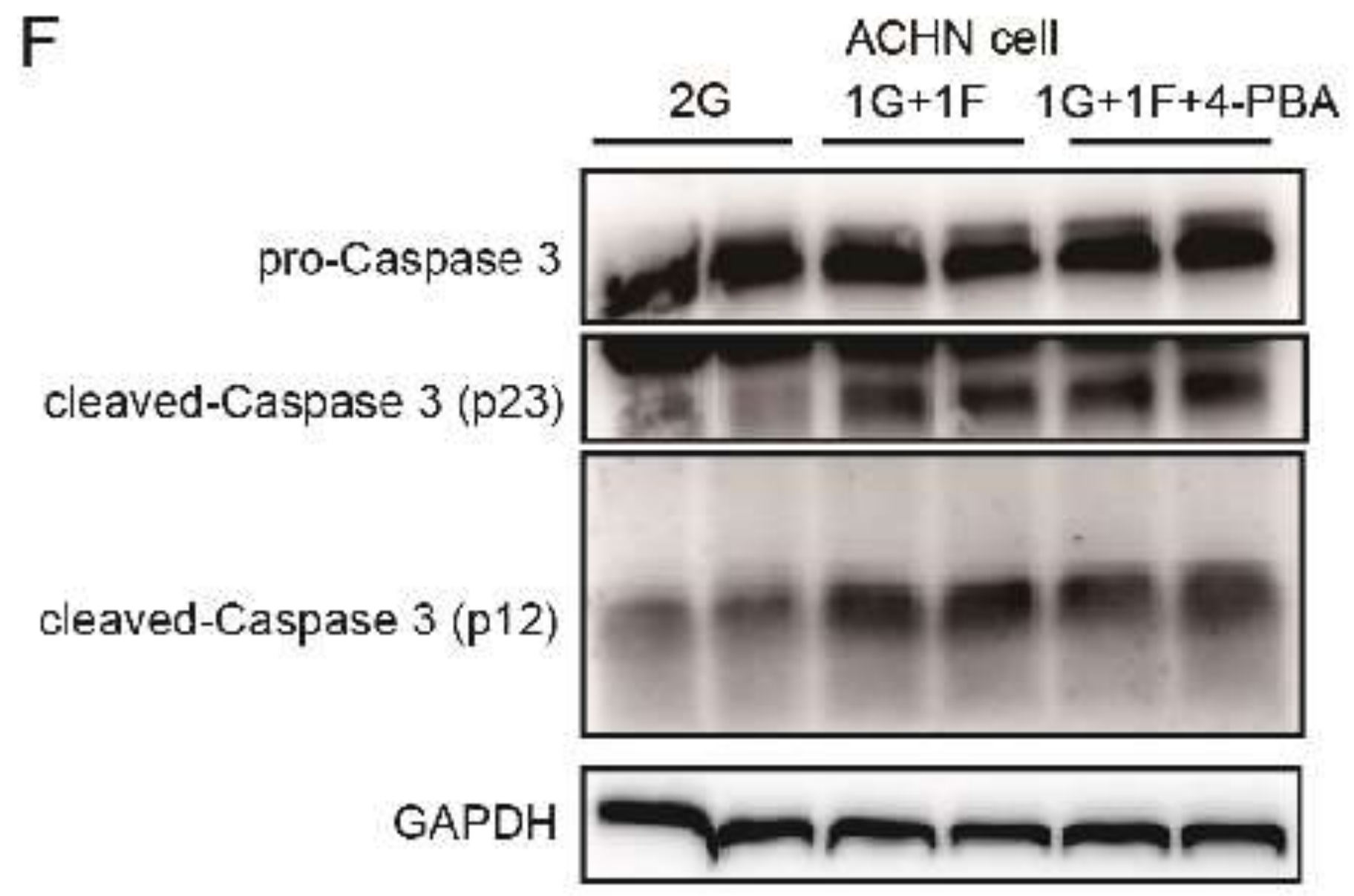

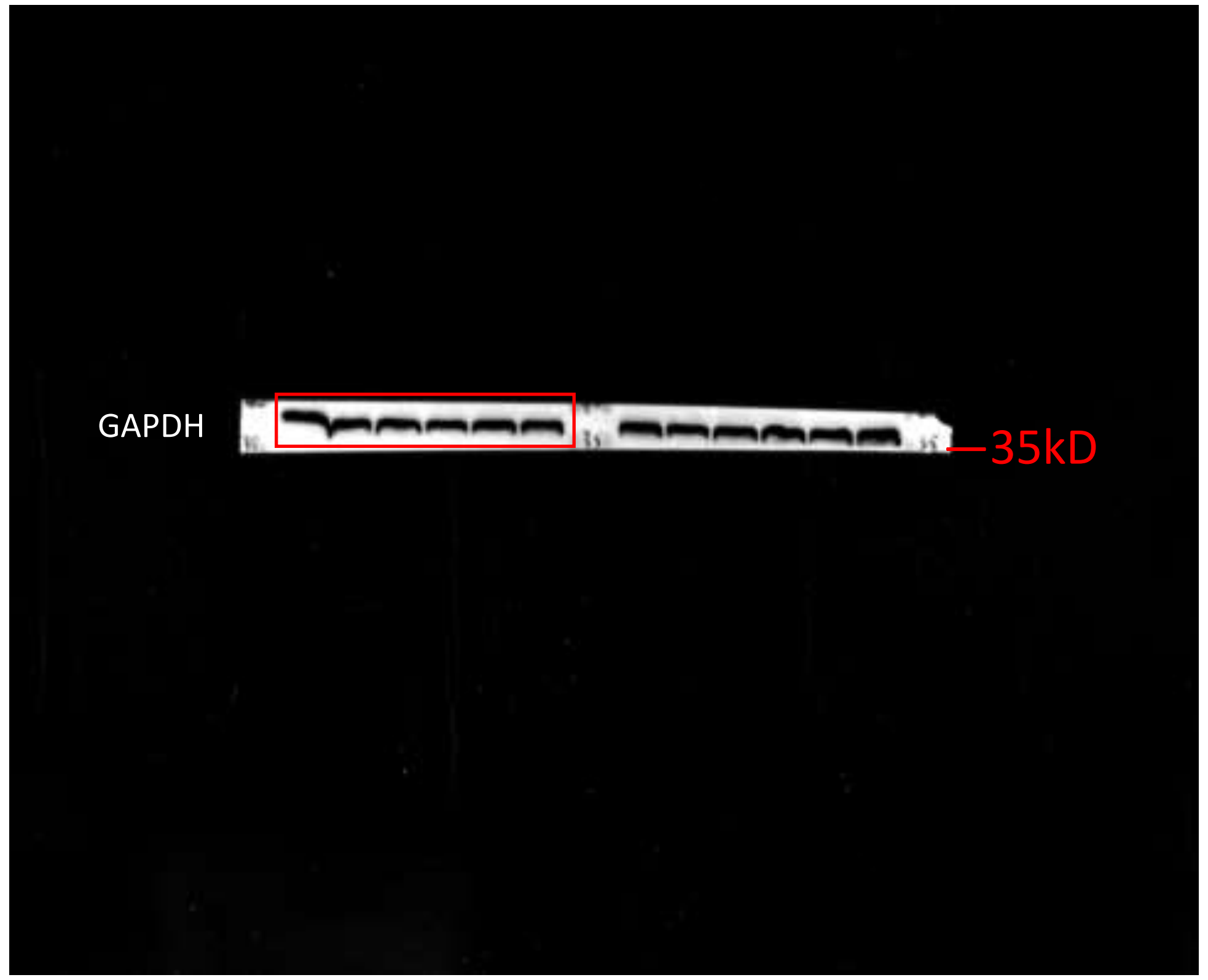

Short exposure

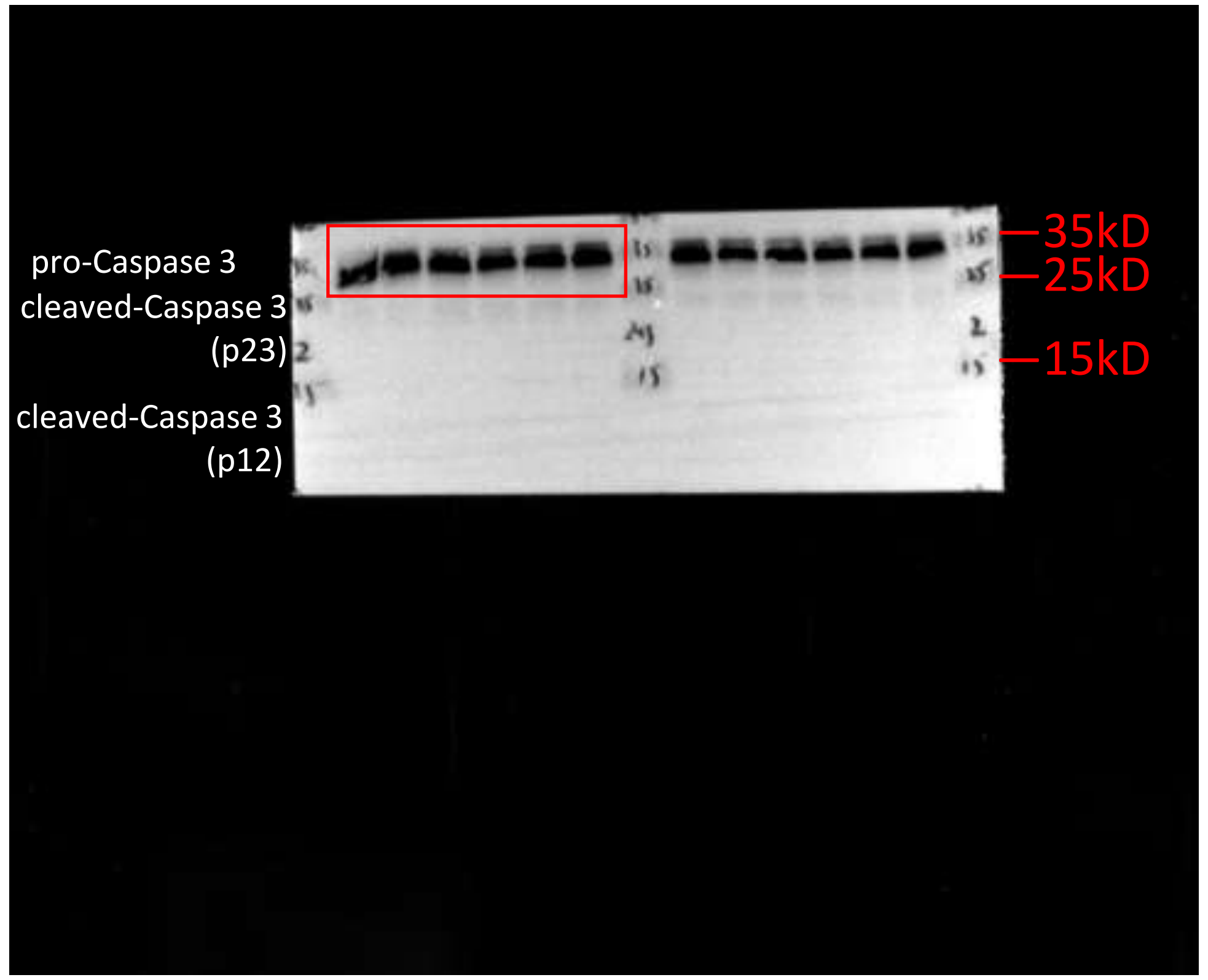

Middle exposure

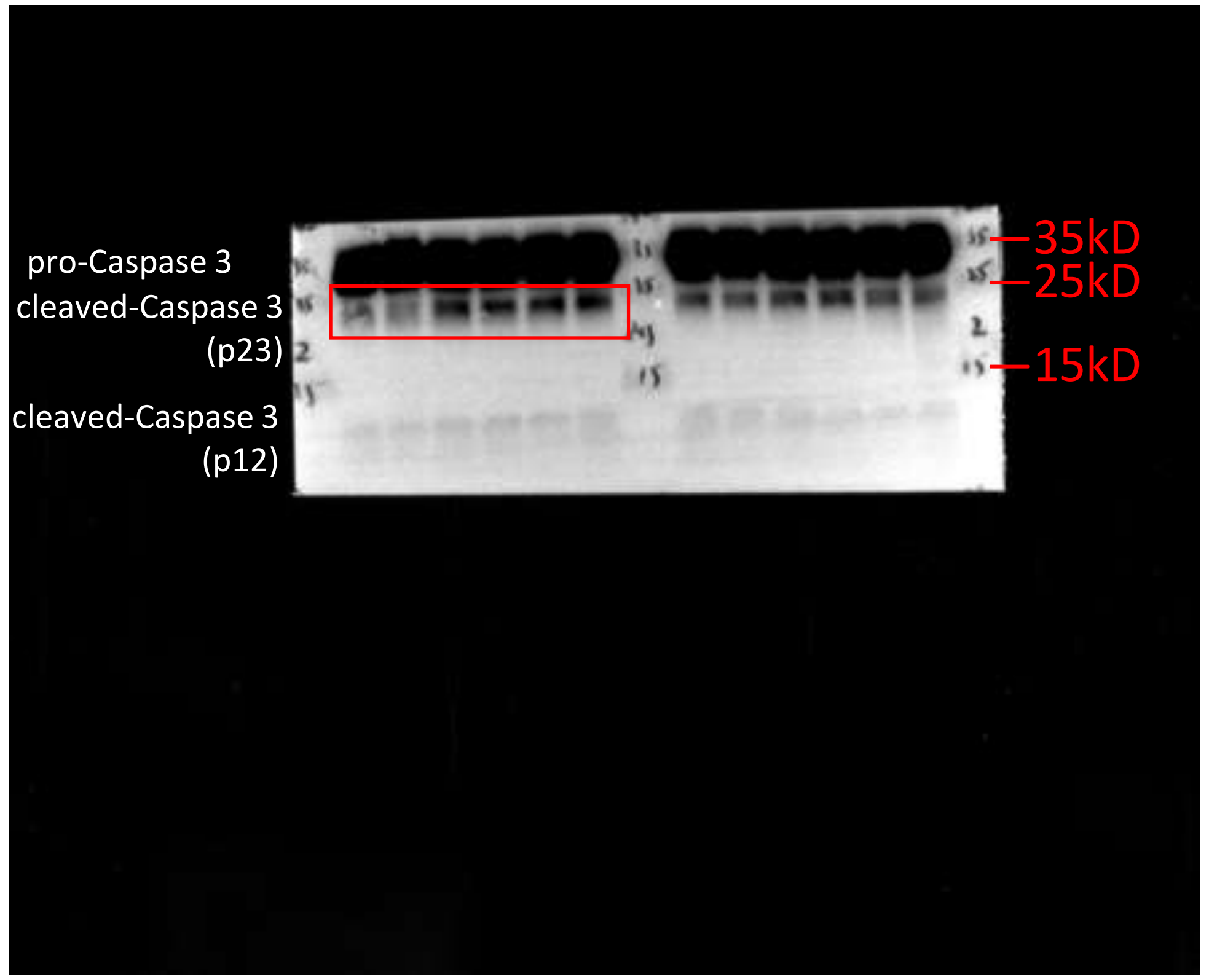

Long exposure

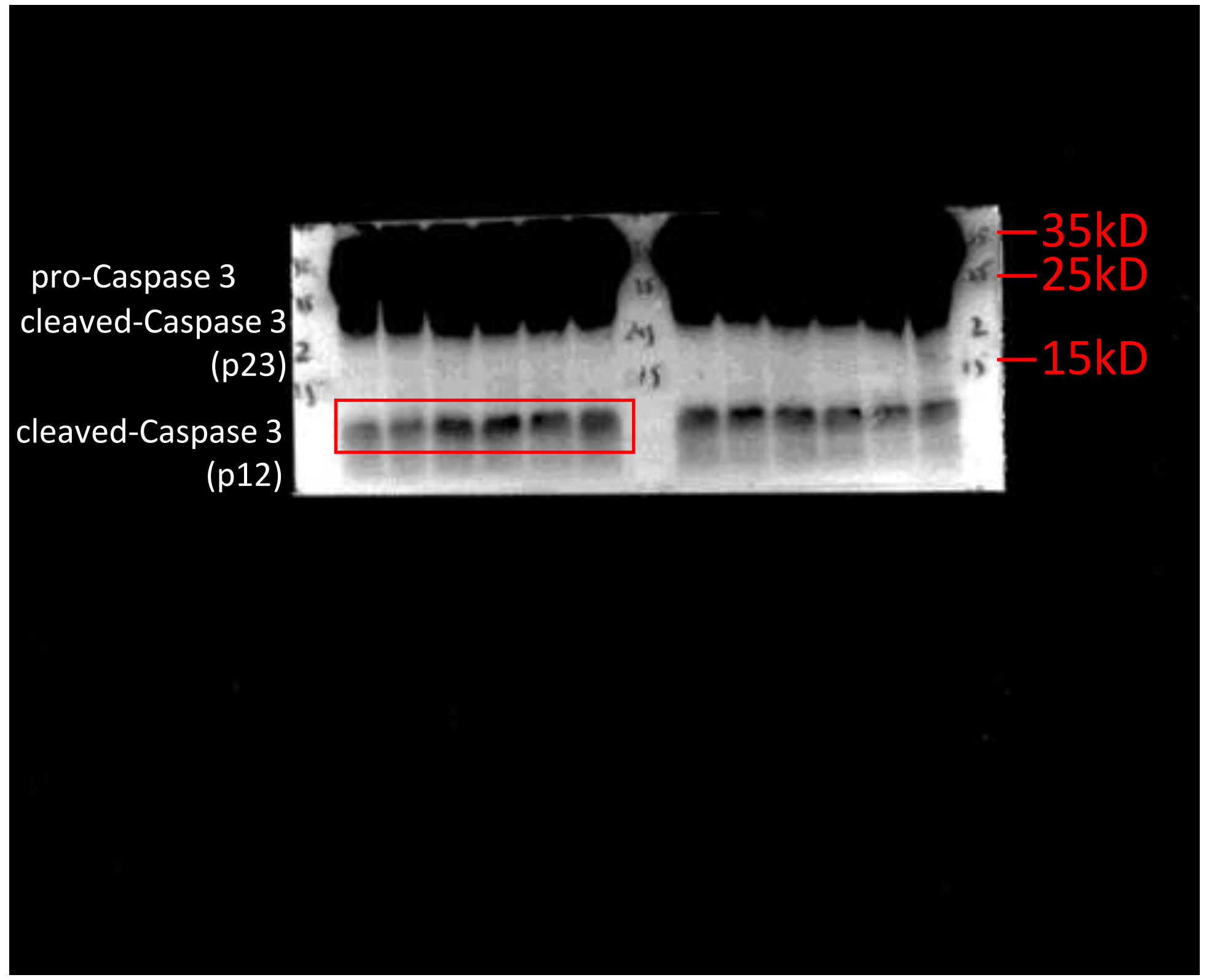

FigureS6

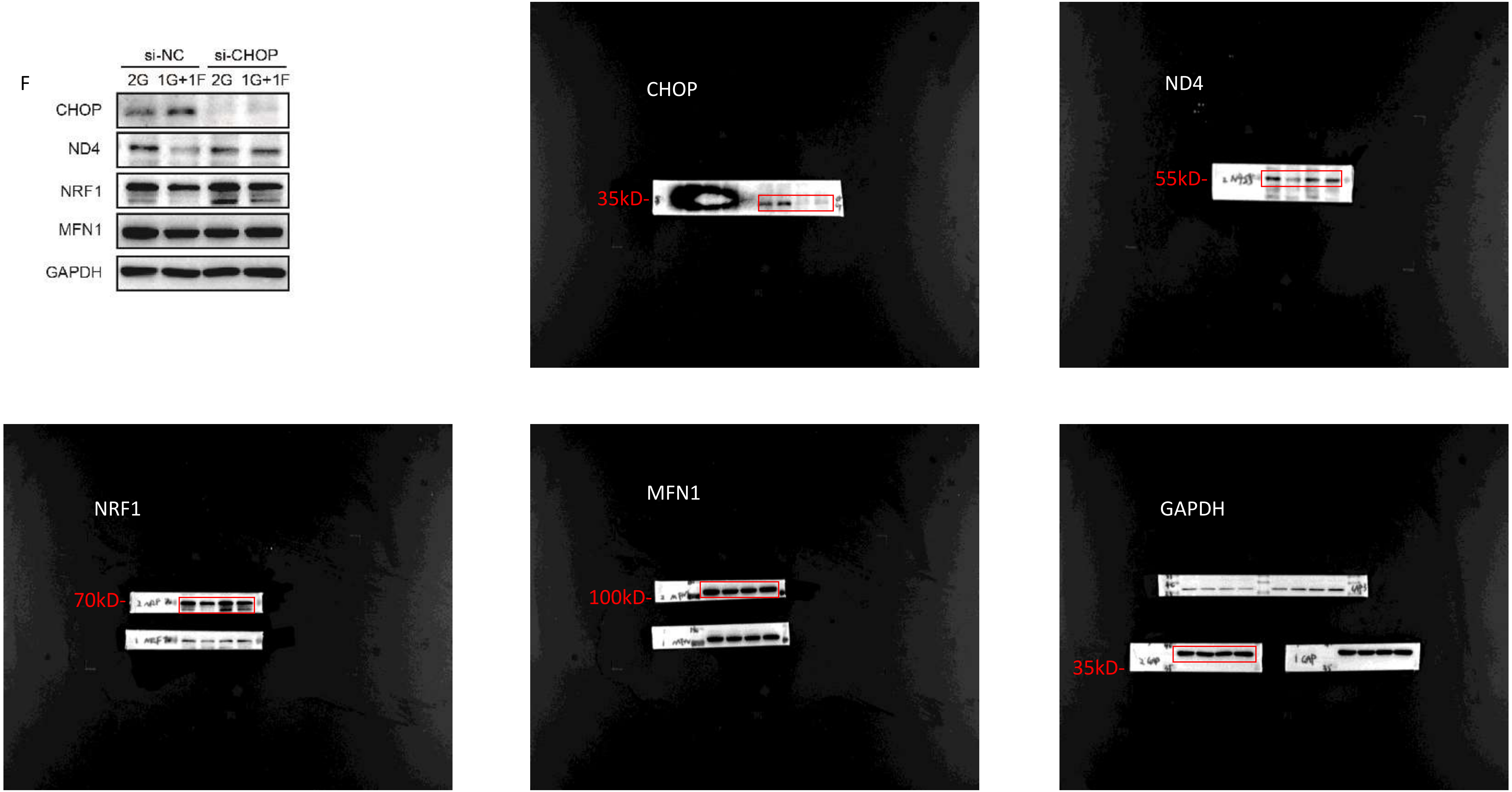
F
si-NC
si-CHOP
2G
1G+1F
2G
1G+1F
CHOP
ND4
NRF1
MFN1
GAPDH
CHOP
35kD-
ND4
55kD-
NRF1
70kD-
MFN1
100kD-
GAPDH
35kD-

FigureS8

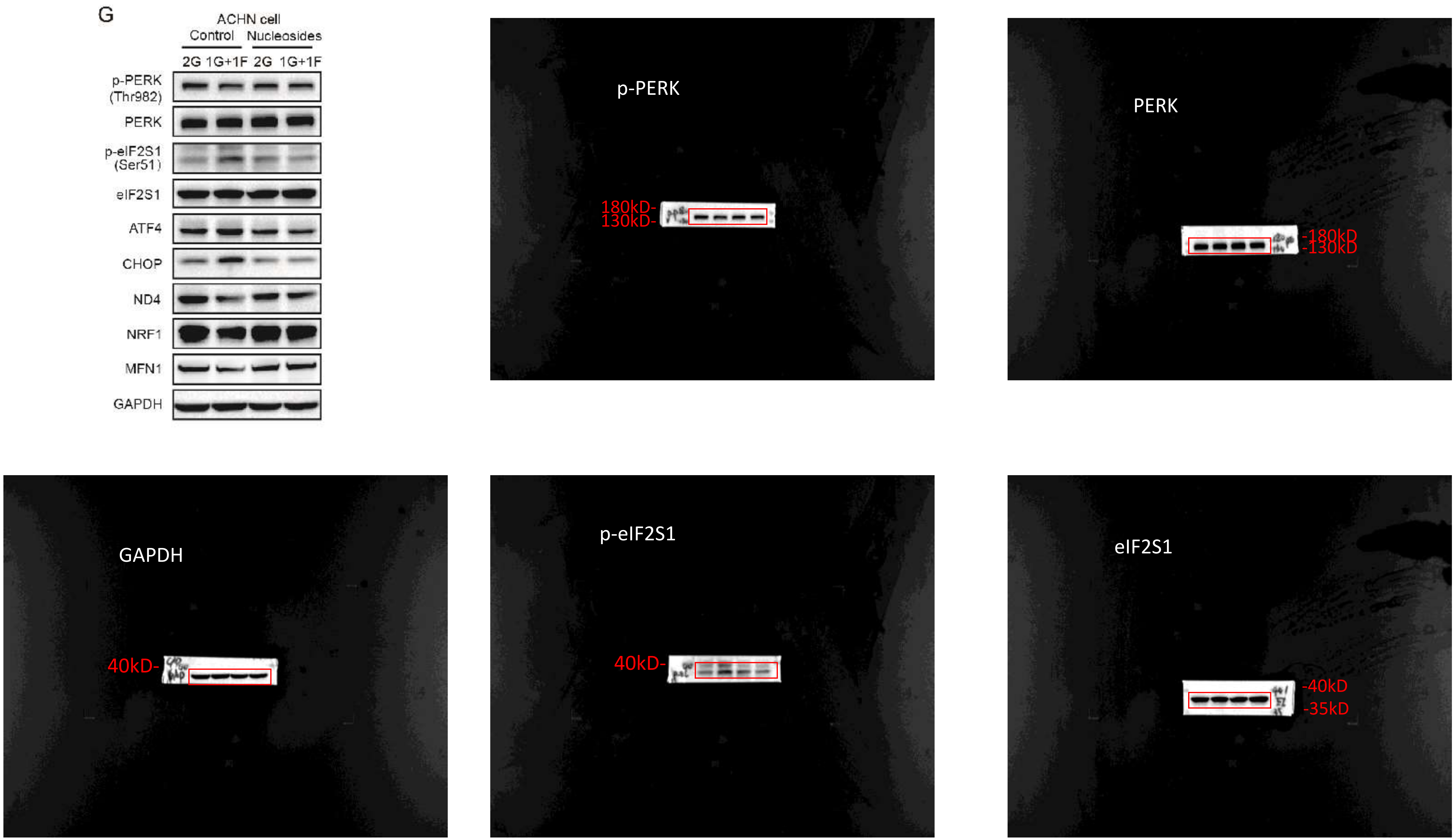
G
ACHN cell
Control
Nucleosides
2G
1G+1F
2G
1G+1F
p-PERK (Thr982)
PERK
p-eIF2S1 (Ser51)
eIF2S1
ATF4
CHOP
ND4
NRF1
MFN1
GAPDH
p-PERK
180kD-
130kD-
PERK
-180kD
-130kD
GAPDH
40kD-
p-eIF2S1
40kD-
eIF2S1
-40kD
-35kD

FigureS8

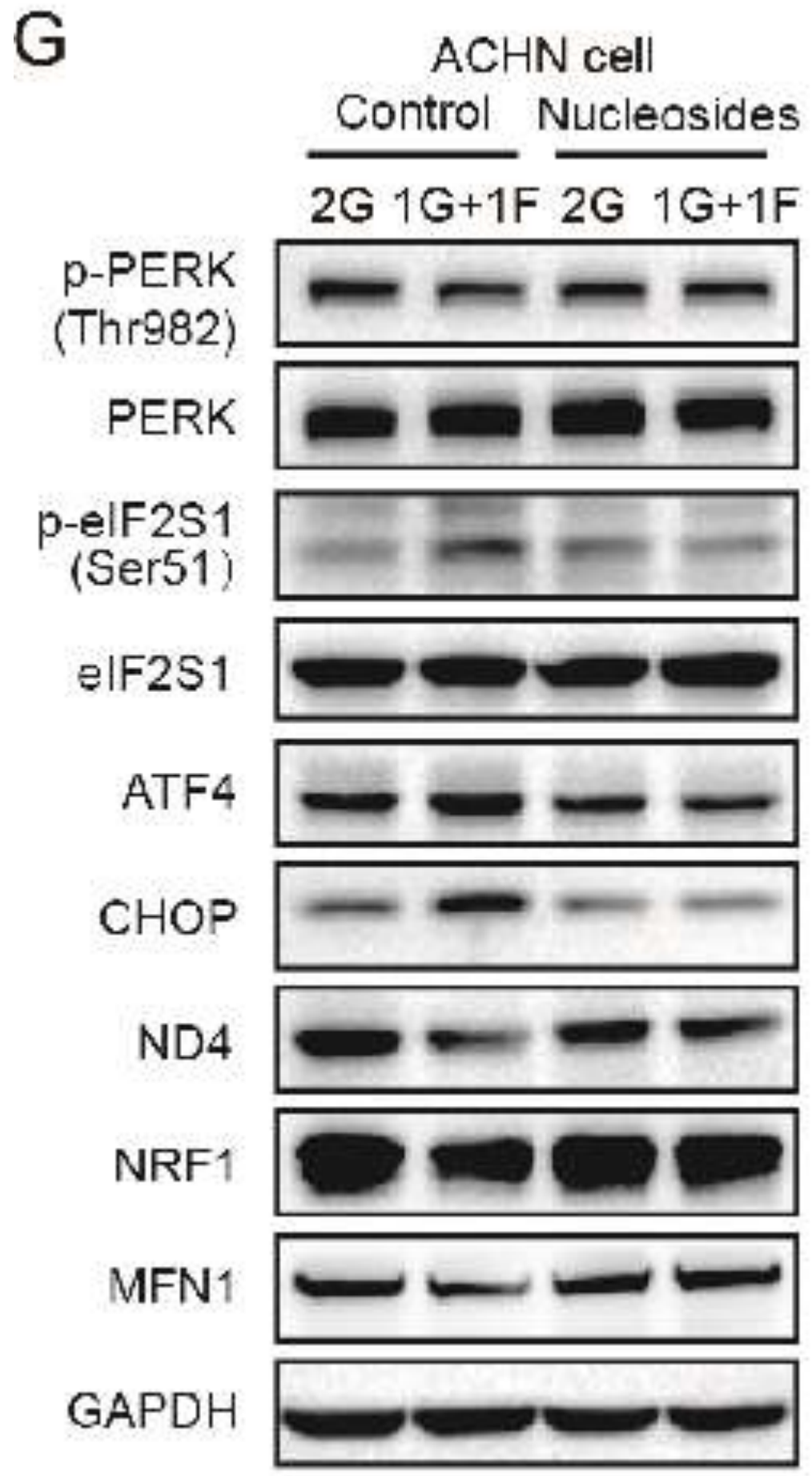
G
ACHN cell
Control
Nucleosides
2G 1G+1F 2G 1G+1F
p-PERK (Thr982)
PERK
p-eIF2S1 (Ser51)
eIF2S1
ATF4
CHOP
ND4
NRF1
MFN1
GAPDH

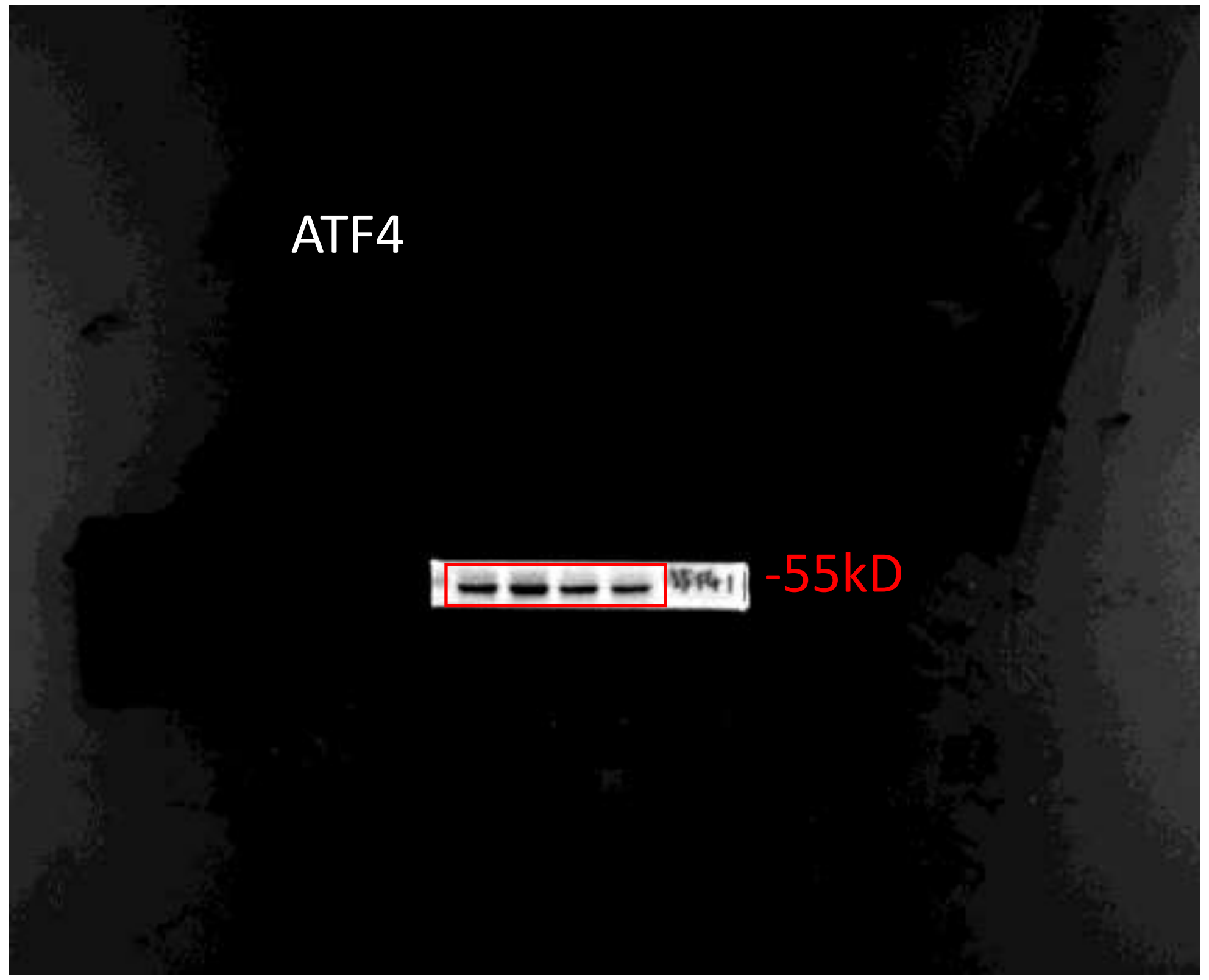
ATF4
-55kD

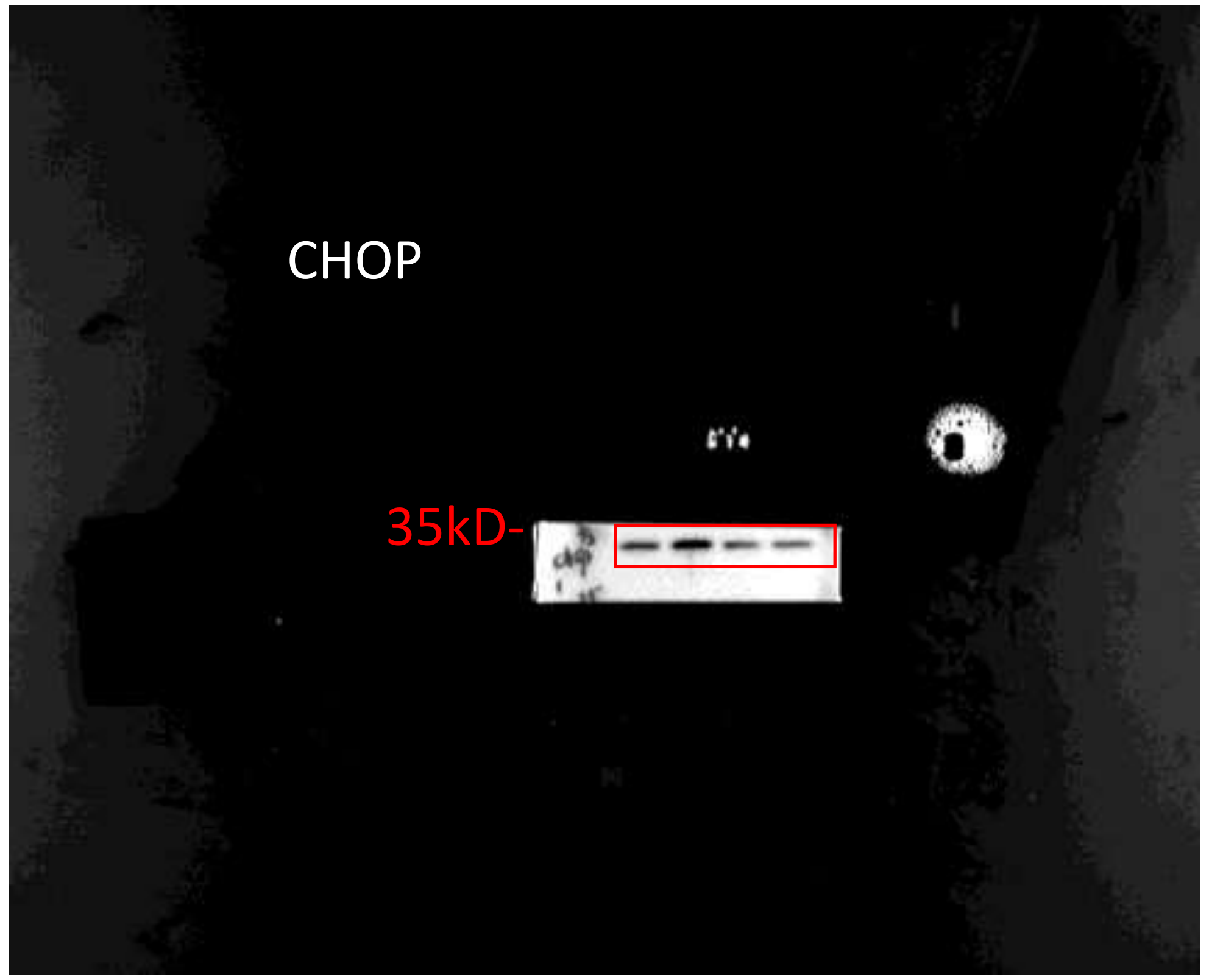
CHOP
35kD-

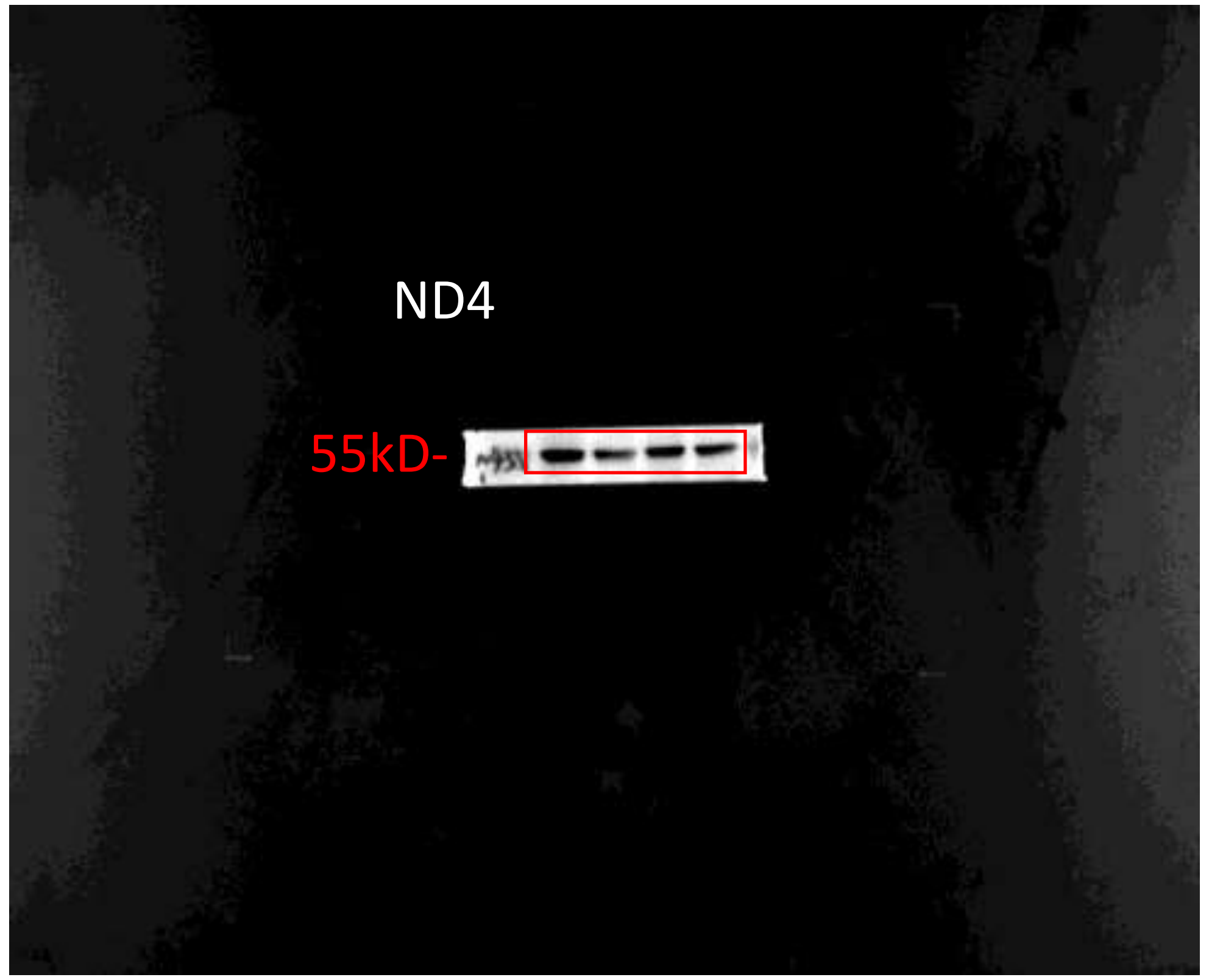
ND4
55kD-

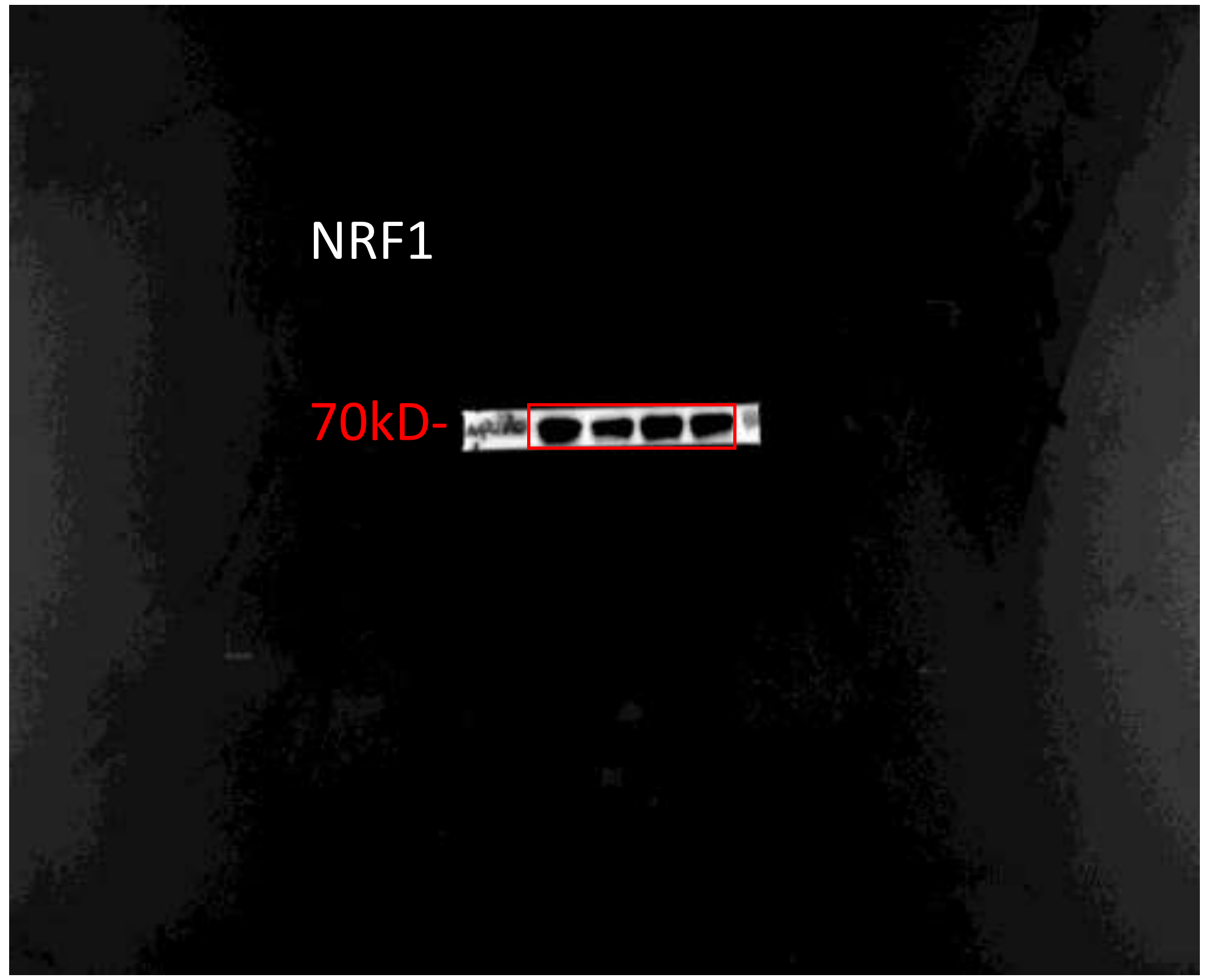
NRF1
70kD-

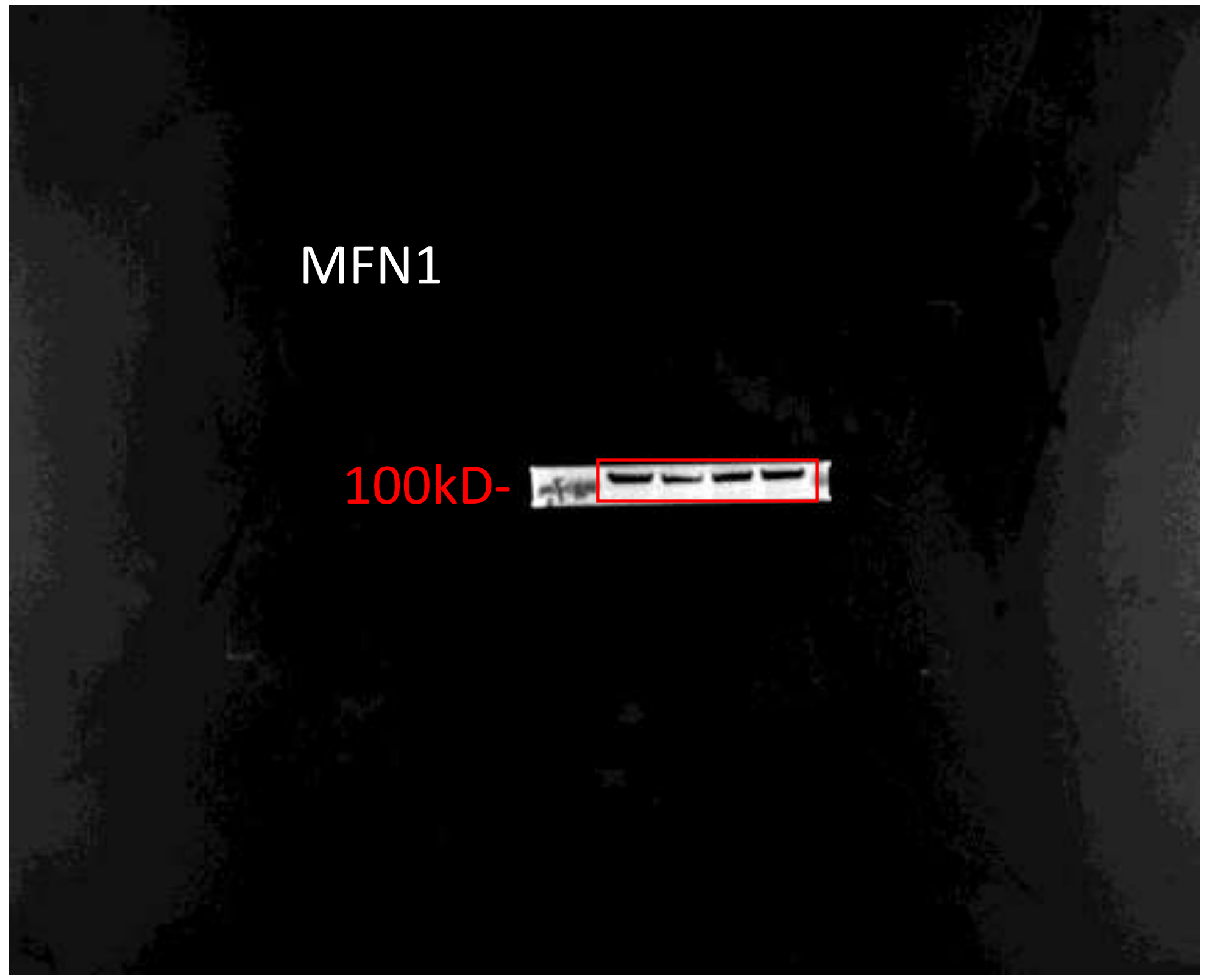
MFN1
100kD-
