# Supplementary material for: Fructose induces PERK-dependent ER stress and mitochondrial dysfunction via nucleotide depletion in clear cell renal cell carcinoma
Source: Cell Death Dis. 2026 Aug 7;17(1):766. doi: 10.1038/s41419-026-09157-3 (PMC13530255; doi:10.1038/s41419-026-09157-3)
Supplement: Supplementary file 3 [file 41419_2026_9157_MOESM2_ESM.docx]

**Supplementary Tables**

**Supplementary Table 1.** Antibodies information for western blot.

| **Antibody** | **Origin** | **Vendor** | **Catalog No.** |
| --- | --- | --- | --- |
| GLUT2 | Rabbit | ABclonal | A9843 |
| GLUT5 | Mouse | Santa Cruz Biotechnology | sc-271055 |
| KHK | Rabbit | Proteintech | 15681-1-AP |
| ALDOB | Rabbit | Proteintech | 18065-1-AP |
| DAK | Rabbit | Proteintech | 12224-1-AP |
| BID | Rabbit | Cell signaling technology | 2002T |
| Caspase 3 | Rabbit | Abmart | T40044 |
| ATF4 | Mouse | Santa Cruz Biotechnology | sc-390063 |
| CHOP | Mouse | Cell signaling technology | 2895S |
| BIP | Rabbit | Proteintech | 11587-1-AP |
| ATF6 | Rabbit | Proteintech | 24169-1-AP |
| XBP1S | Rabbit | Proteintech | 24868-1-AP |
| GAPDH | Mouse | Proteintech | 60004-1-lg |
| α-tubulin | Rabbit | Proteintech | 11224-1-AP |
| ND2 | Rabbit | Proteintech | 19704-1-AP |
| ND4 | Rabbit | ABclonal | A9941 |
| ND5 | Rabbit | ABclonal | A17972 |
| ND6 | Rabbit | ABclonal | A17991 |
| SDHB | Rabbit | ABclonal | A10821 |
| UQCRC2 | Rabbit | ABclonal | A4181 |
| COX1 | Rabbit | ABclonal | A23123 |
| ATP6 | Rabbit | ABclonal | A8193 |
| ATP8 | Rabbit | ABclonal | A17890 |
| ATP5A1 | Rabbit | ABclonal | A5884 |
| PGC-1α | Rabbit | Cell signaling technology | 2178T |
| NRF1 | Rabbit | ABclonal | A5547 |
| DRP1 | Rabbit | YAMEI | R015603 |
| FIS1 | Rabbit | ABclonal | A5821 |
| MFN1 | Rabbit | Immunoway | YN3054 |
| OPA1 | Rabbit | ABclonal | A9833 |
| p-PERK | Rabbit | zenbio | 340846 |
| PERK | Rabbit | YAMEI | R011978 |
| p-eIF2S1(Ser51） | Rabbit | proteintech | 287401-AP |
| eIF2S1 | Rabbit | proteintech | 11701-1-AP |
| ATF6 | Mouse | proteintech | 65563-1-Ig |
| β-actin | Mouse | proteintech | 66009-1-Ig |

**Supplementary Table 2.** Primers design for Real-Time PCR (human).

| **Gene name** | **Forward** | **Reverse** |
| --- | --- | --- |
| *MT-ND2* | ACTGCGCTAAGCTCGCACTG | ATTATGGATGCGGTTGCTTG |
| *MT-ND4* | ACAAGCTCCATCTGCCTACG | TTATGAGAATGACTGCGCCG |
| *MT-ND4L* | TATCGCTCACACCTCATATC | AGGCGGCAAAGACTAGTATG |
| *MT-ND5* | GGTTTCATCCTCGCCTTAGC | ACCTAATTGGGCTGATTTGC |
| *MT-ND6* | ATTGGTGCTGTGGGTGAAAG | GGATCCTCCCGAATCAACCC |
| *NDUFA1* | GCGTACATCCACAGGTTCACT | GCGCCTATCTCTTTCCATCAGA |
| *NDUFA2* | GGGACTTCATTGAGAAACGCT | AGCATTCGCGGATTAGGATGG |
| *NDUFB7* | TCCGGGTAGGAGCTAGGTG | CACCATCTCGCGCTCC |
| *NDUFAF8* | CGCTGCGGCCAAGAA | TTCGCCATTCCCACTTCCTT |
| *UQCC2* | TCAGATGTACGAGAGCTTAGCG | TGTACTCTTCCAACGACAGGC |
| *UQCC3* | AAATGCTGATCTCAGTCGCAA | CCGCCAACCATCCAGTTCT |
| *UQCR10* | ATCGTGGGCGTCATGTTCTTC | ATGTGGTCGTAGATAGCGTCC |
| *UQURQ* | ATCCGCACGTCTTCACTAAAG | TGGATCTCTCGAACTCTTCAGTC |
| *MT-CO1* | TTCGCCGACCGTTGACTATT | TTGTTTATGCGGGGAAACGC |
| *COX5B* | TGTGAAGAGGACAATACCAGCG | CCAGCTTGTAATGGGCTCCAC |
| *COX7C* | GGTCCGTAGGAGCCACTATGA | GTGTCTTACTACAAGGAAGGGTG |
| *COX14* | GCAGTGTCCGAGTCTACCAC | GTGGCAGGTGAAGTCTCTCC |
| *ATP5FIE* | GGCTGGACTCAGCTACATCC | TTACGTTGCTGCCAGAAGTCT |
| *ATP5MPL* | CTGCGCCAAGGGAGCGTA | CATAACGTGGAGGGCAGTGT |
| *MT-ATP6* | CCCACTTCTTACCACAAGGC | GTAGGTGGCCTGCAGTAATG |
| *MT-ATP8* | ATGCCCCAACTAAATACT | TTGTGGGGGCAATGAATG |
| *ATF4* | GAGCCTCGTTCTTCTTTTCC | GAGCCTCGTTCTTCTTTTCC |
| *CHOP* | ATGGCAGCTGAGTCATTGCCTTTC | AGAAGCAGGGTCAAGAGTGGTGAA |
| *18S* | GGCCCTGTAATTGGAATGAGTC | CCAAGATCCAACTACGAGCTT |

**Supplementary Table 3.** Primers used in mtDNA copy number assay (human).

| **Gene name** | **Forward** | **Reverse** |
| --- | --- | --- |
| *MT-ND1* | CACTTTCCACACAGACATCA | TGGTTAGGCTGGTGTTAGGG |
| *B2M* | TGTTCCTGCTGGGTAGCTCT | CCTCCATGATGCTGCTTACA |

**Supplementary Table 4.** Patients’ information.

| Number | Age | Gender | Race | Subtype | Location | Stage | Node Status |
| --- | --- | --- | --- | --- | --- | --- | --- |
| 1 | 64 | M | Asian | ccRCC | Left kidney | II | N0 |
| 2 | 75 | M | Asian | ccRCC | Right kidney | II | N_X_ |
| 3 | 74 | M | Asian | ccRCC | Left kidney | II | N_X_ |
| 4 | 37 | M | Asian | ccRCC | Left kidney | II-III | N_X_ |
| 5 | 77 | M | Asian | ccRCC | Left kidney | II | N0 |
| 6 | 65 | M | Asian | ccRCC | Left kidney | IV | N1 |
| 7 | 65 | M | Asian | ccRCC | Right kidney | II-III | N0 |
| 8 | 57 | M | Asian | ccRCC | Left kidney | III- IV | N_X_ |
| 9 | 56 | F | Asian | ccRCC | Right kidney | II | N_X_ |
| 10 | 53 | F | Asian | ccRCC | Left kidney | II | N_X_ |
| 11 | 58 | M | Asian | ccRCC | Right kidney | II | N_X_ |
| 12 | 53 | M | Asian | ccRCC | Left kidney | II | N0 |
| 13 | 59 | M | Asian | ccRCC | Right kidney | III | N_X_ |
| 14 | 62 | M | Asian | ccRCC | Left kidney | II | N0 |
| 15 | 60 | F | Asian | ccRCC | Left kidney | II | N0 |
| 16 | 59 | F | Asian | ccRCC | Left kidney | II | N0 |
| 17 | 52 | M | Asian | ccRCC | Left kidney | II | N0 |
| 18 | 45 | M | Asian | ccRCC | Left kidney | II | N0 |
| 19 | 49 | F | Asian | ccRCC | Left kidney | II | N_X_ |
| 20 | 72 | F | Asian | ccRCC | Right kidney | III | N_X_ |
| 21 | 67 | M | Asian | ccRCC | Right kidney | II | N0 |
| 22 | 65 | M | Asian | ccRCC | Right kidney | II | N0 |
| 23 | 65 | M | Asian | ccRCC | Left kidney | II | N0 |
| 24 | 52 | M | Asian | ccRCC | Left kidney | II | N0 |

**Supplementary Table 5.** Differentially expressed genes (DEGs) identified by RNA-seq analysis in 786-O cells treated with 1G+1F vs. 2G (|log₂FC| > 1, *P* < 0.05).

| #ID | Symbol | 2G-1_Count | 2G-2_Count | 2G-3_Count | 1G+1F-1_Count | 1G+1F-2_Count | 1G+1F-3_Count | 2G-1_FPKM | 2G-2_FPKM | 2G-3_FPKM | 1G+1F-1_FPKM | 1G+1F-2_FPKM | 1G+1F-3_FPKM | FDR | log_2_FC | regulated |
| --- | --- | --- | --- | --- | --- | --- | --- | --- | --- | --- | --- | --- | --- | --- | --- | --- |
| ENSG00000270136 | MINOS1-NBL1 | 268 | 274 | 254 | 10 | 41 | 60 | 8.11582 | 8.902326 | 8.108969 | 0.296291 | 1.290883 | 1.942717 | 7.29E-11 | -2.762646737 | down |
| ENSG00000270800 | RPS10-NUDT3 | 335 | 368 | 527 | 128 | 57 | 47 | 2.617504 | 2.616459 | 3.572028 | 1.573591 | 0.50542 | 0.472723 | 1.12E-08 | -2.245990474 | down |
| ENSG00000283782 | AC116366.3 | 128 | 90 | 183 | 8 | 19 | 56 | 0.401085 | 0.297589 | 0.596802 | 0.026886 | 0.060024 | 0.189897 | 0.000184666 | -2.190068571 | down |
| ENSG00000233927 | RPS28 | 1417 | 1652 | 951 | 298 | 219 | 405 | 26.612322 | 32.579342 | 18.521376 | 5.970249 | 4.288474 | 8.217517 | 6.75E-15 | -2.022457974 | down |
| ENSG00000161677 | JOSD2 | 316 | 343 | 285 | 47 | 66 | 140 | 8.815715 | 9.388435 | 7.765857 | 1.283116 | 1.794737 | 4.107878 | 5.02E-07 | -1.816142974 | down |
| ENSG00000265972 | TXNIP | 872 | 789 | 687 | 154 | 236 | 254 | 7.41852 | 7.055012 | 6.08097 | 1.436453 | 2.105178 | 2.325063 | 1.22E-16 | -1.760610326 | down |
| ENSG00000125995 | ROMO1 | 924 | 1258 | 890 | 312 | 248 | 279 | 51.146877 | 73.323904 | 50.518253 | 17.945199 | 14.045343 | 16.395172 | 1.48E-14 | -1.755947439 | down |
| ENSG00000130748 | TMEM160 | 289 | 446 | 288 | 74 | 81 | 137 | 10.839547 | 17.585405 | 11.185134 | 2.933397 | 3.156161 | 5.540548 | 2.15E-07 | -1.720070126 | down |
| ENSG00000178922 | HYI | 554 | 729 | 482 | 123 | 140 | 256 | 15.805262 | 22.310181 | 13.082859 | 3.20323 | 4.270212 | 7.505919 | 1.42E-08 | -1.679178226 | down |
| NewGene_1445 | NewGene_1445 | 3523 | 6280 | 3383 | 1061 | 1216 | 1657 | 59.188953 | 110.746567 | 58.933544 | 19.021935 | 21.373001 | 30.083227 | 0.000345462 | -1.658632317 | down |
| ENSG00000229833 | PET100 | 256 | 304 | 202 | 70 | 63 | 97 | 9.442372 | 11.965963 | 8.380951 | 2.832855 | 3.230004 | 4.013425 | 6.65E-07 | -1.624414572 | down |
| ENSG00000197956 | S100A6 | 14369 | 19287 | 13603 | 3571 | 4095 | 7283 | 540.738572 | 761.196602 | 529.293534 | 143.540835 | 161.313402 | 295.423883 | 0.000163694 | -1.575644423 | down |
| ENSG00000130332 | LSM7 | 1297 | 1561 | 1150 | 328 | 360 | 571 | 60.572307 | 76.140469 | 54.72079 | 16.199441 | 16.692789 | 28.897131 | 2.29E-12 | -1.575469199 | down |
| ENSG00000198258 | UBL5 | 2641 | 3451 | 2397 | 761 | 677 | 1239 | 148.523689 | 203.638703 | 139.419199 | 45.546996 | 40.421355 | 75.076048 | 9.23E-12 | -1.570565888 | down |
| ENSG00000255339 | AL133352.1 | 135 | 169 | 156 | 41 | 28 | 77 | 2.591254 | 3.550303 | 3.226672 | 0.842409 | 0.528144 | 1.623068 | 0.0002796 | -1.558939925 | down |
| ENSG00000196754 | S100A2 | 1096 | 1431 | 1100 | 272 | 339 | 557 | 31.386746 | 40.484239 | 30.000677 | 7.748568 | 9.679196 | 16.413638 | 3.87E-10 | -1.54615281 | down |
| ENSG00000137818 | RPLP1 | 17831 | 23335 | 15566 | 4250 | 4666 | 9504 | 374.789008 | 531.481247 | 355.453633 | 95.220803 | 110.247209 | 236.884491 | 0.001570132 | -1.542129271 | down |
| ENSG00000124116 | WFDC3 | 137 | 208 | 131 | 36 | 45 | 74 | 3.393333 | 5.437865 | 3.363182 | 0.942841 | 1.518133 | 1.986364 | 0.000231032 | -1.531854261 | down |
| ENSG00000134825 | TMEM258 | 1122 | 1445 | 978 | 307 | 401 | 454 | 48.227917 | 65.42859 | 43.550765 | 13.55834 | 17.478518 | 21.00826 | 5.39E-12 | -1.51044329 | down |
| ENSG00000152082 | MZT2B | 2079 | 2795 | 1744 | 506 | 598 | 1093 | 58.478537 | 81.860535 | 50.75904 | 14.905014 | 17.824766 | 31.735833 | 0.001292908 | -1.509550126 | down |
| ENSG00000259332 | ST20-MTHFS | 71 | 72 | 119 | 26 | 24 | 35 | 2.041289 | 2.18718 | 3.561062 | 0.825107 | 0.769809 | 1.072219 | 0.002036873 | -1.50520348 | down |
| ENSG00000173237 | C11orf86 | 201 | 231 | 199 | 47 | 49 | 116 | 4.210865 | 5.086336 | 4.314308 | 1.05225 | 1.072075 | 2.611856 | 0.000133413 | -1.48541814 | down |
| ENSG00000100300 | TSPO | 4922 | 6192 | 4326 | 1127 | 1470 | 2717 | 139.526094 | 182.31346 | 122.685468 | 33.724892 | 43.543272 | 81.456362 | 0.00190385 | -1.458789555 | down |
| ENSG00000205544 | TMEM256 | 393 | 613 | 408 | 119 | 137 | 229 | 20.064884 | 32.908259 | 21.60972 | 6.419613 | 7.279457 | 12.618783 | 2.62E-06 | -1.457016183 | down |
| ENSG00000196683 | TOMM7 | 2609 | 3063 | 2306 | 551 | 766 | 1440 | 84.441742 | 104.642751 | 77.262182 | 19.138363 | 26.070182 | 50.318453 | 0.002417835 | -1.453241178 | down |
| ENSG00000122971 | ACADS | 335 | 440 | 356 | 87 | 107 | 198 | 4.268795 | 5.892662 | 4.710441 | 1.180888 | 1.421672 | 2.732261 | 7.02E-06 | -1.441318988 | down |
| ENSG00000171224 | FAM241B | 351 | 363 | 291 | 79 | 120 | 149 | 8.212812 | 8.931575 | 7.043042 | 1.967141 | 2.915083 | 3.750827 | 6.29E-07 | -1.431041724 | down |
| ENSG00000103024 | NME3 | 68 | 115 | 66 | 29 | 30 | 27 | 1.731684 | 3.130708 | 1.666064 | 0.816584 | 0.7308 | 1.056403 | 0.005162524 | -1.426473377 | down |
| ENSG00000184990 | SIVA1 | 1476 | 1980 | 1457 | 435 | 467 | 818 | 44.95995 | 63.759502 | 46.392131 | 13.803085 | 14.713499 | 26.805916 | 5.11E-09 | -1.424435567 | down |
| ENSG00000112306 | RPS12 | 21486 | 27089 | 20095 | 5987 | 5907 | 12394 | 848.494324 | 1122.756226 | 822.838562 | 252.451111 | 244.022858 | 528.879944 | 0.001675396 | -1.413767376 | down |
| ENSG00000141552 | ANAPC11 | 1617 | 2169 | 1503 | 422 | 557 | 913 | 59.491731 | 82.695964 | 57.025527 | 16.101311 | 18.305932 | 34.684087 | 4.49E-08 | -1.39901699 | down |
| ENSG00000174917 | C19orf70 | 757 | 1111 | 724 | 224 | 215 | 503 | 22.396209 | 34.628353 | 21.730839 | 7.031092 | 6.659162 | 16.517772 | 1.52E-05 | -1.38069583 | down |
| ENSG00000172366 | MCRIP2 | 402 | 541 | 301 | 106 | 124 | 221 | 10.065689 | 14.243725 | 7.925029 | 2.829018 | 3.220775 | 5.899155 | 4.04E-05 | -1.379733 | down |
| ENSG00000164405 | UQCRQ | 2028 | 2936 | 2061 | 818 | 626 | 1076 | 65.930786 | 101.902978 | 68.396341 | 26.589975 | 20.485732 | 38.408497 | 5.12E-09 | -1.377681757 | down |
| ENSG00000090971 | NAT14 | 137 | 129 | 87 | 24 | 35 | 69 | 2.396095 | 2.260276 | 1.551951 | 0.462348 | 0.748759 | 1.261735 | 0.003770514 | -1.377138723 | down |
| ENSG00000066379 | ZNRD1 | 400 | 552 | 443 | 134 | 138 | 233 | 12.233838 | 17.83623 | 14.223959 | 4.251198 | 4.513936 | 7.631655 | 1.06E-06 | -1.370774542 | down |
| ENSG00000177700 | POLR2L | 3034 | 3831 | 2686 | 905 | 1057 | 1513 | 84.755882 | 112.297638 | 77.789215 | 26.970825 | 30.87146 | 45.666328 | 5.45E-11 | -1.364306901 | down |
| NewGene_1007 | NewGene_1007 | 60 | 77 | 71 | 24 | 24 | 27 | 4.456851 | 6.023502 | 5.534141 | 1.85217 | 1.849301 | 2.188382 | 0.007133209 | -1.358538706 | down |
| ENSG00000198918 | RPL39 | 7785 | 10889 | 7166 | 3052 | 2759 | 3572 | 483.769409 | 710.177246 | 461.709564 | 202.470016 | 179.367279 | 239.863556 | 1.01E-11 | -1.356383069 | down |
| ENSG00000171858 | RPS21 | 4935 | 6700 | 4376 | 1941 | 1707 | 2170 | 348.381207 | 497.339155 | 318.244707 | 147.773759 | 123.219192 | 163.564874 | 1.98E-11 | -1.353061786 | down |
| NewGene_7115 | NewGene_7115 | 94 | 90 | 87 | 32 | 28 | 38 | 5.356108 | 5.380292 | 5.151172 | 1.93667 | 1.617602 | 2.31932 | 0.002189722 | -1.350769852 | down |
| ENSG00000119632 | IFI27L2 | 477 | 649 | 453 | 122 | 170 | 296 | 23.226074 | 33.183224 | 22.866604 | 6.318435 | 8.63313 | 15.579406 | 1.90E-05 | -1.343332104 | down |
| ENSG00000178449 | COX14 | 310 | 384 | 373 | 115 | 108 | 172 | 10.408524 | 13.632288 | 13.262898 | 4.191212 | 3.94372 | 6.226693 | 1.32E-06 | -1.328434236 | down |
| ENSG00000243449 | C4orf48 | 155 | 245 | 161 | 64 | 69 | 76 | 8.630021 | 14.322756 | 9.266134 | 3.73795 | 3.999768 | 4.564575 | 0.000262067 | -1.321219714 | down |
| ENSG00000164587 | RPS14 | 29372 | 35965 | 26983 | 7728 | 9704 | 17654 | 1196.025651 | 1537.481081 | 1143.665006 | 343.04307 | 406.534495 | 774.866891 | 0.002442225 | -1.312490501 | down |
| ENSG00000125652 | ALKBH7 | 605 | 902 | 639 | 173 | 221 | 427 | 11.665415 | 18.393503 | 12.819049 | 3.552187 | 4.502802 | 8.986207 | 3.04E-05 | -1.30767208 | down |
| ENSG00000198931 | APRT | 1698 | 2403 | 1676 | 499 | 551 | 1177 | 49.510297 | 73.115056 | 49.605017 | 15.247272 | 16.341703 | 38.509064 | 0.008430797 | -1.29703225 | down |
| ENSG00000196976 | LAGE3 | 162 | 241 | 174 | 51 | 53 | 118 | 4.24632 | 6.617387 | 4.719239 | 1.42157 | 1.448895 | 3.335613 | 0.001395849 | -1.294757443 | down |
| ENSG00000213347 | MXD3 | 428 | 667 | 622 | 133 | 173 | 358 | 6.074364 | 9.103471 | 8.231237 | 2.257583 | 2.7778 | 5.113435 | 0.000128084 | -1.291261133 | down |
| ENSG00000135441 | BLOC1S1 | 679 | 1047 | 585 | 252 | 245 | 394 | 26.455492 | 43.457017 | 24.293669 | 10.731154 | 10.123908 | 16.840031 | 9.56E-06 | -1.284243184 | down |
| ENSG00000141933 | TPGS1 | 180 | 220 | 145 | 68 | 31 | 112 | 4.019435 | 5.148402 | 3.354718 | 1.612934 | 0.712512 | 2.698085 | 0.004927947 | -1.273108586 | down |
| ENSG00000103254 | FAM173A | 284 | 316 | 257 | 54 | 103 | 179 | 7.199033 | 7.932481 | 6.581295 | 1.311182 | 2.527221 | 4.542997 | 0.000712497 | -1.270939071 | down |
| ENSG00000224877 | NDUFAF8 | 577 | 673 | 467 | 177 | 161 | 333 | 23.345772 | 28.611621 | 19.56395 | 7.587134 | 6.761324 | 14.52641 | 2.06E-05 | -1.263331555 | down |
| ENSG00000125356 | NDUFA1 | 1198 | 1298 | 977 | 454 | 376 | 509 | 38.951996 | 44.287872 | 32.945984 | 15.751241 | 12.781571 | 17.866179 | 1.21E-09 | -1.261436388 | down |
| ENSG00000124172 | ATP5F1E | 2835 | 3569 | 2326 | 1119 | 989 | 1273 | 19.470892 | 26.150435 | 17.123584 | 8.27076 | 8.380281 | 10.15296 | 1.17E-09 | -1.260358605 | down |
| ENSG00000128228 | SDF2L1 | 526 | 673 | 507 | 135 | 199 | 340 | 15.12515 | 20.323467 | 15.140457 | 4.12904 | 5.97848 | 10.570174 | 3.95E-05 | -1.257136134 | down |
| NewGene_3859 | NewGene_3859 | 155 | 233 | 147 | 51 | 62 | 98 | 2.787285 | 4.761519 | 3.836797 | 0.928898 | 1.111632 | 2.005276 | 0.001225325 | -1.253829911 | down |
| ENSG00000164898 | FMC1 | 163 | 225 | 159 | 69 | 44 | 104 | 3.348755 | 4.855788 | 3.391364 | 1.496694 | 0.927589 | 2.308213 | 0.001884384 | -1.235090528 | down |
| ENSG00000139410 | SDSL | 219 | 333 | 168 | 54 | 92 | 145 | 3.852851 | 6.055395 | 2.997948 | 0.967364 | 1.652646 | 2.797839 | 0.002548396 | -1.232911277 | down |
| ENSG00000068079 | IFI35 | 464 | 653 | 483 | 148 | 195 | 299 | 9.334038 | 13.822758 | 10.099493 | 3.19652 | 4.124203 | 6.514733 | 1.81E-05 | -1.229040242 | down |
| ENSG00000269242 | AC010422.3 | 731 | 834 | 581 | 199 | 241 | 421 | 21.7321 | 26.014641 | 17.889105 | 6.305646 | 7.483336 | 13.511257 | 1.23E-05 | -1.228792145 | down |
| ENSG00000160050 | CCDC28B | 122 | 73 | 98 | 40 | 39 | 35 | 2.543629 | 1.60232 | 1.90329 | 0.742541 | 0.883808 | 0.6551 | 0.005310017 | -1.227150594 | down |
| ENSG00000128626 | MRPS12 | 1072 | 1394 | 957 | 301 | 387 | 694 | 23.596444 | 34.623516 | 21.91669 | 7.662274 | 8.805876 | 16.534304 | 1.29E-05 | -1.226150177 | down |
| NewGene_2495 | NewGene_2495 | 492 | 519 | 429 | 167 | 136 | 272 | 17.341451 | 21.567241 | 16.325074 | 6.126665 | 4.762462 | 10.284363 | 1.29E-05 | -1.222572428 | down |
| ENSG00000002330 | BAD | 909 | 1229 | 740 | 260 | 347 | 565 | 23.414317 | 30.817855 | 19.138393 | 6.831015 | 9.125151 | 15.865274 | 2.57E-05 | -1.21369689 | down |
| ENSG00000168101 | NUDT16L1 | 787 | 838 | 747 | 196 | 218 | 560 | 14.212057 | 15.700599 | 14.162095 | 3.838889 | 4.109695 | 11.04172 | 0.00024798 | -1.203285245 | down |
| ENSG00000129932 | DOHH | 545 | 532 | 430 | 134 | 209 | 271 | 6.684019 | 6.843171 | 5.459199 | 1.749818 | 2.681427 | 3.582224 | 7.91E-06 | -1.198609177 | down |
| ENSG00000083457 | ITGAE | 751 | 881 | 694 | 277 | 280 | 390 | 4.848159 | 5.967138 | 4.645878 | 1.908463 | 1.888914 | 2.719204 | 4.44E-08 | -1.191836879 | down |
| ENSG00000187608 | ISG15 | 428 | 477 | 395 | 128 | 172 | 233 | 16.530801 | 18.522382 | 15.798904 | 4.96652 | 6.882366 | 9.715044 | 6.08E-06 | -1.188024789 | down |
| ENSG00000123810 | B9D2 | 102 | 88 | 67 | 34 | 32 | 38 | 2.453006 | 2.239488 | 1.685398 | 0.8633 | 0.793855 | 0.974559 | 0.009787223 | -1.187652412 | down |
| ENSG00000156411 | ATP5MPL | 1713 | 1970 | 1548 | 522 | 645 | 987 | 61.137473 | 71.46309 | 55.964678 | 19.201128 | 22.234079 | 36.091281 | 7.78E-08 | -1.186614097 | down |
| ENSG00000173436 | MINOS1 | 2543 | 2904 | 2370 | 823 | 949 | 1447 | 17.073771 | 20.467047 | 16.493402 | 5.889126 | 6.664127 | 10.499332 | 5.39E-09 | -1.183608912 | down |
| ENSG00000213920 | MDP1 | 279 | 247 | 214 | 107 | 73 | 121 | 9.784533 | 8.823696 | 7.599006 | 3.871044 | 2.616146 | 4.458022 | 0.000184152 | -1.178885253 | down |
| ENSG00000105404 | RABAC1 | 1973 | 2543 | 1869 | 623 | 803 | 1234 | 60.133652 | 81.283159 | 59.213256 | 20.174341 | 25.501342 | 40.800206 | 6.29E-07 | -1.174654073 | down |
| ENSG00000232112 | TMA7 | 3450 | 4445 | 3217 | 1227 | 1344 | 2052 | 153.203583 | 207.217896 | 148.158493 | 58.164642 | 62.440468 | 98.484879 | 3.79E-08 | -1.171031255 | down |
| ENSG00000149806 | FAU | 7087 | 9896 | 6915 | 2731 | 3249 | 3953 | 211.626447 | 308.991267 | 212.430088 | 88.241401 | 100.802852 | 128.104168 | 2.55E-09 | -1.169112445 | down |
| ENSG00000186010 | NDUFA13 | 2366 | 3333 | 2205 | 903 | 1116 | 1272 | 69.781537 | 88.281495 | 57.384929 | 24.812234 | 29.916669 | 34.906379 | 3.88E-08 | -1.166247315 | down |
| ENSG00000129235 | TXNDC17 | 1880 | 2425 | 1959 | 651 | 713 | 1258 | 24.2055 | 33.546808 | 27.761978 | 8.278048 | 9.942763 | 17.290191 | 8.59E-07 | -1.165976151 | down |
| ENSG00000188290 | HES4 | 372 | 498 | 366 | 170 | 149 | 195 | 9.987229 | 13.997199 | 10.414573 | 4.744192 | 4.124742 | 5.497061 | 1.54E-05 | -1.15698523 | down |
| ENSG00000140365 | COMMD4 | 2077 | 2625 | 1923 | 639 | 812 | 1357 | 38.658693 | 74.926913 | 51.640269 | 16.089949 | 18.741391 | 34.528422 | 3.38E-06 | -1.15222419 | down |
| ENSG00000165644 | COMTD1 | 154 | 222 | 167 | 39 | 85 | 107 | 3.930564 | 5.945845 | 4.416516 | 1.053813 | 2.260094 | 2.93942 | 0.004400405 | -1.150613394 | down |
| ENSG00000149273 | RPS3 | 34430 | 42564 | 32749 | 12113 | 13439 | 21046 | 442.901801 | 861.179932 | 461.360729 | 217.391839 | 203.600721 | 338.511965 | 1.22E-08 | -1.14194264 | down |
| ENSG00000180879 | SSR4 | 2479 | 3126 | 2460 | 817 | 1074 | 1558 | 91.328873 | 120.328558 | 91.33416 | 33.969469 | 43.5653 | 64.130356 | 2.15E-07 | -1.134217468 | down |
| ENSG00000205220 | PSMB10 | 849 | 1116 | 802 | 300 | 329 | 555 | 17.367264 | 24.101644 | 17.047857 | 6.532644 | 7.031744 | 12.265655 | 1.23E-05 | -1.133717359 | down |
| ENSG00000054148 | PHPT1 | 2771 | 3680 | 2490 | 873 | 1097 | 1877 | 77.262008 | 107.733456 | 71.970604 | 26.004693 | 32.021363 | 56.472126 | 1.02E-05 | -1.133333093 | down |
| ENSG00000114395 | CYB561D2 | 542 | 557 | 467 | 143 | 177 | 354 | 10.190648 | 10.645287 | 8.522421 | 2.626471 | 3.335357 | 6.603564 | 0.000240384 | -1.128629099 | down |
| ENSG00000166595 | CIAO2B | 1561 | 2152 | 1485 | 468 | 682 | 1100 | 54.749441 | 79.201556 | 54.00554 | 18.383472 | 25.32004 | 42.203062 | 3.37E-05 | -1.127384893 | down |
| ENSG00000169689 | CENPX | 1131 | 1451 | 908 | 358 | 556 | 585 | 37.248516 | 49.946679 | 30.939775 | 12.600279 | 19.084592 | 20.763116 | 5.75E-06 | -1.12507485 | down |
| ENSG00000189171 | S100A13 | 2370 | 2903 | 2067 | 873 | 918 | 1357 | 98.853163 | 127.00249 | 89.840097 | 38.96866 | 39.810375 | 61.492776 | 7.78E-08 | -1.123468825 | down |
| ENSG00000204922 | UQCC3 | 450 | 553 | 353 | 125 | 189 | 273 | 5.431086 | 7.350992 | 4.605931 | 1.692732 | 2.471863 | 3.769572 | 0.000282911 | -1.121323809 | down |
| ENSG00000214309 | MBLAC1 | 102 | 111 | 87 | 33 | 51 | 44 | 1.685058 | 1.956222 | 1.520207 | 0.615296 | 0.884151 | 0.776755 | 0.008511196 | -1.120523024 | down |
| ENSG00000170889 | RPS9 | 16121 | 21299 | 14564 | 5448 | 7093 | 9978 | 470.887749 | 669.147933 | 450.695375 | 176.408708 | 218.012369 | 308.386468 | 2.72E-07 | -1.117628601 | down |
| ENSG00000187051 | RPS19BP1 | 2096 | 2726 | 1998 | 581 | 945 | 1450 | 57.341537 | 76.895868 | 55.727329 | 16.664355 | 26.540175 | 42.071478 | 2.93E-05 | -1.11574229 | down |
| NewGene_4715 | NewGene_4715 | 249 | 143 | 102 | 78 | 59 | 71 | 5.791177 | 3.338084 | 2.453851 | 1.750392 | 1.46403 | 1.930869 | 0.008181585 | -1.115543516 | down |
| ENSG00000117410 | ATP6V0B | 3062 | 3617 | 2732 | 897 | 1153 | 2058 | 65.210799 | 77.800977 | 55.556413 | 19.35843 | 23.023142 | 43.502687 | 1.15E-05 | -1.111321374 | down |
| ENSG00000129355 | CDKN2D | 384 | 344 | 260 | 91 | 89 | 254 | 7.22537 | 6.976966 | 4.893433 | 1.693212 | 1.627131 | 5.113329 | 0.004704432 | -1.103286027 | down |
| ENSG00000189143 | CLDN4 | 178 | 205 | 150 | 46 | 104 | 82 | 2.255177 | 2.769588 | 2.015312 | 0.537796 | 1.328317 | 1.139586 | 0.003683441 | -1.101770231 | down |
| ENSG00000099795 | NDUFB7 | 1147 | 1625 | 1039 | 445 | 518 | 702 | 53.815971 | 80.028175 | 50.534779 | 22.281292 | 25.42745 | 35.590988 | 5.90E-06 | -1.101055832 | down |
| ENSG00000134248 | LAMTOR5 | 3606 | 4109 | 3257 | 1235 | 1457 | 2092 | 127.820386 | 162.064489 | 127.237249 | 45.274926 | 54.58293 | 83.431347 | 1.54E-08 | -1.100268727 | down |
| ENSG00000131495 | NDUFA2 | 1264 | 1534 | 1161 | 454 | 492 | 785 | 40.686413 | 51.694172 | 38.952249 | 15.816662 | 17.410195 | 27.260406 | 1.64E-06 | -1.098240545 | down |
| ENSG00000131187 | F12 | 159 | 258 | 168 | 76 | 77 | 103 | 1.928237 | 3.292261 | 2.115022 | 0.980442 | 0.971372 | 1.341864 | 0.002273059 | -1.093614909 | down |
| ENSG00000051523 | CYBA | 5867 | 7476 | 5510 | 2064 | 2275 | 3971 | 182.182685 | 244.236097 | 177.080126 | 67.328048 | 73.183393 | 133.187039 | 2.28E-06 | -1.092951192 | down |
| ENSG00000168061 | SAC3D1 | 552 | 874 | 596 | 202 | 249 | 446 | 8.928328 | 15.192229 | 10.295843 | 3.555817 | 4.468079 | 7.747837 | 0.000393679 | -1.091791339 | down |
| ENSG00000184076 | UQCR10 | 2320 | 2926 | 2034 | 995 | 933 | 1248 | 77.420022 | 94.466797 | 64.946816 | 34.440252 | 30.599729 | 42.497738 | 6.73E-08 | -1.091499617 | down |
| ENSG00000244187 | TMEM141 | 6420 | 8572 | 5922 | 2213 | 2977 | 4053 | 208.290741 | 291.881622 | 199.221634 | 76.664093 | 101.039261 | 142.074265 | 7.63E-07 | -1.088549486 | down |
| ENSG00000135940 | COX5B | 1696 | 2308 | 1547 | 624 | 817 | 1008 | 59.350201 | 84.765152 | 56.129658 | 23.30373 | 29.901493 | 38.110596 | 1.32E-06 | -1.086624798 | down |
| ENSG00000148290 | SURF1 | 622 | 855 | 686 | 215 | 269 | 476 | 14.177431 | 20.457829 | 16.204388 | 5.211086 | 6.393577 | 11.706105 | 0.000138708 | -1.086260134 | down |
| ENSG00000099800 | TIMM13 | 1572 | 2000 | 1574 | 696 | 626 | 935 | 19.938441 | 26.503119 | 20.603253 | 9.379262 | 8.264858 | 12.758982 | 1.43E-07 | -1.084651843 | down |
| ENSG00000183172 | SMDT1 | 449 | 519 | 408 | 150 | 177 | 281 | 7.417908 | 8.726486 | 7.240804 | 2.960537 | 3.09611 | 7.482403 | 7.30E-05 | -1.083048811 | down |
| ENSG00000231500 | RPS18 | 36646 | 46399 | 34727 | 14627 | 14617 | 22815 | 1490.494328 | 1980.009738 | 1464.823242 | 635.188713 | 622.041343 | 1002.34596 | 4.44E-08 | -1.080554387 | down |
| ENSG00000137288 | UQCC2 | 1145 | 1661 | 1023 | 420 | 527 | 759 | 23.395926 | 33.549201 | 20.509177 | 8.49418 | 10.57603 | 16.146188 | 3.41E-05 | -1.078698426 | down |
| ENSG00000211450 | SELENOH | 1560 | 1848 | 1410 | 494 | 640 | 1012 | 43.671142 | 56.186639 | 42.081516 | 15.687643 | 18.045417 | 32.156618 | 7.91E-06 | -1.077174137 | down |
| ENSG00000187024 | PTRH1 | 298 | 253 | 220 | 95 | 117 | 126 | 5.816138 | 6.43746 | 6.475745 | 3.108902 | 2.551782 | 3.541932 | 0.000146997 | -1.076109787 | down |
| ENSG00000177600 | RPLP2 | 11398 | 15322 | 10689 | 5590 | 4757 | 6108 | 365.65193 | 515.047021 | 354.159183 | 191.001516 | 159.42762 | 211.647915 | 4.44E-08 | -1.075371984 | down |
| ENSG00000257704 | INAFM1 | 183 | 189 | 129 | 44 | 88 | 91 | 3.617092 | 3.922704 | 2.6521 | 0.922723 | 1.824539 | 1.936217 | 0.005410906 | -1.0725624 | down |
| ENSG00000176383 | B3GNT4 | 472 | 884 | 770 | 278 | 243 | 426 | 4.23325 | 8.570861 | 7.287145 | 2.738766 | 2.415135 | 4.42426 | 0.000338381 | -1.071344548 | down |
| ENSG00000229117 | RPL41 | 21087 | 25981 | 18252 | 8764 | 9317 | 10813 | 1061.107689 | 1380.504181 | 952.917465 | 464.098092 | 494.954727 | 586.911148 | 1.21E-09 | -1.070923824 | down |
| ENSG00000105372 | RPS19 | 17753 | 22784 | 16071 | 6727 | 7701 | 10803 | 210.134395 | 263.947452 | 187.336854 | 84.110487 | 96.923698 | 126.432869 | 7.76E-08 | -1.070707524 | down |
| ENSG00000107872 | FBXL15 | 193 | 207 | 165 | 62 | 98 | 90 | 2.387584 | 2.58629 | 1.840364 | 0.704553 | 1.060151 | 1.056948 | 0.001183699 | -1.070197052 | down |
| ENSG00000178896 | EXOSC4 | 910 | 1047 | 747 | 322 | 360 | 521 | 25.431262 | 30.790898 | 21.469365 | 9.495008 | 10.388091 | 15.742811 | 4.99E-06 | -1.070078646 | down |
| ENSG00000167644 | C19orf33 | 395 | 647 | 372 | 187 | 235 | 206 | 20.224331 | 35.177212 | 19.691357 | 10.431756 | 12.717527 | 11.570692 | 0.00035387 | -1.069017228 | down |
| ENSG00000130255 | RPL36 | 7496 | 10391 | 6895 | 3059 | 3488 | 4512 | 280.897856 | 431.499575 | 270.131789 | 129.432104 | 138.333811 | 184.79658 | 2.18E-07 | -1.06789668 | down |
| ENSG00000111325 | OGFOD2 | 314 | 303 | 242 | 79 | 97 | 210 | 4.798521 | 4.779981 | 3.971676 | 1.198437 | 1.477643 | 3.373635 | 0.003070511 | -1.067541341 | down |
| ENSG00000170619 | COMMD5 | 926 | 1116 | 876 | 313 | 329 | 669 | 16.215865 | 21.364531 | 16.437837 | 6.002712 | 6.355822 | 13.553552 | 0.000117545 | -1.066534223 | down |
| ENSG00000167969 | ECI1 | 1809 | 2276 | 1619 | 700 | 593 | 1265 | 36.066353 | 52.033102 | 34.494168 | 15.525179 | 11.819949 | 30.244357 | 5.79E-05 | -1.065816528 | down |
| ENSG00000117691 | NENF | 874 | 1135 | 923 | 322 | 406 | 585 | 22.933294 | 31.26449 | 25.120024 | 9.011498 | 11.140232 | 16.598965 | 6.69E-06 | -1.065100529 | down |
| ENSG00000177674 | AGTRAP | 364 | 385 | 290 | 117 | 154 | 192 | 8.285425 | 9.102729 | 6.771846 | 2.771994 | 3.617889 | 4.64995 | 0.000109078 | -1.064633971 | down |
| ENSG00000008382 | MPND | 358 | 323 | 304 | 105 | 129 | 206 | 6.258965 | 5.434614 | 5.667489 | 1.766371 | 2.272424 | 3.891538 | 0.000228242 | -1.061857216 | down |
| ENSG00000160813 | PPP1R35 | 797 | 861 | 750 | 312 | 317 | 442 | 19.245323 | 21.837742 | 18.783985 | 8.042708 | 8.004005 | 11.523094 | 2.11E-07 | -1.061810436 | down |
| ENSG00000197019 | SERTAD1 | 248 | 282 | 202 | 66 | 123 | 140 | 2.911676 | 3.48608 | 2.468741 | 0.82386 | 1.51453 | 1.781518 | 0.001694237 | -1.061266898 | down |
| ENSG00000173171 | MTX1 | 1056 | 1436 | 984 | 457 | 375 | 730 | 16.691125 | 27.013782 | 20.005867 | 7.993146 | 6.137598 | 12.496693 | 7.05E-05 | -1.059453911 | down |
| ENSG00000179886 | TIGD5 | 698 | 751 | 604 | 187 | 282 | 461 | 3.226152 | 3.639382 | 2.892127 | 0.919512 | 1.360111 | 2.300892 | 0.000217259 | -1.055490031 | down |
| ENSG00000240065 | PSMB9 | 1253 | 1668 | 1258 | 468 | 581 | 837 | 39.958337 | 55.783482 | 41.520897 | 15.905966 | 19.360561 | 28.804918 | 5.63E-06 | -1.055330113 | down |
| ENSG00000124074 | ENKD1 | 280 | 371 | 276 | 123 | 112 | 181 | 4.439779 | 6.122599 | 4.517487 | 2.108163 | 1.843432 | 3.062493 | 0.000439437 | -1.055168263 | down |
| ENSG00000115350 | POLE4 | 341 | 488 | 333 | 135 | 128 | 264 | 6.803732 | 10.225417 | 6.884833 | 2.867915 | 2.667418 | 5.681997 | 0.001392105 | -1.053525593 | down |
| ENSG00000129911 | KLF16 | 1056 | 1244 | 920 | 380 | 484 | 584 | 9.167105 | 11.308401 | 8.222708 | 3.70333 | 4.361082 | 7.247258 | 8.42E-07 | -1.052795963 | down |
| ENSG00000125835 | SNRPB | 10777 | 12182 | 9371 | 3668 | 4095 | 6857 | 243.178309 | 284.221729 | 215.603798 | 86.398835 | 95.052619 | 164.697689 | 8.61E-07 | -1.052209936 | down |
| ENSG00000116586 | LAMTOR2 | 1086 | 1299 | 1008 | 451 | 448 | 628 | 41.305224 | 51.848577 | 39.806321 | 18.291597 | 17.816477 | 25.790182 | 4.84E-07 | -1.047762557 | down |
| ENSG00000163344 | PMVK | 874 | 1072 | 806 | 303 | 386 | 560 | 17.007042 | 21.898243 | 16.250671 | 6.286483 | 7.842103 | 11.761292 | 1.52E-05 | -1.046465556 | down |
| ENSG00000125877 | ITPA | 1253 | 1374 | 1069 | 357 | 503 | 825 | 26.341836 | 30.475548 | 23.297928 | 8.331072 | 11.051224 | 19.075498 | 6.07E-05 | -1.045892451 | down |
| ENSG00000106153 | CHCHD2 | 11905 | 13964 | 10291 | 3765 | 4687 | 8043 | 329.234467 | 405.314362 | 295.109528 | 111.189819 | 135.611633 | 240.367371 | 9.59E-06 | -1.045833452 | down |
| ENSG00000129354 | AP1M2 | 948 | 907 | 748 | 240 | 394 | 552 | 17.676804 | 19.727734 | 15.215575 | 4.979283 | 7.334718 | 10.365932 | 7.03E-05 | -1.042166545 | down |
| ENSG00000173660 | UQCRH | 5377 | 6245 | 4878 | 2308 | 2191 | 2945 | 220.308555 | 268.548392 | 207.254283 | 100.308559 | 93.698161 | 130.062693 | 2.25E-09 | -1.040854548 | down |
| ENSG00000135114 | OASL | 161 | 226 | 164 | 59 | 86 | 106 | 1.32934 | 2.268363 | 1.312838 | 0.476776 | 0.751212 | 0.989307 | 0.003405784 | -1.040268601 | down |
| ENSG00000179958 | DCTPP1 | 3360 | 4077 | 3203 | 1129 | 1545 | 2189 | 68.959323 | 87.371036 | 68.003135 | 24.860807 | 33.238197 | 48.742075 | 1.55E-06 | -1.03836967 | down |
| ENSG00000147403 | RPL10 | 30091 | 36764 | 28483 | 10284 | 13953 | 19345 | 452.183431 | 659.064682 | 416.451518 | 191.40188 | 233.936643 | 353.239755 | 3.70E-07 | -1.037846605 | down |
| ENSG00000086544 | ITPKC | 390 | 423 | 268 | 95 | 190 | 210 | 2.867642 | 3.263578 | 2.045083 | 0.739913 | 1.457957 | 1.66636 | 0.001606618 | -1.037074818 | down |
| ENSG00000172590 | MRPL52 | 1462 | 1779 | 1388 | 473 | 651 | 998 | 39.039991 | 48.590197 | 36.362781 | 12.835421 | 17.017321 | 27.535437 | 1.96E-05 | -1.036716532 | down |
| ENSG00000101084 | RAB5IF | 5499 | 6329 | 5195 | 1820 | 2244 | 3745 | 122.311465 | 147.116834 | 120.153513 | 42.93122 | 52.352828 | 91.526293 | 3.89E-06 | -1.034329226 | down |
| ENSG00000143977 | SNRPG | 2998 | 3745 | 2620 | 1482 | 1147 | 1602 | 119.333778 | 156.539955 | 108.038789 | 62.802875 | 47.604522 | 69.003325 | 9.55E-07 | -1.034294596 | down |
| ENSG00000105755 | ETHE1 | 268 | 326 | 289 | 103 | 104 | 197 | 6.897208 | 8.872281 | 7.73821 | 2.813191 | 2.788527 | 5.471218 | 0.001047341 | -1.032642067 | down |
| ENSG00000167779 | IGFBP6 | 1242 | 1486 | 1066 | 387 | 528 | 831 | 26.803592 | 33.861775 | 23.89168 | 8.884432 | 11.822791 | 19.382066 | 6.07E-05 | -1.032454897 | down |
| ENSG00000149541 | B3GAT3 | 963 | 1010 | 917 | 348 | 360 | 611 | 13.779671 | 15.84391 | 14.2026 | 5.61824 | 5.723915 | 9.884563 | 6.14E-06 | -1.031696494 | down |
| NewGene_1446 | NewGene_1446 | 2471 | 3430 | 2486 | 1091 | 1179 | 1568 | 21.434669 | 31.229931 | 22.363739 | 10.100721 | 10.700957 | 14.698828 | 6.17E-07 | -1.029052281 | down |
| ENSG00000196544 | BORCS6 | 238 | 255 | 185 | 65 | 85 | 163 | 2.294762 | 2.58056 | 1.8519 | 0.664929 | 0.855562 | 1.694233 | 0.005551188 | -1.028615221 | down |
| ENSG00000143947 | RPS27A | 27957 | 32329 | 25698 | 11568 | 10869 | 16904 | 699.211531 | 845.023419 | 691.147723 | 312.137299 | 291.68476 | 449.030075 | 2.95E-08 | -1.025724467 | down |
| ENSG00000172428 | COPS9 | 968 | 1245 | 779 | 442 | 445 | 477 | 57.620159 | 77.698373 | 47.943785 | 28.082155 | 27.642951 | 30.571206 | 1.26E-05 | -1.024452719 | down |
| ENSG00000137133 | HINT2 | 352 | 491 | 332 | 190 | 152 | 193 | 13.934074 | 20.431911 | 13.642736 | 8.038307 | 6.300545 | 8.240924 | 0.000340898 | -1.02361638 | down |
| ENSG00000042753 | AP2S1 | 4483 | 5444 | 4058 | 1766 | 2013 | 2640 | 123.020007 | 158.390844 | 115.714828 | 51.241606 | 57.820704 | 77.338082 | 4.49E-08 | -1.02359577 | down |
| ENSG00000204237 | OXLD1 | 337 | 358 | 336 | 107 | 136 | 234 | 10.524753 | 11.984323 | 10.787133 | 3.49099 | 4.119394 | 7.97222 | 0.000705929 | -1.017871985 | down |
| ENSG00000030110 | BAK1 | 1597 | 1766 | 1479 | 557 | 714 | 965 | 18.591763 | 21.597408 | 17.866964 | 6.911871 | 8.656373 | 12.14043 | 5.02E-07 | -1.016306571 | down |
| ENSG00000106927 | AMBP | 283 | 385 | 275 | 93 | 105 | 244 | 4.902981 | 7.008775 | 4.937666 | 1.723206 | 1.893301 | 4.577766 | 0.006315127 | -1.014708456 | down |
| ENSG00000011052 | NME1-NME2 | 3707 | 3242 | 2353 | 937 | 1652 | 1732 | 86.527519 | 76.336442 | 56.547886 | 24.64023 | 41.246008 | 42.921191 | 1.80E-05 | -1.007448682 | down |
| ENSG00000158716 | DUSP23 | 412 | 516 | 356 | 153 | 181 | 266 | 13.706969 | 17.843845 | 12.351181 | 5.491103 | 6.217207 | 9.723111 | 0.000368669 | -1.00288452 | down |
| ENSG00000111671 | SPSB2 | 353 | 337 | 318 | 120 | 162 | 185 | 6.908979 | 6.836382 | 6.488842 | 2.313125 | 3.208277 | 3.891499 | 7.30E-05 | -1.001989207 | down |
| ENSG00000204387 | C6orf48 | 1865 | 2084 | 1623 | 530 | 776 | 1319 | 62.497294 | 73.218325 | 56.450274 | 19.828182 | 28.47578 | 47.476116 | 0.000158774 | -1.001977212 | down |
| ENSG00000127184 | COX7C | 2274 | 2857 | 2148 | 1079 | 1098 | 1190 | 84.66741 | 111.60572 | 83.001676 | 42.130868 | 42.77831 | 47.221139 | 5.11E-08 | -1.001505355 | down |
| NewGene_5600 | NewGene_5600 | 142 | 150 | 182 | 79 | 49 | 90 | 1.728949 | 1.906763 | 2.27161 | 1.088907 | 0.61311 | 1.160353 | 0.006974472 | -1.000414003 | down |
| ENSG00000243716 | NPIPB5 | 739 | 550 | 628 | 1464 | 1224 | 768 | 8.034826 | 6.17301 | 6.648602 | 11.834428 | 10.575628 | 10.087058 | 0.000240384 | 1.000504203 | up |
| ENSG00000149548 | CCDC15 | 153 | 97 | 166 | 312 | 243 | 198 | 0.964998 | 0.640255 | 1.097536 | 2.11643 | 1.614307 | 1.360963 | 0.002565711 | 1.005845737 | up |
| ENSG00000120685 | PROSER1 | 884 | 717 | 906 | 1792 | 1616 | 1207 | 4.25864 | 3.623656 | 4.529678 | 9.221833 | 8.151247 | 6.286806 | 3.27E-06 | 1.022140643 | up |
| NewGene_4301 | NewGene_4301 | 182 | 170 | 261 | 358 | 452 | 332 | 1.934354 | 1.844478 | 2.957168 | 3.89953 | 5.349187 | 3.560806 | 0.000149777 | 1.026546004 | up |
| ENSG00000110675 | ELMOD1 | 68 | 48 | 62 | 131 | 96 | 103 | 0.5864 | 0.438339 | 0.823092 | 1.220564 | 0.869907 | 1.165042 | 0.009789333 | 1.028720321 | up |
| ENSG00000196081 | ZNF724 | 173 | 93 | 139 | 320 | 282 | 139 | 1.554203 | 0.876284 | 1.291494 | 3.07182 | 2.657761 | 1.34473 | 0.009239448 | 1.031809117 | up |
| ENSG00000285447 | ZNF883 | 137 | 122 | 128 | 243 | 252 | 236 | 1.652776 | 1.554086 | 1.607441 | 3.142813 | 3.196843 | 3.088564 | 9.34E-05 | 1.042172018 | up |
| ENSG00000005483 | KMT2E | 1746 | 1170 | 1798 | 3403 | 3207 | 2164 | 7.087248 | 4.87444 | 6.961123 | 13.900242 | 12.64479 | 8.912035 | 1.06E-05 | 1.044672473 | up |
| ENSG00000186566 | GPATCH8 | 694 | 414 | 602 | 1097 | 1158 | 978 | 3.677773 | 2.302775 | 3.309113 | 6.220985 | 6.423937 | 5.607253 | 8.76E-07 | 1.056004963 | up |
| ENSG00000154493 | C10orf90 | 125 | 137 | 128 | 311 | 282 | 144 | 1.941564 | 2.171903 | 1.072318 | 5.173297 | 2.387244 | 2.392458 | 0.003991574 | 1.061163717 | up |
| ENSG00000108684 | ASIC2 | 44 | 62 | 57 | 119 | 89 | 107 | 0.312473 | 0.466 | 0.420816 | 0.914939 | 0.667279 | 0.833509 | 0.009136603 | 1.07203667 | up |
| ENSG00000183354 | KIAA2026 | 596 | 445 | 659 | 1222 | 1225 | 791 | 2.188087 | 1.827316 | 2.630118 | 4.671376 | 4.840772 | 4.22021 | 1.55E-05 | 1.074220582 | up |
| ENSG00000256043 | CTSO | 97 | 52 | 81 | 191 | 143 | 112 | 0.812588 | 0.456552 | 0.706821 | 1.716936 | 1.260481 | 1.014877 | 0.005945209 | 1.108485294 | up |
| ENSG00000153790 | C7orf31 | 186 | 176 | 224 | 441 | 454 | 251 | 1.721226 | 1.422348 | 1.827779 | 3.851474 | 4.115811 | 2.375746 | 0.000362406 | 1.109778288 | up |
| NewGene_808 | NewGene_808 | 186 | 131 | 166 | 418 | 308 | 215 | 0.933894 | 0.729559 | 0.862506 | 2.066179 | 1.813071 | 1.245398 | 0.000860719 | 1.115084925 | up |
| NewGene_3774 | NewGene_3774 | 205 | 185 | 256 | 509 | 477 | 282 | 2.292737 | 1.913445 | 3.70456 | 5.577835 | 5.863436 | 3.628197 | 0.000284588 | 1.118713973 | up |
| ENSG00000145850 | TIMD4 | 56 | 74 | 71 | 169 | 115 | 115 | 1.038799 | 1.472066 | 1.33164 | 3.286775 | 2.234263 | 2.256266 | 0.004481659 | 1.12191261 | up |
| ENSG00000198315 | ZKSCAN8 | 770 | 559 | 902 | 1734 | 1619 | 1028 | 2.601067 | 1.980907 | 3.15776 | 6.257889 | 5.765804 | 3.752375 | 1.53E-05 | 1.123213177 | up |
| ENSG00000197372 | ZNF675 | 331 | 295 | 381 | 793 | 711 | 486 | 3.664314 | 3.487554 | 4.510903 | 9.177589 | 8.109616 | 5.787127 | 1.81E-05 | 1.125770966 | up |
| NewGene_4504 | NewGene_4504 | 95 | 54 | 83 | 176 | 183 | 99 | 1.052317 | 0.583001 | 1.040279 | 2.027288 | 2.153975 | 1.087843 | 0.005559155 | 1.130984004 | up |
| NewGene_4017 | NewGene_4017 | 594 | 385 | 1059 | 1306 | 1315 | 1466 | 3.989672 | 2.721226 | 7.403203 | 9.402988 | 9.265845 | 10.675278 | 7.51E-05 | 1.139447613 | up |
| ENSG00000169246 | NPIPB3 | 100 | 131 | 120 | 307 | 258 | 141 | 1.851654 | 2.783723 | 2.327127 | 6.117439 | 5.039373 | 2.934441 | 0.002480349 | 1.149712061 | up |
| NewGene_4038 | NewGene_4038 | 41 | 50 | 68 | 122 | 88 | 117 | 0.410047 | 0.45864 | 0.704917 | 1.150091 | 0.882723 | 1.170286 | 0.005039682 | 1.166409973 | up |
| ENSG00000162614 | NEXN | 1252 | 1043 | 1467 | 3229 | 2749 | 1667 | 10.091868 | 8.767833 | 13.54271 | 28.437955 | 24.94473 | 15.975635 | 7.80E-06 | 1.174587634 | up |
| ENSG00000138587 | MNS1 | 249 | 165 | 254 | 588 | 444 | 341 | 3.060477 | 2.129828 | 3.23476 | 7.724699 | 5.713531 | 4.530063 | 7.53E-05 | 1.191638237 | up |
| ENSG00000138336 | TET1 | 65 | 33 | 84 | 126 | 126 | 128 | 0.173247 | 0.09221 | 0.230958 | 0.359216 | 0.352032 | 0.369724 | 0.003664388 | 1.199604651 | up |
| ENSG00000185864 | NPIPB4 | 78 | 76 | 101 | 229 | 196 | 104 | 0.53692 | 0.547899 | 0.925079 | 1.768869 | 1.435202 | 0.949211 | 0.003011861 | 1.202613125 | up |
| NewGene_2755 | NewGene_2755 | 322 | 210 | 323 | 666 | 672 | 469 | 0.867037 | 0.593095 | 0.902221 | 1.916253 | 1.893996 | 1.364961 | 2.98E-06 | 1.223236363 | up |
| NewGene_3572 | NewGene_3572 | 59 | 72 | 88 | 182 | 171 | 116 | 0.38084 | 0.584831 | 0.589359 | 1.258285 | 1.154403 | 0.81157 | 0.001187707 | 1.234512345 | up |
| NewGene_3323 | NewGene_3323 | 258 | 298 | 366 | 865 | 705 | 407 | 2.035455 | 2.543565 | 3.240754 | 7.709424 | 6.396685 | 2.753795 | 7.59E-05 | 1.24830228 | up |
| ENSG00000198804 | MT-CO1 | 239672 | 234002 | 262018 | 698186 | 580075 | 314189 | 3873.162598 | 3968.86499 | 4390.54834 | 12047.94043 | 9807.008789 | 5486.62207 | 0.004400405 | 1.264772599 | up |
| NewGene_1299 | NewGene_1299 | 32 | 22 | 44 | 82 | 66 | 70 | 0.503543 | 0.356987 | 0.725682 | 1.380275 | 1.088537 | 1.199626 | 0.009211381 | 1.292148396 | up |
| NewGene_5092 | NewGene_5092 | 612 | 557 | 565 | 1685 | 1403 | 777 | 5.646334 | 5.637183 | 5.197872 | 15.808669 | 12.980986 | 7.855151 | 5.63E-06 | 1.305706988 | up |
| NewGene_4680 | NewGene_4680 | 31 | 21 | 26 | 62 | 59 | 59 | 0.31414 | 0.227031 | 0.362327 | 0.680553 | 0.640949 | 0.693479 | 0.009406718 | 1.336578098 | up |
| ENSG00000243660 | ZNF487 | 47 | 44 | 34 | 140 | 76 | 73 | 0.547727 | 0.534719 | 0.41396 | 1.759891 | 0.937327 | 0.930329 | 0.005519632 | 1.355067963 | up |
| ENSG00000198064 | NPIPB13 | 53 | 46 | 65 | 164 | 131 | 86 | 0.587097 | 0.34784 | 0.550911 | 1.406959 | 0.960519 | 0.960293 | 0.001395849 | 1.364500179 | up |
| ENSG00000185869 | ZNF829 | 75 | 45 | 45 | 123 | 163 | 102 | 0.375449 | 0.234088 | 0.231374 | 0.655286 | 0.853488 | 0.551106 | 0.000889759 | 1.366529492 | up |
| NewGene_4087 | NewGene_4087 | 54 | 39 | 47 | 173 | 111 | 50 | 2.476174 | 1.854413 | 2.249931 | 8.718662 | 5.375157 | 2.456858 | 0.007412092 | 1.421923241 | up |
| ENSG00000198899 | MT-ATP6 | 146314 | 145094 | 170257 | 548670 | 412840 | 180840 | 5353.935547 | 5572.294434 | 6459.943359 | 21438.29492 | 15804.16211 | 7150.643555 | 0.009542862 | 1.467353818 | up |
| NewGene_3813 | NewGene_3813 | 137 | 115 | 141 | 392 | 426 | 165 | 2.109727 | 1.849406 | 2.247794 | 6.457554 | 6.880347 | 2.745699 | 0.000102092 | 1.472471837 | up |
| ENSG00000198695 | MT-ND6 | 38401 | 42480 | 46966 | 172382 | 127481 | 49123 | 1822.681885 | 2116.179443 | 2311.499756 | 8736.916016 | 6330.282715 | 2519.549072 | 0.009034273 | 1.609927564 | up |
| NewGene_3533 | NewGene_3533 | 44 | 13 | 39 | 81 | 74 | 113 | 0.630033 | 0.135205 | 0.847788 | 1.202572 | 0.972553 | 1.375903 | 0.001922312 | 1.612859126 | up |
| ENSG00000198886 | MT-ND4 | 282093 | 263225 | 305123 | 1140528 | 848573 | 382389 | 5101.250977 | 4995.845215 | 5721.342285 | 22023.32422 | 16053.76856 | 7472.292969 | 0.003112839 | 1.640373983 | up |
| ENSG00000228253 | MT-ATP8 | 40907 | 38187 | 45197 | 170501 | 131722 | 49990 | 4924.380371 | 4824.766602 | 5641.632324 | 21916.96875 | 16589.17578 | 6502.958984 | 0.004870885 | 1.665982978 | up |
| ENSG00000198763 | MT-ND2 | 100987 | 100284 | 117405 | 431339 | 331761 | 143523 | 2415.071777 | 2517.073975 | 2911.310791 | 11014.79688 | 8300.302734 | 3708.944824 | 0.002694086 | 1.668007974 | up |
| NewGene_1843 | NewGene_1843 | 30 | 8 | 23 | 62 | 63 | 53 | 0.330151 | 0.093059 | 0.266069 | 0.741852 | 0.742322 | 0.641356 | 0.005133277 | 1.692467286 | up |
| ENSG00000145416 | 1-Mar | 26 | 13 | 16 | 71 | 52 | 39 | 0.109339 | 0.058672 | 0.072665 | 0.31824 | 0.248214 | 0.176188 | 0.005442104 | 1.71106051 | up |
| ENSG00000198786 | MT-ND5 | 104104 | 96200 | 124393 | 464067 | 344470 | 140413 | 1431.669678 | 1388.506714 | 1773.823608 | 6814.728027 | 4955.989258 | 2086.641357 | 0.003957473 | 1.711970559 | up |
| NewGene_852 | NewGene_852 | 50 | 42 | 53 | 244 | 111 | 91 | 0.72779 | 0.607604 | 0.729733 | 3.644134 | 1.553105 | 1.391998 | 0.000223029 | 1.786383701 | up |
| NewGene_7052 | NewGene_7052 | 11 | 9 | 17 | 36 | 52 | 43 | 0.229439 | 0.179615 | 0.379257 | 0.79521 | 1.304394 | 0.959988 | 0.00295371 | 1.946854162 | up |
| ENSG00000212907 | MT-ND4L | 33592 | 30074 | 37320 | 200060 | 128694 | 46241 | 2818.447266 | 2648.296387 | 3246.80835 | 17923.76758 | 11296.30273 | 4192.393066 | 0.002659972 | 2.065573294 | up |
| NewGene_6246 | NewGene_6246 | 94 | 148 | 94 | 449 | 624 | 625 | 0.446367 | 0.741602 | 0.465328 | 2.296657 | 3.128257 | 3.239047 | 6.91E-22 | 2.436281447 | up |
| NewGene_3660 | NewGene_3660 | 0 | 0 | 0 | 308 | 97 | 110 | 0 | 0 | 0 | 0.894442 | 0.274243 | 0.322868 | 1.12E-08 | 10.03614016 | up |
